# Systematic genetic and proteomic screens during gametogenesis identify H2BK34 methylation as an evolutionary conserved meiotic mark

**DOI:** 10.1186/s13072-020-00349-5

**Published:** 2020-09-15

**Authors:** Marion Crespo, Lacey J. Luense, Marie Arlotto, Jialei Hu, Jean Dorsey, Encar García-Oliver, Parisha P. Shah, Delphine Pflieger, Shelley L. Berger, Jérôme Govin

**Affiliations:** 1 Univ. Grenoble Alpes, CEA, Inserm, IRIG-BGE, 38000 Grenoble, France; 2 CNRS, IRIG-BGE, 38000 Grenoble, France; 3 Department of Cell and Developmental Biology, University of Pennsylvania, Philadelphia, PA 19104 USA; 4 Epigenetics Institute, University of Pennsylvania, Philadelphia, PA 19104 USA; 5 Univ. Grenoble Alpes, Inserm, CNRS, IAB, 38000 Grenoble, France; 6 Present Address: Institut de Génétique Moléculaire de Montpellier, 3400 Montpellier, France

## Abstract

**Background:**

Gametes are highly differentiated cells specialized to carry and protect the parental genetic information. During male germ cell maturation, histone proteins undergo distinct changes that result in a highly compacted chromatin organization. Technical difficulties exclude comprehensive analysis of precise histone mutations during mammalian spermatogenesis. The model organism *Saccharomyces cerevisiae* possesses a differentiation pathway termed sporulation which exhibits striking similarities to mammalian spermatogenesis. This study took advantage of this yeast pathway to first perform systematic mutational and proteomics screens on histones, revealing amino acid residues which are essential for the formation of spores.

**Methods:**

A systematic mutational screen has been performed on the histones H2A and H2B, generating ~ 250 mutants using two genetic backgrounds and assessing their ability to form spores. In addition, histones were purified at key stages of sporulation and post-translational modifications analyzed by mass spectrometry.

**Results:**

The mutation of 75 H2A H2B residues affected sporulation, many of which were localized to the nucleosome lateral surface. The use of different genetic backgrounds confirmed the importance of many of the residues, as 48% of yeast histone mutants exhibited impaired formation of spores in both genetic backgrounds. Extensive proteomic analysis identified 67 unique post-translational modifications during sporulation, 27 of which were previously unreported in yeast. Furthermore, 33 modifications are located on residues that were found to be essential for efficient sporulation in our genetic mutation screens. The quantitative analysis of these modifications revealed a massive deacetylation of all core histones during the pre-meiotic phase and a close interplay between H4 acetylation and methylation during yeast sporulation. Methylation of H2BK37 was also identified as a new histone marker of meiosis and the mouse paralog, H2BK34, was also enriched for methylation during meiosis in the testes, establishing conservation during mammalian spermatogenesis.

**Conclusion:**

Our results demonstrate that a combination of genetic and proteomic approaches applied to yeast sporulation can reveal new aspects of chromatin signaling pathways during mammalian spermatogenesis.

## Introduction

Gametes are highly specialized cells dedicated to the reproduction of their species by contributing the parental genetic information that generates a new individual. The organization of the germ cell genome is essential for the viability of the fertilized egg and the future development of an embryo. Indeed, a defect in the organization of chromatin, the combination of DNA and associated proteins, is a classic cause of male infertility [1–4]. During male gamete differentiation, germ cells undergo meiosis to produce four unique haploid cells. These cells subsequently undergo profound changes in cellular morphology and nuclear organization, ultimately resulting in the dramatic chromatin compaction and condensation in the mature gamete. Chromatin organization plays an important role during each of these steps and is regulated by a number of key mechanisms, including: non-coding regulatory RNAs (ncRNA), remodeling factors, histone variants and covalent post-translational modifications (PTMs) of histones [5–10]. Histone PTMs are an essential regulatory element of spermatogenic chromatin dynamics. For example, H2A.X phosphorylation on serine 139 (gamma H2A.X) is essential for meiosis and meiotic sex chromosome inactivation (MSCI) [11]. Acetylation of lysine (K)44 of histone H4 (H4K44ac) during meiosis promotes chromatin accessibility during homologous recombination [12]. In post-meiotic cells, histones H2A and H4 are hyperacetylated prior to their replacement by sperm-specific proteins in the final stages of maturation [13–15]. Numerous other histone PTMs have been identified in distinct stages of spermatogenesis and mature sperm [16, 17] and several are associated with distinct functions in male germ cells [15, 18].

The formation of functional mammalian gametes relies on the successful completion of several complex events. First, meiotic recombination mixes parental genetic information to form unique haploid gametes. Subsequent post-meiotic differentiation of gametes, especially in the paternal germline, is associated with a dramatic reorganization of chromatin [19]. This begins with a wave of hyperacetylation of histones, which are subsequently evicted and replaced sequentially by transition proteins followed by protamines, which are small, highly basic, sperm-specific proteins [20–25]. This unique, male gamete-specific mechanism of chromatin compaction allows for a dramatic change in cell size and morphology that culminates with formation of the mature spermatozoa.

Furthermore, this sequence of events is conserved in unicellular organisms [26]. Sporulation is induced in divergent yeast species, such as *Saccharomyces cerevisiae* and *Schizosaccharomyces pombe,* to initiate sexual differentiation and subsequent meiotic recombination and division. Following meiosis, the genetically unique haploid daughter cells undergo extreme differentiation to become spores. These changes include formation of a highly compacted nucleus and spore wall, which safeguards against extreme environmental conditions, such as heat shock or dehydration [27, 28]. Chromatin undergoes reorganization and compaction during the later stages of sporulation to facilitate the formation of a viable spore. These conserved cellular processes and changes to chromatin organization make yeast an ideal genetic model to study the complex physiological process of mammalian spermatogenesis.

The complexity of mammalian gametogenesis hinders utilization of systematic genetic and epigenetic screens. However, improvement of biochemical and proteomic technologies has allowed elucidation of several key aspects of chromatin dynamics during sperm differentiation. For example, liquid chromatography–tandem mass spectrometry (nanoLC–MS/MS) has revealed new histone variants which are important for sperm differentiation and discovered the diversity of acyl modifications of histones, not limited to acetylation but also including different lengths of the carbon chain [16, 29, 30]. However, the development of mouse genetic models remains lengthy and costly. For these reasons, utilization of the *S. cerevisiae* sporulation model, which exhibits numerous similarities with mammalian spermatogenesis, provides the amenability of combining powerful genetic and proteomic screens. Indeed, histone PTM analysis has benefited from the recent improvement of proteomic technologies leading to detection of novel sites of post-translational modifications [16] and of new covalently attached modifications, including new acyl modifications on Lys and O-GlcNAc on Ser/Thr [29]. Some of these histone PTMs are now associated with clear biological functions [15, 31, 32]; however, the function of most modified sites remains to be characterized.

We previously performed a systematic mutational screen on H3 and H4, revealing new modifications important for meiosis and spore differentiation [12, 33]. Here, we report a similar functional screen for H2A and H2B and find that many amino acids of these histones are essential for the formation of functional spores. We also utilized nanoLC–MS/MS for comprehensive analysis of histone PTMs during sporulation, identifying a total of 67 PTMs with 27 new sites of acetylation, methylation or phosphorylation in yeasts. In addition, 33 PTMs are located on residues which are critical for the formation of spores. The quantitative analysis of these modifications reveals a massive deacetylation of all core histones during the pre-meiotic phase and a close interplay between H4 acetylation and methylation during yeast sporulation. We further identify methylation of H2BK37 as meiotic-specific and essential for viable spores, and demonstrate its evolutionary conservation in meiosis during mouse spermatogenesis. Thus, the combination of genetic mutational screening with proteomic analysis is synergistic in revealing new and functionally important PTMs during gametogenesis.

## Results

### H2A H2B genetic screens reveal residues essential for sporulation

Histone mutant collections are highly valuable in the yeast *S. cerevisiae* [34–39]. Here, we utilized previously generated mutant collections to perform a systematic mutational screen on the histones H2A and H2B during sporulation. Specifically, we focused on analyzing the highly modified amino acid residues lysine, arginine, serine, threonine, proline and histidine. These residues were mutated individually to alanine. Strains from the SHIMA collection of the s288c genetic background [34] were diploidized and induced into sporulation. Conditions were optimized to ensure a reproducible and significant efficiency of the WT strain, with 5 days incubation under strong aeration using baffled flasks. Sporulation efficiency was assessed as the frequency of yeast cells able to form spores.

**Fig. 1:**
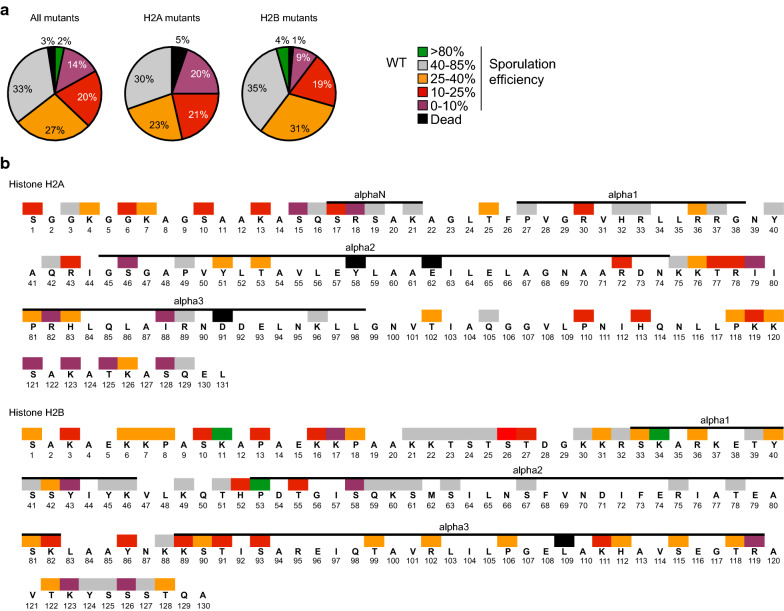
A systematic screen on the histones H2A H2B identifies residues essential for sporulation in the s228c genetic background. **a** Statistical analysis of the sporulation efficiency of the H2A H2B mutants. The severity of the sporulation defects is described using the following color code: a sporulation efficiency included within 0–10%, 10–25%, 25–40%, 40–80% and 80–100% is represented in purple, red, orange, grey and green, respectively. Lethal mutations are represented in black. This same color-coding is used throughout the figures of the manuscript for the s288c background. **b** Localization of the substitution mutants with affected sporulation efficiency. Histone fold is represented on the top of each histone based on the yeast nucleosome structure [81]

Out of 118 mutant strains, 75 were defective for the formation of spores (Table 1, Fig. 1a, Additional file 1, sporulation efficiency below 40%). The corresponding mutated residues are located on the N- and C-terminal tails and in the loops of the histone fold of H2A and H2B, which are the most accessible parts of the histones when incorporated into nucleosomes (Fig. 1b). Importantly, several mutations corroborate phenotypes observed in previous studies. Here we found that mutation of H2BK123 and subsequent loss of ubiquitination (ub) totally abolished the formation of spores (Fig. 1b) which is confirmed by previous reports that intact H2BK123 is critical for meiosis [40, 41]. In addition, the phosphorylation (ph) of H2AS121 by Bub1 in *S. cerevisiae* and *S. pombe* is important for chromosomal stability during meiosis [42]. Indeed, we found that the mutation of this amino acid severely affected the progression of sporulation (Fig. 1b).

### Histone local binary switches during sporulation

The possible interactions or exclusion of modifications on two neighboring residues has been described as histone “local binary switches” [43]. Several were identified in the genetic screen performed during sporulation. In the s288c genetic background, WT strains usually form at ~ 50% of spores 5 days after induction, thus allowing for residues to be revealed whose mutations are associated with a gain of function. This screen revealed that H2BK11 and H2BP53 are normally inhibitory to the formation of spores, as their mutation to an alanine increases the efficiency of these strains to sporulate (Fig. 1b, Additional file 1: Figure S1). Intriguingly, crosstalk between H2BS10 and neighboring amino acid residue H2BK11 has previously been described. H2BS10 is phosphorylated by the yeast Ste20 kinase during apoptosis and meiosis [44, 45]. H2BK11 acetylation is inhibitory for the action of Ste20 on H2BS10 and must be deacetylated to maximize Ste20-induced phosphorylation [46]. Our results are in accordance with these findings, as H2BS10 mutation disrupts the spore formation, while H2BK11 mutation prevents acetylation and likely promotes the formation of spores by facilitating Ste20 activity on H2BS10 (Additional file 1: Figure S1). Interestingly, we observed similar contrasting effects on sporulation with mutation of residues H2BS33 (decreased sporulation) and H2BK34 (increased sporulation, Fig. 1b and Additional file 1: Figure S1) as well as H2BH52 (decrease) and H2BP53 (increase, Fig. 1b, Additional file 1: Figure S1). These patterns suggest novel sites of histone binary switches, whose potential chromatin writers and readers remain to be characterized [43].

### Generation of a new H2A H2B mutant collection in the SK1 background

**Fig. 2:**
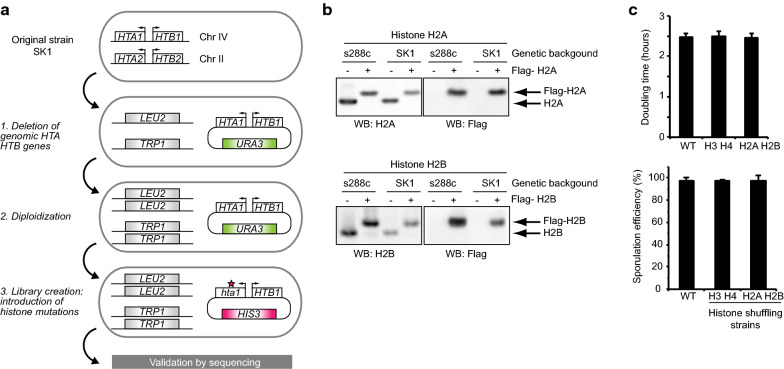
Creation of the histone H2A and H2B shuffle strain in the SK1 genetic background. **a** Creation strategy. Genomic copies encoding H2A (*HTA1* and *HTA2*) and H2B (*HTB1* and *HBT2*) have been deleted and the *HTA1* and *HTB1* genes are expressed from an autonomous plasmid. The original *URA3* plasmid can be replaced by a new *HIS3* plasmid with any desired mutation. **b** Validation of the H2A and H2B shuffle strain. Plasmids encoding Flag-tagged H2A or H2B have been introduced in the strain and these histones have been detected by western blot. They confirm that all genomic copies of H2A/H2B have been deleted and the only H2A/H2B proteins are expressed from the autonomous plasmid in both SK1 and s288c genetic backgrounds. **c** No growth or sporulation defects are detected in the H2A and H2B shuffle strain in the SK1 background. The genetic manipulation did not affect doubling times during vegetative growth (top) or sporulation efficiency (bottom, see Additional file 1: Figure S1 for more details) when comparing the H2A H2B shuffling strain to a WT or a previously constructed H3 H4 shuffle strain [33]

We next performed a complementary mutation screen in the SK1 genetic background. This genetic background is classically used for sporulation studies, as cells synchronously undergo differentiation and > 98% form spores (Additional file 1: Figure S2). Endogenously, two clusters of H2A and H2B genes (*HTA1*-*HTB1* and *HTA2*-*HTB2)*, each organized in tandem with a common promoter, encode for these proteins. We constructed a new collection of H2A H2B mutant strains in the SK1 genetic background by deleting both *HTA1*-*HTB1* and *HTA2*-*HTB2* gene clusters and expressing histones H2A and H2B from a rescue plasmid (Fig. 2a). A collection of plasmids mutated for lysine, arginine, serine, threonine, proline and histidine residues in H2A and H2B were obtained from the SHIMA collection [34] and transformed individually into the parental strain. The introduction of plasmids with Flag-tagged H2A or H2B confirmed that all genomic copies are deleted and that H2A and H2B are only expressed from the *HIS3* plasmid (Fig. 2b). Importantly, following diploidization, the SK1 parental strain exhibited no growth or sporulation defects (Fig. 2c, Additional file 1: Figure S2). A complete rationale of yeast genetic experiments is detailed in the “Material and Method” section.

### Construction of H2A H2B mutant collection in the SK1 genetic background

**Fig. 3:**
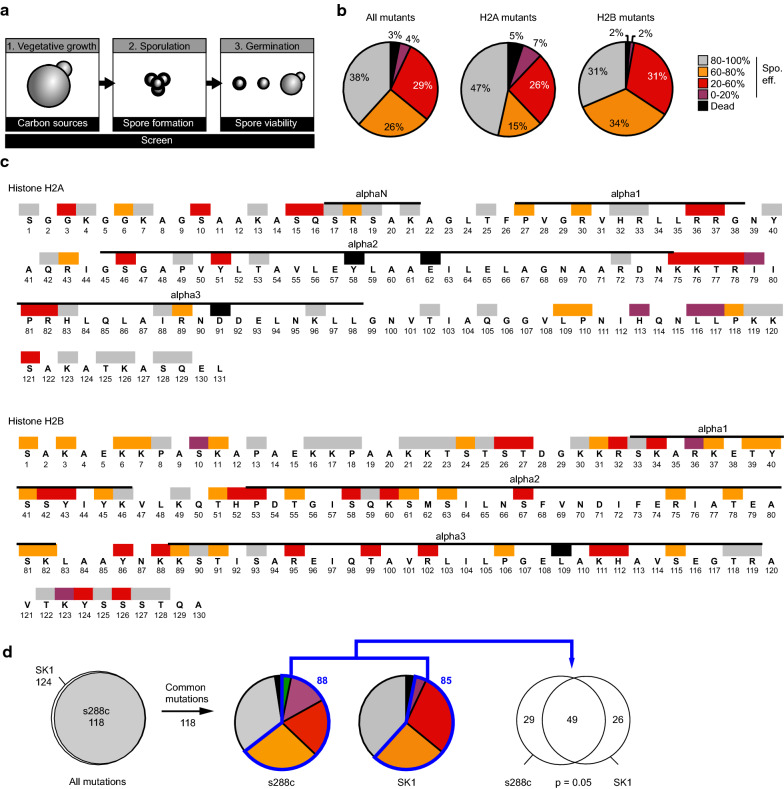
A systematic genetic screen identifies H2A and H2B residues essential for the formation of spores in the SK1 genetic background. **a** Schematic of the strategy used for the genetic screen which systematically mutated H2A and H2B residues to alanine. > 250 mutant strains with at least three independent isolates per mutation have been created and validated by sequencing. The growth of strains with non-lethal mutations was first assessed on acetate, a non-fermentable carbon source. Then, their ability to form spores and the viability and stress resistance of these spores has been characterized. **b** Statistical analysis of the sporulation efficiency of the H2A H2B mutants. The severity of the sporulation defects is described using the following color code: a sporulation efficiency included within 0–20%, 20–60%, 60–80%, and 80–100% is represented in purple, red, orange, and grey, respectively. Lethal mutations are represented in black. This same color-coding is used throughout the figures of the manuscript for the SK1 background in accordance with Ref. [33]. **c** Localization of the substitution mutants with affected sporulation efficiency. Histone fold is represented on the top of each histone based on the yeast nucleosome structure [81]. **d** Venn diagram representing the repartition of the residues important for sporulation in SK1 and s288c genetic backgrounds. 49 mutations are associated with sporulation defects in both s288c and SK1 background, out of a total of 88 and 85, respectively. This enrichment is statistically significant (hypergeometric test, *p* value of 0.05)

Isolates of each H2A/H2B SK1 genetic background mutant were sequence validated and subsequently analyzed. Similar to previously published data, individual mutations of H2A Y58A, E62A, D91A and H2B L109 were lethal and such strains excluded from our screen [34–36]. None of the other mutants displayed significant growth defects. Sporulation efficiency was assessed as the frequency of yeast cells able to form spores (Fig. 3a–c, Additional file 2). This screen revealed that 75 mutants out of 124, representing 60% of tested strains, are important for the formation of spores (< 80% efficiency), even if they do not have a phenotype during vegetative growth (Table 1).

A strong similarity was observed between genetic strains, as 65% of mutants with sporulation defects in the SK1 background (49 out of 75) are also defective in the s288c background (Fig. 3d, p value < 10^−5^ with hypergeometric testing). Similar to the s288c background, H2BK123 is essential for sporulation in the SK1 strain. Interestingly, the adjacent residue, H2BY124, is also important for sporulation efficiency (Fig. 3f), however it remains unclear whether this phenotype is related to H2BK123, as the mutation of this residue does not affect the ubiquitination of H2BK123 in vegetative cells [36]. Additionally, mutation of H2AS121 resulted in a profound sporulation defect (sporulation efficiency below 9%) in SK1 genetic background, indicating that it is essential for sporulation in both genetic backgrounds [42].

The systematic mutation of the residues of the N-terminal tail of H2A and H2B have previously revealed their importance for gene repression in *S. cerevisiae* [47, 48]. H2A S17 and R18 and the H2B region between amino acids 30 and 37, named H2B repression domain (HBR), are particularly important for this repression [47, 48]. In the s288c background, these residues were important for the formation of spores (Fig. 1). However, in the SK1 background, only mutations in the HBR region exhibited strong consequences on the formation of spores (Fig. 3, Sporulation efficiencies: H2BR32: 57%, K34: 35%, R35: 36%). The mutation of H2AS17 or R18 did not severely affect sporulation efficiency, suggesting strain-specific functions of these particular residues.

The mutation of H2BR102 and K111 resulted in severe sporulation defects in both s288c and SK1 backgrounds. These residues have been shown to be important for sub-telomeric silencing and suggested to participate in the recruitment of Sir proteins [49]. Altogether, H2BR102 and K111 appear to behave in a similar pathway as H3 loss of sporulation (LOS) mutants [33].

### The formation of dyads is increased in many H2A H2B mutants

**Fig. 4:**
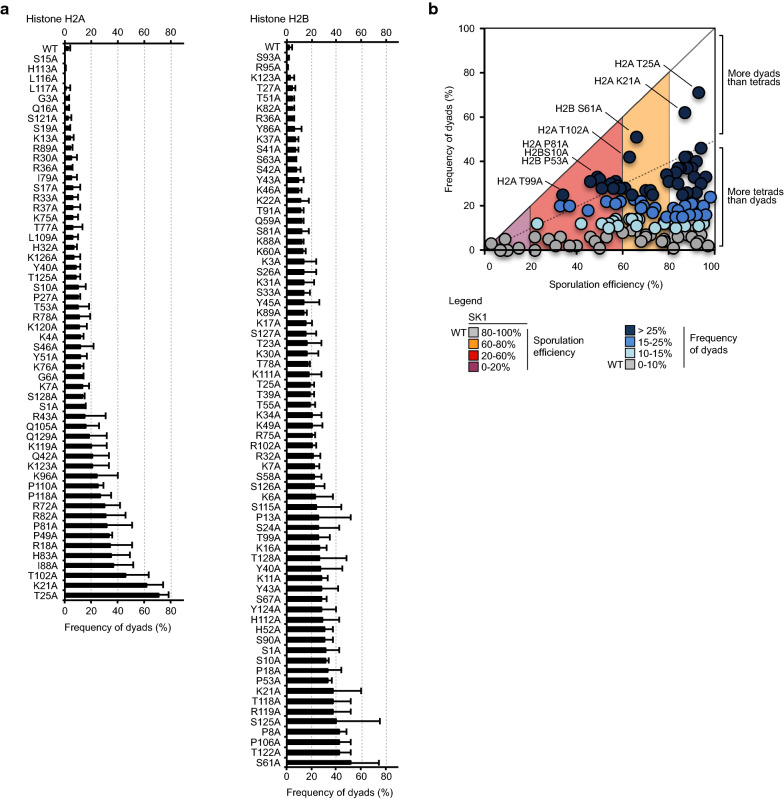
Increased formation of dyads in H2A or H2B mutant strains in the SK1 genetic background. **a** Frequencies of dyads observed for each mutant. Value for the WT strain is presented as the first entry. **b** Representation of the frequency of dyad and spores (dyads and tetrads). Residues with the most defects are highlighted

Sporulation ends with the formation of four spores grouped in an ascus. Surprisingly, the frequency of ascus with only two spores (named dyads) was above 15% for > 50% of the SK1 mutant strains (Fig. 4). An increased frequency of dyads is observed on both H2A and H2B mutants, yet with a higher proportion affecting H2B (80%, 56 out of 70) compared to H2A (58%, 32 out of 55). This phenotype had not been observed when mutating H3 or H4 residues [33]. This phenotype is observed after the induction of sporulation and is independent of vegetative growth, where no defects were observed in rich media (YPD or YPA). The formation of dyads reflects defects in meiotic divisions and altered spindle pole bodies [27, 28]. This increased frequency of dyads seemed independent from the abundance of tetrads (Fig. 4). Particularly, the mutation of H2AT25, K21, and H2BS61, P106 or T122 increased significantly the frequency of dyads upon sporulation induction.

### Importance of the DNA–nucleosome interface for sporulation completion

**Fig. 5:**
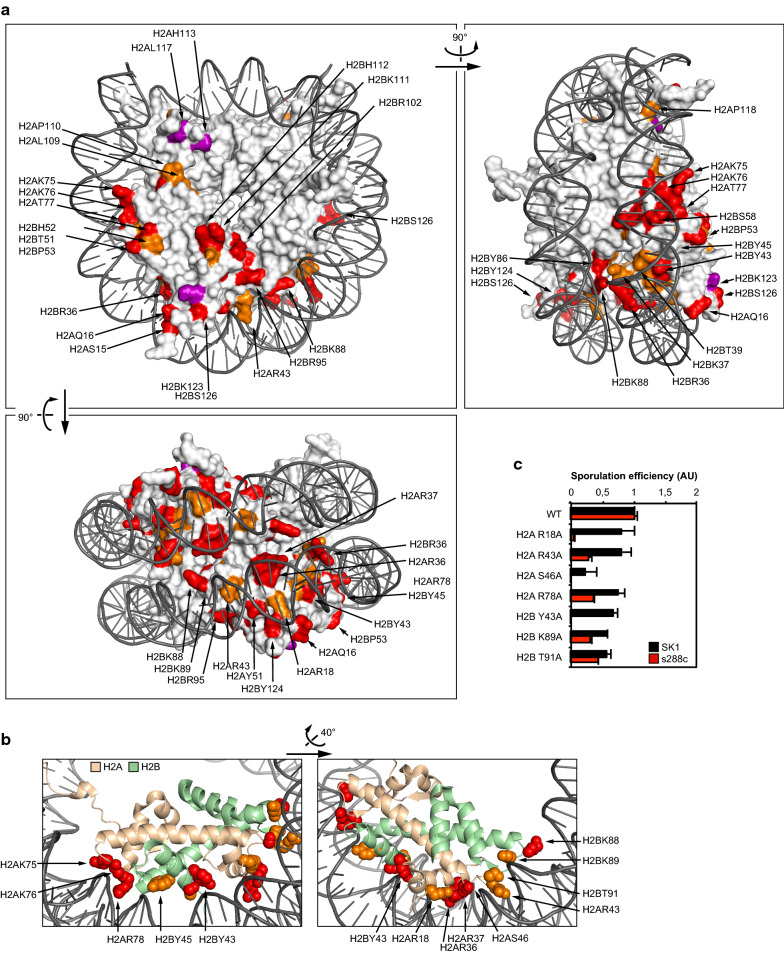
Representation of the nucleosome highlighting the residues essential for the completion of sporulation. **a** Residues which are important for sporulation are highlighted on the nucleosomal structure. Color scheme of the legend identical to Fig. 3. Structural information downloaded from entry 1ID3 [81] of the PDB database [105]. **b** Visualization of H2A H2B residues important for sporulation localized at the nucleosome–DNA interface. **c** Sporulation efficiency of a selection of residues presented in **b**. Sporulation efficiencies are expressed in arbitrary units, with WT normalized to 1 in SK1 and s288c backgrounds

In addition to the many residues important for sporulation on the N- and C-terminal tails and globular domain loops, numerous residues necessary for sporulation are located on the lateral surface of the nucleosome or near the DNA interacting region (Fig. 5). Some residues directly mediate an interaction with DNA and are particularly important for sporulation (H2AR18, R36, R37, R43, S46, K75, K76, R78 and H2BY43, Y45, K88, K89, T91), as their mutation is associated with a strong phenotype in both SK1 and s288c genetic backgrounds (Fig. 5b, c). It is highly intriguing that these residues interacting with DNA are essential for sporulation, yet are dispensable for vegetative growth. We hypothesize that these residues, or their modifications, are involved in meiosis related pathways and more specifically mechanisms associated with sister chromatid recombination.

### Stress resistance of mutated spores

Nearly 40% of the mutants of the SK1 genetic background tested exhibited no sporulation defects under normal conditions. However, as environmental survival is key element of spore viability, we next tested their resistance to heat shock (55 °C for 40 min) and ether vapors. A control strain with mutated *SMK1* kinase was used as a positive control for stress resistance, as the deletion of this gene is responsible for a defective assembly of the spore wall and sensitivity of this strain to heat shock and ether treatment (Additional file 1: Figure S2 and [50]). The resistance to these stresses was tested for the spores obtained from all mutants without any significant sporulation defects (sporulation efficiency > 80%). Germination defects were only observed after a stress was applied on spores in strains mutated for H2A P118, Q129, H2B T118 and H2B S127. The contribution of these residues to the formation of mature spores remains to be characterized.

### NanoLC–MS/MS-based identification of histone modifications during sporulation

**Fig. 6:**
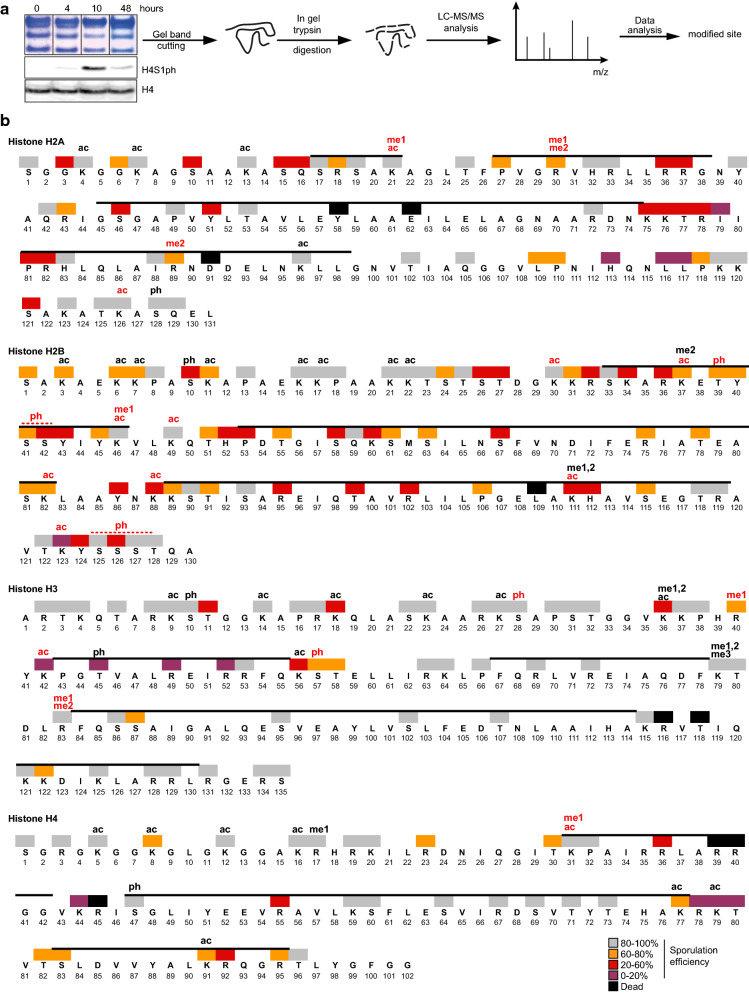
Mass spectrometry identification of post-translational modifications on core histones during sporulation. **a** Histones were purified during sporulation and analyzed by mass spectrometry. Technical details are presented in the Method section. **b** Modifications identified for each histone during sporulation. Ac, acetylation; me1/me2/me3, mono-, di- and trimethylation; ph, phosphorylation. Sites which had not been identified in yeast are highlighted in red, based on Table 3. Their MS/MS spectra are presented in Additional file 3. Phosphorylated H2B residues with ambiguous localization are indicated with a red bar. All histones were identified as N-terminally acetylated. Color coding of the residues is from SK1 background (Fig. 3)

Histone PTMs are important mediators of the chromatin signaling pathway due to their regulation of chromatin structure and recruitment of molecular machineries. For this reason, we investigated the PTMs present on residues critical for sporulation. We used an unbiased, nanoLC–MS/MS approach, to identify histone PTMs at key stages of sporulation: before induction (0 h), during meiosis (4 h), after meiotic divisions (10 h) and in mature spores (48 h). Histones were purified from defined stages and validated by western blot for the sporulation marker H4S1ph (Fig. 6a and Refs. [33, 51]). Purified histones were subsequently analyzed by nanoLC–MS/MS to identify histone PTMs. Given the diversity of currently described histone PTMs, this study focused specifically on the following modifications: phosphorylation of serine and threonine, acetylation of lysine, and methylation of lysine and arginine residues.

The proteomic pipeline implemented in this study identified 67 sites of acetylation, methylation (me1, me2 or me3) and phosphorylation of serine or threonine across all time points (Fig. 6b). Modified sites with previously well-defined functions were identified, including: H2AS128ph [52, 53]; H2BS10ph and H2BK11ac [44–46]; multiple H3 acetylations (residues K9, K14, K18, K23, K27, K56), methylations (residues K36, K79) and phosphorylations [54]; and classical acetylation sites on H4 (lysines K5, K8, K12 and K16). Of note, the function of two of these PTMs have extensively been explored in yeast, including H2BS10ph in meiotic progression [45] and the importance of H4 acetylation in the formation of functional spores [33].

We identified 27 modifications previously not identified in *S. cerevisiae* (highlighted in red Fig. 6b, Tables 2 and 3). MS/MS spectra of these modifications are presented in Additional file 3. These PTMs were defined as novel in *S. cerevisiae* if they were not identified in a recent comprehensive catalog of histone modifications [29] and present through a detailed search of the literature. Novel *S. cerevisiae* histone PTMs were further separated into three classes: (i) novel PTMs on amino acid residues previously described as modified in *S. cerevisiae*; (ii) PTMs not described in yeast but reported in other species; (iii) PTMs not reported in yeast or other species.

Interestingly, 51% (33 out of 67) of the PTMs are located on residues identified as functionally important for sporulation [either in this study or in our published H3 H4 screen in ref. 33]. For example, we identified here H3K56ac, a PTM previously described to be enriched during meiosis [33]. Interestingly, the neighboring residue H3S57 has been found to be phosphorylated [this study and 55]. However, the putative role of H3S57ph on H3K56ac remains to be further investigated [56]. This pattern of dually modified, neighboring amino acid residues follows the same pattern described above (Fig. 3, Additional file 1: Figure S2) and also refers to similar residues on H3 (H3S10/H3K9 and H3S28/H3K27) that may be prone to potential crosstalk between PTMs. We additionally identified H2AS10ph, a residue which is important for sporulation. In addition, two S/T patches of H2B were identified to be phosphorylated but the precise localization of the phosphorylated residues could not be mapped (H2BS41–42 and H2BS125–T128). H2B residues S41, S42 and S126 are essential for the formation of spores (Figs. 1, 2). However, whether sporulation function is dependent specifically on the phosphorylation of these residues remains to be confirmed.

Several acetylation and methylation sites were identified on residues important for sporulation, such as H2AR30me, H2AR89me, H2BK7ac, H2BK37ac, H2BK49ac, H2BK111ac, H3R40me and H3K42ac [33]. Although several of these residues had previously been shown to be modified in mammals or with other modifications (Table 3), all of these are newly identified PTMs in *S. cerevisiae* [29 and other publications].

### Histone modification in the globular domain

The mutation of many residues of the globular domain has a strong effect on sporulation efficiency. This could be due to the neutralization of the negative charge of a lysine or arginine when mutated to an alanine. However, several of these residues are modified during sporulation (Additional file 4) and are present in various parts of the globular domain, such as the DNA exit and entry, the dyad, or the lateral surface of the nucleosome. The modification of several of these, such as H3T45ph, H4K44ac, H4K79ac or H3K56ac, has already been shown to be important for chromatin organization [12, 57, 58], while the functional role of others remains to be explored.

### Core histones are massively deacetylated after sporulation induction

**Fig. 7:**
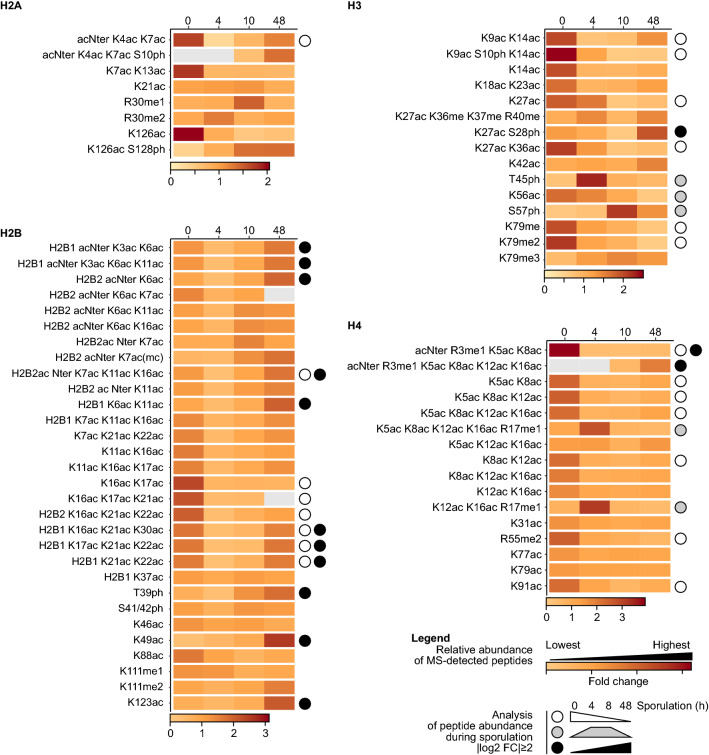
Quantification of the histone modifications identified by proteomics. The abundance of modified peptides was quantified and normalized to the signals of reference non-modified peptides for each histone, namely HLQLAIR and AGLTFPVGR for H2A, KETYSSYIYK and ETYSSYIYK for H2B, STELLIR for H3 and ISGLIYEETR and DNIQGITKPAIR for H4. The time after induction of sporulation is indicated in hours (4 h, meiosis; 10 h, post meiosis; 48 h, mature spores). The intensity of the brown color indicates the relative abundance during the progression of sporulation. Significant changes of abundance over the course of sporulation are highlighted with circles (| log2 of fold change | ≥ 2). Data are available in Additional file 5. acNter, N-terminal acetylation

We next quantified the abundance of modified peptides to assess changes in specific histone PTMs at distinct stages of sporulation (Fig. 7, Additional file 5). Histone PTMs exhibit dynamic modifications during sporulation, as 50% of identified PTMs undergo significant changes in abundance during spore differentiation (35 out of 70 quantified peptides with | log2(foldchange) | ≥ 2, Additional file 1: Figure S3). Three categories can be distinguished, with modifications most abundant (1) before induction, (2) over the course of meiosis and/or post meiosis and (3) enriched in mature spores [represented by (1) white, (2) grey and (3) black dots in Fig. 7]. First, a large proportion of acetylated peptides undergo a strong deacetylation prior to meiosis, between 0 and 4 h after sporulation induction. This deacetylation is significantly detected on H2A K4, K7, K13, K126, H2B K16, K17, H3 K9, K14, K27, and H4 at K5, K8, K12 K16 and H4 K91. Of note, this deacetylation was not previously detected by western blot analysis [33], likely due to multiple acetylated residues on the deacetylated peptides which cannot be detected by western blot. Interestingly, many of these sites have been described to be actively acetylated during gene activation, notably on the N terminus of H2A, H2B, H3 and H4, thus suggesting temporal regulation of transcription during sporulation. Another possibility that remains unexplored is pre-meiotic metabolic changes that may alter histone PTM signatures. However, the ultimate function of this wave of deacetylation remains to be elucidated.

### Meiotic-specific histone modifications

Several amino acid residues are specifically modified during meiosis. The methylation status of H2A R30 is particularly interesting, with an increase of H2A R30me2 during meiosis, and an increase of H2A R30me1 after meiotic divisions (Fig. 7, 10 h time point). This modification has been detected in human H2A R29, where this residue is methylated by PRMT6 [59]. The yeast arginine methyl transferase orthologue is HMT1 and could be involved in the methylation of H2A R30me; however its functional role remains undescribed [60].

H3 T45ph significantly increases during meiosis and could be related to pre-meiotic DNA replication. This modification is independent of H3K56ac [54], which is also increasing during meiosis (Fig. 7 and Ref. [33]). Remarkably, the increase of H3K56ac during meiosis (4 h) is followed by a phosphorylation of H3S57ph, detected in the post-meiotic phase. However, a potential molecular crosstalk between those two modifications is not yet described.

We further identified methylation of H4R17, which, while previously reported as methylated in human [61], has not been previously described in yeast. This modification is found enriched during meiosis when combined with acetylation of the H4 N-terminal tail.

### Histone modifications enriched in the chromatin of spores

The dynamics of modifications on the H4 tail appear particularly complex. As described above, H4 K5, K8, K12 and K16 are massively deacetylated during the pre-meiotic phase. Our previous work described a hyperacetylation of H4 in mature spores, which was detected by western blot [33]. This is confirmed by this proteomics-based quantification. However, it seems to be restricted to co-acetylation events on the same H4 tail, which is also associated with H4R3 methylation (Fig. 7). Extensive proteomics analyses on human H4 indicated that H4R3me is often associated with H4ac [62, 63]. Altogether, this quantitative analysis reveals a close interplay between H4 acetylation and methylation during yeast sporulation.

Several other amino acid residues exhibit increased acetylation in mature spores, notably on the N-terminal tail of H2B (Fig. 7). H2BK123ac undergoes one of the strongest inductions of acetylation during the post-meiotic (10 h) to spore (48 h) transition.

### H2BK37 methylation is a novel yeast meiotic modification

**Fig. 8:**
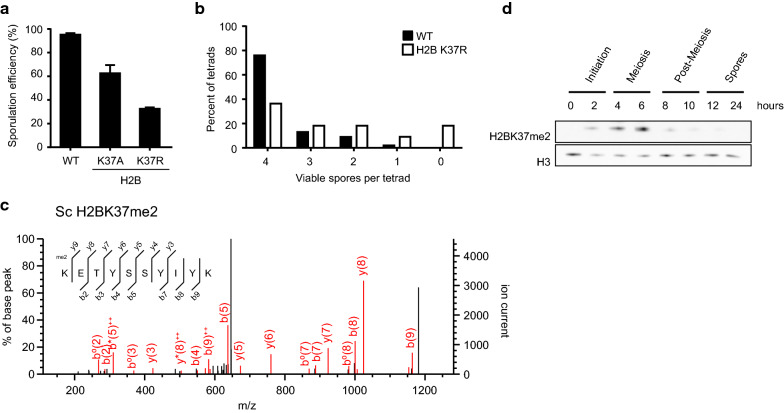
H2BK37 is methylated during yeast meiosis. **a** Sporulation efficiency in H2BK37A and H2BK37R mutants. Both of them significantly affect the formation of spores. **b** Spores viability upon H2BK37R mutation. **c** MS/MS fragmentation spectrum identifying H2BK37me2 in yeast histone purified during meiosis. *Sc, Saccharomyces cerevisiae*. **d** Analysis of the abundance of H2BK37me2 during sporulation

Based on the findings of our genetic and proteomic approaches, we further analyzed the specific residue H2BK37 which exhibited a strong sporulation defect upon mutation in our initial screen (Figs. 1, 3). To further investigate the function of this modified residue, we created a lysine (K) to arginine (R) mutant and analyzed sporulation efficiency. The mutation of K to R preserves the basic property of the original residue, while inhibiting its enzymatic modification. H2BK37R mutation resulted in a strong defect to sporulation efficiency, even greater than observed in the initial H2BK37A mutant (Fig. 8a). The viability of the H2BK37R mutated spores was also decreased (Fig. 8b) suggesting a meiotic defect associated with this residue.

NanoLC–MS/MS analysis detected H2BK37me2 in yeast during meiosis (4 h post sporulation induction, Fig. 8c). An antibody had previously been developed against H2BK37me2 [64]. To further investigate the timing of H2BK37 methylation during yeast meiosis, we analyzed protein lysates from a sporulation time course by western blot. H2BK37me2 was first observed 2 h post sporulation initiation and peaked during meiosis (6 h) before rapidly diminishing by 8 h in the post-meiotic cell (Fig. 8d). This pattern of histone methylation supports a novel and specific role during meiosis.

### H2BK34 methylation is a meiosis-specific PTM in mouse testes

**Fig. 9:**
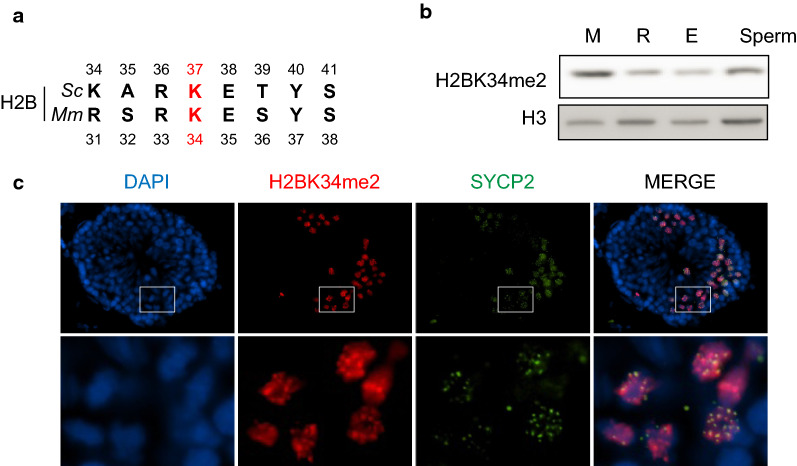
Mouse H2BK34me2 is detected in meiosis during spermatogenesis. **a** Alignment of H2B sequences in yeast and human around the yeast H2BK37 residue. *Sc*, *Saccharomyces cerevisiae. Mm, Mus musculus*. **b** H2BK34me2 levels during spermatogenesis. Cells were purified at different stages and analyzed by western blot. M, meiosis (spermatocytes); R, round spermatids; E, elongating spermatids. C. H2BK34me2 detection in mouse seminiferous tubules analyzed by immunofluorescence. H2BK34me2 were co-detected with Sycp2, a classic marker of meiosis. Insets are magnified in the bottom images

H2BK37 is a highly conserved amino acid residue and is present in mouse and human H2B at the position K34 (mH2BK34me2, Fig. 9a). To determine if mH2BK34me2 is also present in mammalian testes, we next analyzed protein lysates from distinct stages of mouse spermatogenesis for mH2BK34me2 abundance. Wild-type SV129 adult testes were fractionated using the STA-PUT separation to collect cells at distinct stages of spermatogenesis [65]. mH2BK34me2 appeared enriched during meiosis compared to post-meiotic differentiated round (R) and elongating/condensing (E) spermatids (Fig. 9b). We further performed immunofluorescence on testis cryosections and observed a similar pattern of mH2BK34me2 enrichment in meiotic spermatocytes (Fig. 9c). The co-detection of SYCP2 confirmed the increased abundance of mH2BK34me2 during meiosis, thus suggesting a potential conserved function for this novel PTM during mammalian spermatogenesis.

## Discussion

In this study, we utilized a systematic genetic screen with mutations for all modifiable residues of histones H2A and H2B in the yeast *S. cerevisiae* to explore if they are necessary for the formation of spores. The mutation of 75 amino acid residues affected sporulation, many of which were localized to the nucleosome lateral surface (Table 1). The use of different backgrounds confirmed the importance of many of the residues, as 48% of yeast histone mutants exhibit impaired formation of spores in both genetic backgrounds (70% of the mutants defective in the SK1 background were also defective in s288c). We next performed a complementary, unbiased, analysis of histone PTMs present during sporulation. Extensive proteomic analysis identified 67 unique PTMs during sporulation, 27 of which were previously unreported in yeast. Furthermore, 33 PTMs are located on residues that were found to be essential for efficient sporulation in our genetic mutation screens. This strongly suggests that residues essential for the formation of spores do not only play a structural role, but are also important for chromatin signaling pathways. We further identify a striking deacetylation of all core histones during the pre-meiotic phase of sporulation. This study also reveals that H2BK37, a residue identified by genetic screen as important for sporulation in yeast, is dimethylated during meiosis, and necessary for spore viability. Furthermore, this modification is conserved in mammals at mH2BK34 and enriched during meiosis in mouse testes.

**Table 1:** Histone mutations tested during sporulation

	Number of mutations	
	Tested^a^	Sporulation defects^b^
This study (s288c)		
H2A	52	36 (69%)
H2B	66	42 (64%)
This study (SK1)		
H2A	55	28 (51%)
H2B	69	47 (68%)
Govin et al. 2010 (SK1)		
H3	54	13 (24%)
H4	34	15 (44%)
Total (SK1)	212	103 (48%)

^a^Excluding lethal mutations

^b^With sporulation efficiency below 40% and 80% for s288c and SK1 genetic backgrounds, respectively. Expressed as a percentage of the total of mutant tested by category (rows)

### H2B binary cassettes during sporulation

Two collections of H2A and H2B mutants were screened for sporulation defects. They differ by genetic background, with each revealing interesting aspects of the importance of these histone residues for sporulation. First, existing H2A H2B collections in the s288c background were diploidized and induced for sporulation. 64% (75 out of 118) of mutants were defective in the formation of spores. Interestingly, the use of this genetic background revealed the importance of two binary cassettes during sporulation [43]. First, H2BS10ph/K11ac, which has previously been described as mutually exclusive PTMs important for apoptosis [44, 45], were found to affect sporulation. Here, the mutation of H2BK11 increases sporulation efficiency, suggesting that the mutation of this residue could favor the deposition of H2BS10ph and promote the formation of spores. Another modification cassette is present at positions H2BS33 and K34, however no modification was identified previously or by our proteomic analysis at these residues. Further study is necessary to identify the molecular process by which the mutation of H2BK34 increases sporulation efficiency in the s288c background. Finally, another binary cassette containing H2BH52 and P53 may similarly be involved in histone PTM crosstalk, as the mutation of H2BP53 increases sporulation efficiency. However, the molecular mechanisms involved in this cassette remain to be discovered, specifically how the mutation of a proline residue could affect phosphorylation of H2BH52. In the SK1 background, where 98% of spores are observed 24 h after induction, the mutation of residues involved in binary cassettes affected sporulation, even if the same mutation increased sporulation efficiency in s288c background. This suggests that those residues, H2BK12, K34 and P53, could functionally repress sporulation induction but also be required later in the differentiation of mature spores.

### H2BR102, K111 and H3 loss of sporulation (LOS) mutants

The histone amino acid residues H2BR102 and K111 are important mediators of DNA damage, telomeric silencing, and Sir4 binding [49]. Both of these residues have been demonstrated as important mediators of sporulation and their role could be similar to the H3 LOS mutants [33]. These H2B residues and loss of rDNA silencing (LRS) mutants on H3 and H4 have been shown to create a Sir3 binding interface important for its recruitment through its BAH domain [66]. However, it is questionable whether the sporulation phenotype of H2BR102 and K111 mutants are caused by a disruption of this interface, as not all LRS mutants display a sporulation phenotype [33]. The H3 LOS mutants are a sub-selection of the LRS mutants with a sporulation-specific phenotype. It has been suggested that the formation of the telomeric cluster during meiosis, which is critical for the formation of chromosomal pairs and meiotic division, could involve a Sir3-independent pathway mediated by H3 LOS and H2BR102 and K111 residues. Interestingly, we detected methylation and acetylation marks on H2BK111 during sporulation (Fig. 6). This residue was previously found to be methylated in yeast [67] and human [68]. Whether this specific PTM is responsible for the defects observed upon mutation of this residue in our genetic screen remains to be explored.

### Dyad formation and carbon metabolism

In contrast to mammals, mitosis and meiosis in yeast takes place without disruption of the nuclear envelope. During meiosis I and then II, a single pole body (SPB) duplicates to form two nucleation centers for spindle microtubules, which will drive the separation of the chromosomal content. Defects in the SPB at meiosis I or II will generate different forms of dyads (diploid but also non-sister dyads, resulting from a defects of SPB meiosis II outer plates) [28]. Such defects can be induced by changes in acetate-mediated metabolism or gene mutation (*HFD1, ADY1, SPO21, SPO74, MPC54, SPC4,* reviewed in [28]). The formation of dyads was not increased upon mutation of H3 or H4 [33], but many H2A and H2B mutants in the SK1 background which exhibited defective formation of tetrads were instead prone to forming dyads. The mutation of H2A or H2B could deregulate the expression of genes which are essential for the formation of the SPB, its outer plaque, and proper separation of genetic material.

In addition, dyad formation is regulated by the availability of carbon sources during sporulation, and the depletion of a non-fermentable carbon source such as acetate triggers dyad formation [28, 69]. In this study, no growth defects using fermentable (glucose) or non-fermentable (acetate) carbon sources were identified for any of the residues whose mutation increases the frequency of dyads. However, H2A or H2B mutations could deregulate the expression of genes important for acetate metabolism during meiosis, which would subsequently impair the proper formation of tetrads. Regardless, the molecular processes by which these amino acid residues regulate the proper function of spindle pole bodies and the formation of the spore wall remain to be elucidated.

### Histone modifications during sporulation

Together with our previous study, we have now utilized genetic screens to identify H3, H4, H2A and H2B amino acid residues important for the differentiation of spores [33 and this study]. Chromatin signaling pathways classically involve histone PTMs which are deposited, removed, and read by dedicated complexes. For this reason, we explored whether the residues which are critical for the formation of spores are modified during sporulation. The analysis by mass spectrometry of purified histones at key sporulation stages revealed a total of 67 unique PTMs, 27 of which were previously unreferenced in yeast and 8 being identified for the first time in any cell type (Tables 2 and 3). This data contributes to the growing catalog of histone PTMs that has continually increased over the last several years.

**Table 2:** Histone post-translational modifications identified during sporulation

	Number of modifications detected	Modifications not described in yeast^a^	Modifications present on residues important for sporulation^b^
H2A	12	7	3
H2B	24	12	17
H3	20	6	8
H4	11	2	5
Total	67	27	33

^a^Mainly based on references [29, 67]

^b^With sporulation efficiency below 80% in the SK1 genetic background

**Table 3:** List of new histone modifications identified in *S. cerevisiae*

Identified in this study and not documented elsewhere			
Residue	Spo. eff.	Comment	References
H2A S10ph			
H2B K30ac			
H2B S41-S42 ph^a^			
H2B K49ac		Acylated in human (H2B K46)	[31, 91, 92]
H2B K82ac		Methylated in human (H2B K79me1)	[16]
H2B S125-T128 ph^a^			
H3 R40me1			

Identified in this study, not described in *S. cerevisiae* but identified in other species			
Yeast residue	Spo. eff.	Mammalian residue	References
H2A R30me1, me2		H2A R29me1, me2	[93]
H2A R89me2		H2A R88me1	[16]
H2B T39ph		H2B T36ph	[94, 95]
H2B K88ac		H2B K85ac	[16, 31, 96, 97]
H2B K111ac		H2B K108ac	[61, 91, 98, 99]
H3 S28ph		H3 S28ph	[100]
H3 S57ph		H3 S57ph	[55]
H3 R83me1, me2		H3 R83me1, me2	[61]
H4 R17me1		H4 R17me1, me2, me3	[61]
H4 K31ac		H4 K31ac	[101]
H4 K31me1		H4 K31me1	[101]

Identified in this study, not described in *S. cerevisiae* but other modification of the same residue identified in yeast			
Residue	Spo. eff.	Known modifications	References
H2A K21ac		Succinylation	[92]
H2A K21me1		Succinylation	[92]
H2A K126ac		Sumoylation	[102]
H2B K37ac		Methylation (me1)	[67]
H2B K46ac		Formylation, succinylation, 2-hydroxyisobutyrylation	[92]
H2B K46me1		“	[92]
H2B K111ac		Methylation	[67]
H2B K123ac		Ubiquitination	[103]
H3 K42ac		Methylation	[104]

The column “Spo. eff.” represents sporulation efficiencies in the SK1 background (color-coded as described in Fig. 3f for H2A and H2B and in Ref. [33] for H3 and H4)

^a^The phosphorylation site could not be precisely mapped

We observe several distinct patterns of histone PTM dynamics during sporulation that coincide with critical molecular and biological functions, including initiation of sporulation, meiosis, and post-meiotic spore maturation. For example, during meiosis we observe dynamic changing of the histone PTM H3T45ph. This modification has previously been shown to be involved in DNA nicking during apoptosis, suggesting it may play a role in DNA double stranded break formation during meiotic recombination [57]. The rapid enrichment of this histone PTM during meiosis, followed by subsequent reduction during post-meiotic spore maturation may be critical for limiting DNA damage outside of specific recombination events.

### Dynamics of histone acetylation during sporulation

The functional roles of many of the PTMs identified during sporulation have been previously documented in other cell types and their quantification during sporulation provides a new perspective on their dynamics. Indeed, a recent study predicted the impact of acetylation on amino acid residues involved with nucleosomal DNA accessibility [70]. Many of the residues whose modifications are predicted to have a significant impact on DNA accessibility were also important for sporulation in our genetic substitution screen (Additional file 4).

This study reveals a massive deacetylation of all core histones during the pre-meiotic phase. It largely affects patches of multi-acetylated amino acid residues on N-terminal tails. Metabolic changes are well described to be interconnected with histone modifications and could explain this massive deacetylation. Sporulation is induced by nitrogen deprivation, which occurs upon transferring yeasts from a rich media with a non-fermentable carbon source (acetate, medium YPA) to carbon acetate only (K acetate 2% with essential amino acids). However, the putative interplay between metabolism and histone acetylation remains to be explored during sporulation. It is particularly interesting to note that metabolic signaling and transcription has recently been linked through histone crotonylation and Taf14, which represses pro-growth genes upon activation of fatty acid ß-oxidation genes [71].

We further demonstrate that H2A and H2B N- and C-terminal tails can bear many modifications (this study and ref. [29, 67]). They are dramatically deacetylated after the induction of sporulation before becoming re-acetylated during the final maturation stages of spores. In addition, previous reports suggest that the acetylation of the N-terminal tail of H4 is important for nuclear compaction in spores as mediator by the BET protein Bdf1 [33, 72]. Here, the MS-based quantification analysis reveals a close interplay between H4 acetylation and methylation during yeast sporulation. H4 R3 methylation appears to be required for H4 hyperacetylation. A co-occurrence of H4R3me and H4ac has also been described with systematic MS analysis of H4 modifications in human [62, 63]. However, the molecular mechanisms that underlie the modification of these amino acid residues remains to be discovered. One potential explanation yet to be explored is whether H4R3me can affect the recruitment of H4 ac binders, notably the BET proteins to acetylated lysine residues [72].

H3K56ac has long been known to be involved in nucleosome assembly during replication [58, 73, 74]. Indeed, this PTM is enriched prior to meiosis and mutation of this residue affects the production of spores [33]. In this study, we identified the adjacent residue, H3S57, to be phosphorylated during sporulation. H3S57ph has been experimentally confirmed and its abundance quantified in mouse brain [55], with a potential relationship to Alzheimer’s disease. The effect of mutations on these residues has also been previously studied in yeast, where H3S57 mutation interplays with H3K56 mutation during transcription elongation and recovery from S-phase stress [56]. Here, H3K56ac and H3S57ph were sequentially detected as enriched at 4 h (meiotic) and 8 h (post-meiotic) after sporulation induction, respectively. Additional studies will likely provide further details into the molecular mechanisms of histone PTM crosstalk between H3K56ac and H3S57ph.

### Evolutionary conservation of H2BK37 methylation during meiosis

Systematic mutations of tail amino acid residues revealed their importance for gene repression in yeast, especially of the HBR domain [H2BK30 to K37 and Ref. 47, 48]. This study revealed the influence of this domain during sporulation. The HBR domain has recently been shown to be important for histone deposition by the FACT complex and its mutation results in a loss of histone occupancy [75]. As mentioned above, correct histone dosage is important for sporulation and a lower amount of H2A and H2B is deleterious for sporulation [76–80]. Therefore, it is tempting to speculate that the sporulation phenotype of HBR domain mutations may be related to a loss in histone occupancy.

Furthermore, multiple residues residing in the HBR domain are potentially modifiable, however only the H2BK37 residue has been identified as methylated. This modification had previously been identified, however no functional role was reported during vegetative growth [64]. Here, the analysis of the abundance of this modification during sporulation showed increased H2BK37me2 during meiosis in yeast. This residue is located at the extremity of the HBR domain, and it is not yet known if mutation of the H2BK37 residue alone recapitulates the phenotype of a full deletion of the HBR domain (residues 30 to 37). Especially, regarding its ability to mediate the interaction of H2A/H2B with FACT to support proper histone loading onto chromatin. Indeed, full deletion of the HBR domain may have a much larger impact on the nucleosomal structure than the individual mutation of H2BK37, as it removes the five first amino acids of the first alpha helix of H2B histone fold [81].

Furthermore, this meiosis-specific marker identified during yeast sporulation has been evolutionarily conserved and is annotated as the residue H2BK34 in human and mouse. In this study we demonstrate similar dynamic changes to H2BK34me2 during mammalian spermatogenesis, with a robust enrichment of this PTM during meiosis in the mouse testis.

### Histone modifications and chromatin structure in gametes

The processes of gamete differentiation and maturation in yeast sporulation and mammalian spermatogenesis share remarkable similarities during meiosis and post-meiotic chromatin compaction. During mammalian spermatogenesis, a dramatic increase in histone acetylation occurs following meiosis, which ultimately results in chromatin compaction. In this study, we report a similar molecular pathway in yeast spores, with dynamic changes in hyperacetylation of histone lysine residues, particularly histone H2B and H4, after meiosis (Fig. 6) [33], thus strongly suggesting conservation of modified amino acid residues and function.

Interestingly, protamines, an essential component of mature mammalian sperm, are not present in some species such as yeast and zebrafish [82, 83]. However, it has been hypothesized that protamines are either evolutionary derived [84, 85] or biochemically cleaved from linker histones [86], thus further supporting the evolutionary conservation of chromatin dynamics during gametogenesis of multiple species. Indeed, previous studies have found specific histone PTMs, including H4S1ph [51], H3T11ph, H3S10ph, and H3K56ac [33] conserved between yeast sporulation and mouse spermatogenesis. Our study utilizes these similarities between gametogenesis pathways to discover candidate PTMs that may play critical roles during spermatogenesis that may otherwise remain unknown, due to the difficulties in generating genetic mutations of histones in higher organisms. While further validation is needed of the PTMs defined in this manuscript, this screen provides an important resource to identify these potential regulators of mammalian spermatogenesis.

## Conclusion

During the last several years, advances in proteomic technologies, particularly mass spectrometry, have allowed for the extensive analysis of histones, progressively reaching a plateau of saturation in the discovery of histone PTMs. In this study however, the combination of proteomic analysis and genetic screens facilitated the identification of amino acid residues whose modifications may be functionally relevant for the formation of spores. Importantly, given the mechanistic similarities between yeast sporulation and mammalian spermatogenesis, these findings can be used to identify potential epigenetic regulators of gamete formation in mice and humans. Indeed, we have identified and validated the function of H2BK37me2 in yeast sporulation and further demonstrated its conservation in the mouse testis. The next challenge resides in the exploration of potential roles in chromatin dynamics during gametogenesis.

## Materials and methods

### Antibodies

Antibodies were obtained from the following providers: Flag (Sigma F7425), H2A (Active Motif, reference 39235), H2B (Active Motif, reference 39237), H3 (Abcam Ab1791) and H2BK34me2 (kind gift of Brian Strahl, Ref. [64]).

### Yeast manipulation

Lists of strains and plasmids are presented in Additional file 1, Tables S1 and S2.

*s288c genetic background.* Mutant strains were kindly provided by Ali Shilatifard [34]. In these strains, the mutations of H2A or H2B had been introduced on a *HIS3* containing plasmid. They were haploid and the transformation of an *HO*-*URA3* plasmid generated diploid strains. Individual isolates were collected and the *HO*-*URA3* plasmid evicted using 5-fluoroorotic acid (FOA). Ploidy was confirmed by PCR [87].

*SK1 genetic background* H2A and H2B encoding loci were deleted in the SK1 background using standard methods [88]. In short, the fragment containing the *LEU2* gene and the flanking regions of the *HTA1*-*HTB1* locus was amplified by PCR from the H2A/H2B collection of the FY406 strain [34] and transformed into an SK1 diploid strain. The *HTA2*-*HTB2* locus was deleted similarly using the *TRP1* gene obtained from the FY406 strain. Next, the pSAB6 plasmid, which contains the *HTA1*-*HTB1* locus, was chosen to ectopically express H2A and H2B proteins. Indeed, reduced levels of H2A and H2B induce pleiotropic phenotypes on general transcription, chromosome segregation, heat shock response and cell cycle progression [76–79]. Furthermore, the *HTA1*-*HTB1* locus is more expressed than the *HTA2*-*HTB2* locus [76] and it is critical for the expression of sporulation master-regulators and the formation of spores [80]. Finally, the promoter of the *HTA1*-*HTB1* locus contains a regulatory sequence which modulates the expression of these two genes depending on the levels of H2A and H2B histone proteins, therefore autogenously regulating a normal level of histone [79]. For all these reasons, histones H2A and H2B were expressed from the *HTA1*-*HTB1* sequence from the pSAB6 plasmid to limit any undesirable effects of the genomic deletion of the H2A and H2B encoding genes.

pSAB6 containing spores were dissected, selected and mated to obtain a heterozygous strain for each locus, in which the expression of H2A and H2B is rescued by the pSAB6 plasmid. This strain was sporulated to obtain a haploid strain in the SK1 background in which both H2A H2B encoding loci are deleted (Fig. 3a). Haploids of A and alpha mating types were mated to obtain a diploid strain. Mutations were introduced by transforming each *HIS3* plasmid of the H2A H2B mutant collection. The original *URA3* plasmid was evicted by counter-selection on FOA.

### Yeast sporulation and germination screens

For the s288c strains, a culture was started in YPD for 5 h. Then 25 mL of YPA was inoculated at an OD600 of 0.1 and grown for 16 h in standard conditions. When OD is reaching 1 in YPA, yeasts were washed and transferred into 50 mL of s288c sporulation medium (Yeast Extract 0.1%, K Acetate 1%, Glucose 0.05%) with essential amino acids. Sporulation efficiency was monitored after 5 days of incubation at 30 °C on a rotating wheel by counting > 400 cells, spores and dyads.

The sporulation of SK1 mutants was induced and studied as previously described [33]. In short, yeasts are grown for 6 h in YPD and transferred in YPA at OD 0.02. They are collected 12–14 h later when reaching an OD 1 and transferred to 35 mL of sporulation medium (2% K acetate supplemented with essential amino acids) at OD 2. Sporulation efficiency was assessed after 48 h of incubation at 30 °C under agitation.

Screening the viability of spores has been performed by first frogging a constant quantity of spores. Then a secondary screen has been performed by frogging a decreasing amount of spores (Additional file 1: Figure S3). Heat shock has been performed in a water bath during 40 min at 55 °C. Ether vapor test has been performed by incubation of the frogged plates in saturating ether for 2 h at room temperature [89].

## 3D representation on the nucleosome

The yeast nucleosomal structure was obtained from PDB entry 1ID3 [81]. Residues essential for sporulation were highlighted using PyMol (The PyMOL Molecular Graphics System, Version 1.8 Schrödinger, LLC).

### Histone purification and mass spectrometry analysis

*Histone purification* The strain yJG109 in which H3 was N-terminally Flag-tagged has been used to purify histones at different stages of sporulation [33]. In brief, cells were resuspended in 600 µL of Tris 50 mM pH7.5, EDTA 1 mM, NaCl 300 mM, NP-40 0.5%, glycerol 10%, DTT 1 mM, cOmplete Protease Inhibitor Cocktail (Roche), Trichostatin A 100 nM and Phosphatase Inhibitor Cocktail (Ref P0044, Sigma) (TENG-300 buffer). Glass beads were added in the tube and cells are disrupted in a FastPrep (MP Biomedicals) for 45 s at 6.5 m s^−1^. Lysates were then sonicated 3 times 30 s with 30 s resting intervals on a Epishear sonicator (Active Motif) at 100% of intensity, representing ~ 150 kJ. Lysates were then clarified by centrifugation for 15 min at 20,000*g* for 15 min. Cell extracts were incubated with anti Flag M2 resin (A2220, Sigma) for 3 h at 4 °C under rotation. The resin has been washed four times with TENG buffer containing NaCl 500 mM, then with a final wash in TENG-300. Elution of bound proteins was performed in TENG-300 added with 0.5 mg mL^−1^ of M2 Flag peptide with 30 min of incubation under rotation at 4 °C. Supernatant was collected and the quality of histones analyzed by SDS-PAGE.

*Sample preparation and LC*–*MS/MS analysis* Purified yeast histones were migrated on a 12% polyacrylamide gel to efficiently separate the four core histones. Gel bands containing histones were cut, reduced and alkylated using dithiothreitol and iodoacetamide. They were then proteolyzed with 0.1 ug of trypsin by overnight incubation at 37 °C. The dried extracted peptides were resuspended in 2.5% acetonitrile and 0.05% trifluoroacetic acid and analyzed via online nanoLC–MS/MS using an Ultimate 3000 LC system coupled to an LTQ-Orbitrap Velos instrument (Thermo Fisher Scientific). Peptide separation was performed while starting with 96% solvent A (98% water, 2% ACN, 0.1% FA) and 4% solvent B (80% ACN, 20% water, 0.1% FA). 12% B was reached at *t* = 15 min, 30% B at *t* = 78 min, 40% at *t* = 84 min, 90% B at 85.5 min; this concentration of solvent B was held for 12 min to flush the column, before re-equilibrating it at 4% B for 18 min. After one MS full scan over the m/z range 400–1400, up to 20 peptides were selected for MS/MS fragmentation by CID in the linear ion trap. Dynamic exclusion of already fragmented m/z values was allowed for 20 s.

*MS/MS data interpretation and visual verification* Mass spectrometry RAW files produced by LC–MS/MS analysis of histone tryptic peptides were submitted to the Mascot program (version 2.5.1) via Mascot Daemon. MS/MS data acquired on yeast histones were matched to a database made up of yeast proteins from Swiss-Prot and their decoy version (13,458 protein sequences), plus a list of about 500 contaminants commonly observed in MS analyses (keratins, trypsin, etc.). The following modifications were considered as variable ones: N-terminal protein acetylation; Lys acetylation; Lys and Arg mono- and di-methylation, Lys trimethylation and Ser and Thr phosphorylation. For all Mascot searches, the tolerance on mass measurement was set to 5 ppm for peptides and to 0.6 Da for fragment ions. Up to five tryptic missed cleavages were allowed for histones H2A, H2B and H4, whereas allowing up to 3 missed cleavages was sufficient for H3. It is uncommon to consider so many variable modifications and possible tryptic missed cleavages; however, this does not impact the Mascot score indicative of the unlikelihood that the suggested sequence/spectrum match is a random event. In addition, all MS/MS spectra leading to the identification of tryptic peptides were visually examined: all major intensity fragment peaks had to be interpreted in terms of y/b ions and a minimal series of 5 continuous amino acids had to be readable by y-type or b-type fragments. The reliability of modification site positioning was also critically assessed; when a dimethylation was proposed to be present on a peptide sequence, close K and R residues were both considered to be possibly mono- or di-methylated. Finally, identifications of lower certainty (such as those corresponding to lower-abundance peptides leading to lower signal-to-noise MS/MS spectra) were supported by the identification of the non-modified peptides, by verifying that very similar fragmentation patterns were obtained in both cases (Additional file 3). Paired identifications of sequences HLQLAIR and HLQLAIRme2 of which we provide the MS/MS spectra annotated by Mascot are such examples (Additional file 3).

*MS/MS data quantification* Quantification of the abundance of modified peptides was performed using a label free method consisting of measuring the MS intensity of the corresponding chromatographic peak at the top of the curve. Normalization was done by dividing the raw MS signals of modified peptides by the signals of reference non-modified peptides for each histone, namely HLQLAIR and AGLTFPVGR for H2A, KETYSSYIYK and ETYSSYIYK for H2B, STELLIR for H3 and ISGLIYEETR and DNIQGITKPAIR for H4. Raw and normalized data are presented in Additional file 5.

### Western blot analyses and immunostaining

Analysis of yeast and mouse samples by western blot and immunostaining have been performed similarly to previously published protocols [33].

### Spore nuclear size assays

These assays have been performed as previously described [12, 33, 51, 83].

## Supplementary information

**Additional file 1.** Supplementary Figures and Tables.

**Additional file 2.** Sporulation data for s288c and SK1 backgrounds.

**Additional file 3.** MS/MS spectra of the modified histone tryptic peptides identified during yeast sporulation.

**Additional file 4.** Histone modifications in the globular domain and prediction of their effect on DNA accessibility.

**Additional file 5.** Quantification of the proteomic analysis of histone modifications.

## Data Availability

The mass spectrometry proteomics data have been deposited to the ProteomeXchange Consortium via the PRIDE partner repository with the dataset identifier PXD006213 and 10.6019/pxd006213 [90].
