# Supplementary material for: Systematic genetic and proteomic screens during gametogenesis identify H2BK34 methylation as an evolutionary conserved meiotic mark
Source: Epigenetics Chromatin. 2020 Sep 15;13:35. doi: 10.1186/s13072-020-00349-5 (PMC7493871; doi:10.1186/s13072-020-00349-5)
Supplement: Supplementary file 1 — Additional file 1. Supplementary Figures and Tables. [file 13072_2020_349_MOESM1_ESM.pdf]

Systematic genetic and proteomic screens during gametogenesis identify H2BK34 methylation as an evolutionary conserved meiotic mark

Additional File 1. Supplementary Figures and Tables

In addition, other Additional Files contain:

Additional File 2. Sporulation data for s288c and SK1 backgrounds.

Additional File 3. MS/MS spectra of the modified histone tryptic peptides identified during yeast sporulation.

Additional File 4. Histone modifications in the globular domain and prediction of their effect on DNA accessibility.

Additional File 5. Quantification of the proteomic analysis of histone modifications.

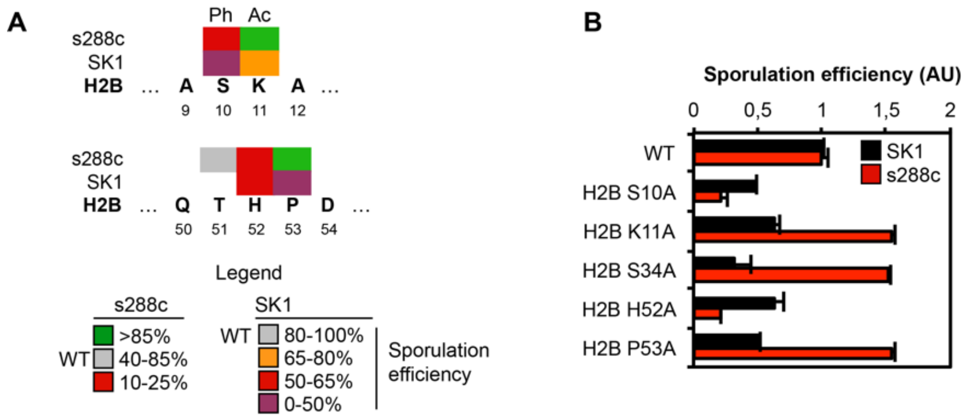

Supp. Figure S1. Binary cassettes involved in sporulation

A. Two examples of neighboring residues with opposite effects on sporulation. The mutation of H2BS10 prevents the formation in spores, whereas the mutation of the next residue, H2BK11, increases sporulation efficiency in the s288c background. This phenotype is coherent with the presence of mutually exclusive post-translational modifications on these two resides (1, 2). Acetylation of mutated H2BK11 is impossible and promotes H2BS10ph, itself essential for the formation of spores. A similar phenotype is observed upon mutation of H2BH52 and H2BP53 in the s288c genetic background but its molecular basis remains to be characterized.

B. Sporulation efficiencies in SK1 and s288c background. Data are presented in arbitrary units, with WT normalized to 1.

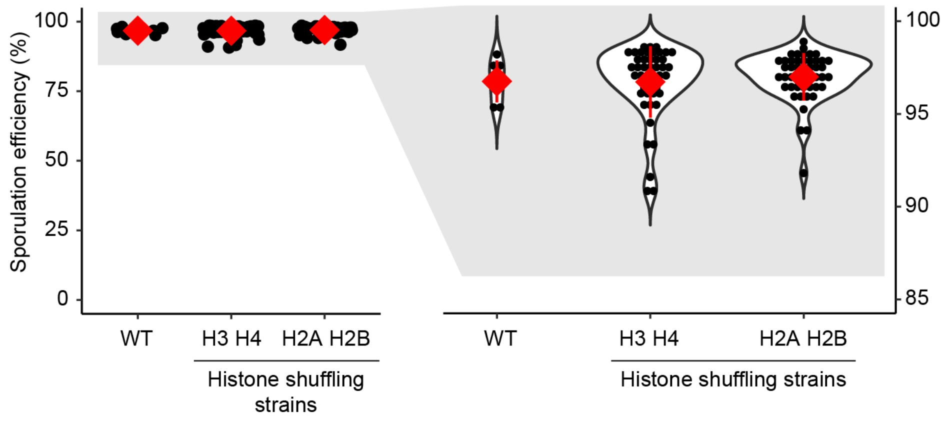

Supp. Figure S2. Sporulation efficiency is not affected in histone shuffling strains.

Left. Sporulation efficiency for WT, H3 H4 and H2A H2B shuffling strains is presented as individual data points (black dot) or averaged (red diamond). Right. Scale has been adapted to illustrate a similar data distribution between strains.

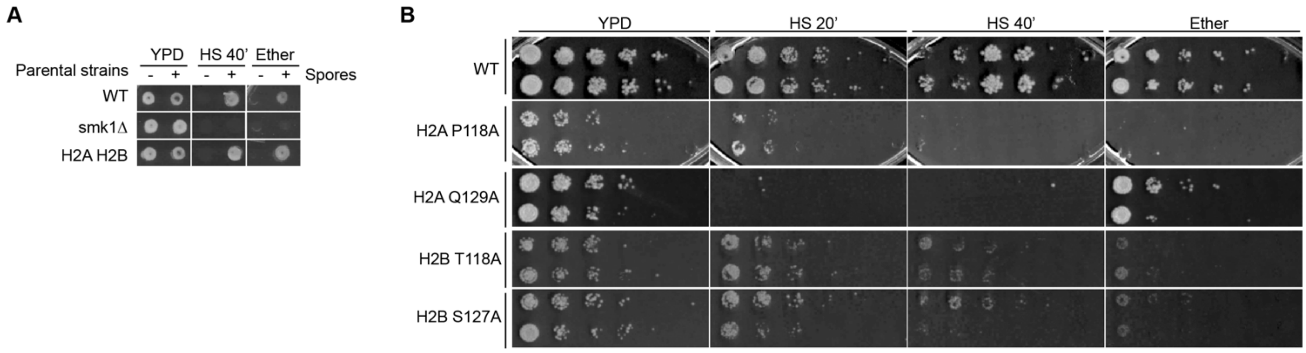

Supp Figure S3. Residues essential for stress resistance of spores in the SK1 genetic background.

A. WT spores from parental and H2A H2B shuffle strains are resistant to heat shock (HS) at 55°C during 40 min and exposure to ether.

B. H2A and H2B residues essential for the resistance of spores to heat shock (HS) or ether exposure.

Supp. Table S1. List of yeast strains

Name	Usage	Parental strain	Genetic background	Genotype	Plasmid	Source
yJG279	H2A H2B parental strain	-	SK1	*MATA/alpha ura3/" leu2::hisG/" trp1::hisG/" lys2/" ho::LYS2/" met4-445/" his3Δ/"*	pSAB6 *HTA1-HTB1* CEN ARS *URA3*	This study
yJG280	H2A H2B	yJG279	SK1	"	pJH23 *HTA1-HTB1* CEN ARS *HIS3*	This study
yJG281	Flag-H2A	yJG279	SK1	"	Derived from pJH23 with Flag-HTA1	This study
yJG282	Flag H2B	yJG279	SK1	"	Derived from pJH23 with Flag-HTB1	This study
FY406	H2A H2B	-	s288c	*MATa (hta1-htb1)Δ::LEU2, (hta2-htb2)Δ::TRP1, his3Δ200 leu2Δ1 ura3-52 trp1Δ63 lys2-128Δ*	pSAB6 *HTA1-HTB1* CEN ARS *URA3*	This study
yJG283	H2A H2B	FY406	s288c	"	pJH23 *HTA1-HTB1* CEN ARS *HIS3*	This study
yJG284	Flag-H2A	FY406	s288c	"	Derived from pJH23 with Flag-HTA1	This study
yJG285	Flag H2B	FY406	s288c	"	Derived from pJH23 with Flag-HTB1	This study
yJG109	Flag-H3	yJG52	SK1	*MATA/alpha leu2::hisG/" trp1::hisG/" lys2-SK1/" his4-N/his4-G ura3-SK1/" ho::LYS2/" hhf1-hht1::LEU2/" hhf2-hht2::trp1::KanMX3"*	*pRM204 Flag-H3 (Flag-HHT2-HHF2 CEN ARS TRP1)*	This study

The collection of histone mutants in the s288c background is derived from the SHIMA collection (3). Haploid strains of the collection have been diploidized before the sporulation screen as described in the Methods section.

The collection of histone mutants in the SK1 background has been derived from yJG279 by transforming plasmids from the SHIMA collection and selection on SC-TRP (3) followed by an eviction of the parental pSAB6 plasmid on 5-FOA.

Supp. Table S2. List of plasmids.

Name	Plasmids	Reference
pSAB6	*HTA1-HTB1* CEN ARS *URA3*	(4)
pJH23	pJH23 *HTA1-HTB1* CEN ARS *HIS3*	(4)
Flag-H2A	Derived from pJH23 with Flag-HTA1	(4)
Flag H2B	Derived from pJH23 with Flag-HTB1	(4)
Flag-H3	*pRM204 Flag-H3 (Flag-HHT2-HHF2 CEN ARS TRP1)*	(5)

Plasmids obtained from reference (3)

WT				
HTA1 S1A	HTA1 Y51A	HTA1 K123A	HTB1 R32A	HTB1 T78A
HTA1 G3A	HTA1 T53A	HTA1 T125A	HTB1 S33A	HTB1 S81A
HTA1 K4A	HTA1 R72A	HTA1 K126A	HTB1 K34A	HTB1 K82A
HTA1 G6A	HTA1 K75A	HTA1 S128A	HTB1 R36A	HTB1 Y86A
HTA1 K7A	HTA1 K76A	HTA1 Q129A	HTB1 K37A	HTB1 K88A
HTA1 S10A	HTA1 T77A	HTB1 S1A	HTB1 T39A	HTB1 K89A
HTA1 K13A	HTA1 R78A	HTB1 K3A	HTB1 Y40A	HTB1 S90A
HTA1 S15A	HTA1 I79A	HTB1 K6A	HTB1 S41A	HTB1 T91A
HTA1 Q16A	HTA1 P81A	HTB1 K7A	HTB1 S42A	HTB1 S93A
HTA1 S17A	HTA1 R82A	HTB1 P8A	HTB1 Y43A	HTB1 R95A
HTA1 R18A	HTA1 H83A	HTB1 S10A	HTB1 Y43A	HTB1 T99A
HTA1 S19A	HTA1 I88A	HTB1 K11A	HTB1 Y45A	HTB1 R102A
HTA1 K21A	HTA1 R89A	HTB1 P13A	HTB1 K46A	HTB1 P106A
HTA1 T25A	HTA1 K96A	HTB1 K16A	HTB1 K49A	HTB1 K111A
HTA1 P27A	HTA1 T102A	HTB1 K17A	HTB1 T51A	HTB1 H112A
HTA1 R30A	HTA1 Q105A	HTB1 P18A	HTB1 H52A	HTB1 S115A
HTA1 H32A	HTA1 L109A	HTB1 K21A	HTB1 P53A	HTB1 T118A
HTA1 R33A	HTA1 P110A	HTB1 K22A	HTB1 T55A	HTB1 R119A
HTA1 R36A	HTA1 H113A	HTB1 T23A	HTB1 S58A	HTB1 T122A
HTA1 R37A	HTA1 L116A	HTB1 S24A	HTB1 Q59A	HTB1 K123A
HTA1 Y40A	HTA1 L117A	HTB1 T25A	HTB1 K60A	HTB1 Y124A
HTA1 Q42A	HTA1 P118A	HTB1 S26A	HTB1 S61A	HTB1 S125A
HTA1 R43A	HTA1 K119A	HTB1 T27A	HTB1 S63A	HTB1 S126A
HTA1 S46A	HTA1 K120A	HTB1 K30A	HTB1 S67A	HTB1 S127A
HTA1 P49A	HTA1 S121A	HTB1 K31A	HTB1 R75A	HTB1 T128A

Plasmids created in this study:

HTB1 K37R

Supp. References

1. Ahn,S.-H., Henderson,K.A., Keeney,S. and Allis,C.D. (2005) H2B (Ser10) phosphorylation is induced during apoptosis and meiosis in S. cerevisiae. *Cell Cycle*, **4**, 780–783.

2. Ahn,S.-H., Diaz,R.L., Grunstein,M. and Allis,C.D. (2006) Histone H2B deacetylation at lysine 11 is required for yeast apoptosis induced by phosphorylation of H2B at serine 10. *Mol Cell*, **24**, 211–220.

3. Nakanishi,S., Sanderson,B.W., Delventhal,K.M., Bradford,W.D., Staehling-Hampton,K. and Shilatifard,A. (2008) A comprehensive library of histone mutants identifies nucleosomal residues required for H3K4 methylation. *Nat Struct Mol Biol*, **15**, 881–888.

4. Hirschhorn,J.N., Bortvin,A.L., Ricupero-Hovasse,S.L. and Winston,F. (1995) A new class of histone H2A mutations in Saccharomyces cerevisiae causes specific transcriptional defects in vivo. *Mol Cell Biol*, **15**, 1999–2009.

5. Govin,J., Dorsey,J., Gaucher,J., Rousseaux,S., Khochbin,S. and Berger,S.L. (2010) Systematic screen reveals new functional dynamics of histones H3 and H4 during gametogenesis. *Genes Dev*, **24**, 1772–1786.
