# Supplementary material for: Systematic genetic and proteomic screens during gametogenesis identify H2BK34 methylation as an evolutionary conserved meiotic mark
Source: Epigenetics Chromatin. 2020 Sep 15;13:35. doi: 10.1186/s13072-020-00349-5 (PMC7493871; doi:10.1186/s13072-020-00349-5)
Supplement: Supplementary file 3 — Additional file 3. MS/MS spectra of the modified histone tryptic peptides identified during yeast sporulation. [file 13072_2020_349_MOESM3_ESM.pdf]

Mascot Search Results

Peptide View

MS/MS Fragmentation of **SGGKGGKAGSAAK**
Found in **H2A1_YEAST** in **S_cerevisiae_D**, sp|P04911|H2A1_YEAST Histone H2A.1 OS=Saccharomyces cerevisiae (strain ATCC 204508 / S288c) GN=HTA1 PE=1 SV=2

Match to Query 87289: 1200.609848 from(601.312200,2+) intensity(43510.7890) scans(1394) rawscans(sn1394) rtinseconds(880.3948) index(83479)
Title: 979: Scan 1394 (rt=14.6732) [D:\MSData\All\VELOS23968.raw]
Data file D:\Data\MGF\530 Final H2A H2B yeast classical PTMs\mascot_daemon_merge.mgf

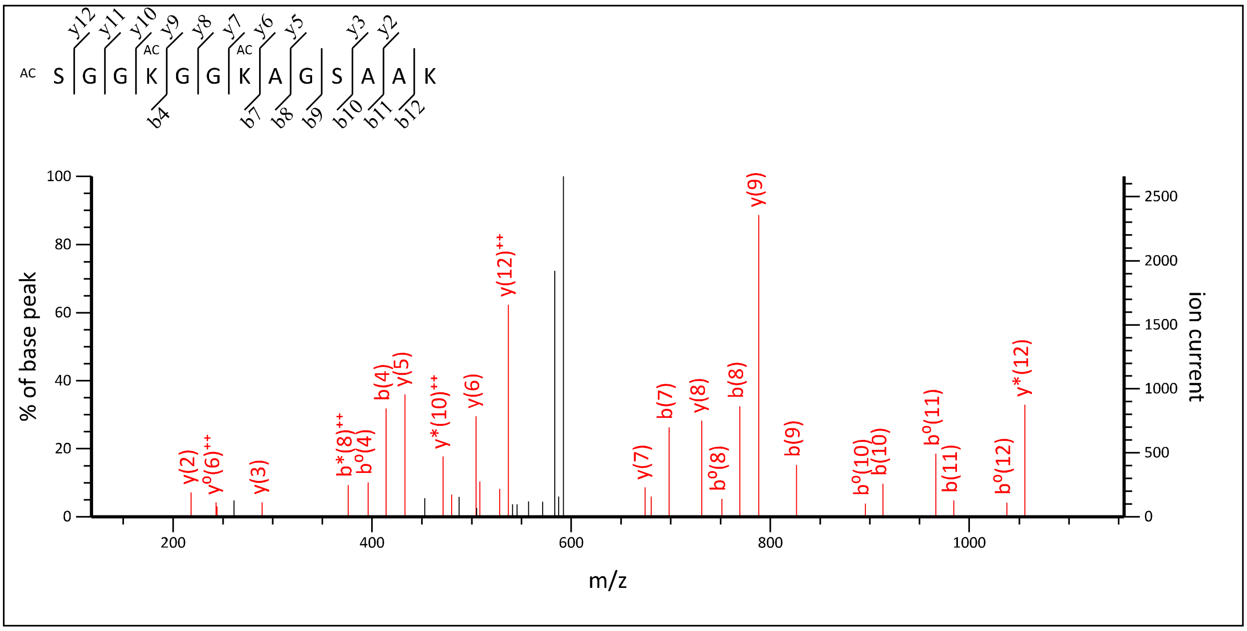

118.08 to 1155.57

Label all possible matches Label matches used for scoring

Monoisotopic mass of neutral peptide Mr(calc): 1200.6098
Fixed modifications: Carbamidomethyl (C) (apply to specified residues or termini only)
Variable modifications:
N-term : Acetyl (Protein N-term)
K4 : Acetyl (K)
K7 : Acetyl (K)
Ions Score: 107 **Expect:** 1.4e-09
Matches : 38/132 fragment ions using 32 most intense peaks (help)

#	b	b^{++}	b*	b^{*++}	b^0	b^{0++}	Seq.	y	y^{++}	y*	y^{*++}	y^0	y^{0++}	#
1	130.0499	65.5286			112.0393	56.5233	S							13
2	187.0713	94.0393			169.0608	85.0340	G	1072.5746	**536.7909**	1055.5480	528.2776	1054.5640	527.7856	12
3	***244.0928***	122.5500			226.0822	113.5448	G	1015.5531	**508.2802**	998.5265	499.7669	997.5425	499.2749	11
4	***414.1983***	207.6028	397.1718	199.0895	396.1878	198.5975	K	958.5316	**479.7694**	941.5051	471.2562	940.5211	470.7642	10
5	***471.2198***	236.1135	454.1932	227.6003	453.2092	227.1082	G	**788.4261**	394.7167	771.3995	386.2034	770.4155	385.7114	9
6	***528.2413***	264.6243	511.2147	256.1110	510.2307	255.6190	G	**731.4046**	366.2060	714.3781	357.6927	713.3941	357.2007	8
7	***698.3468***	349.6770	681.3202	341.1638	680.3362	340.6717	K	**674.3832**	337.6952	657.3566	329.1819	656.3726	328.6899	7
8	***769.3839***	385.1956	752.3573	376.6823	751.3733	376.1903	A	**504.2776**	252.6425	487.2511	244.1292	486.2671	243.6372	6
9	***826.4054***	**413.7063**	809.3788	405.1930	808.3948	404.7010	G	**433.2405**	217.1239	416.2140	208.6106	415.2300	208.1186	5
10	***913.4374***	457.2223	896.4108	448.7091	895.4268	448.2170	S	**376.2191**	188.6132	359.1925	180.0999	358.2085	179.6079	4
11	***984.4745***	492.7409	967.4480	484.2276	966.4639	483.7356	A	**289.1870**	145.0972	272.1605	136.5839			3
12	***1055.5116***	**528.2594**	1038.4851	519.7462	1037.5011	519.2542	A	**218.1499**	109.5786	201.1234	101.0653			2
13							K	147.1128	74.0600	130.0863	65.5468			1

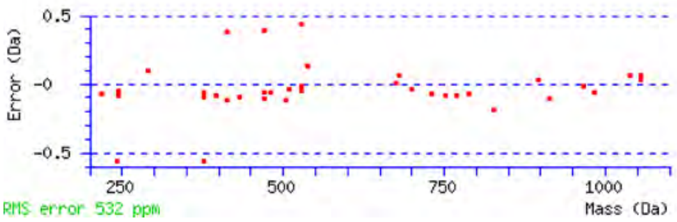

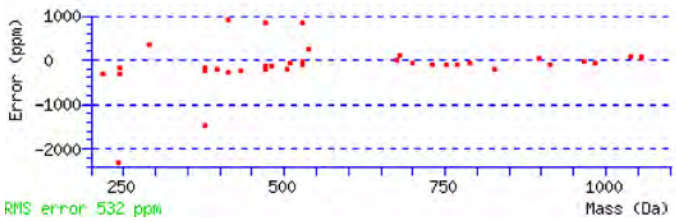

NCBI **BLAST** search of SGGKGGKAGSAAK
(Parameters: blastp, nr protein database, expect=20000, no filter, PAM30)
Other BLAST web gateways

All matches to this query

Score	Mr(calc)	Delta	Sequence	Site Analysis
106.7	1200.6098	0.0000	SGGKGGKAGSAAK	Acetyl N-term, K4, K7 100.00%
16.9	1200.6098	0.0000	SGGKGGKAGSAAK	Acetyl N-term, K4, K13 0.00%
12.3	1200.6098	0.0000	NKERDEKAK	
10.9	1200.6155	-0.0057	ITFLTQKSK	
9.8	1200.6155	-0.0057	VKLTDFGTAK	
8.9	1200.6155	-0.0057	ITFLTQKSK	
8.8	1200.6155	-0.0057	IKLTDFGTAK	
8.7	1200.6155	-0.0056	TKYDKIAAK	
8.6	1200.6128	-0.0030	SHKARELPR	
8.3	1200.6071	0.0027	ENRGRREDR	

Mascot: http://www.matrixscience.com/

Mascot Search Results

Peptide View

MS/MS Fragmentation of **SGGKGGKAGSAAK**
Found in **H2A1_YEAST** in **S_cerevisiae_D**, sp|P04911|H2A1_YEAST Histone H2A.1 OS=Saccharomyces cerevisiae (strain ATCC 204508 / S288c) GN=HTA1 PE=1 SV=2

Match to Query 103322: 1280.576708 from(641.295630,2+) intensity(13638.3540) scans(1873) rawscans(sn1873) rtinseconds(1019.1969) index(42439)
Title: 1410: Scan 1873 (rt=16.9866) [D:\MSData\All\VELOS23966.raw]
Data file D:\Data\MGF\530 Final H2A H2B yeast classical PTMs\mascot_daemon_merge.mgf

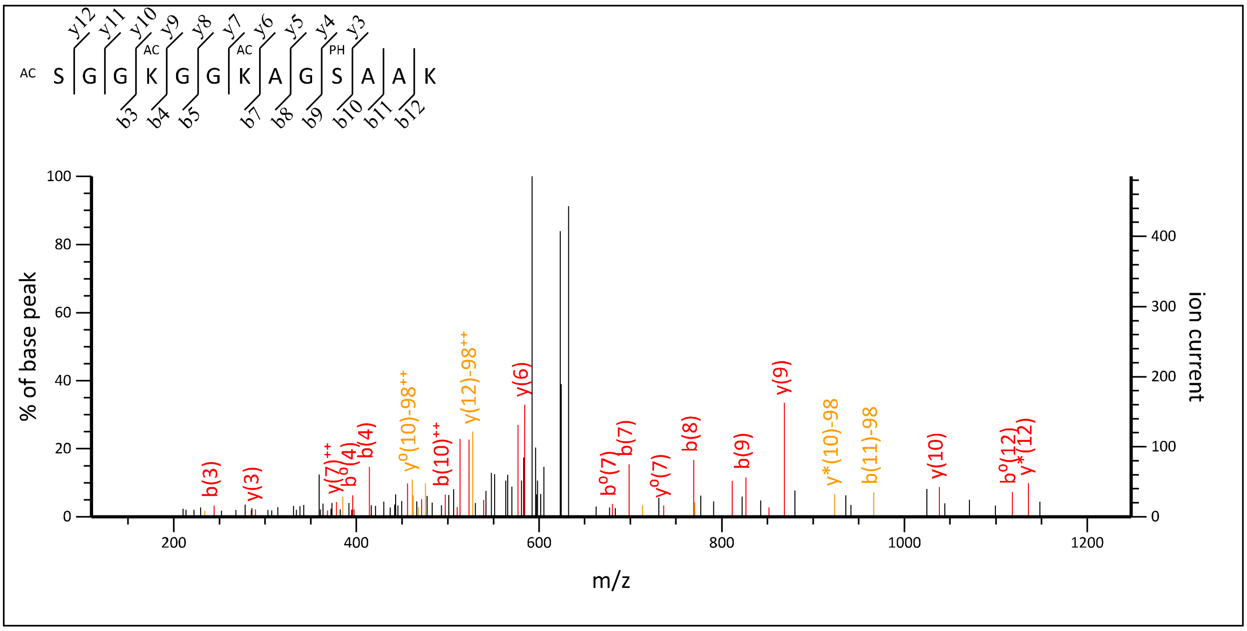

110.04 to 1248.1

Label all possible matches ◉ Label matches used for scoring ○

```
Monoisotopic mass of neutral peptide Mr(calc): 1280.5762
Fixed modifications: Carbamidomethyl (C) (apply to specified residues or termini only)
Variable modifications:
N-term : Acetyl (Protein N-term)
K4     : Acetyl (K)
K7     : Acetyl (K)
S10    : Phospho (ST), with neutral losses 0.0000(shown in table), 97.9769
Ions Score: 30  Expect: 0.012   (help)
```

#	b	b^{++}	b*	b^{*++}	b^0	b^{0++}	Seq.	y	y^{++}	y*	y^{*++}	y^0	y^{0++}	#
1	130.0499	65.5286			112.0393	56.5233	S							13
2	187.0713	94.0393			169.0608	85.0340	G	1152.5409	**576.7741**	**1135.5143**	568.2608	1134.5303	567.7688	12
3	**244.0928**	122.5500			226.0822	113.5448	G	1095.5194	548.2633	1078.4929	539.7501	1077.5089	**539.2581**	11
4	**414.1983**	207.6028	**397.1718**	199.0895	**396.1878**	198.5975	K	**1038.4980**	519.7526	1021.4714	511.2393	1020.4874	**510.7473**	10
5	**471.2198**	236.1135	454.1932	227.6003	453.2092	227.1082	G	**868.3924**	434.6999	**851.3659**	426.1866	850.3819	425.6946	9
6	528.2413	264.6243	511.2147	256.1110	**510.2307**	255.6190	G	**811.3710**	406.1891	794.3444	**397.6758**	793.3604	**397.1838**	8
7	**698.3468**	349.6770	681.3202	341.1638	**680.3362**	340.6717	K	754.3495	**377.6784**	737.3229	369.1651	**736.3389**	**368.6731**	7
8	**769.3839**	**385.1956**	752.3573	376.6823	751.3733	376.1903	A	**584.2440**	292.6256	567.2174	**284.1123**	566.2334	283.6203	6
9	**826.4054**	**413.7063**	809.3788	405.1930	808.3948	404.7010	G	**513.2069**	257.1071	496.1803	248.5938	495.1963	248.1018	5
10	993.4037	**497.2055**	976.3772	488.6922	975.3932	488.2002	S	**456.1854**	228.5963	439.1588	220.0831	438.1748	219.5911	4
11	1064.4408	532.7241	1047.4143	524.2108	1046.4303	**523.7188**	A	**289.1870**	145.0972	272.1605	136.5839			3
12	**1135.4779**	568.2426	1118.4514	559.7293	**1117.4674**	559.2373	A	218.1499	109.5786	201.1234	101.0653			2
13							K	147.1128	74.0600	130.0863	65.5468			1

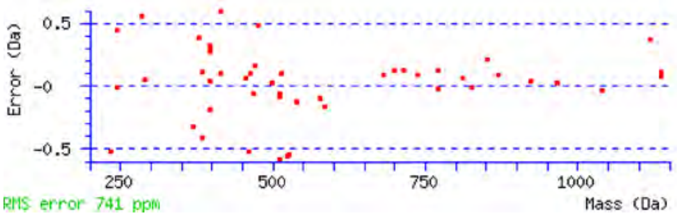

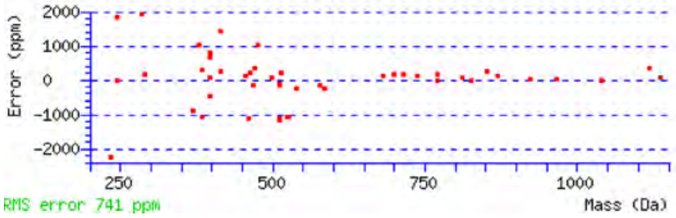

NCBI **BLAST** search of SGGKGGKAGSAAK
(Parameters: blastp, nr protein database, expect=20000, no filter, PAM30)
Other BLAST web gateways

All matches to this query

Score	Mr(calc)	Delta	Sequence	Site Analysis
29.9	1280.5762	0.0006	SGGKGGKAGSAAK	Acetyl N-term, K4, K7, Phospho S10; 97.44%
8.8	1280.5762	0.0006	SGGKGGKAGSAAK	Acetyl N-term, K4, K13, Phospho S10; 0.76%
8.8	1280.5762	0.0006	DKSQNPASNGK	
8.6	1280.5802	-0.0035	NNKKSISYYG	
8.2	1280.5802	-0.0035	NNKKSISYYG	
8.2	1280.5818	-0.0051	KDKTLSLFK	
8.0	1280.5802	-0.0035	SKYETHAPVK	
7.8	1280.5802	-0.0035	NNKKSISYYG	
6.7	1280.5802	-0.0035	NNKKSISYYG	
5.8	1280.5713	0.0054	RASKSSGKMK	

Mascot: http://www.matrixscience.com/

MATRIX SCIENCE Mascot Search Results

Peptide View

MS/MS Fragmentation of **GGKAGSAAKASQSR**
Found in **H2A1_YEAST** in **S_cerevisiae_D**, sp|P04911|H2A1_YEAST Histone H2A.1 OS=Saccharomyces cerevisiae (strain ATCC 204508 / S288c) GN=HTA1 PE=1 SV=2

Match to Query 121751: 1358.689128 from(680.351840,2+) intensity(8178.7710) scans(1265) rawscans(sn1265) rtinseconds(805.8807) index(214650)
Title: 912: Scan 1265 (rt=13.4313) [D:\MSData\All\VELOS23658.raw]
Data file D:\Data\MGF\530 Final H2A H2B yeast classical PTMs\mascot_daemon_merge.mgf

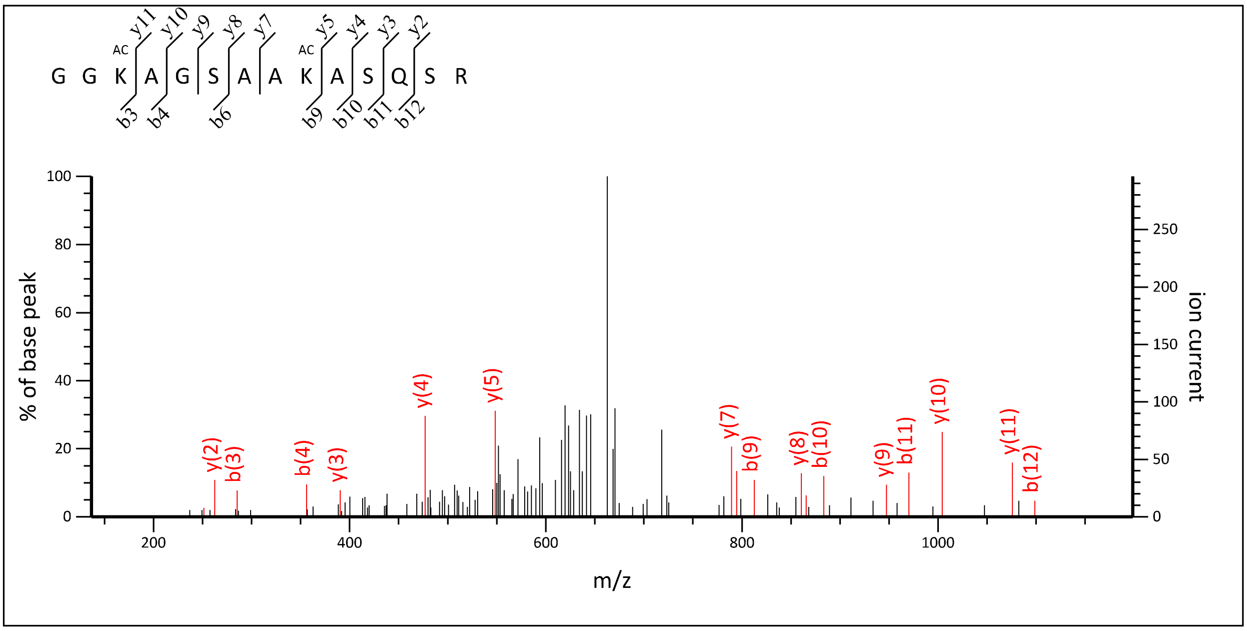

136.7 to 1198.45

Label all possible matches ○ Label matches used for scoring ◉

```
Monoisotopic mass of neutral peptide Mr(calc): 1358.6902
Fixed modifications: Carbamidomethyl (C) (apply to specified residues or termini only)
Variable modifications:
K3     : Acetyl (K)
K9     : Acetyl (K)
Ions Score: 66  Expect: 3.8e-06
Matches : 20/140 fragment ions using 27 most intense peaks   (help)
```

#	b	b^{++}	b*	b^{*++}	b^0	b^{0++}	Seq.	y	y^{++}	y*	y^{*++}	y^0	y^{0++}	#
1	58.0287	29.5180					G							14
2	115.0502	58.0287					G	1302.6761	651.8417	1285.6495	643.3284	1284.6655	642.8364	13
3	**285.1557**	143.0815	268.1292	134.5682			K	1245.6546	623.3309	1228.6280	614.8177	1227.6440	614.3257	12
4	**356.1928**	178.6001	339.1663	170.0868			A	***1075.5491***	538.2782	1058.5225	529.7649	1057.5385	529.2729	11
5	413.2143	207.1108	396.1878	198.5975			G	***1004.5119***	502.7596	987.4854	494.2463	986.5014	493.7543	10
6	500.2463	250.6268	483.2198	242.1135	482.2358	241.6215	S	***947.4905***	474.2489	930.4639	465.7356	929.4799	465.2436	9
7	571.2835	286.1454	554.2569	277.6321	553.2729	277.1401	A	***860.4585***	430.7329	843.4319	422.2196	842.4479	421.7276	8
8	642.3206	321.6639	625.2940	313.1506	624.3100	312.6586	A	***789.4213***	395.2143	772.3948	386.7010	771.4108	386.2090	7
9	**812.4261**	406.7167	795.3995	398.2034	794.4155	397.7114	K	718.3842	359.6958	701.3577	351.1825	700.3737	350.6905	6
10	**883.4632**	442.2352	866.4367	433.7220	865.4526	433.2300	A	***548.2787***	274.6430	531.2522	266.1297	530.2681	265.6377	5
11	**970.4952**	485.7513	953.4687	477.2380	952.4847	476.7460	S	***477.2416***	239.1244	460.2150	230.6112	459.2310	230.1191	4
12	**1098.5538**	549.7805	1081.5273	541.2673	1080.5432	540.7753	Q	***390.2096***	195.6084	373.1830	187.0951	372.1990	186.6031	3
13	1185.5858	593.2966	1168.5593	584.7833	1167.5753	584.2913	S	***262.1510***	131.5791	245.1244	123.0659	244.1404	122.5738	2
14							R	175.1190	88.0631	158.0924	79.5498			1

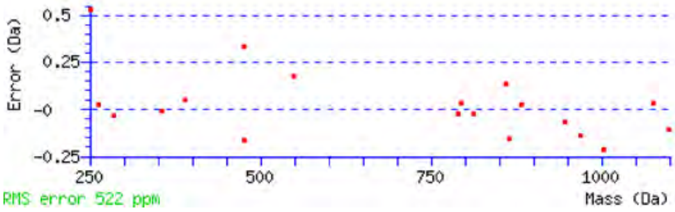

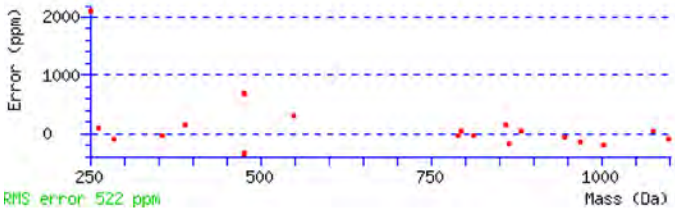

NCBI **BLAST** search of GGKAGSAAKASQSR
(Parameters: blastp, nr protein database, expect=20000, no filter, PAM30)
Other BLAST web gateways

All matches to this query

Score	Mr(calc)	Delta	Sequence
66.1	1358.6902	-0.0011	GGKAGSAAKASQSR
7.8	1358.6902	-0.0011	HSSTSRNLSNSK
7.6	1358.6902	-0.0011	RNNTRSDEPTK
7.4	1358.6830	0.0061	YIYNTNNSKSK
6.8	1358.6902	-0.0011	KRAAGSGESTPER
6.1	1358.6959	-0.0068	YILSQVKVSSR
5.9	1358.6959	-0.0067	VKAGTYSASRK
5.9	1358.6959	-0.0067	VKAGTYSASRK
4.3	1358.6959	-0.0067	KKYADISRSK
4.3	1358.6959	-0.0067	KKYADISRSK

Mascot: http://www.matrixscience.com/

MATRIX SCIENCE Mascot Search Results

Peptide View

MS/MS Fragmentation of **SAKAGLTFPVGR**
Found in **H2A1_YEAST** in **S_cerevisiae_D**, sp|P04911|H2A1_YEAST Histone H2A.1 OS=Saccharomyces cerevisiae (strain ATCC 204508 / S288c) GN=HTA1 PE=1 SV=2

Match to Query 95901: 1244.686948 from(623.350750,2+) intensity(44986.5390) scans(6109) rawscans(sn6109) rtinseconds(2261.7605) index(16970)
Title: 4901: Scan 6109 (rt=37.696) [D:\MSData\All\VELOS23962.raw]
Data file D:\Data\MGF\530 Final H2A H2B yeast classical PTMs\mascot_daemon_merge.mgf

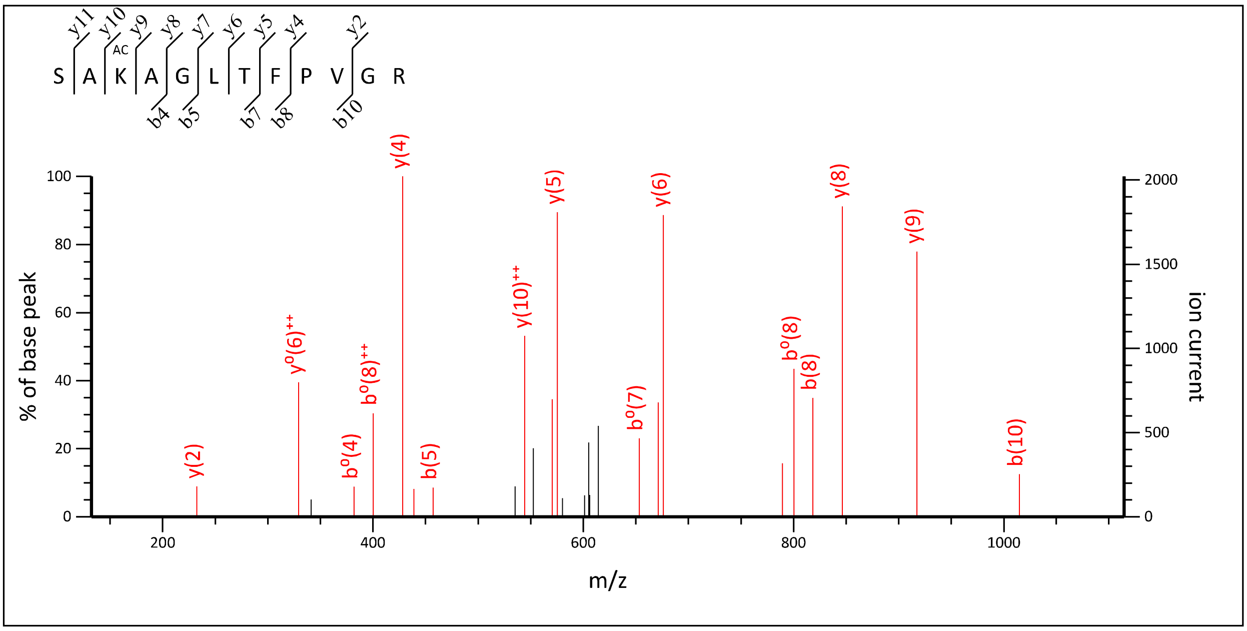

132.26 to 1114.64

Label all possible matches Label matches used for scoring

```
Monoisotopic mass of neutral peptide Mr(calc): 1244.6877
Fixed modifications: Carbamidomethyl (C) (apply to specified residues or termini only)
Variable modifications:
K3     : Acetyl (K)
Ions Score: 74  Expect: 2.2e-06
Matches : 22/118 fragment ions using 19 most intense peaks   (help)
```

#	b	b^{++}	b*	b^{*++}	b^0	b^{0++}	Seq.	y	y^{++}	y*	y^{*++}	y^0	y^{0++}	#
1	88.0393	44.5233			70.0287	35.5180	S							12
2	159.0764	80.0418			141.0659	71.0366	A	1158.6630	579.8351	1141.6364	571.3218	1140.6524	570.8298	11
3	***329.1819***	165.0946	312.1554	156.5813	311.1714	156.0893	K	1087.6259	544.3166	1070.5993	535.8033	1069.6153	535.3113	10
4	***400.2191***	200.6132	383.1925	192.0999	382.2085	191.6079	A	***917.5203***	459.2638	900.4938	450.7505	899.5098	450.2585	9
5	***457.2405***	229.1239	440.2140	220.6106	439.2300	220.1186	G	***846.4832***	423.7452	829.4567	415.2320	828.4726	414.7400	8
6	***570.3246***	285.6659	553.2980	277.1527	552.3140	276.6606	L	***789.4618***	395.2345	772.4352	386.7212	771.4512	386.2292	7
7	***671.3723***	336.1898	654.3457	327.6765	653.3617	327.1845	T	***676.3777***	338.6925	659.3511	330.1792	658.3671	329.6872	6
8	***818.4407***	409.7240	801.4141	401.2107	800.4301	400.7187	F	***575.3300***	288.1686	558.3035	279.6554			5
9	915.4934	458.2504	898.4669	449.7371	897.4829	449.2451	P	***428.2616***	214.6344	411.2350	206.1212			4
10	***1014.5619***	507.7846	997.5353	499.2713	996.5513	498.7793	V	331.2088	166.1081	314.1823	157.5948			3
11	1071.5833	536.2953	1054.5568	527.7820	1053.5728	527.2900	G	***232.1404***	116.5738	215.1139	108.0606			2
12							R	175.1190	88.0631	158.0924	79.5498			1

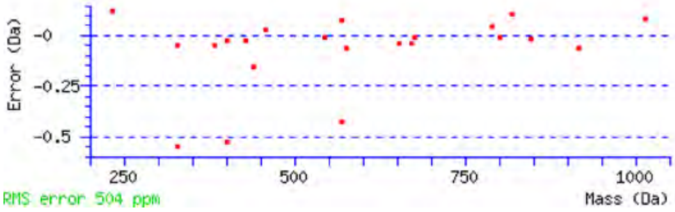

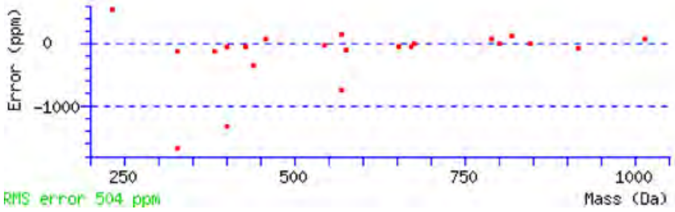

NCBI **BLAST** search of SAKAGLTFPVGR
(Parameters: blastp, nr protein database, expect=20000, no filter, PAM30)
Other BLAST web gateways

All matches to this query

Score	Mr(calc)	Delta	Sequence
74.2	1244.6877	-0.0008	SAKAGLTFPVGR
13.0	1244.6911	-0.0041	KEGKIVNMER
10.6	1244.6877	-0.0008	KADQLYPGKR
10.6	1244.6837	0.0033	DKRDKEEKR
10.6	1244.6837	0.0033	DKRDKEEKR
10.2	1244.6911	-0.0041	EAKQIMRSVK
9.9	1244.6837	0.0033	DKRDKEEKR
9.9	1244.6837	0.0033	DKRDKEEKR
9.9	1244.6837	0.0033	DKRDKEEKR
9.9	1244.6837	0.0033	DKRDKEEKR

Mascot: http://www.matrixscience.com/

MATRIX SCIENCE Mascot Search Results

Peptide View

MS/MS Fragmentation of **SAKAGLTFPVGR**
Found in **H2A1_YEAST** in **S_cerevisiae_D**, sp|P04911|H2A1_YEAST Histone H2A.1 OS=Saccharomyces cerevisiae (strain ATCC 204508 / S288c) GN=HTA1 PE=1 SV=2

Match to Query 90615: 1216.692042 from(406.571290,3+) intensity(48575.9300) scans(3521) rawscans(sn3521) rtinseconds(1610.1544) index(2673)
Title: 2674: Scan 3521 (rt=26.8359) [D:\MSData\All\VELOS23662.raw]
Data file D:\Data\MGF\530 Final H2A H2B yeast classical PTMs\mascot_daemon_merge.mgf

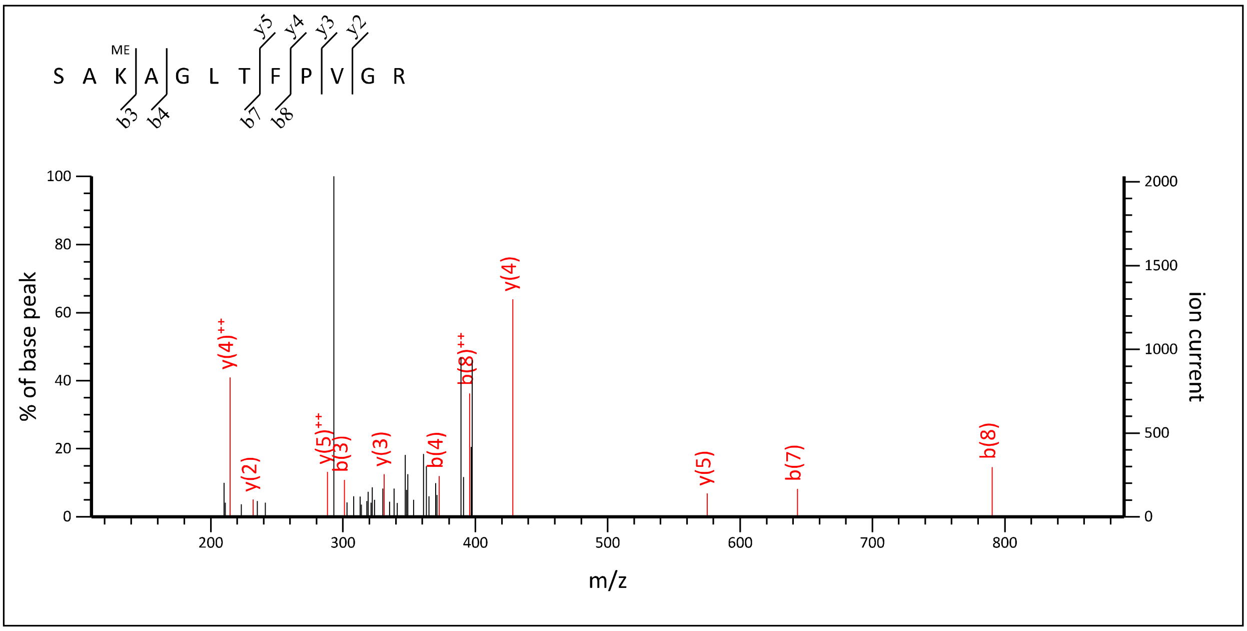

109.95 to 890.33

Label all possible matches ○ Label matches used for scoring ◉

Monoisotopic mass of neutral peptide Mr(calc): 1216.6928
Fixed modifications: Carbamidomethyl (C) (apply to specified residues or termini only)
Variable modifications:
K3 : Methyl (K)
Ions Score: 20 **Expect:** 0.71
Matches : 12/118 fragment ions using 24 most intense peaks (help)

#	b	b^{++}	b*	b^{*++}	b^{0}	b^{0++}	Seq.	y	y^{++}	y*	y^{*++}	y^{0}	y^{0++}	#
1	88.0393	44.5233			70.0287	35.5180	S							12
2	159.0764	80.0418			141.0659	71.0366	A	1130.6681	565.8377	1113.6415	557.3244	1112.6575	556.8324	11
3	***301.1870***	151.0972	284.1605	142.5839	283.1765	142.0919	K	1059.6309	530.3191	1042.6044	521.8058	1041.6204	521.3138	10
4	***372.2241***	186.6157	355.1976	178.1024	354.2136	177.6104	A	917.5203	459.2638	900.4938	450.7505	899.5098	450.2585	9
5	429.2456	215.1264	412.2191	206.6132	411.2350	206.1212	G	846.4832	423.7452	829.4567	415.2320	828.4726	414.7400	8
6	542.3297	271.6685	525.3031	263.1552	524.3191	262.6632	L	789.4618	**395.2345**	772.4352	386.7212	771.4512	386.2292	7
7	***643.3774***	322.1923	626.3508	313.6790	625.3668	313.1870	T	676.3777	338.6925	659.3511	330.1792	658.3671	329.6872	6
8	***790.4458***	**395.7265**	773.4192	387.2132	772.4352	386.7212	F	***575.3300***	**288.1686**	558.3035	279.6554			5
9	887.4985	444.2529	870.4720	435.7396	869.4880	435.2476	P	***428.2616***	**214.6344**	411.2350	206.1212			4
10	986.5669	493.7871	969.5404	485.2738	968.5564	484.7818	V	***331.2088***	166.1081	314.1823	157.5948			3
11	1043.5884	522.2978	1026.5619	513.7846	1025.5778	513.2926	G	***232.1404***	116.5738	215.1139	108.0606			2
12							R	175.1190	88.0631	158.0924	79.5498			1

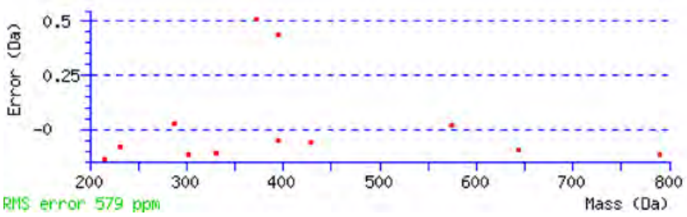

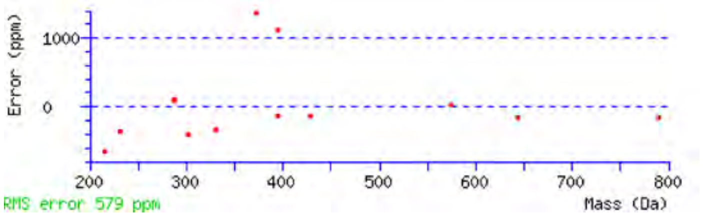

NCBI **BLAST** search of SAKAGLTFPVGR
(Parameters: blastp, nr protein database, expect=20000, no filter, PAM30)
Other BLAST web gateways

All matches to this query

Score	Mr(calc)	Delta	Sequence
20.3	1216.6928	-0.0008	SAKAGLTFPVGR
17.6	1216.6962	-0.0041	DAKVKMGGTVR
12.9	1216.6928	-0.0007	ASINSKFAGPR
10.8	1216.6896	0.0024	TMRMNKRPK
10.8	1216.6896	0.0024	TMRMNKRPK
10.8	1216.6896	0.0024	TMRMNKRPK
9.7	1216.6961	-0.0041	LGEKEGKRMK
9.7	1216.6961	-0.0041	LGEKEGKRMK
9.7	1216.6962	-0.0041	LGEKEGKRMK
8.5	1216.6927	-0.0007	KEKFRKQK

Mascot: http://www.matrixscience.com/

Mascot Search Results

Peptide View

MS/MS Fragmentation of **AGLTFPVGR**
Found in **H2A1_YEAST** in **S_cerevisiae_D**, sp|P04911|H2A1_YEAST Histone H2A.1 OS=Saccharomyces cerevisiae (strain ATCC 204508 / S288c) GN=HTA1 PE=1 SV=2

Match to Query 33322: 930.527928 from(466.271240,2+) intensity(27740.6410) scans(6393) rawscans(sn6393) rtinseconds(2137.2181) index(247569)
Title: 5368: Scan 6393 (rt=35.6203) [D:\MSData\All\VELOS23976.raw]
Data file D:\Data\MGF\530 Final H2A H2B yeast classical PTMs\mascot_daemon_merge.mgf

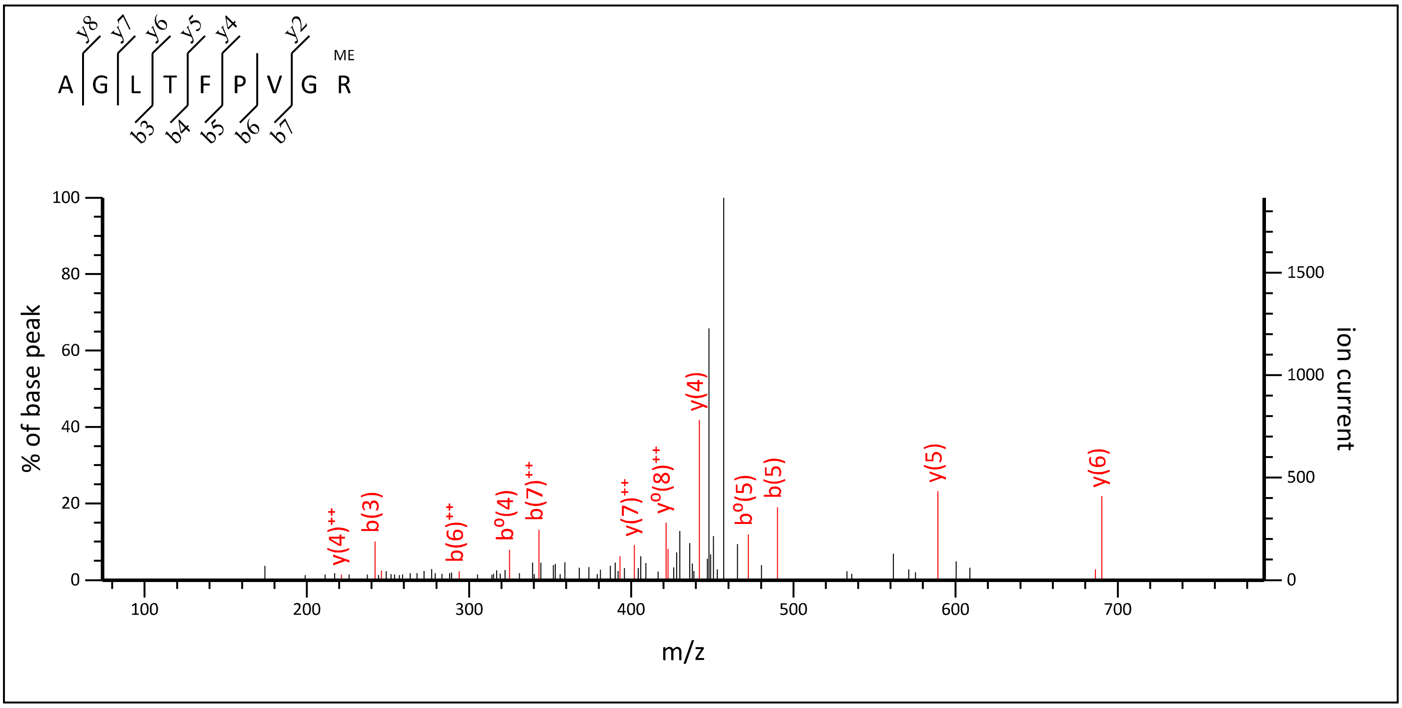

74.11 to 790.26

Label all possible matches ◉ Label matches used for scoring ○

Monoisotopic mass of neutral peptide Mr(calc): 930.5287
Fixed modifications: Carbamidomethyl (C) (apply to specified residues or termini only)
Variable modifications:
R9 : Methyl (R)
Ions Score: 30 **Expect:** 0.08 (help)

#	b	b++	b0	b0++	Seq.	y	y++	y*	y*++	y0	y0++	#
1	72.0444	36.5258			A							9
2	129.0659	65.0366			G	860.4989	430.7531	843.4723	**422.2398**	842.4883	**421.7478**	8
3	**242.1499**	121.5786			L	803.4774	**402.2423**	786.4509	393.7291	785.4668	**393.2371**	7
4	**343.1976**	172.1024	**325.1870**	163.0972	T	**690.3933**	345.7003	673.3668	337.1870	672.3828	336.6950	6
5	**490.2660**	**245.6366**	**472.2554**	236.6314	F	**589.3457**	295.1765	572.3191	286.6632			5
6	587.3188	**294.1630**	569.3082	285.1577	P	**442.2772**	**221.6423**	425.2507	213.1290			4
7	**686.3872**	**343.6972**	668.3766	334.6920	V	345.2245	173.1159	328.1979	164.6026			3
8	743.4087	372.2080	725.3981	363.2027	G	**246.1561**	123.5817	229.1295	115.0684			2
9					R	189.1346	95.0709	172.1081	86.5577			1

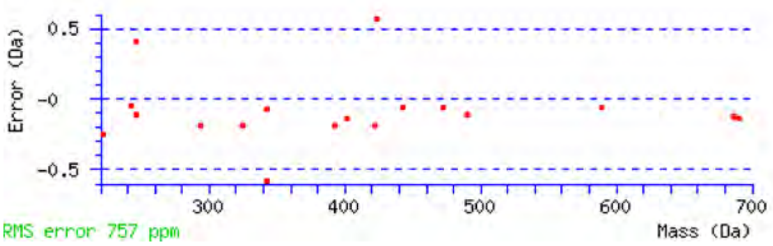

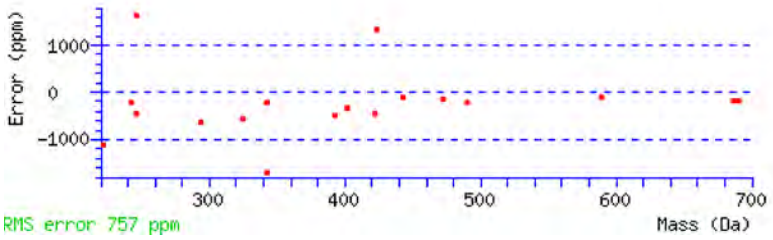

NCBI **BLAST** search of AGLTFPVGR
(Parameters: blastp, nr protein database, expect=20000, no filter, PAM30)
Other BLAST web gateways

All matches to this query

Score	Mr(calc)	Delta	Sequence
29.6	930.5287	-0.0008	AGLTFPVGR
18.2	930.5287	-0.0007	AREFAAPK
15.5	930.5247	0.0033	RVSNQTGK
13.3	930.5246	0.0033	IKTREER
10.4	930.5320	-0.0041	KLELLCR
9.7	930.5287	-0.0008	SLKVEWR
7.9	930.5320	-0.0041	MKINVSR
7.2	930.5287	-0.0007	TKAHNYK
7.2	930.5246	0.0033	LASASRQAK
7.2	930.5247	0.0033	GRVTNSNK

Mascot: http://www.matrixscience.com/

MATRIX SCIENCE Mascot Search Results

Peptide View

MS/MS Fragmentation of **AGLTFPVGR**
Found in **H2A1_YEAST** in **S_cerevisiae_D**, sp|P04911|H2A1_YEAST Histone H2A.1 OS=Saccharomyces cerevisiae (strain ATCC 204508 / S288c) GN=HTA1 PE=1 SV=2

Match to Query 36238: 944.543888 from(473.279220,2+) intensity(121521.2500) scans(7942) rawscans(sn7942) rtinseconds(2760.6603) index(220216)
Title: 6478: Scan 7942 (rt=46.011) [D:\MSData\All\VELOS23658.raw]
Data file D:\Data\MGF\530 Final H2A H2B yeast classical PTMs\mascot_daemon_merge.mgf

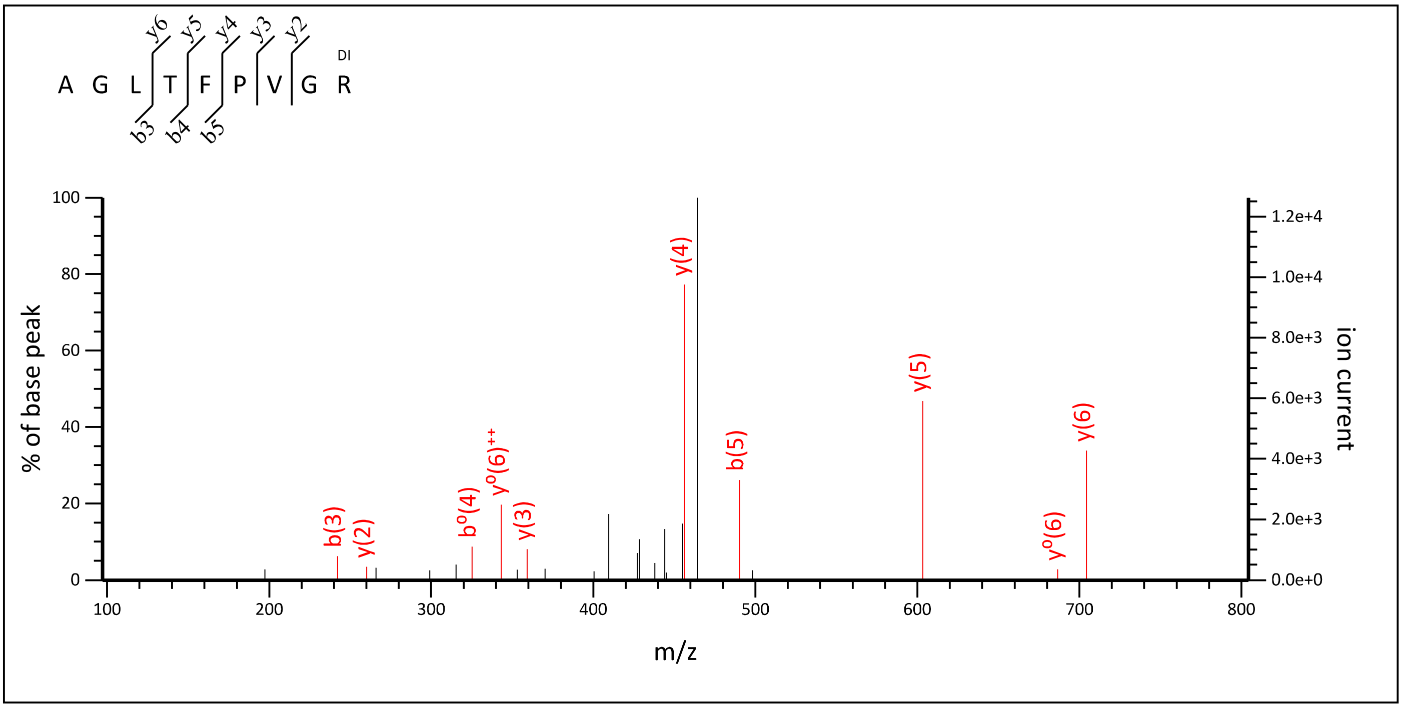

97.21 to 804.38

Label all possible matches ○ Label matches used for scoring ◉

Monoisotopic mass of neutral peptide Mr(calc): 944.5444
Fixed modifications: Carbamidomethyl (C) (apply to specified residues or termini only)
Variable modifications:
R9 : Dimethyl (R)
Ions Score: 45 **Expect:** 0.0025
Matches : 13/64 fragment ions using 13 most intense peaks (help)

#	b	b^{++}	b^{0}	b^{0++}	Seq.	y	y^{++}	y*	y^{*++}	y^{0}	y^{0++}	#
1	72.0444	36.5258			A							9
2	129.0659	65.0366			G	874.5145	437.7609	857.4880	429.2476	856.5040	428.7556	8
3	**242.1499**	121.5786			L	817.4931	409.2502	800.4665	400.7369	799.4825	400.2449	7
4	**343.1976**	172.1024	325.1870	163.0972	T	***704.4090***	352.7081	687.3824	344.1949	686.3984	343.7028	6
5	**490.2660**	245.6366	472.2554	236.6314	F	***603.3613***	302.1843	586.3348	293.6710			5
6	587.3188	294.1630	569.3082	285.1577	P	***456.2929***	228.6501	439.2663	220.1368			4
7	**686.3872**	343.6972	668.3766	334.6920	V	***359.2401***	180.1237	342.2136	171.6104			3
8	743.4087	372.2080	725.3981	363.2027	G	***260.1717***	130.5895	243.1452	122.0762			2
9					R	203.1503	102.0788	186.1237	93.5655			1

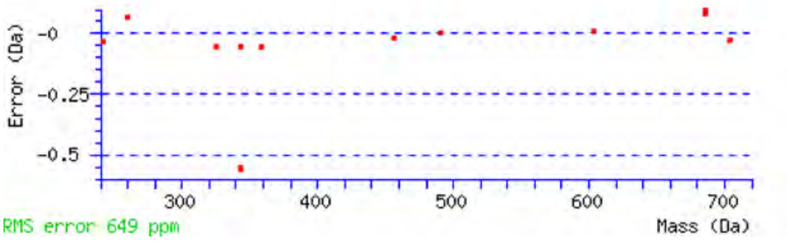

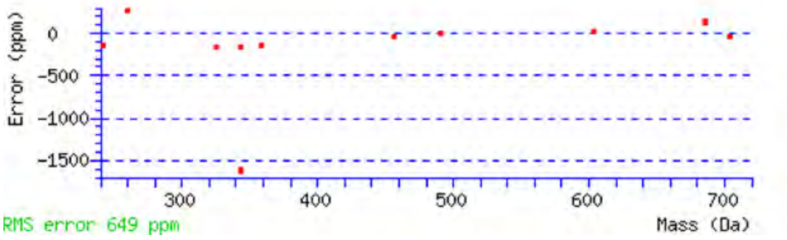

NCBI **BLAST** search of AGLTFPVGR
(Parameters: blastp, nr protein database, expect=20000, no filter, PAM30)
Other BLAST web gateways

All matches to this query

Score	Mr(calc)	Delta	Sequence
45.4	944.5444	-0.0005	AGLTFPVGR
17.0	944.5477	-0.0038	IKDVMRK
15.8	944.5443	-0.0004	NDLFPRK
15.8	944.5443	-0.0005	NDLFPRK
14.6	944.5403	0.0036	IKTREER
14.6	944.5403	0.0036	IKTREER
13.9	944.5484	-0.0045	KWFIGPK
12.3	944.5477	-0.0038	RAIEMALK
12.0	944.5403	0.0036	DGRKQSLK
10.9	944.5477	-0.0038	KIKCNAK

Mascot: http://www.matrixscience.com/

Mascot Search Results

Peptide View

MS/MS Fragmentation of **HLQLAIR**
Found in **H2A1_YEAST** in **S_cerevisiae_D**, sp|P04911|H2A1_YEAST Histone H2A.1 OS=Saccharomyces cerevisiae (strain ATCC 204508 / S288c) GN=HTA1 PE=1 SV=2

Match to Query 17771: 849.518228 from(425.766390,2+) intensity(133018.1600) scans(3492) rawscans(sn3492) rtinseconds(1529.9571) index(43751)
Title: 2722: Scan 3492 (rt=25.4993) [D:\MSData\All\VELOS23966.raw]
Data file D:\Data\MGF\530 Final H2A H2B yeast classical PTMs\mascot_daemon_merge.mgf

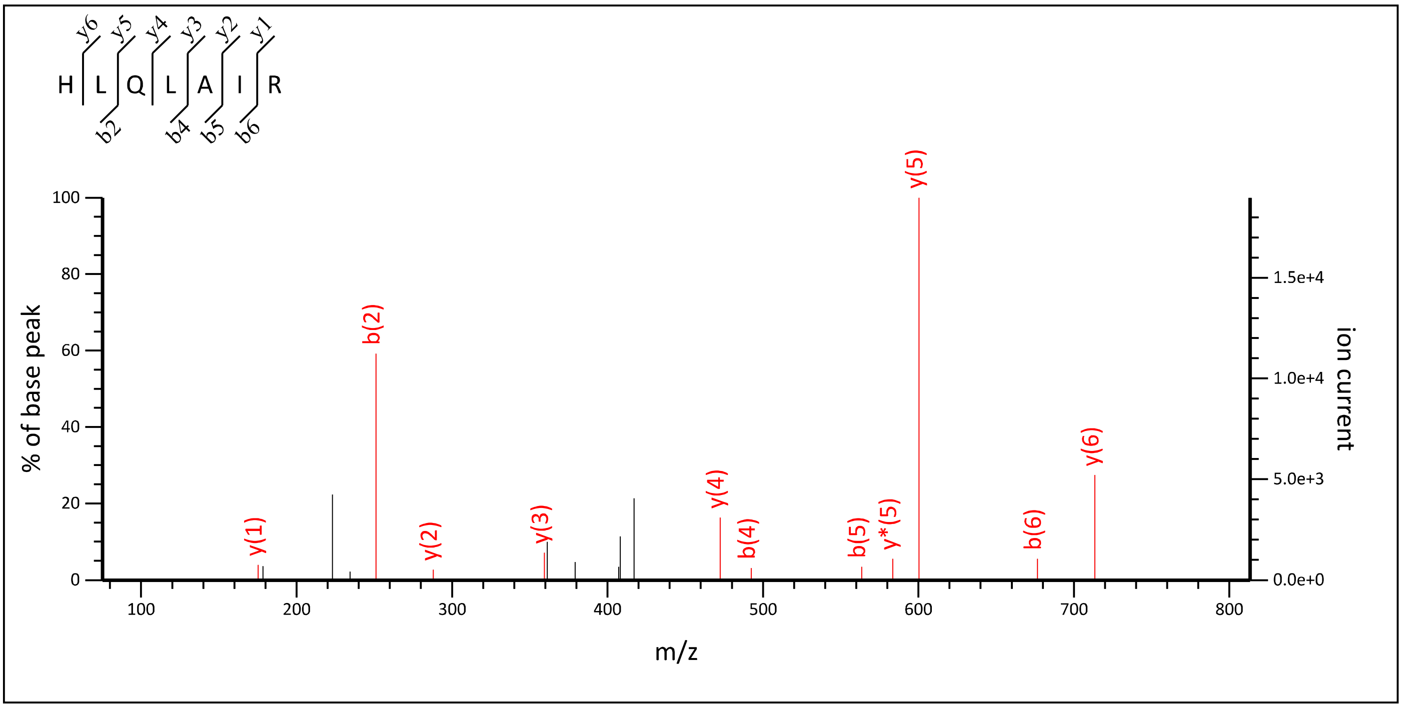

75.21 to 813.42

Label all possible matches ○ Label matches used for scoring ◉

Monoisotopic mass of neutral peptide Mr(calc): 849.5184
Fixed modifications: Carbamidomethyl (C) (apply to specified residues or termini only)
Ions Score: 61 **Expect:** 1.9e-05
Matches : 11/44 fragment ions using 15 most intense peaks (help)

#	b	b^{++}	b*	b^{*++}	Seq.	y	y^{++}	y*	y^{*++}	#
1	138.0662	69.5367			H					7
2	**251.1503**	126.0788			L	*713.4668*	357.2371	696.4403	348.7238	6
3	379.2088	190.1081	362.1823	181.5948	Q	*600.3828*	300.6950	583.3562	292.1817	5
4	**492.2929**	246.6501	475.2663	238.1368	L	*472.3242*	236.6657	455.2976	228.1525	4
5	**563.3300**	282.1686	546.3035	273.6554	A	*359.2401*	180.1237	342.2136	171.6104	3
6	**676.4141**	338.7107	659.3875	330.1974	I	*288.2030*	144.6051	271.1765	136.0919	2
7					R	*175.1190*	88.0631	158.0924	79.5498	1

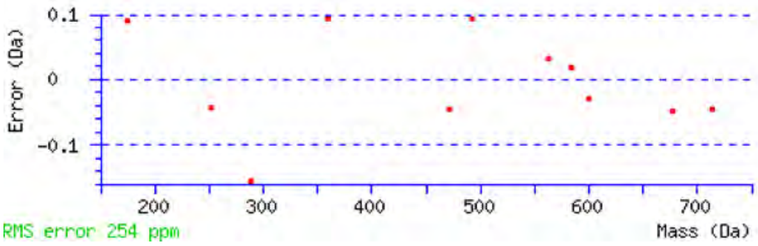

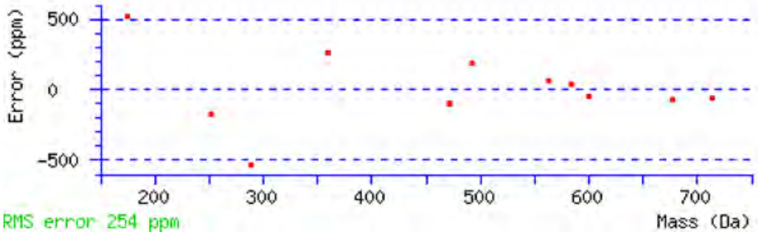

NCBI **BLAST** search of HLQLAIR
(Parameters: blastp, nr protein database, expect=20000, no filter, PAM30)
Other BLAST web gateways

All matches to this query

Score	Mr(calc)	Delta	Sequence
60.9	849.5184	-0.0002	HLQLAIR
30.4	849.5184	-0.0002	SYKRLR
23.3	849.5184	-0.0002	SYKRLR
17.9	849.5184	-0.0002	HLARKK
17.9	849.5185	-0.0002	HLIQGLR
17.9	849.5184	-0.0002	HLKAKR
17.7	849.5184	-0.0002	SYKRLR
16.1	849.5185	-0.0002	HLPVKSR
11.3	849.5184	-0.0002	IQHALLR
11.3	849.5184	-0.0002	KYSRIR

Mascot: http://www.matrixscience.com/

Mascot Search Results

Peptide View

MS/MS Fragmentation of **HLQLAIR**
Found in **H2A1_YEAST** in **S_cerevisiae_D**, sp|P04911|H2A1_YEAST Histone H2A.1 OS=Saccharomyces cerevisiae (strain ATCC 204508 / S288c) GN=HTA1 PE=1 SV=2

Match to Query 23351: 877.549588 from(439.782070,2+) intensity(36232.1130) scans(4122) rawscans(sn4122) rtinseconds(1641.3617) index(15328)
Title: 3259: Scan 4122 (rt=27.356) [D:\MSData\All\VELOS23962.raw]
Data file D:\Data\MGF\530 Final H2A H2B yeast classical PTMs\mascot_daemon_merge.mgf

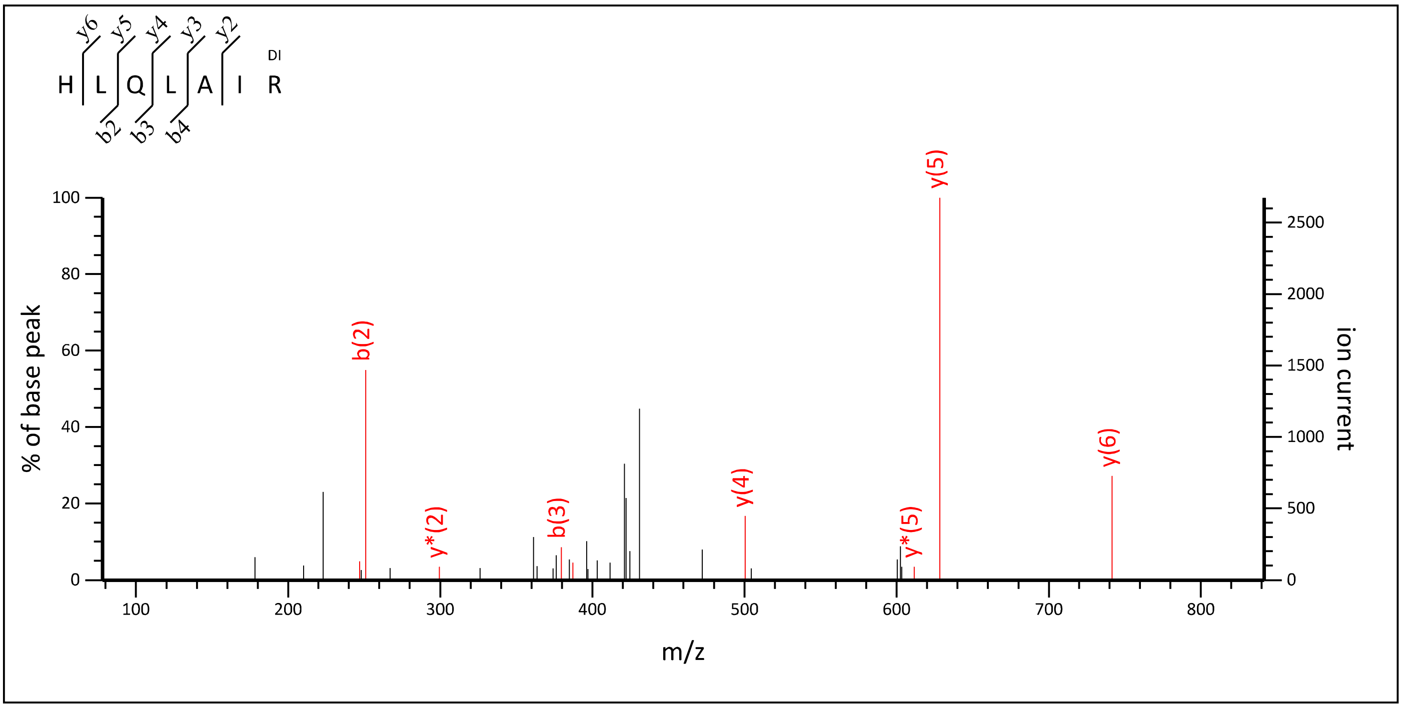

78.11 to 841.52

Label all possible matches Label matches used for scoring

Monoisotopic mass of neutral peptide Mr(calc): 877.5497
Fixed modifications: Carbamidomethyl (C) (apply to specified residues or termini only)
Variable modifications:
R7 : Dimethyl (R)
Ions Score: 21 **Expect:** 0.13 (help)

#	b	b++	b*	b*++	Seq.	y	y++	y*	y*++	#
1	138.0662	69.5367			H					7
2	**251.1503**	126.0788			L	**741.4981**	371.2527	724.4716	362.7394	6
3	**379.2088**	190.1081	362.1823	181.5948	Q	**628.4141**	314.7107	**611.3875**	306.1974	5
4	492.2929	**246.6501**	475.2663	238.1368	L	**500.3555**	**250.6814**	483.3289	242.1681	4
5	563.3300	282.1686	546.3035	273.6554	A	**387.2714**	194.1394	370.2449	185.6261	3
6	676.4141	338.7107	659.3875	330.1974	I	316.2343	158.6208	**299.2078**	150.1075	2
7					R	203.1503	102.0788	186.1237	93.5655	1

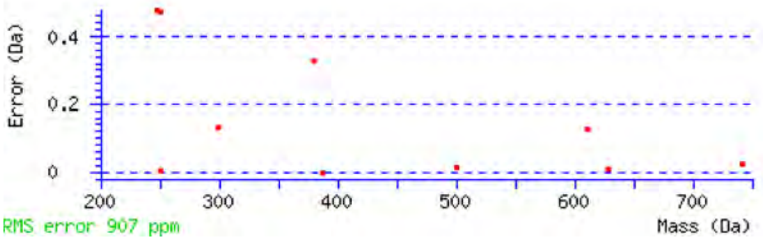

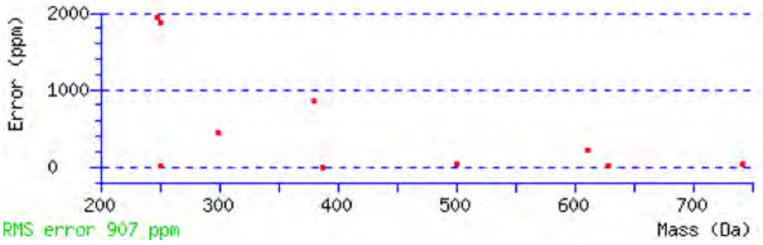

NCBI **BLAST** search of HLQLAIR
(Parameters: blastp, nr protein database, expect=20000, no filter, PAM30)
Other BLAST web gateways

All matches to this query

Score	Mr(calc)	Delta	Sequence
20.9	877.5497	-0.0002	HLQLAIR
10.7	877.5498	-0.0002	HLGLRVK
10.7	877.5497	-0.0001	HLKKIR
9.4	877.5497	-0.0001	SYKRLR
9.4	877.5497	-0.0002	HLAKVLR
9.4	877.5497	-0.0002	HLIKLGR
9.4	877.5498	-0.0002	HLPVKSR
9.4	877.5498	-0.0002	HLPVKSR
9.4	877.5498	-0.0002	HLPVKSR
2.4	877.5497	-0.0001	LHKRLK

Mascot: http://www.matrixscience.com/

MATRIX SCIENCE Mascot Search Results

Peptide View

MS/MS Fragmentation of **NDDELNKLLGNVTIAQGGVLPNIHQNLLPK**
Found in **H2A1_YEAST** in **S_cerevisiae_D**, sp|P04911|H2A1_YEAST Histone H2A.1 OS=Saccharomyces cerevisiae (strain ATCC 204508 / S288c) GN=HTA1 PE=1 SV=2

Match to Query 335832: 3278.761136 from(820.697560,4+) intensity(4808.7051) scans(15077) rawscans(sn15077) rtinseconds(4689.7181) index(24613)
Title: 12544: Scan 15077 (rt=78.162) [D:\MSData\All\VELOS23962.raw]
Data file D:\Data\MGF\530 Final H2A H2B yeast classical PTMs\mascot_daemon_merge.mgf

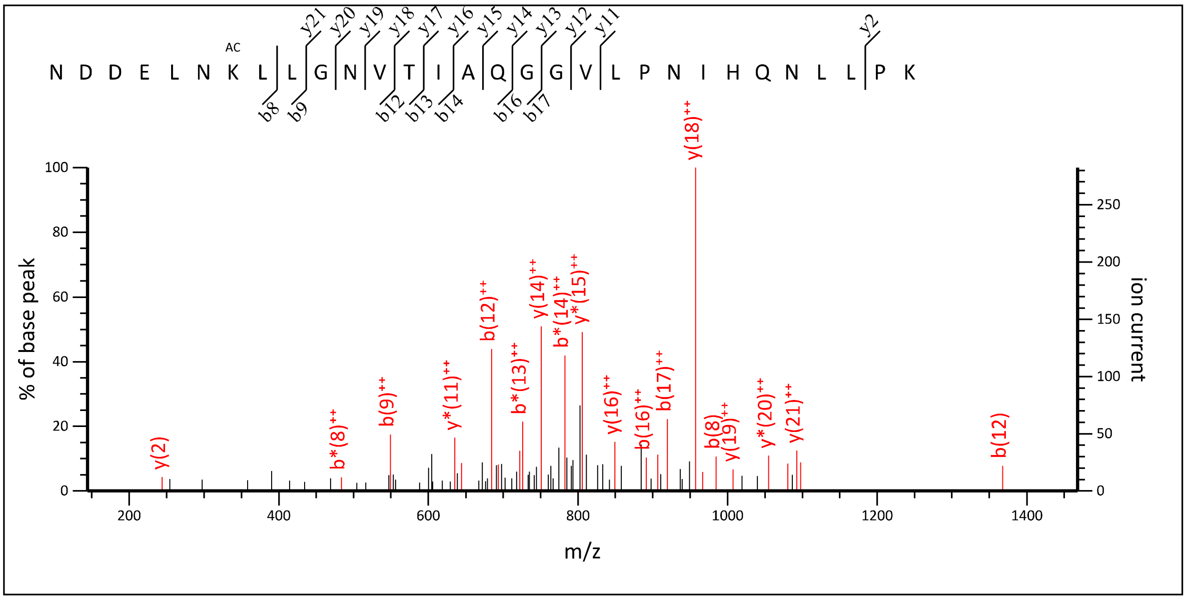

144.15 to 1467.7

Label all possible matches ○ Label matches used for scoring ◉

```
Monoisotopic mass of neutral peptide Mr(calc): 3278.7623
Fixed modifications: Carbamidomethyl (C) (apply to specified residues or termini only)
Variable modifications:
K7     : Acetyl (K)
Ions Score: 33  Expect: 0.026
Matches : 32/312 fragment ions using 47 most intense peaks   (help)
```

#	b	b++	b*	b*++	b0	b0++	Seq.	y	y++	y*	y*++	y0	y0++	#
1	115.0502	58.0287	98.0237	49.5155			N							30
2	230.0771	115.5422	213.0506	107.0289	212.0666	106.5369	D	3165.7266	1583.3670	3148.7001	1574.8537	3147.7161	1574.3617	29
3	345.1041	173.0557	328.0775	164.5424	327.0935	164.0504	D	3050.6997	1525.8535	3033.6731	1517.3402	3032.6891	1516.8482	28
4	474.1467	237.5770	457.1201	229.0637	456.1361	228.5717	E	2935.6728	1468.3400	2918.6462	1459.8267	2917.6622	1459.3347	27
5	587.2307	294.1190	570.2042	285.6057	569.2202	285.1137	L	2806.6302	1403.8187	2789.6036	1395.3054	2788.6196	1394.8134	26
6	701.2737	351.1405	684.2471	342.6272	683.2631	342.1352	N	2693.5461	1347.2767	2676.5195	1338.7634	2675.5355	1338.2714	25
7	871.3792	436.1932	854.3527	427.6800	853.3686	427.1880	K	2579.5032	1290.2552	2562.4766	1281.7419	2561.4926	1281.2499	24
8	**984.4633**	492.7353	967.4367	484.2220	966.4527	483.7300	L	2409.3976	1205.2025	2392.3711	1196.6892	2391.3871	1196.1972	23
9	**1097.5473**	**549.2773**	1080.5208	540.7640	1079.5368	540.2720	L	2296.3136	1148.6604	2279.2870	1140.1472	2278.3030	1139.6551	22
10	1154.5688	577.7880	1137.5422	569.2748	1136.5582	568.7828	G	2183.2295	***1092.1184***	2166.2030	1083.6051	2165.2189	1083.1131	21
11	1268.6117	**634.8095**	1251.5852	626.2962	1250.6012	625.8042	N	2126.2080	1063.6077	2109.1815	1055.0944	2108.1975	1054.6024	20
12	**1367.6801**	**684.3437**	1350.6536	675.8304	1349.6696	675.3384	V	2012.1651	***1006.5862***	1995.1386	998.0729	1994.1546	997.5809	19
13	1468.7278	734.8675	1451.7013	726.3543	1450.7172	725.8623	T	1913.0967	***957.0520***	1896.0702	948.5387	1895.0861	948.0467	18
14	1581.8119	791.4096	1564.7853	782.8963	1563.8013	782.4043	I	1812.0490	***906.5282***	1795.0225	898.0149			17
15	1652.8490	826.9281	1635.8224	818.4149	1634.8384	817.9229	A	1698.9650	***849.9861***	1681.9384	841.4728			16
16	1780.9076	**890.9574**	1763.8810	882.4441	1762.8970	881.9521	Q	1627.9279	814.4676	1610.9013	805.9543			15
17	1837.9290	**919.4682**	1820.9025	910.9549	1819.9185	910.4629	G	1499.8693	***750.4383***	1482.8427	741.9250			14
18	1894.9505	947.9789	1877.9239	939.4656	1876.9399	938.9736	G	1442.8478	***721.9275***	1425.8213	713.4143			13
19	1994.0189	997.5131	1976.9924	988.9998	1976.0083	988.5078	V	1385.8263	***693.4168***	1368.7998	684.9035			12
20	2107.1030	1054.0551	2090.0764	1045.5419	2089.0924	1045.0498	L	1286.7579	***643.8826***	1269.7314	635.3693			11
21	2204.1557	1102.5815	2187.1292	1094.0682	2186.1452	1093.5762	P	1173.6739	587.3406	1156.6473	578.8273			10

22	2318.1987	1159.6030	2301.1721	1151.0897	2300.1881	1150.5977	N	1076.6211	538.8142	1059.5946	530.3009			9
23	2431.2827	1216.1450	2414.2562	1207.6317	2413.2722	1207.1397	I	962.5782	481.7927	945.5516	473.2795			8
24	2568.3416	1284.6745	2551.3151	1276.1612	2550.3311	1275.6692	H	849.4941	425.2507	832.4676	416.7374			7
25	2696.4002	1348.7037	2679.3737	1340.1905	2678.3897	1339.6985	Q	712.4352	356.7212	695.4087	348.2080			6
26	2810.4431	1405.7252	2793.4166	1397.2119	2792.4326	1396.7199	N	584.3766	292.6919	567.3501	284.1787			5
27	2923.5272	1462.2672	2906.5007	1453.7540	2905.5166	1453.2620	L	470.3337	235.6705	453.3071	227.1572			4
28	3036.6113	1518.8093	3019.5847	1510.2960	3018.6007	1509.8040	L	357.2496	179.1285	340.2231	170.6152			3
29	3133.6640	1567.3357	3116.6375	1558.8224	3115.6535	1558.3304	P	244.1656	122.5864	227.1390	114.0731			2
30							K	147.1128	74.0600	130.0863	65.5468			1

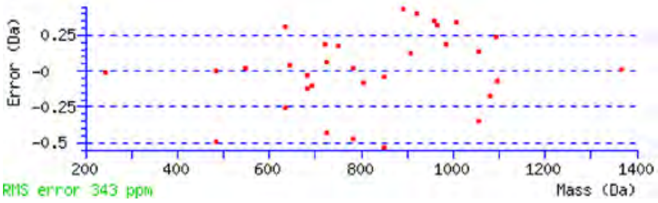

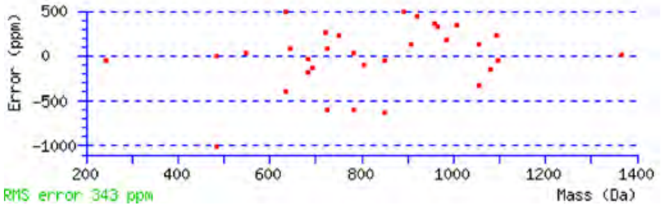

NCBI **BLAST** search of NDDELNKLLGNVTIAQGGVLPNIHQNLLPK
(Parameters: blastp, nr protein database, expect=20000, no filter, PAM30)
Other BLAST web gateways

All matches to this query

Score	Mr(calc)	Delta	Sequence
33.3	3278.7623	-0.0011	NDDELNKLLGNVTIAQGGVLPNIHQNLLPK
15.8	3278.7686	-0.0075	STNKSKMVNTGKNYILGGHNKVKNNSR
15.5	3278.7649	-0.0038	GKLVDLLSDEGIVNSAGDTLTVKKNYLYK
15.1	3278.7580	0.0031	EFLKGIGSKKNPVFHRVVKEENIYNK
15.1	3278.7721	-0.0110	DKISQKIINKEINLPDPNSNQGESTTKK
15.1	3278.7721	-0.0110	DKISQKIINKEINLPDPNSNQGESTTKK
15.1	3278.7721	-0.0110	DKISQKIINKEINLPDPNSNQGESTTKK
15.1	3278.7721	-0.0110	DKISQKIINKEINLPDPNSNQGESTTKK
15.0	3278.7516	0.0095	DSALVPAEALISLAKRRVAVARTTDGLKK
15.0	3278.7516	0.0095	DSALVPAEALISLAKRRVAVARTTDGLKK

Mascot: http://www.matrixscience.com/

MATRIX SCIENCE Mascot Search Results

Peptide View

MS/MS Fragmentation of **ATKASQEL**
Found in **H2A1_YEAST** in **S_cerevisiae_D**, sp|P04911|H2A1_YEAST Histone H2A.1 OS=Saccharomyces cerevisiae (strain ATCC 204508 / S288c) GN=HTA1 PE=1 SV=2

Match to Query 24757: 888.455068 from(445.234810,2+) intensity(67322.0390) scans(3024) rawscans(sn3024) rtinseconds(1271.5559) index(98128)
Title: 2380: Scan 3024 (rt=21.1926) [D:\MSData\All\VELOS23970.raw]
Data file D:\Data\MGF\530 Final H2A H2B yeast classical PTMs\mascot_daemon_merge.mgf

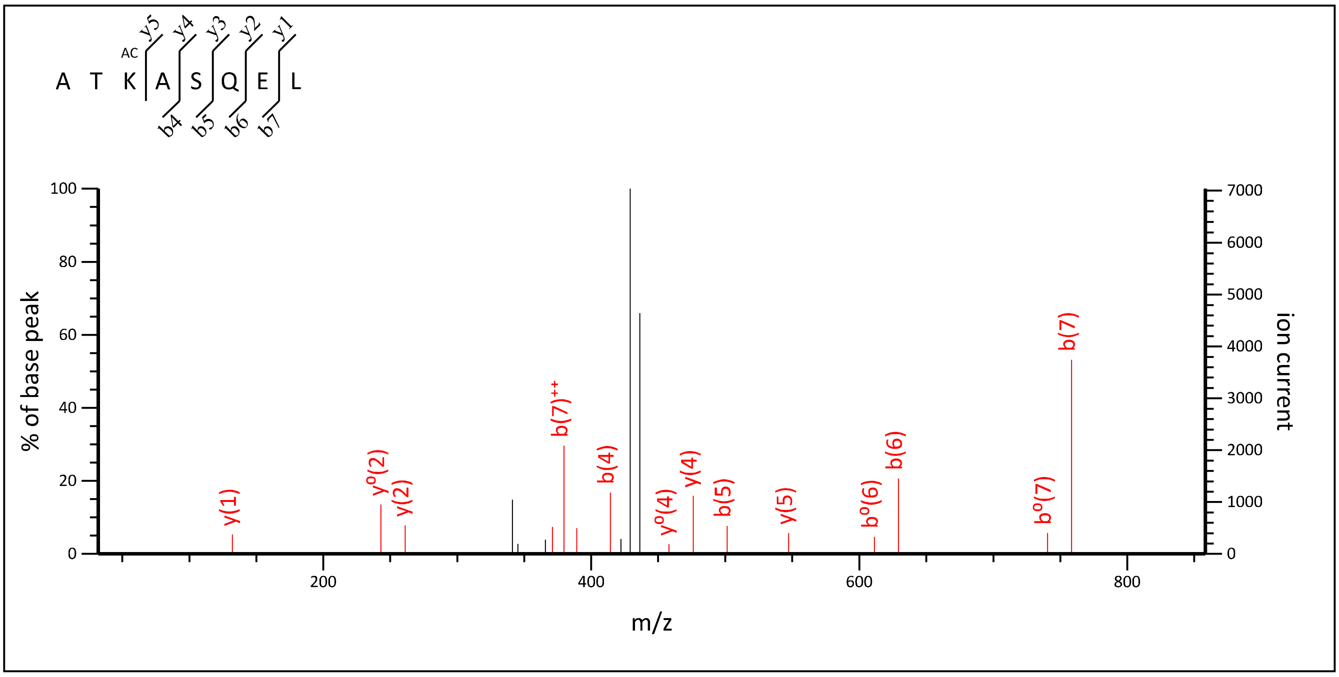

32.18 to 858.33

Label all possible matches ○ Label matches used for scoring ◉

Monoisotopic mass of neutral peptide Mr(calc): 888.4552
Fixed modifications: Carbamidomethyl (C) (apply to specified residues or termini only)
Variable modifications:
K3 : Acetyl (K)
Ions Score: 43 **Expect:** 0.00058
Matches : 18/72 fragment ions using 18 most intense peaks (help)

#	b	b^{++}	b*	b^{*++}	b^0	b^{0++}	Seq.	y	y^{++}	y*	y^{*++}	y^0	y^{0++}	#
1	72.0444	36.5258					A							8
2	173.0921	87.0497			155.0815	78.0444	T	818.4254	409.7164	801.3989	401.2031	800.4149	400.7111	7
3	343.1976	172.1024	326.1710	163.5892	325.1870	163.0972	K	717.3777	359.1925	700.3512	350.6792	699.3672	350.1872	6
4	**414.2347**	207.6210	397.2082	199.1077	396.2241	198.6157	A	***547.2722***	274.1397	530.2457	265.6265	529.2617	265.1345	5
5	**501.2667**	251.1370	484.2402	242.6237	483.2562	242.1317	S	***476.2351***	238.6212	459.2086	230.1079	458.2245	229.6159	4
6	**629.3253**	315.1663	612.2988	306.6530	611.3148	306.1610	Q	***389.2031***	195.1052	372.1765	186.5919	371.1925	186.0999	3
7	**758.3679**	379.6876	741.3414	371.1743	740.3573	370.6823	E	***261.1445***	131.0759			243.1339	122.0706	2
8							L	***132.1019***	66.5546					1

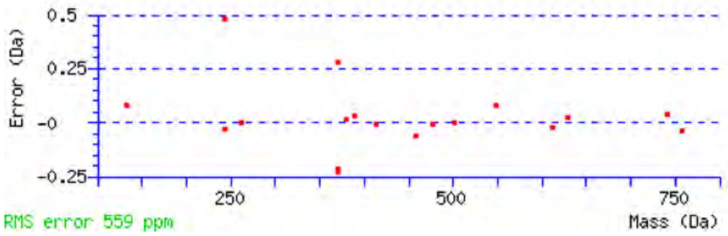

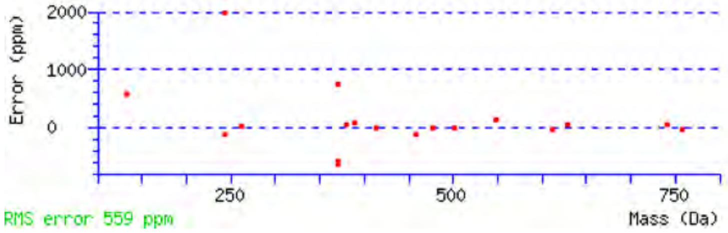

NCBI **BLAST** search of ATKASQEL
(Parameters: blastp, nr protein database, expect=20000, no filter, PAM30)
Other BLAST web gateways

All matches to this query

Score	Mr(calc)	Delta	Sequence
42.6	888.4552	-0.0002	ATKASQEL
42.6	888.4552	-0.0002	TAKASQEL
7.6	888.4552	-0.0002	IGDQSISK
5.5	888.4552	-0.0001	ENKSLSK
5.5	888.4552	-0.0002	TADTSKPK
5.1	888.4561	-0.0010	MPVLGMNK
4.2	888.4552	-0.0001	EEKSINK
4.2	888.4552	-0.0001	EKEAENK
4.2	888.4552	-0.0002	SLDLAENK
2.7	888.4552	-0.0002	NVEADSIK

Mascot: http://www.matrixscience.com/

MATRIX SCIENCE Mascot Search Results

Peptide View

MS/MS Fragmentation of **ATKASQEL**
Found in **H2A1_YEAST** in **S_cerevisiae_D**, sp|P04911|H2A1_YEAST Histone H2A.1 OS=Saccharomyces cerevisiae (strain ATCC 204508 / S288c) GN=HTA1 PE=1 SV=2

Match to Query 42313: 968.421508 from(485.218030,2+) intensity(33457.2150) scans(2082) rawscans(sn2082) rtinseconds(1089.4626) index(84057)
Title: 1557: Scan 2082 (rt=18.1577) [D:\MSData\All\VELOS23968.raw]
Data file D:\Data\MGF\530 Final H2A H2B yeast classical PTMs\mascot_daemon_merge.mgf

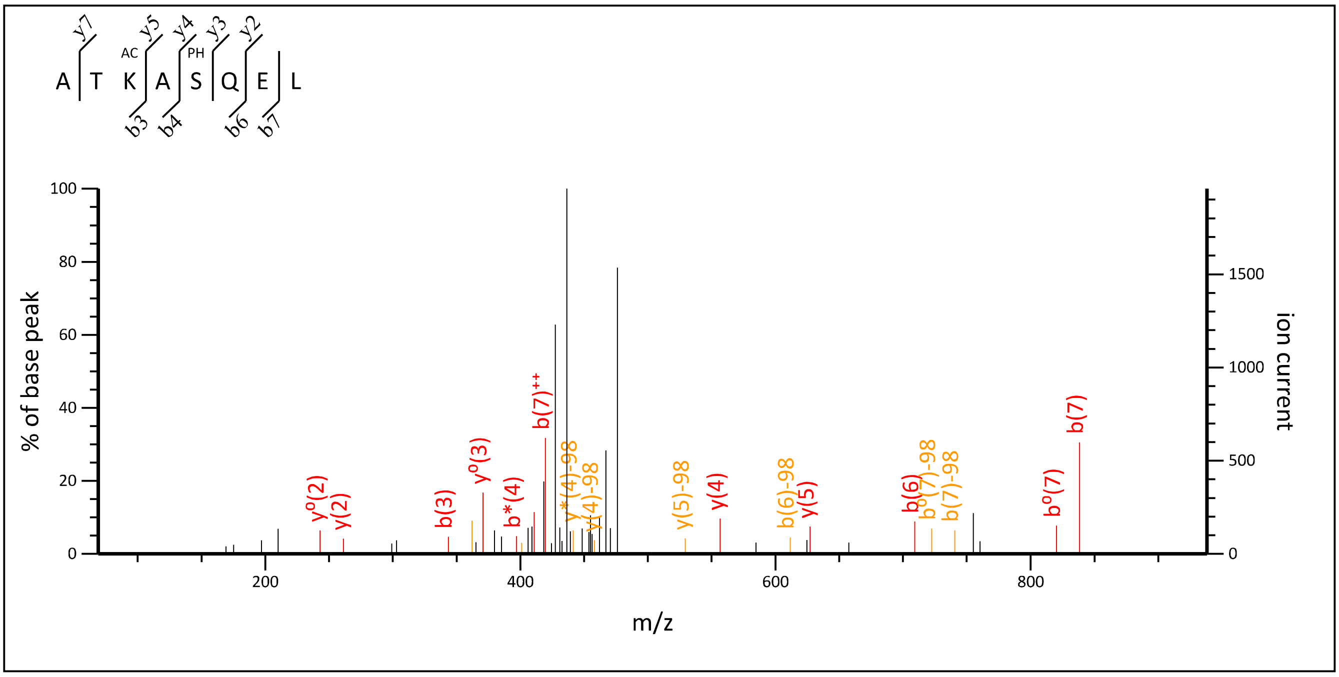

69.13 to 938.29

Label all possible matches Label matches used for scoring

Monoisotopic mass of neutral peptide Mr(calc): 968.4216
Fixed modifications: Carbamidomethyl (C) (apply to specified residues or termini only)
Variable modifications:
K3 : Acetyl (K)
S5 : Phospho (ST), with neutral losses 0.0000(shown in table), 97.9769
Ions Score: 21 **Expect:** 0.088 (help)

#	b	b^{++}	b*	b^{*++}	b^0	b^{0++}	Seq.	y	y^{++}	y*	y^{*++}	y^0	y^{0++}	#
1	72.0444	36.5258					A							8
2	173.0921	87.0497			155.0815	78.0444	T	898.3918	449.6995	881.3652	**441.1862**	880.3812	440.6942	7
3	**343.1976**	172.1024	326.1710	163.5892	325.1870	163.0972	K	797.3441	399.1757	780.3175	390.6624	779.3335	390.1704	6
4	414.2347	207.6210	**397.2082**	199.1077	396.2241	198.6157	A	**627.2385**	314.1229	610.2120	305.6096	609.2280	305.1176	5
5	581.2331	291.1202	564.2065	282.6069	563.2225	282.1149	S	**556.2014**	278.6044	539.1749	270.0911	538.1909	269.5991	4
6	**709.2916**	355.1495	692.2651	346.6362	691.2811	346.1442	Q	389.2031	195.1052	372.1765	186.5919	**371.1925**	186.0999	3
7	**838.3342**	**419.6708**	821.3077	**411.1575**	**820.3237**	**410.6655**	E	**261.1445**	131.0759			**243.1339**	122.0706	2
8							L	132.1019	66.5546					1

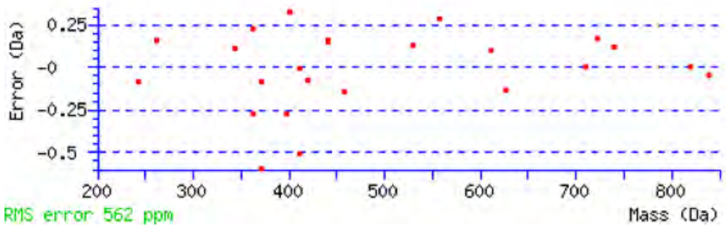

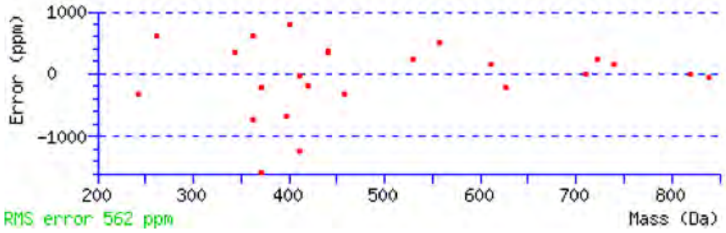

NCBI **BLAST** search of ATKASQEL
(Parameters: blastp, nr protein database, expect=20000, no filter, PAM30)
Other BLAST web gateways

All matches to this query

Score	Mr(calc)	Delta	Sequence
21.3	968.4216	-0.0000	ATKASQEL
21.3	968.4216	-0.0000	TAKASQEL
9.4	968.4216	-0.0001	NDLVSATK
8.0	968.4216	-0.0001	DVQDIGSK
7.8	968.4216	-0.0000	ATGLAAESK
7.4	968.4216	-0.0001	NVEADSIK
6.2	968.4216	-0.0000	ATKASQEL
6.2	968.4216	-0.0000	TAKASQEL
5.8	968.4191	0.0024	MPHSTFK
4.4	968.4216	-0.0000	KEIDSNK

**Mascot:** http://www.matrixscience.com/

MATRIX SCIENCE Mascot Search Results

Peptide View

MS/MS Fragmentation of **SAKAEKKPASK**
Found in **H2B1_YEAST** in **S_cerevisiae_D**, sp|P02293|H2B1_YEAST Histone H2B.1 OS=Saccharomyces cerevisiae (strain ATCC 204508 / S288c) GN=HTB1 PE=1 SV=2

Match to Query 100881: 1269.692188 from(635.853370,2+) intensity(69641.7970) scans(1517) rawscans(sn1517) rtinseconds(832.1771) index(258719)
Title: 1200: Scan 1517 (rt=13.8696) [D:\MSData\All\VELOS23664.raw]
Data file D:\Data\MGF\530 Final H2A H2B yeast classical PTMs\mascot_daemon_merge.mgf

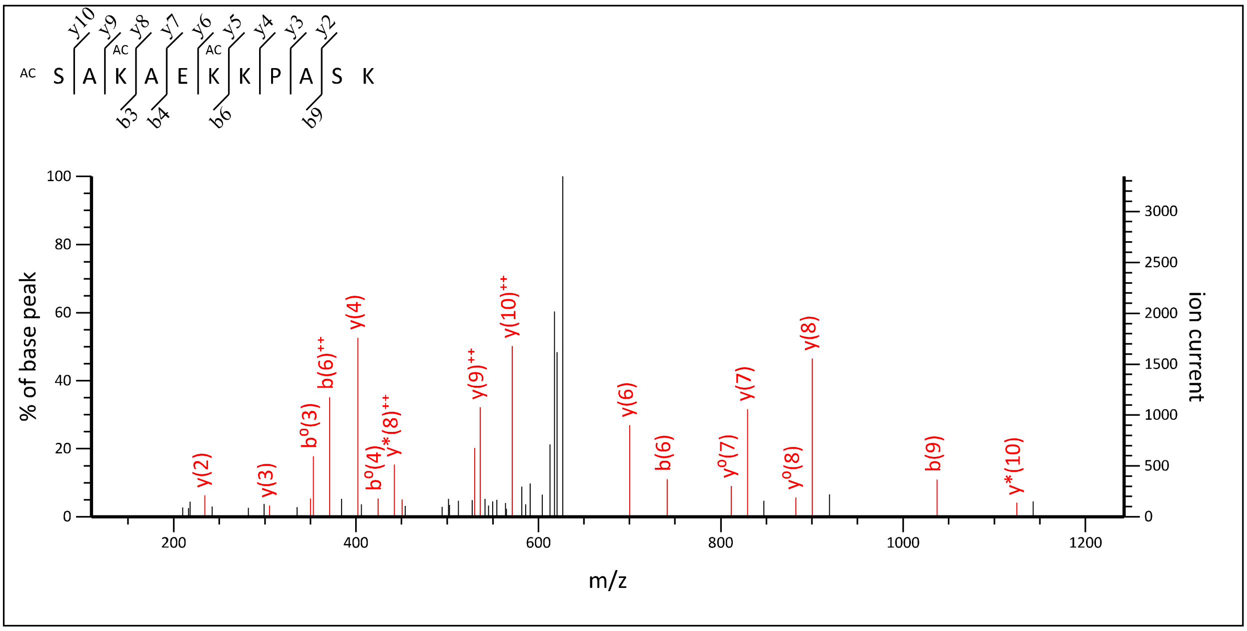

109.94 to 1242.58

Label all possible matches ○ Label matches used for scoring ◉

Monoisotopic mass of neutral peptide Mr(calc): 1269.6928
Fixed modifications: Carbamidomethyl (C) (apply to specified residues or termini only)
Variable modifications:
N-term : Acetyl (Protein N-term)
K3 : Acetyl (K)
K6 : Acetyl (K)
Ions Score: 54 **Expect:** 0.00025
Matches : 25/114 fragment ions using 29 most intense peaks (help)

#	b	b^{++}	b*	b*$^{++}$	b^{0}	b^{0++}	Seq.	y	y^{++}	y*	y*$^{++}$	y^{0}	y^{0++}	#
1	130.0499	65.5286			112.0393	56.5233	S							11
2	201.0870	101.0471			183.0764	92.0418	A	1141.6576	**571.3324**	1124.6310	562.8191	1123.6470	562.3271	10
3	***371.1925***	186.0999	354.1660	177.5866	353.1819	177.0946	K	1070.6204	**535.8139**	1053.5939	527.3006	1052.6099	526.8086	9
4	***442.2296***	221.6185	425.2031	213.1052	424.2191	212.6132	A	***900.5149***	**450.7611**	883.4884	442.2478	882.5043	441.7558	8
5	***571.2722***	286.1397	554.2457	277.6265	553.2617	277.1345	E	***829.4778***	415.2425	812.4512	406.7293	811.4672	406.2373	7
6	***741.3777***	371.1925	724.3512	362.6792	723.3672	362.1872	K	***700.4352***	**350.7212**	683.4087	342.2080	682.4246	341.7160	6
7	869.4727	435.2400	852.4462	426.7267	851.4621	426.2347	K	***530.3297***	265.6685	513.3031	257.1552	512.3191	256.6632	5
8	966.5255	483.7664	949.4989	475.2531	948.5149	474.7611	P	***402.2347***	201.6210	385.2082	193.1077	384.2241	192.6157	4
9	***1037.5626***	519.2849	1020.5360	510.7717	1019.5520	510.2796	A	***305.1819***	153.0946	288.1554	144.5813	287.1714	144.0893	3
10	***1124.5946***	562.8009	1107.5681	554.2877	1106.5841	553.7957	S	***234.1448***	117.5761	217.1183	109.0628	216.1343	108.5708	2
11							K	147.1128	74.0600	130.0863	65.5468			1

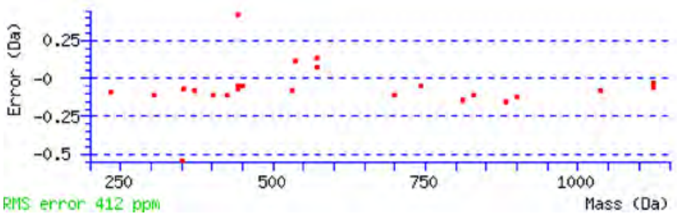

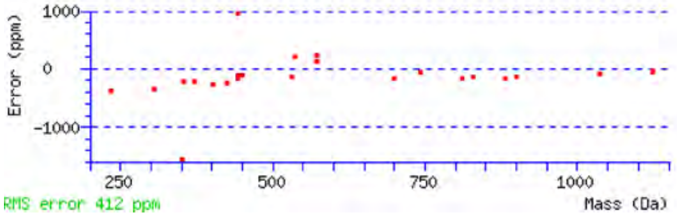

NCBI **BLAST** search of SAKAEKKPASK
(Parameters: blastp, nr protein database, expect=20000, no filter, PAM30)
Other BLAST web gateways

All matches to this query

Score	Mr(calc)	Delta	Sequence	Site Analysis
53.8	1269.6928	-0.0006	SAKAEKKPASK	Acetyl N-term, K3, K6 96.32%
39.6	1269.6928	-0.0006	SAKAEKKPASK	Acetyl N-term, K3, K7 3.64%
16.3	1269.6928	-0.0006	SAKAEKKPASK	Acetyl N-term, K3, K11 0.02%
14.5	1269.6945	-0.0023	TIKSELSKSK	
12.8	1269.6928	-0.0006	EKAEAAKAKAK	
12.7	1269.6929	-0.0007	QLEDIIVQNGK	
10.9	1269.6958	-0.0036	ERKSLFRAK	
10.9	1269.6958	-0.0036	ERKSLFRAK	
10.9	1269.6958	-0.0036	ERKSLFRAK	
10.0	1269.6880	0.0042	KACKNSTKK	

Mascot: http://www.matrixscience.com/

Mascot Search Results

Peptide View

MS/MS Fragmentation of **SAKAEKKPASK**
Found in **H2B1_YEAST** in **S_cerevisiae_D**, sp|P02293|H2B1_YEAST Histone H2B.1 OS=Saccharomyces cerevisiae (strain ATCC 204508 / S288c) GN=HTB1 PE=1 SV=2

Match to Query 100872: 1269.691408 from(635.852980,2+) intensity(17760.9200) scans(1423) rawscans(sn1423) rtinseconds(814.7078) index(156851)
Title: 1083: Scan 1423 (rt=13.5785) [D:\MSData\All\VELOS23670.raw]
Data file D:\Data\MGF\530 Final H2A H2B yeast classical PTMs\mascot_daemon_merge.mgf

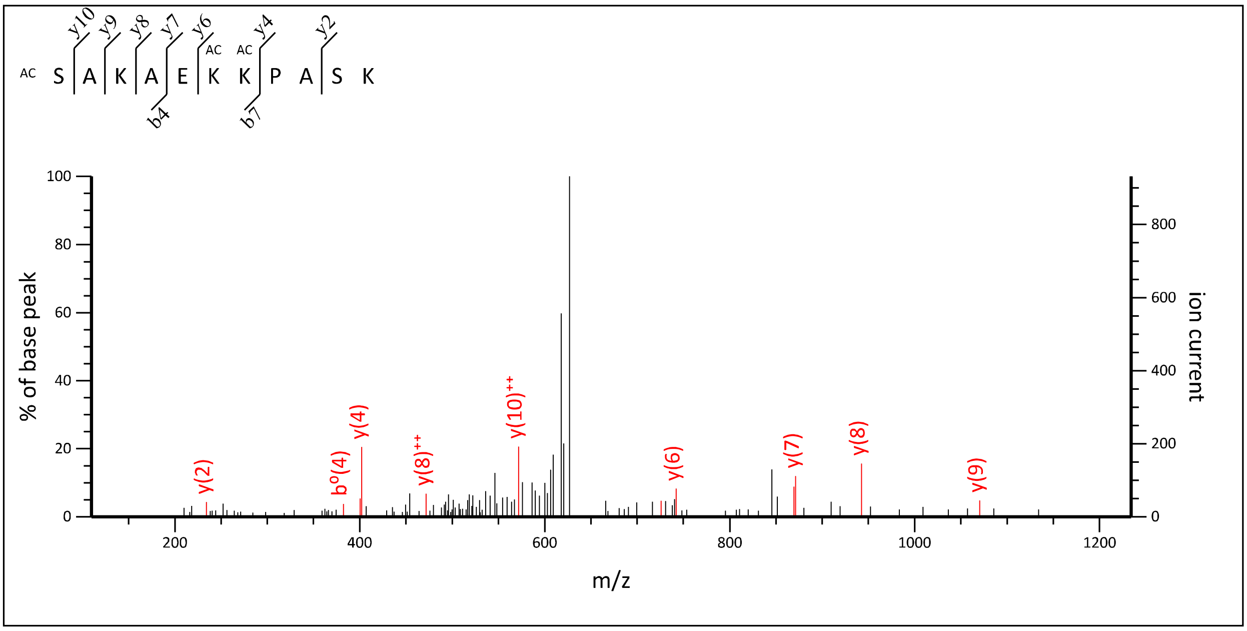

109.93 to 1234.06

Label all possible matches ○ Label matches used for scoring ◉

```
Monoisotopic mass of neutral peptide Mr(calc): 1269.6928
Fixed modifications: Carbamidomethyl (C) (apply to specified residues or termini only)
Variable modifications:
N-term : Acetyl (Protein N-term)
K6     : Acetyl (K)
K7     : Acetyl (K)
Ions Score: 31  Expect: 0.086
Matches : 12/114 fragment ions using 28 most intense peaks   (help)
```

#	b	b^{++}	b*	b^{*++}	b^0	b^{0++}	Seq.	y	y^{++}	y*	y^{*++}	y^0	y^{0++}	#
1	130.0499	65.5286			112.0393	56.5233	S							11
2	201.0870	101.0471			183.0764	92.0418	A	1141.6576	**571.3324**	1124.6310	562.8191	1123.6470	562.3271	10
3	329.1819	165.0946	312.1554	156.5813	311.1714	156.0893	K	***1070.6204***	535.8139	1053.5939	527.3006	1052.6099	526.8086	9
4	**400.2191**	200.6132	383.1925	192.0999	382.2085	191.6079	A	***942.5255***	**471.7664**	925.4989	463.2531	924.5149	462.7611	8
5	529.2617	265.1345	512.2351	256.6212	511.2511	256.1292	E	***871.4884***	436.2478	854.4618	427.7345	853.4778	427.2425	7
6	699.3672	350.1872	682.3406	341.6740	681.3566	341.1819	K	***742.4458***	371.7265	725.4192	363.2132	724.4352	362.7212	6
7	**869.4727**	435.2400	852.4462	426.7267	851.4621	426.2347	K	572.3402	286.6738	555.3137	278.1605	554.3297	277.6685	5
8	966.5255	483.7664	949.4989	475.2531	948.5149	474.7611	P	***402.2347***	201.6210	385.2082	193.1077	384.2241	192.6157	4
9	1037.5626	519.2849	1020.5360	510.7717	1019.5520	510.2796	A	305.1819	153.0946	288.1554	144.5813	287.1714	144.0893	3
10	1124.5946	562.8009	1107.5681	554.2877	1106.5841	553.7957	S	***234.1448***	117.5761	217.1183	109.0628	216.1343	108.5708	2
11							K	147.1128	74.0600	130.0863	65.5468			1

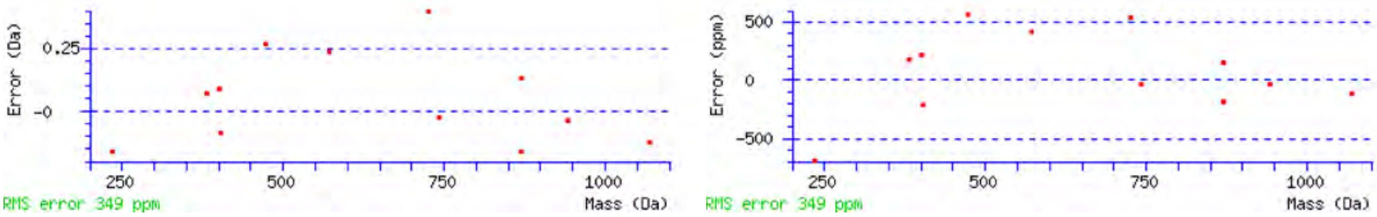

NCBI **BLAST** search of SAKAEKKPASK
(Parameters: blastp, nr protein database, expect=20000, no filter, PAM30)
Other BLAST web gateways

All matches to this query

Score	Mr(calc)	Delta	Sequence
30.8	1269.6928	-0.0014	SAKAEKKPASK
18.3	1269.6928	-0.0014	SAKAEKKPASK
12.1	1269.6928	-0.0014	SAKAEKKPASK
10.7	1269.6928	-0.0014	SAKAEKKPASK
5.2	1269.6928	-0.0014	AEKNKTENPK
5.1	1269.6928	-0.0014	AEKNKTENPK
5.0	1269.6928	-0.0014	SAKAEKKPASK
4.1	1269.6928	-0.0014	AEKNKTENPK
4.0	1269.6928	-0.0014	AEKNKTENPK
4.0	1269.6928	-0.0014	SAKAEKKPASK

Mascot: http://www.matrixscience.com/

MATRIX SCIENCE Mascot Search Results

Peptide View

MS/MS Fragmentation of **SSAAEKKPASK**
Found in **H2B2_YEAST** in **S_cerevisiae_D**, sp|P02294|H2B2_YEAST Histone H2B.2 OS=Saccharomyces cerevisiae (strain ATCC 204508 / S288c) GN=HTB2 PE=1 SV=2

Match to Query 91952: 1228.626128 from(615.320340,2+) intensity(18453.7660) scans(2442) rawscans(sn2442) rtinseconds(1068.117) index(319253)
Title: 1970: Scan 2442 (rt=17.802) [D:\MSData\All\VELOS23982.raw]
Data file D:\Data\MGF\530 Final H2A H2B yeast classical PTMs\mascot_daemon_merge.mgf

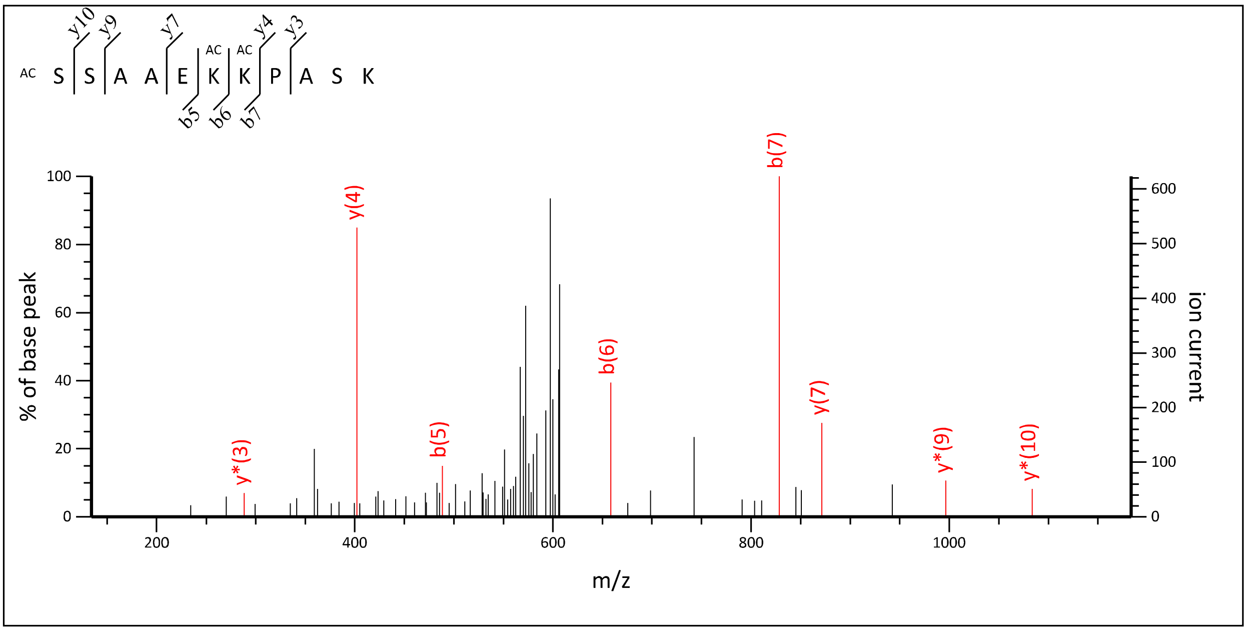

134.22 to 1183.53

Label all possible matches ○ Label matches used for scoring ◉

```
Monoisotopic mass of neutral peptide Mr(calc): 1228.6299
Fixed modifications: Carbamidomethyl (C) (apply to specified residues or termini only)
Variable modifications:
N-term : Acetyl (Protein N-term)
K6     : Acetyl (K)
K7     : Acetyl (K)
Ions Score: 59  Expect: 8.6e-05
Matches : 11/108 fragment ions using 9 most intense peaks   (help)
```

#	b	b^{++}	b*	b^{*++}	b^0	b^{0++}	Seq.	y	y^{++}	y*	y^{*++}	y^0	y^{0++}	#
1	130.0499	65.5286			112.0393	56.5233	S							11
2	217.0819	109.0446			199.0713	100.0393	S	1100.5946	550.8009	1083.5681	542.2877	1082.5841	541.7957	10
3	***288.1190***	144.5631			270.1084	135.5579	A	1013.5626	507.2849	996.5360	498.7717	995.5520	498.2796	9
4	359.1561	180.0817			341.1456	171.0764	A	942.5255	471.7664	925.4989	463.2531	924.5149	462.7611	8
5	***488.1987***	244.6030			470.1882	235.5977	E	**871.4884**	436.2478	854.4618	427.7345	853.4778	427.2425	7
6	***658.3042***	329.6558	641.2777	321.1425	640.2937	320.6505	K	742.4458	371.7265	725.4192	363.2132	724.4352	362.7212	6
7	***828.4098***	414.7085	811.3832	406.1953	810.3992	405.7032	K	572.3402	286.6738	555.3137	278.1605	554.3297	277.6685	5
8	925.4625	463.2349	908.4360	454.7216	907.4520	454.2296	P	**402.2347**	201.6210	385.2082	193.1077	384.2241	192.6157	4
9	***996.4997***	498.7535	979.4731	490.2402	978.4891	489.7482	A	305.1819	153.0946	288.1554	144.5813	287.1714	144.0893	3
10	***1083.5317***	542.2695	1066.5051	533.7562	1065.5211	533.2642	S	234.1448	117.5761	217.1183	109.0628	216.1343	108.5708	2
11							K	147.1128	74.0600	130.0863	65.5468			1

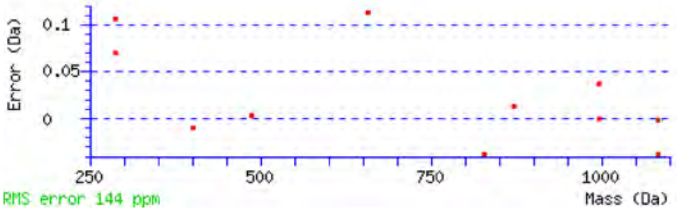

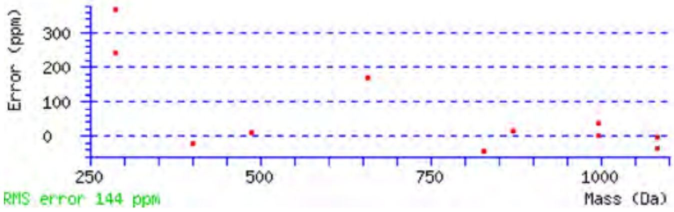

NCBI **BLAST** search of SSAAEKKPASK
(Parameters: blastp, nr protein database, expect=20000, no filter, PAM30)
Other BLAST web gateways

All matches to this query

Score	Mr(calc)	Delta	Sequence	Site Analysis
59.3	1228.6299	-0.0037	SSAAEKKPASK	Acetyl N-term, K6, K7 99.98%
20.2	1228.6299	-0.0037	SSAAEKKPASK	Acetyl N-term, K6, K11 0.01%
18.8	1228.6216	0.0045	SAYRKLSVK	
13.5	1228.6233	0.0028	ISSPLAKAVK	
11.8	1228.6299	-0.0037	SSAAEKKPASK	Acetyl N-term, K7, K11 0.00%
11.3	1228.6299	-0.0038	DDNGKEKKK	
11.3	1228.6299	-0.0038	DDNGKEKKK	
11.3	1228.6299	-0.0038	DDNGKEKKK	
11.1	1228.6308	-0.0046	QMKAPKMPDK	
10.9	1228.6217	0.0045	RDEKVFSAK	

Mascot: http://www.matrixscience.com/

MATRIX SCIENCE Mascot Search Results

Peptide View

MS/MS Fragmentation of **AEKKPASKAPAEKKPAAK**
Found in **H2B1_YEAST** in **S_cerevisiae_D**, sp|P02293|H2B1_YEAST Histone H2B.1 OS=Saccharomyces cerevisiae (strain ATCC 204508 / S288c) GN=HTB1 PE=1 SV=2

Match to Query 228300: 1975.109502 from(659.377110,3+) intensity(22931.4470) scans(897) rawscans(sn897) rtinseconds(650.1091) index(200073)
Title: 668: Scan 897 (rt=10.8352) [D:\MSData\All\VELOS23958.raw]
Data file D:\Data\MGF\530 Final H2A H2B yeast classical PTMs\mascot_daemon_merge.mgf

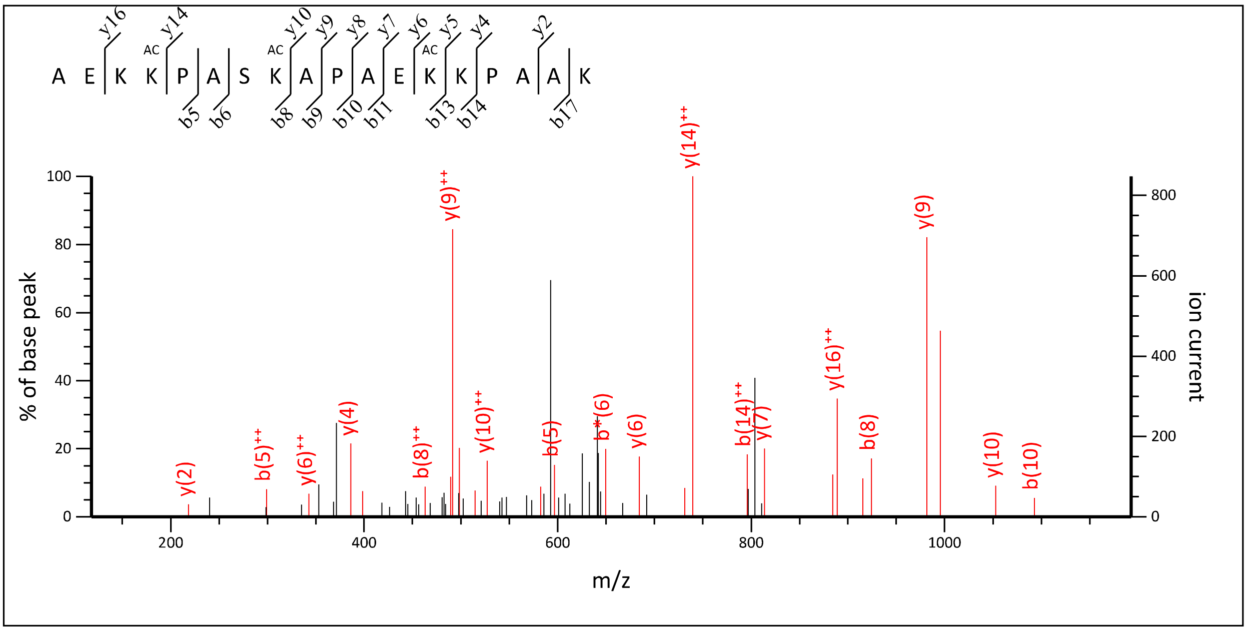

118.17 to 1192.7

Label all possible matches ○ Label matches used for scoring ◉

```
Monoisotopic mass of neutral peptide Mr(calc): 1975.1101
Fixed modifications: Carbamidomethyl (C) (apply to specified residues or termini only)
Variable modifications:
K4     : Acetyl (K)
K8     : Acetyl (K)
K13    : Acetyl (K)
Ions Score: 60  Expect: 0.0003
Matches : 31/186 fragment ions using 37 most intense peaks   (help)
```

#	b	b^{++}	b*	b^{*++}	b^0	b^{0++}	Seq.	y	y^{++}	y*	y^{*++}	y^0	y^{0++}	#
1	72.0444	36.5258					A							18
2	201.0870	101.0471			183.0764	92.0418	E	1905.0804	953.0438	1888.0538	944.5306	1887.0698	944.0386	17
3	329.1819	165.0946	312.1554	156.5813	311.1714	156.0893	K	1776.0378	**888.5225**	1759.0112	880.0093	1758.0272	879.5173	16
4	499.2875	250.1474	482.2609	241.6341	481.2769	241.1421	K	1647.9428	824.4751	1630.9163	815.9618	1629.9323	815.4698	15
5	**596.3402**	***298.6738***	579.3137	290.1605	578.3297	289.6685	P	1477.8373	**739.4223**	1460.8108	730.9090	1459.8267	730.4170	14
6	667.3774	334.1923	650.3508	325.6790	649.3668	325.1870	A	1380.7845	690.8959	1363.7580	682.3826	1362.7740	681.8906	13
7	754.4094	377.7083	737.3828	369.1951	736.3988	368.7030	S	1309.7474	655.3774	1292.7209	646.8641	1291.7369	646.3721	12
8	**924.5149**	***462.7611***	907.4884	454.2478	906.5043	453.7558	K	1222.7154	611.8613	1205.6889	603.3481	1204.7048	602.8561	11
9	**995.5520**	***498.2796***	978.5255	489.7664	977.5415	489.2744	A	***1052.6099***	**526.8086**	1035.5833	518.2953	1034.5993	517.8033	10
10	**1092.6048**	546.8060	1075.5782	538.2928	1074.5942	537.8007	P	***981.5728***	**491.2900**	964.5462	482.7767	963.5622	482.2847	9
11	1163.6419	***582.3246***	1146.6154	573.8113	1145.6313	573.3193	A	***884.5200***	442.7636	867.4934	434.2504	866.5094	433.7584	8
12	1292.6845	646.8459	1275.6579	638.3326	1274.6739	637.8406	E	***813.4829***	407.2451	796.4563	398.7318	795.4723	398.2398	7
13	1462.7900	***731.8986***	1445.7635	723.3854	1444.7795	722.8934	K	***684.4403***	**342.7238**	667.4137	334.2105			6
14	1590.8850	***795.9461***	1573.8584	787.4329	1572.8744	786.9408	K	***514.3348***	257.6710	497.3082	249.1577			5
15	1687.9377	844.4725	1670.9112	835.9592	1669.9272	835.4672	P	***386.2398***	193.6235	369.2132	185.1103			4
16	1758.9749	879.9911	1741.9483	871.4778	1740.9643	870.9858	A	289.1870	145.0972	272.1605	136.5839			3
17	1830.0120	***915.5096***	1812.9854	906.9964	1812.0014	906.5043	A	***218.1499***	109.5786	201.1234	101.0653			2

18							K	147.1128	74.0600	130.0863	65.5468			1

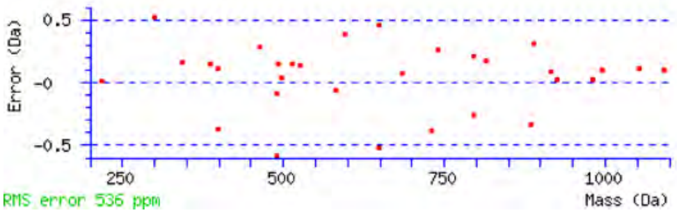

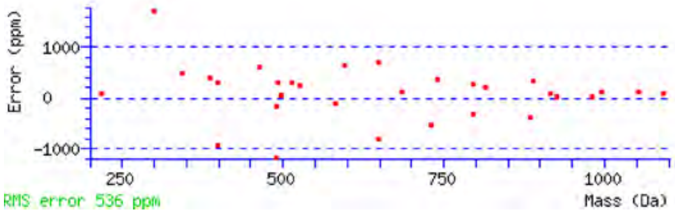

NCBI **BLAST** search of AEKKPASKAPAEKKPAAK
(Parameters: blastp, nr protein database, expect=20000, no filter, PAM30)
Other BLAST web gateways

All matches to this query

Score	Mr(calc)	Delta	Sequence	Site Analysis
60.3	1975.1101	-0.0006	AEKKPASKAPAEKKPAAK	Acetyl K4, K8, K13 99.89%
29.0	1975.1003	0.0092	ERGQVKKVANKFNGFK	
27.2	1975.1101	-0.0006	AEKKPASKAPAEKKPAAK	Acetyl K3, K4, K18 0.05%
23.4	1975.1091	0.0004	NLKRRTGGVKNNASLK	
23.4	1975.1091	0.0004	NLKRRTGGVKNNASLK	
20.9	1975.1003	0.0092	ERGQVKKVANKFNGFK	
20.7	1975.1003	0.0092	ERGQVKKVANKFNGFK	
18.5	1975.1118	-0.0023	DIVSKNNAEKLILAKK	
18.5	1975.1118	-0.0023	DIVSKNNAEKLILAKK	
13.9	1975.1003	0.0092	ERGQVKKVANKFNGFK	

Mascot: http://www.matrixscience.com/

MATRIX SCIENCE Mascot Search Results

Peptide View

MS/MS Fragmentation of **KPASKAPAEKKPAAK**
Found in **H2B2_YEAST** in **S_cerevisiae_D**, sp|P02294|H2B2_YEAST Histone H2B.2 OS=Saccharomyces cerevisiae (strain ATCC 204508 / S288c) GN=HTB2 PE=1 SV=2

Match to Query 178665: 1684.889022 from(562.636950,3+) intensity(60246.8500) scans(988-1002) rawscans(sn988:sn1002) rtinseconds(709.4411-713.9901) index(54619)
Title: 714: Sum of 2 scans in range 988 (rt=11.824) to 1002 (rt=11.8998) [D:\MSData\All\VELOS23666.raw]
Data file D:\Data\MGF\530 Final H2A H2B yeast classical PTMs\mascot_daemon_merge.mgf

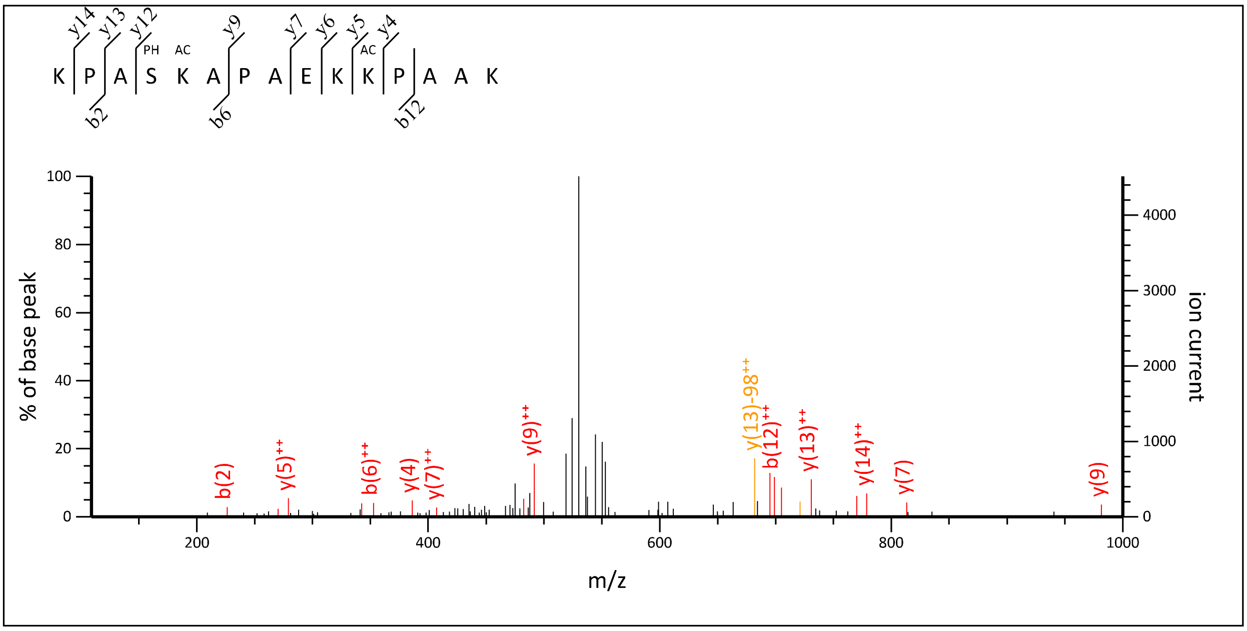

109.03 to 1000

Label all possible matches ○ Label matches used for scoring ◉

```
Monoisotopic mass of neutral peptide Mr(calc): 1684.8912
Fixed modifications: Carbamidomethyl (C) (apply to specified residues or termini only)
Variable modifications:
S4     : Phospho (ST), with neutral losses 0.0000(shown in table), 97.9769
K5     : Acetyl (K)
K11    : Acetyl (K)
Ions Score: 40  Expect: 0.0075
Matches : 24/234 fragment ions using 30 most intense peaks   (help)
```

#	b	b^{++}	b*	b^{*++}	b^0	b^{0++}	Seq.	y	y^{++}	y*	y^{*++}	y^0	y^{0++}	#
1	129.1022	65.0548	112.0757	56.5415			K							15
2	**226.1550**	113.5811	209.1285	105.0679			P	1557.8036	***779.4055***	1540.7771	770.8922	1539.7931	770.4002	14
3	297.1921	149.0997	280.1656	140.5864			A	1460.7509	***730.8791***	1443.7243	722.3658	1442.7403	721.8738	13
4	464.1905	232.5989	447.1639	224.0856	446.1799	223.5936	S	1389.7138	***695.3605***	1372.6872	686.8472	1371.7032	686.3552	12
5	634.2960	317.6516	617.2695	309.1384	616.2854	308.6464	K	1222.7154	611.8613	1205.6889	603.3481	1204.7048	602.8561	11
6	**705.3331**	**353.1702**	688.3066	344.6569	687.3226	344.1649	A	1052.6099	526.8086	1035.5833	518.2953	1034.5993	517.8033	10
7	802.3859	401.6966	785.3593	393.1833	784.3753	392.6913	P	**981.5728**	***491.2900***	964.5462	482.7767	963.5622	482.2847	9
8	873.4230	437.2151	856.3964	428.7019	855.4124	428.2099	A	884.5200	442.7636	867.4934	434.2504	866.5094	433.7584	8
9	1002.4656	501.7364	985.4390	493.2232	984.4550	492.7312	E	**813.4829**	***407.2451***	796.4563	398.7318	795.4723	398.2398	7
10	1130.5606	565.7839	1113.5340	557.2706	1112.5500	556.7786	K	684.4403	***342.7238***	667.4137	334.2105			6
11	1300.6661	650.8367	1283.6395	642.3234	1282.6555	641.8314	K	556.3453	***278.6763***	539.3188	270.1630			5
12	1397.7188	**699.3631**	1380.6923	690.8498	1379.7083	690.3578	P	**386.2398**	193.6235	369.2132	185.1103			4
13	1468.7560	734.8816	1451.7294	726.3683	1450.7454	725.8763	A	289.1870	145.0972	272.1605	136.5839			3
14	1539.7931	**770.4002**	1522.7665	761.8869	1521.7825	761.3949	A	218.1499	109.5786	201.1234	101.0653			2
15							K	147.1128	74.0600	130.0863	65.5468			1

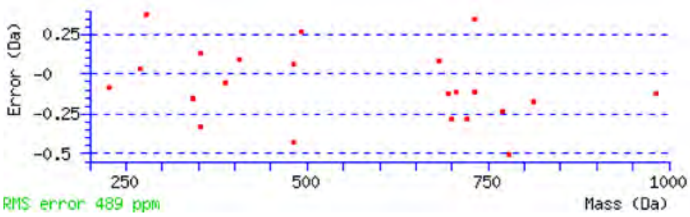

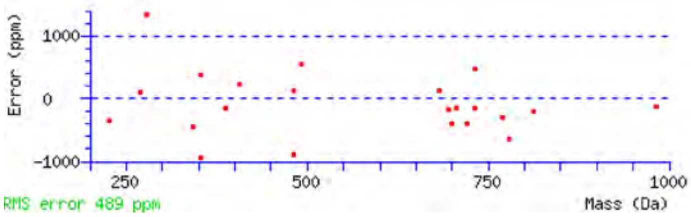

NCBI **BLAST** search of KPASKAPAEKKPAAK
(Parameters: blastp, nr protein database, expect=20000, no filter, PAM30)
Other BLAST web gateways

All matches to this query

Score	Mr(calc)	Delta	Sequence	Site Analysis
39.8	1684.8912	-0.0022	KPASKAPAEKKPAAK	Acetyl K5, K11 69.31%
34.2	1684.8912	-0.0022	KPASKAPAEKKPAAK	Acetyl K5, K15 19.18%
31.4	1684.8912	-0.0022	KPASKAPAEKKPAAK	Acetyl K5, K10 9.88%
16.2	1684.8807	0.0083	KRKSGSNSGTLRMK	
13.8	1684.8807	0.0083	KRKSGSNSGTLRMK	
13.8	1684.8807	0.0083	KRKSGSNSGTLRMK	
13.8	1684.8807	0.0083	KRKSGSNSGTLRMK	
13.8	1684.8807	0.0083	KRKSGSNSGTLRMK	
13.8	1684.8807	0.0083	KRKSGSNSGTLRMK	
13.8	1684.8807	0.0083	KRKSGSNSGTLRMK	

Mascot: http://www.matrixscience.com/

MATRIX SCIENCE Mascot Search Results

Peptide View

MS/MS Fragmentation of **KPASKAPAEKKPAAKK**
Found in **H2B2_YEAST** in **S_cerevisiae_D**, sp|P02294|H2B2_YEAST Histone H2B.2 OS=Saccharomyces cerevisiae (strain ATCC 204508 / S288c) GN=HTB2 PE=1 SV=2

Match to Query 192337: 1775.028852 from(592.683560,3+) intensity(58053.7540) scans(667) rawscans(sn667) rtinseconds(590.3626) index(271782)
Title: 474: Scan 667 (rt=9.83938) [D:\MSData\All\VELOS23960.raw]
Data file D:\Data\MGF\530 Final H2A H2B yeast classical PTMs\mascot_daemon_merge.mgf

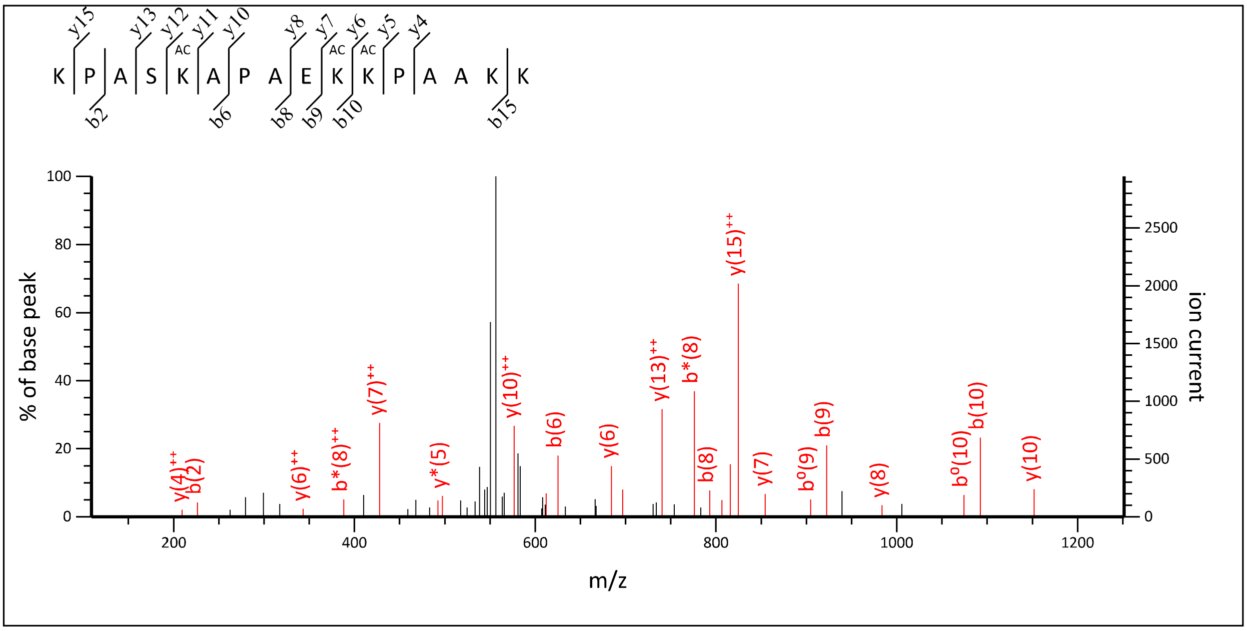

109.06 to 1251.71

Label all possible matches Label matches used for scoring

```
Monoisotopic mass of neutral peptide Mr(calc): 1775.0304
Fixed modifications: Carbamidomethyl (C) (apply to specified residues or termini only)
Variable modifications:
K5     : Acetyl (K)
K10    : Acetyl (K)
K11    : Acetyl (K)
Ions Score: 68  Expect: 3.2e-05
Matches : 31/160 fragment ions using 39 most intense peaks   (help)
```

#	b	b^{++}	b*	b^{*++}	b^0	b^{0++}	Seq.	y	y^{++}	y*	y^{*++}	y^0	y^{0++}	#
1	129.1022	65.0548	112.0757	56.5415			K							16
2	**226.1550**	113.5811	209.1285	105.0679			P	1647.9428	***824.4751***	1630.9163	815.9618	1629.9323	815.4698	15
3	297.1921	149.0997	280.1656	140.5864			A	1550.8901	***775.9487***	1533.8635	767.4354	1532.8795	766.9434	14
4	384.2241	192.6157	367.1976	184.1024	366.2136	183.6104	S	1479.8530	***740.4301***	1462.8264	731.9168	1461.8424	731.4248	13
5	554.3297	277.6685	537.3031	269.1552	536.3191	268.6632	K	1392.8209	***696.9141***	1375.7944	688.4008	1374.8104	687.9088	12
6	**625.3668**	313.1870	608.3402	304.6738	607.3562	304.1817	A	1222.7154	***611.8613***	1205.6889	603.3481	1204.7048	602.8561	11
7	722.4196	361.7134	705.3930	353.2001	704.4090	352.7081	P	**1151.6783**	***576.3428***	1134.6517	567.8295	1133.6677	567.3375	10
8	**793.4567**	397.2320	776.4301	388.7187	775.4461	388.2267	A	1054.6255	527.8164	1037.5990	519.3031	1036.6150	518.8111	9
9	**922.4993**	461.7533	905.4727	453.2400	904.4887	452.7480	E	**983.5884**	***492.2978***	966.5619	483.7846	965.5778	483.2926	8
10	**1092.6048**	546.8060	1075.5782	538.2928	1074.5942	537.8007	K	**854.5458**	***427.7765***	837.5193	419.2633			7
11	1262.7103	631.8588	1245.6838	623.3455	1244.6997	622.8535	K	**684.4403**	***342.7238***	667.4137	334.2105			6
12	1359.7631	680.3852	1342.7365	671.8719	1341.7525	671.3799	P	514.3348	257.6710	497.3082	249.1577			5
13	1430.8002	715.9037	1413.7736	707.3905	1412.7896	706.8985	A	417.2820	***209.1446***	400.2554	200.6314			4
14	1501.8373	751.4223	1484.8108	742.9090	1483.8267	742.4170	A	346.2449	173.6261	329.2183	165.1128			3
15	1629.9323	815.4698	1612.9057	806.9565	1611.9217	806.4645	K	275.2078	138.1075	258.1812	129.5942			2
16							K	147.1128	74.0600	130.0863	65.5468			1

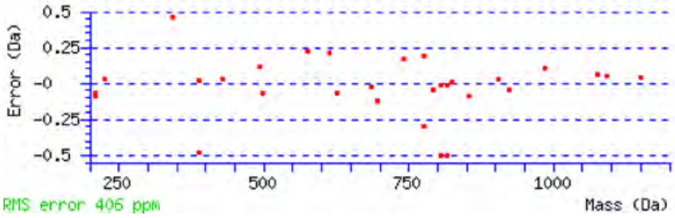

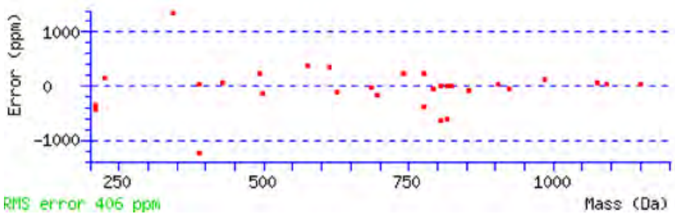

NCBI **BLAST** search of KPASKAPAEKKPAAKK
(Parameters: blastp, nr protein database, expect=20000, no filter, PAM30)
Other BLAST web gateways

All matches to this query

Score	Mr(calc)	Delta	Sequence
68.5	1775.0304	-0.0016	KPASKAPAEKKPAAKK
28.9	1775.0377	-0.0089	SGVKRKRGTSSGSEKK
28.9	1775.0377	-0.0089	SGVKRKRGTSSGSEKK
28.9	1775.0377	-0.0089	SGVKRKRGTSSGSEKK
21.9	1775.0377	-0.0089	SGVKRKRGTSSGSEKK
21.9	1775.0377	-0.0089	SGVKRKRGTSSGSEKK
21.9	1775.0377	-0.0089	SGVKRKRGTSSGSEKK
19.7	1775.0377	-0.0089	SGVKRKRGTSSGSEKK
19.7	1775.0377	-0.0089	SGVKRKRGTSSGSEKK
19.7	1775.0377	-0.0089	SGVKRKRGTSSGSEKK

Mascot: http://www.matrixscience.com/

MATRIX SCIENCE Mascot Search Results

Peptide View

MS/MS Fragmentation of **PASKAPAEKKPAAK**
Found in **H2B2_YEAST** in **S_cerevisiae_D**, sp|P02294|H2B2_YEAST Histone H2B.2 OS=Saccharomyces cerevisiae (strain ATCC 204508 / S288c) GN=HTB2 PE=1 SV=2

Match to Query 145204: 1476.829482 from(493.283770,3+) intensity(26094.0270) scans(850) rawscans(sn850) rtinseconds(636.1738) index(286713)
Title: 605: Scan 850 (rt=10.6029) [D:\MSData\All\VELOS23978.raw]
Data file D:\Data\MGF\530 Final H2A H2B yeast classical PTMs\mascot_daemon_merge.mgf

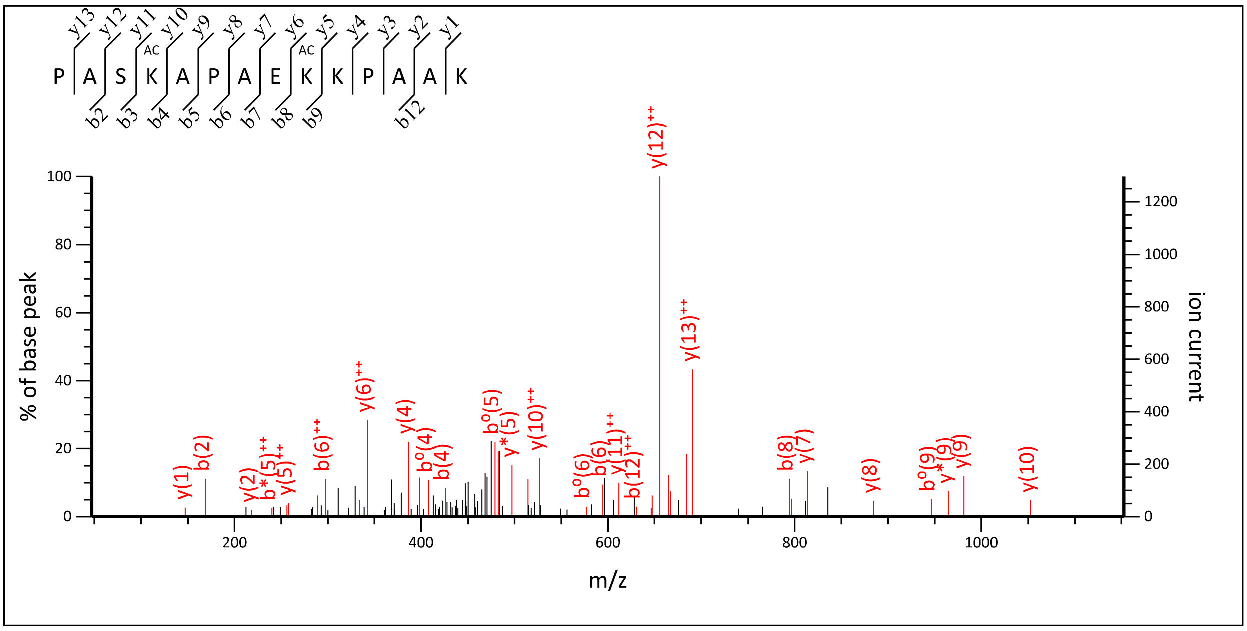

47.16 to 1152.82

Label all possible matches ○ Label matches used for scoring ◉

Monoisotopic mass of neutral peptide Mr(calc): 1476.8300
Fixed modifications: Carbamidomethyl (C) (apply to specified residues or termini only)
Variable modifications:
K4 : Acetyl (K)
K9 : Acetyl (K)
Ions Score: 70 **Expect:** 7.3e-06
Matches : 47/134 fragment ions using 61 most intense peaks (help)

#	b	b++	b*	b*++	b0	b0++	Seq.	y	y++	y*	y*++	y0	y0++	#
1	98.0600	49.5337					P							14
2	**169.0972**	85.0522					A	1380.7845	***690.8959***	1363.7580	682.3826	1362.7740	681.8906	13
3	**256.1292**	128.5682			238.1186	119.5629	S	1309.7474	***655.3774***	1292.7209	646.8641	1291.7369	646.3721	12
4	**426.2347**	213.6210	409.2082	205.1077	408.2241	204.6157	K	1222.7154	***611.8613***	1205.6889	603.3481	1204.7048	602.8561	11
5	**497.2718**	249.1395	480.2453	240.6263	479.2613	240.1343	A	***1052.6099***	***526.8086***	1035.5833	518.2953	1034.5993	517.8033	10
6	**594.3246**	**297.6659**	577.2980	289.1527	576.3140	288.6606	P	***981.5728***	491.2900	964.5462	482.7767	963.5622	482.2847	9
7	**665.3617**	333.1845	648.3352	324.6712	647.3511	324.1792	A	***884.5200***	442.7636	867.4934	434.2504	866.5094	433.7584	8
8	**794.4043**	397.7058	777.3777	389.1925	776.3937	388.7005	E	***813.4829***	407.2451	796.4563	398.7318	795.4723	398.2398	7
9	**964.5098**	**482.7585**	947.4833	474.2453	946.4993	473.7533	K	***684.4403***	***342.7238***	667.4137	334.2105			6
10	1092.6048	546.8060	1075.5782	538.2928	1074.5942	537.8007	K	***514.3348***	***257.6710***	497.3082	249.1577			5
11	1189.6576	595.3324	1172.6310	586.8191	1171.6470	586.3271	P	***386.2398***	193.6235	369.2132	185.1103			4
12	1260.6947	**630.8510**	1243.6681	622.3377	1242.6841	621.8457	A	***289.1870***	145.0972	272.1605	136.5839			3
13	1331.7318	666.3695	1314.7052	657.8563	1313.7212	657.3642	A	***218.1499***	109.5786	201.1234	101.0653			2
14							K	***147.1128***	74.0600	130.0863	65.5468			1

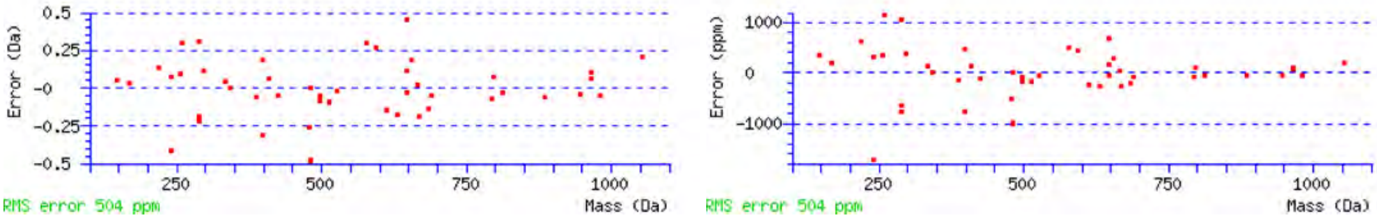

NCBI **BLAST** search of PASKAPAEKKPAAK
(Parameters: blastp, nr protein database, expect=20000, no filter, PAM30)
Other BLAST web gateways

All matches to this query

Score	Mr(calc)	Delta	Sequence	Site Analysis
70.0	1476.8300	-0.0005	PASKAPAEKKPAAK	Acetyl K4, K9 85.52%
62.2	1476.8300	-0.0005	PASKAPAEKKPAAK	Acetyl K4, K10 14.46%
35.2	1476.8300	-0.0005	PASKAPAEKKPAAK	Acetyl K4, K14 0.03%
14.3	1476.8317	-0.0022	KVVKPSEPTKSK	
11.4	1476.8317	-0.0022	KVVKPSEPTKSK	
10.3	1476.8317	-0.0022	KVVKPSEPTKSK	
9.9	1476.8317	-0.0022	KVVKPSEPTKSK	
9.0	1476.8317	-0.0022	TGKPTKLSVQLK	
8.4	1476.8317	-0.0022	KVVKPSEPTKSK	
7.8	1476.8317	-0.0022	KKPAPTGSESKK	

Mascot: http://www.matrixscience.com/

MATRIX SCIENCE Mascot Search Results

Peptide View

MS/MS Fragmentation of **PASKAPAEKKPAAKKTSTSTDGK**
Found in **H2B1_YEAST** in **S_cerevisiae_D**, sp|P02293|H2B1_YEAST Histone H2B.1 OS=Saccharomyces cerevisiae (strain ATCC 204508 / S288c) GN=HTB1 PE=1 SV=2

Match to Query 298122: 2466.296772 from(823.106200,3+) intensity(7859.2515) scans(2377) rawscans(sn2377) rtinseconds(1021.7702) index(288057)
Title: 1949: Scan 2377 (rt=17.0295) [D:\MSData\All\VELOS23978.raw]
Data file D:\Data\MGF\530 Final H2A H2B yeast classical PTMs\mascot_daemon_merge.mgf

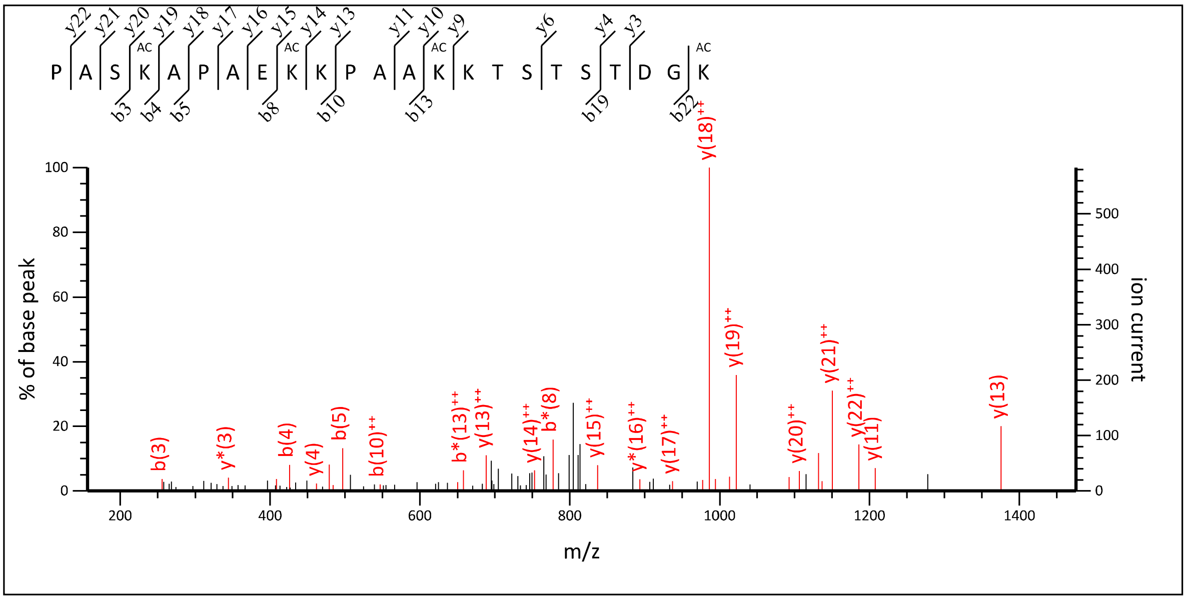

156.24 to 1475.6

Label all possible matches ○ Label matches used for scoring ◉

```
Monoisotopic mass of neutral peptide Mr(calc): 2466.2965
Fixed modifications: Carbamidomethyl (C) (apply to specified residues or termini only)
Variable modifications:
K4     : Acetyl (K)
K9     : Acetyl (K)
K14    : Acetyl (K)
K23    : Acetyl (K)
Ions Score: 40  Expect: 0.0089
Matches : 35/250 fragment ions using 69 most intense peaks   (help)
```

#	b	b^{++}	b*	b^{*++}	b^0	b^{0++}	Seq.	y	y^{++}	y*	y^{*++}	y^0	y^{0++}	#
1	98.0600	49.5337					P							23
2	169.0972	85.0522					A	2370.2511	**1185.6292**	2353.2245	1177.1159	2352.2405	1176.6239	22
3	**256.1292**	128.5682			238.1186	119.5629	S	2299.2140	**1150.1106**	2282.1874	1141.5974	2281.2034	1141.1053	21
4	**426.2347**	213.6210	409.2082	205.1077	408.2241	204.6157	K	2212.1820	**1106.5946**	2195.1554	1098.0813	2194.1714	1097.5893	20
5	**497.2718**	249.1395	480.2453	240.6263	479.2613	240.1343	A	2042.0764	**1021.5419**	2025.0499	1013.0286	2024.0659	1012.5366	19
6	594.3246	297.6659	577.2980	289.1527	576.3140	288.6606	P	1971.0393	**986.0233**	1954.0128	977.5100	1953.0287	977.0180	18
7	665.3617	333.1845	648.3352	324.6712	647.3511	324.1792	A	1873.9865	**937.4969**	1856.9600	928.9836	1855.9760	928.4916	17
8	794.4043	397.7058	777.3777	389.1925	776.3937	388.7005	E	1802.9494	901.9784	1785.9229	893.4651	1784.9389	892.9731	16
9	964.5098	482.7585	947.4833	474.2453	946.4993	473.7533	K	1673.9068	**837.4571**	1656.8803	828.9438	1655.8963	828.4518	15
10	**1092.6048**	546.8060	1075.5782	538.2928	1074.5942	537.8007	K	1503.8013	**752.4043**	1486.7748	743.8910	1485.7907	743.3990	14
11	1189.6576	595.3324	1172.6310	586.8191	1171.6470	586.3271	P	**1375.7064**	**688.3568**	1358.6798	679.8435	1357.6958	679.3515	13
12	1260.6947	630.8510	1243.6681	622.3377	1242.6841	621.8457	A	1278.6536	639.8304	1261.6270	631.3172	1260.6430	630.8251	12
13	1331.7318	666.3695	1314.7052	657.8563	1313.7212	657.3642	A	**1207.6165**	604.3119	1190.5899	595.7986	1189.6059	595.3066	11
14	1501.8373	751.4223	1484.8108	742.9090	1483.8267	742.4170	K	**1136.5794**	568.7933	1119.5528	560.2800	1118.5688	559.7880	10
15	1629.9323	815.4698	1612.9057	806.9565	1611.9217	806.4645	K	966.4738	**483.7406**	949.4473	475.2273	948.4633	474.7353	9
16	1730.9799	865.9936	1713.9534	857.4803	1712.9694	856.9883	T	838.3789	419.6931	821.3523	411.1798	820.3683	410.6878	8
17	1818.0120	909.5096	1800.9854	900.9964	1800.0014	900.5043	S	737.3312	369.1692	720.3046	360.6560	719.3206	360.1640	7
18	1919.0597	960.0335	1902.0331	951.5202	1901.0491	951.0282	T	**650.2992**	325.6532	633.2726	317.1399	632.2886	316.6479	6
19	2006.0917	1003.5495	1989.0651	995.0362	1988.0811	994.5442	S	549.2515	275.1294	532.2249	266.6161	531.2409	266.1241	5

20	2107.1394	1054.0733	2090.1128	1045.5600	2089.1288	1045.0680	T	462.2195	231.6134	445.1929	223.1001	444.2089	222.6081	4
21	2222.1663	1111.5868	2205.1398	1103.0735	2204.1557	1102.5815	D	361.1718	181.0895	344.1452	172.5763	343.1612	172.0842	3
22	2279.1878	1140.0975	2262.1612	1131.5842	2261.1772	1131.0922	G	246.1448	123.5761	229.1183	115.0628			2
23							K	189.1234	95.0653	172.0968	86.5520			1

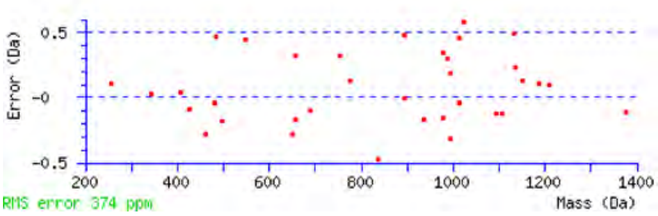

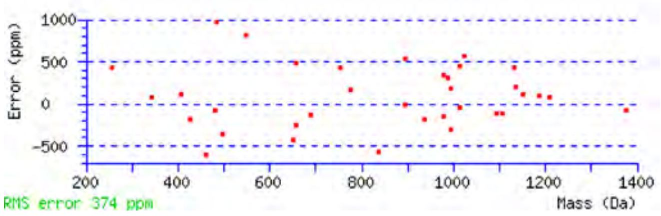

NCBI **BLAST** search of PASKAPAEKKPAAKKTSTSTDGK
(Parameters: blastp, nr protein database, expect=20000, no filter, PAM30)
Other BLAST web gateways

All matches to this query

Score	Mr(calc)	Delta	Sequence	Site Analysis
39.5	2466.2965	0.0003	PASKAPAEKKPAAKKTSTSTDGK	Acetyl K4, K9, K14, K23 34.04%
38.8	2466.2965	0.0003	PASKAPAEKKPAAKKTSTSTDGK	Acetyl K4, K9, K15, K23 28.45%
35.3	2466.2965	0.0003	PASKAPAEKKPAAKKTSTSTDGK	Acetyl K4, K9, K14, K15 12.79%
32.8	2466.2965	0.0003	PASKAPAEKKPAAKKTSTSTDGK	Acetyl K4, K10, K14, K15 7.23%
31.2	2466.2965	0.0003	PASKAPAEKKPAAKKTSTSTDGK	Acetyl K4, K10, K14, K23 4.95%
28.9	2466.2965	0.0003	PASKAPAEKKPAAKKTSTSTDGK	Acetyl K4, K10, K15, K23 2.94%
23.8	2466.2965	0.0003	PASKAPAEKKPAAKKTSTSTDGK	Acetyl K4, K9, K10, K23 0.91%
21.9	2466.2965	0.0003	PASKAPAEKKPAAKKTSTSTDGK	Acetyl K4, K9, K10, K15 0.58%
21.6	2466.2965	0.0003	PASKAPAEKKPAAKKTSTSTDGK	Acetyl K4, K14, K15, K23 0.55%
19.8	2466.2965	0.0003	PASKAPAEKKPAAKKTSTSTDGK	Acetyl K4, K9, K10, K14 0.36%

Mascot: http://www.matrixscience.com/

MATRIX SCIENCE Mascot Search Results

Peptide View

MS/MS Fragmentation of **APAEKKPAAKKTSTSTDGK**
Found in **H2B1_YEAST** in **S_cerevisiae_D**, sp|P02293|H2B1_YEAST Histone H2B.1 OS=Saccharomyces cerevisiae (strain ATCC 204508 / S288c) GN=HTB1 PE=1 SV=2

Match to Query 231824: 1999.058502 from(667.360110,3+) intensity(52096.4650) scans(646) rawscans(sn646) rtinseconds(613.6208) index(54333)
Title: 428: Scan 646 (rt=10.227) [D:\MSData\All\VELOS23666.raw]
Data file D:\Data\MGF\530 Final H2A H2B yeast classical PTMs\mascot_daemon_merge.mgf

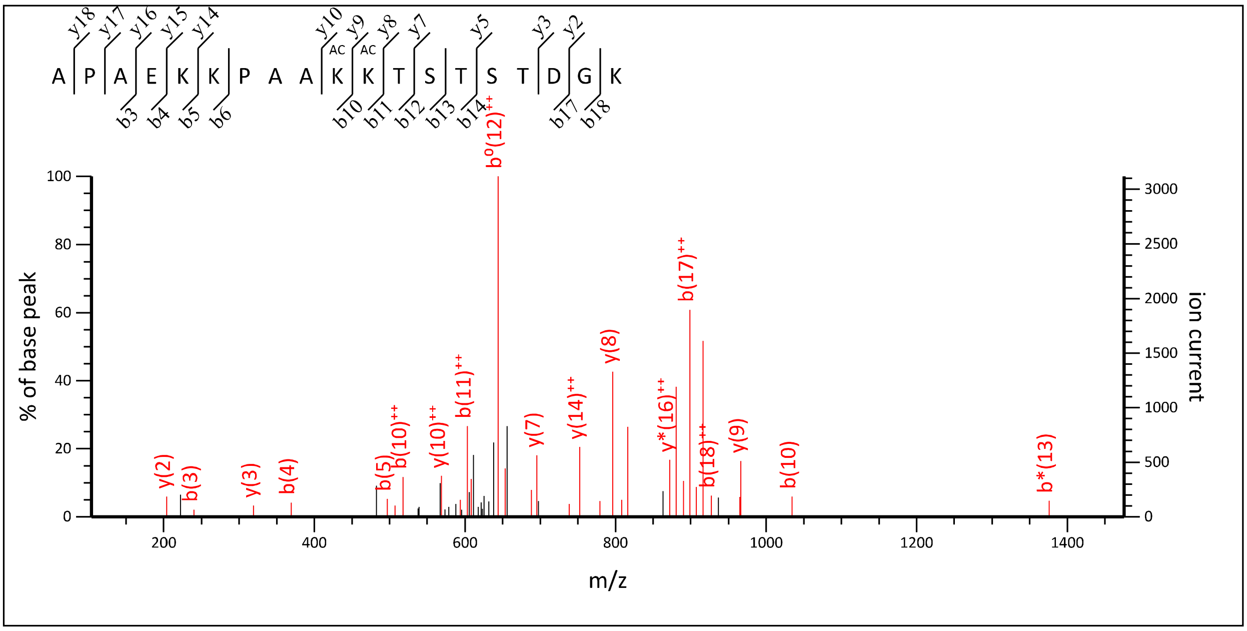

104.02 to 1475.66

Label all possible matches ○ Label matches used for scoring ◉

Monoisotopic mass of neutral peptide Mr(calc): 1999.0585
Fixed modifications: Carbamidomethyl (C) (apply to specified residues or termini only)
Variable modifications:
K10 : Acetyl (K)
K11 : Acetyl (K)
Ions Score: 77 **Expect:** 1.9e-06
Matches : 41/198 fragment ions using 42 most intense peaks (help)

#	b	b++	b*	b*++	b0	b0++	Seq.	y	y++	y*	y*++	y0	y0++	#
1	72.0444	36.5258					A							19
2	169.0972	85.0522					P	1929.0287	965.0180	1912.0022	956.5047	1911.0182	956.0127	18
3	240.1343	120.5708					A	1831.9760	916.4916	1814.9494	907.9784	1813.9654	907.4863	17
4	369.1769	185.0921			351.1663	176.0868	E	1760.9389	880.9731	1743.9123	872.4598	1742.9283	871.9678	16
5	497.2718	249.1395	480.2453	240.6263	479.2613	240.1343	K	1631.8963	816.4518	1614.8697	807.9385	1613.8857	807.4465	15
6	625.3668	313.1870	608.3402	304.6738	607.3562	304.1817	K	1503.8013	752.4043	1486.7748	743.8910	1485.7908	743.3990	14
7	722.4196	361.7134	705.3930	353.2001	704.4090	352.7081	P	1375.7064	688.3568	1358.6798	679.8435	1357.6958	679.3515	13
8	793.4567	397.2320	776.4301	388.7187	775.4461	388.2267	A	1278.6536	639.8304	1261.6270	631.3172	1260.6430	630.8251	12
9	864.4938	432.7505	847.4672	424.2373	846.4832	423.7452	A	1207.6165	604.3119	1190.5899	595.7986	1189.6059	595.3066	11
10	1034.5993	517.8033	1017.5728	509.2900	1016.5887	508.7980	K	1136.5794	568.7933	1119.5528	560.2800	1118.5688	559.7880	10
11	1204.7048	602.8561	1187.6783	594.3428	1186.6943	593.8508	K	966.4738	483.7406	949.4473	475.2273	948.4633	474.7353	9
12	1305.7525	653.3799	1288.7260	644.8666	1287.7419	644.3746	T	796.3683	398.6878	779.3418	390.1745	778.3577	389.6825	8
13	1392.7845	696.8959	1375.7580	688.3826	1374.7740	687.8906	S	695.3206	348.1640	678.2941	339.6507	677.3101	339.1587	7
14	1493.8322	747.4197	1476.8057	738.9065	1475.8217	738.4145	T	608.2886	304.6479	591.2620	296.1347	590.2780	295.6427	6
15	1580.8642	790.9358	1563.8377	782.4225	1562.8537	781.9305	S	507.2409	254.1241	490.2144	245.6108	489.2304	245.1188	5
16	1681.9119	841.4596	1664.8854	832.9463	1663.9014	832.4543	T	420.2089	210.6081	403.1823	202.0948	402.1983	201.6028	4
17	1796.9389	898.9731	1779.9123	890.4598	1778.9283	889.9678	D	319.1612	160.0842	302.1347	151.5710	301.1506	151.0790	3

**18**	1853.9603	**927.4838**	1836.9338	918.9705	1835.9498	918.4785	**G**	*204.1343*	102.5708	187.1077	94.0575			**2**
**19**							**K**	147.1128	74.0600	130.0863	65.5468			**1**

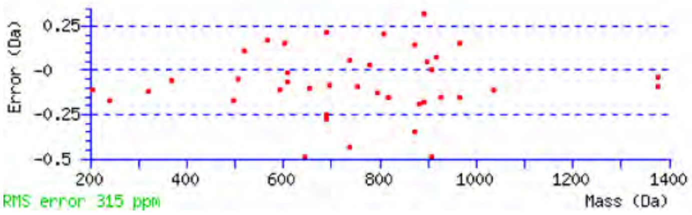

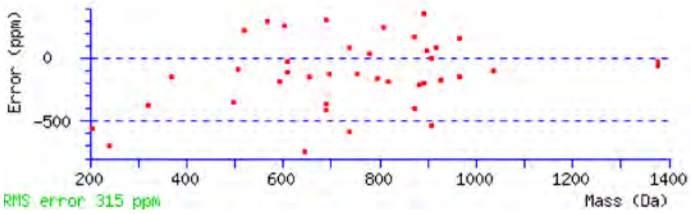

NCBI **BLAST** search of APAEKKPAAKKTSTSTDGK
(Parameters: blastp, nr protein database, expect=20000, no filter, PAM30)
Other BLAST web gateways

All matches to this query

Score	Mr(calc)	Delta	Sequence	Site Analysis
77.1	1999.0585	-0.0000	APAEKKPAAKKTSTSTDGK	Acetyl K10, K11 98.40%
58.2	1999.0585	-0.0000	APAEKKPAAKKTSTSTDGK	Acetyl K6, K11 1.28%
48.0	1999.0585	-0.0000	APAEKKPAAKKTSTSTDGK	Acetyl K5, K11 0.12%
48.0	1999.0585	-0.0000	APAEKKPAAKKTSTSTDGK	Acetyl K6, K10 0.12%
44.2	1999.0585	-0.0000	APAEKKPAAKKTSTSTDGK	Acetyl K5, K6 0.05%
40.1	1999.0585	-0.0000	APAEKKPAAKKTSTSTDGK	Acetyl K5, K10 0.02%
25.5	1999.0585	-0.0000	APAEKKPAAKKTSTSTDGK	Acetyl K10, K19 0.00%
19.8	1999.0585	-0.0000	APAEKKPAAKKTSTSTDGK	Acetyl K11, K19 0.00%
15.4	1999.0577	0.0008	SKIDVIDKEYMKRPK	
14.1	1999.0503	0.0082	KEPVKTPSPAPAAKISSR	

Mascot: http://www.matrixscience.com/

MATRIX SCIENCE Mascot Search Results

Peptide View

MS/MS Fragmentation of **KETYSSYIYK**
Found in **H2B2_YEAST** in **S_cerevisiae_D**, sp|P02294|H2B2_YEAST Histone H2B.2 OS=Saccharomyces cerevisiae (strain ATCC 204508 / S288c) GN=HTB2 PE=1 SV=2

Match to Query 111539: 1308.660608 from(655.337580,2+) intensity(67607.4380) scans(4126) rawscans(sn4126) rtinseconds(1514.1556) index(202836)
Title: 3431: Scan 4126 (rt=25.2359) [D:\MSData\All\VELOS23958.raw]
Data file D:\Data\MGF\530 Final H2A H2B yeast classical PTMs\mascot_daemon_merge.mgf

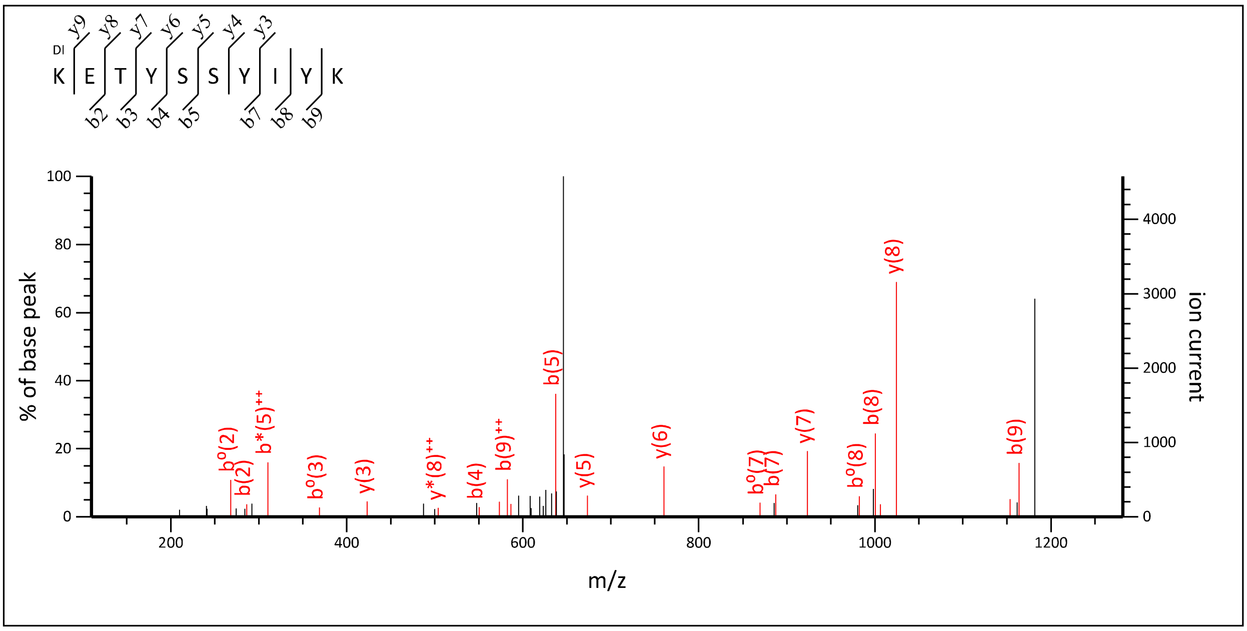

109.95 to 1281.56

Label all possible matches Label matches used for scoring

```
Monoisotopic mass of neutral peptide Mr(calc): 1308.6601
Fixed modifications: Carbamidomethyl (C) (apply to specified residues or termini only)
Variable modifications:
K1     : Dimethyl (K)
Ions Score: 51  Expect: 0.00084
Matches : 26/98 fragment ions using 42 most intense peaks   (help)
```

#	b	b^{++}	b*	b^{*++}	b^0	b^{0++}	Seq.	y	y^{++}	y*	y^{*++}	y^0	y^{0++}	#
1	157.1335	79.0704	140.1070	70.5571			K							10
2	**286.1761**	143.5917	269.1496	135.0784	268.1656	134.5864	E	***1153.5412***	577.2742	1136.5146	568.7610	1135.5306	568.2689	9
3	387.2238	194.1155	370.1973	185.6023	369.2132	185.1103	T	***1024.4986***	512.7529	1007.4720	504.2397	1006.4880	503.7477	8
4	**550.2871**	275.6472	533.2606	267.1339	532.2766	266.6419	Y	***923.4509***	462.2291	906.4244	453.7158	905.4403	453.2238	7
5	**637.3192**	319.1632	620.2926	310.6499	619.3086	310.1579	S	***760.3876***	380.6974	743.3610	372.1842	742.3770	371.6921	6
6	724.3512	362.6792	707.3246	354.1660	706.3406	353.6740	S	***673.3556***	337.1814	656.3290	328.6681	655.3450	328.1761	5
7	**887.4145**	444.2109	870.3880	435.6976	869.4040	435.2056	Y	***586.3235***	293.6654	569.2970	285.1521			4
8	**1000.4986**	500.7529	983.4720	492.2397	982.4880	491.7477	I	***423.2602***	212.1337	406.2336	203.6205			3
9	**1163.5619**	582.2846	1146.5354	573.7713	1145.5514	573.2793	Y	***310.1761***	155.5917	293.1496	147.0784			2
10							K	147.1128	74.0600	130.0863	65.5468			1

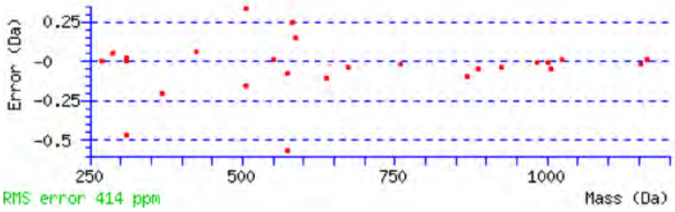

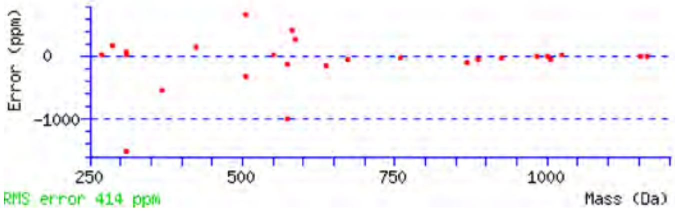

NCBI **BLAST** search of KETYSSYIYK
(Parameters: blastp, nr protein database, expect=20000, no filter, PAM30)
Other BLAST web gateways

All matches to this query

Score	Mr(calc)	Delta	Sequence
50.6	1308.6601	0.0005	KETYSSYIYK
21.8	1308.6591	0.0015	GDIHYIRLTK
16.9	1308.6607	-0.0001	TKRLISIYK
11.6	1308.6608	-0.0002	IPVIKHITTK
10.0	1308.6561	0.0045	KKENDDTIYK
10.0	1308.6561	0.0045	KKENDDTIYK
9.5	1308.6551	0.0055	KVSANDKNRK
9.1	1308.6608	-0.0002	QYMEKIREGR
9.1	1308.6607	-0.0001	TKRLISIYK
9.0	1308.6591	0.0015	KTQKFHNEK

Mascot: http://www.matrixscience.com/

MATRIX SCIENCE Mascot Search Results

Peptide View

MS/MS Fragmentation of **KETYSSYIYKVLK**
Found in **H2B2_YEAST** in **S_cerevisiae_D**, sp|P02294|H2B2_YEAST Histone H2B.2 OS=Saccharomyces cerevisiae (strain ATCC 204508 / S288c) GN=HTB2 PE=1 SV=2

Match to Query 174679: 1662.886392 from(555.302740,3+) intensity(41989.3130) scans(9021) rawscans(sn9021) rtinseconds(2997.0281) index(75479)
Title: 7476: Scan 9021 (rt=49.9505) [D:\MSData\All\VELOS23668.raw]
Data file D:\Data\MGF\530 Final H2A H2B yeast classical PTMs\mascot_daemon_merge.mgf

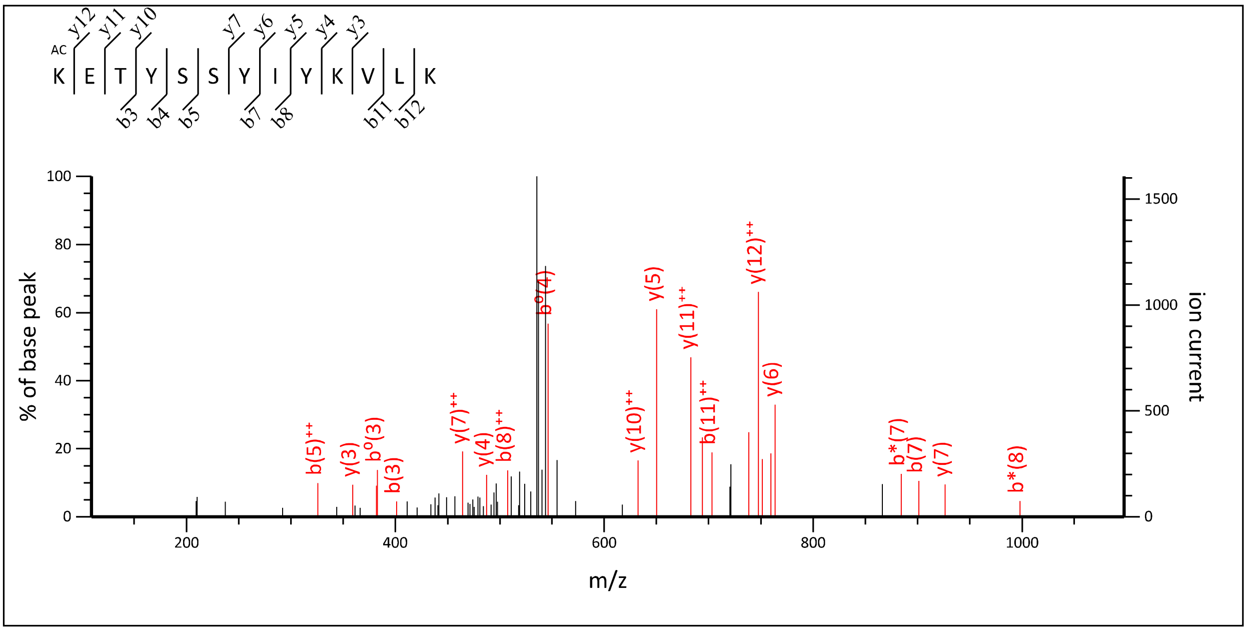

109.09 to 1097.89

Label all possible matches ○ Label matches used for scoring ◉

Monoisotopic mass of neutral peptide Mr(calc): 1662.8868
Fixed modifications: Carbamidomethyl (C) (apply to specified residues or termini only)
Variable modifications:
K1 : Acetyl (K)
Ions Score: 46 **Expect:** 0.002
Matches : 27/128 fragment ions using 34 most intense peaks (help)

#	b	b^{++}	b*	b^{*++}	b^0	b^{0++}	Seq.	y	y^{++}	y*	y^{*++}	y^0	y^{0++}	#
1	171.1128	86.0600	154.0863	77.5468			K							13
2	300.1554	150.5813	283.1288	142.0681	282.1448	141.5761	E	1493.7886	***747.3980***	1476.7621	738.8847	1475.7781	738.3927	12
3	**401.2031**	201.1052	384.1765	192.5919	383.1925	192.0999	T	1364.7460	***682.8767***	1347.7195	674.3634	1346.7355	673.8714	11
4	564.2664	282.6368	547.2399	274.1236	546.2558	273.6316	Y	1263.6984	***632.3528***	1246.6718	623.8395	1245.6878	623.3475	10
5	651.2984	***326.1529***	634.2719	317.6396	633.2879	317.1476	S	1100.6350	550.8211	1083.6085	542.3079	1082.6245	541.8159	9
6	**738.3305**	369.6689	721.3039	361.1556	720.3199	360.6636	S	1013.6030	***507.3051***	996.5764	498.7919	995.5924	498.2999	8
7	**901.3938**	451.2005	884.3672	442.6873	883.3832	442.1953	Y	***926.5710***	***463.7891***	909.5444	455.2758			7
8	1014.4779	***507.7426***	997.4513	499.2293	996.4673	498.7373	I	***763.5076***	***382.2575***	746.4811	373.7442			6
9	1177.5412	589.2742	1160.5146	580.7610	1159.5306	580.2689	Y	***650.4236***	***325.7154***	633.3970	317.2022			5
10	1305.6361	653.3217	1288.6096	644.8084	1287.6256	644.3164	K	***487.3602***	244.1838	470.3337	235.6705			4
11	1404.7046	***702.8559***	1387.6780	694.3426	1386.6940	693.8506	V	***359.2653***	180.1363	342.2387	171.6230			3
12	1517.7886	***759.3980***	1500.7621	750.8847	1499.7781	750.3927	L	260.1969	130.6021	243.1703	122.0888			2
13							K	147.1128	74.0600	130.0863	65.5468			1

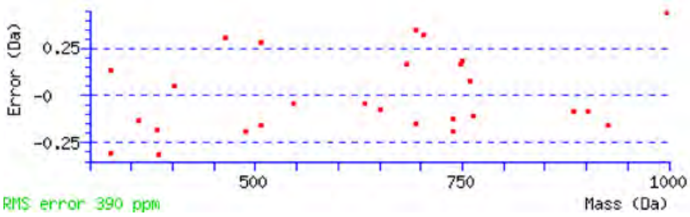

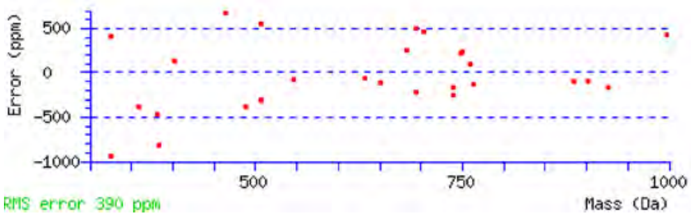

NCBI **BLAST** search of KETYSSYIYKVLK
(Parameters: blastp, nr protein database, expect=20000, no filter, PAM30)
Other BLAST web gateways

All matches to this query

Score	Mr(calc)	Delta	Sequence
46.0	1662.8868	-0.0004	KETYSSYIYKVLK
16.4	1662.8842	0.0022	YNSNKREVAWGVAK
13.4	1662.8858	0.0006	AFNKVDNITLKHK
11.9	1662.8874	-0.0010	LGSAKLKSLILYR
11.4	1662.8858	0.0006	AFNKVDNITLKHK
11.3	1662.8858	0.0006	GLERSIINVLAWGK
11.2	1662.8858	0.0006	AFNKVDNITLKHK
11.2	1662.8858	0.0006	AFNKVDNITLKHK
10.8	1662.8842	0.0022	RPVLRNYFTADNK
7.1	1662.8842	0.0022	RPVLRNYFTADNK

Mascot: http://www.matrixscience.com/

MATRIX SCIENCE Mascot Search Results

Peptide View

MS/MS Fragmentation of **KETYSSYIYK**
Found in **H2B2_YEAST** in **S_cerevisiae_D**, sp|P02294|H2B2_YEAST Histone H2B.2 OS=Saccharomyces cerevisiae (strain ATCC 204508 / S288c) GN=HTB2 PE=1 SV=2

Match to Query 122225: 1360.594748 from(681.304650,2+) intensity(29592.3050) scans(4663) rawscans(sn4663) rtinseconds(1648.8972) index(306031)
Title: 3916: Scan 4663 (rt=27.4816) [D:\MSData\All\VELOS23980.raw]
Data file D:\Data\MGF\530 Final H2A H2B yeast classical PTMs\mascot_daemon_merge.mgf

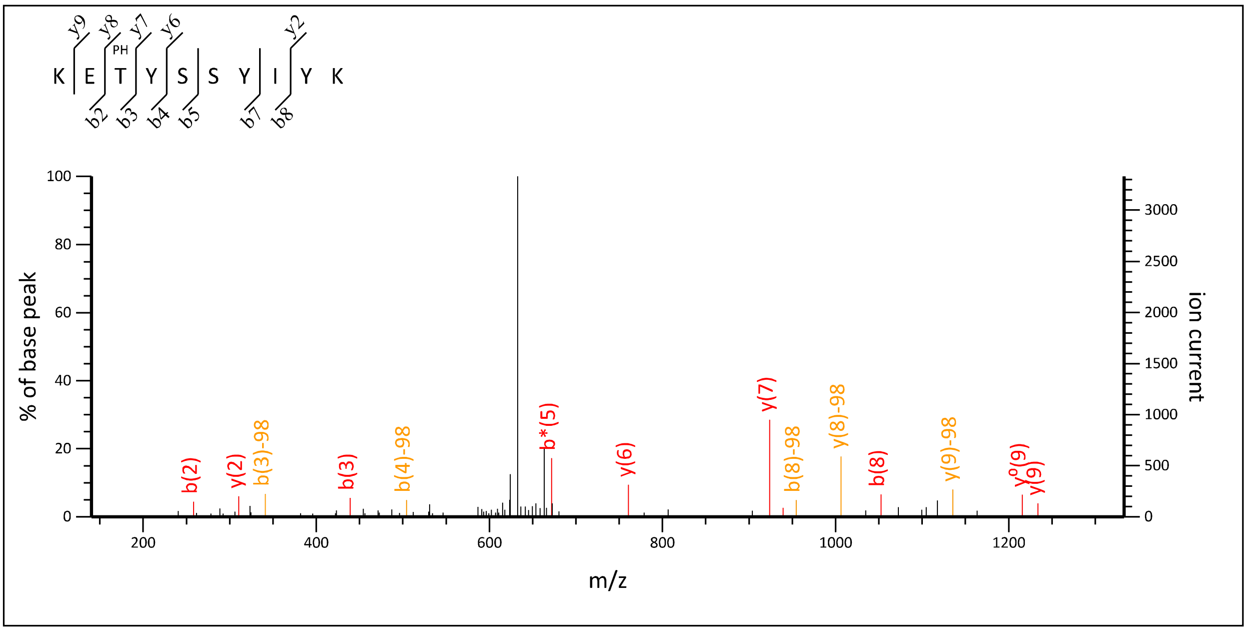

140.22 to 1333.48

Label all possible matches ○ Label matches used for scoring ◉

```
Monoisotopic mass of neutral peptide Mr(calc): 1360.5952
Fixed modifications: Carbamidomethyl (C) (apply to specified residues or termini only)
Variable modifications:
T3     : Phospho (ST), with neutral losses 0.0000(shown in table), 97.9769
Ions Score: 45  Expect: 0.0012
Matches : 16/152 fragment ions using 20 most intense peaks   (help)
```

#	b	b^{++}	b^{*}	b^{*++}	b^{0}	b^{0++}	Seq.	y	y^{++}	y^{*}	y^{*++}	y^{0}	y^{0++}	#
1	129.1022	65.0548	112.0757	56.5415			K							10
2	***258.1448***	129.5761	241.1183	121.0628	240.1343	120.5708	E	***1233.5075***	617.2574	1216.4810	608.7441	1215.4969	608.2521	9
3	***439.1588***	220.0831	422.1323	211.5698	421.1483	211.0778	T	1104.4649	552.7361	1087.4384	544.2228	1086.4544	543.7308	8
4	602.2222	301.6147	585.1956	293.1014	584.2116	292.6094	Y	***923.4509***	462.2291	906.4244	453.7158	905.4403	453.2238	7
5	689.2542	345.1307	672.2277	336.6175	671.2436	336.1255	S	***760.3876***	380.6974	743.3610	372.1842	742.3770	371.6921	6
6	776.2862	388.6468	759.2597	380.1335	758.2757	379.6415	S	673.3556	337.1814	656.3290	328.6681	655.3450	328.1761	5
7	***939.3496***	470.1784	922.3230	461.6651	921.3390	461.1731	Y	586.3235	293.6654	569.2970	285.1521			4
8	***1052.4336***	526.7204	1035.4071	518.2072	1034.4231	517.7152	I	423.2602	212.1337	406.2336	203.6205			3
9	***1215.4969***	608.2521	1198.4704	599.7388	1197.4864	599.2468	Y	***310.1761***	155.5917	293.1496	147.0784			2
10							K	147.1128	74.0600	130.0863	65.5468			1

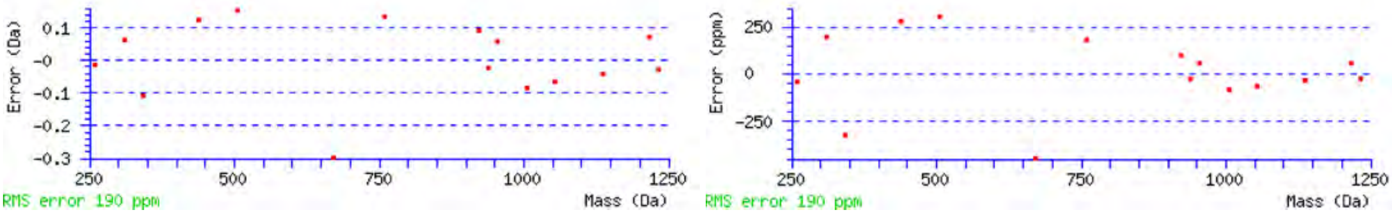

NCBI **BLAST** search of KETYSSYIYK
(Parameters: blastp, nr protein database, expect=20000, no filter, PAM30)
Other BLAST web gateways

All matches to this query

Score	Mr(calc)	Delta	Sequence	Site Analysis
44.8	1360.5952	-0.0004	KETYSSYIYK	Phospho T3 98.87%
22.4	1360.5952	-0.0004	KETYSSYIYK	Phospho S6 0.56%
22.4	1360.5952	-0.0004	KETYSSYIYK	Phospho S5 0.56%
18.2	1360.5911	0.0036	KSDGNDSILYK	
14.9	1360.5911	0.0036	NTDKEKAEYK	
14.7	1360.5911	0.0036	KSDGNDSILYK	
9.8	1360.5911	0.0036	NTDKEKAEYK	
7.5	1360.6007	-0.0060	SSHPQNQGSEYK	
6.4	1360.5885	0.0063	VSGGRSNHNDPK	
5.3	1360.6013	-0.0066	KRTNRNNTK	

Mascot: http://www.matrixscience.com/

Mascot Search Results

Peptide View

MS/MS Fragmentation of **KETYSSYIYK**
Found in **H2B1_YEAST** in **S_cerevisiae_D**, sp|P02293|H2B1_YEAST Histone H2B.1 OS=Saccharomyces cerevisiae (strain ATCC 204508 / S288c) GN=HTB1 PE=1 SV=2

Match to Query 122217: 1360.589448 from(681.302000,2+) intensity(12669.4620) scans(3860) rawscans(sn3860) rtinseconds(1438.0211) index(320459)
Title: 3176: Scan 3860 (rt=23.967) [D:\MSData\All\VELOS23982.raw]
Data file D:\Data\MGF\530 Final H2A H2B yeast classical PTMs\mascot_daemon_merge.mgf

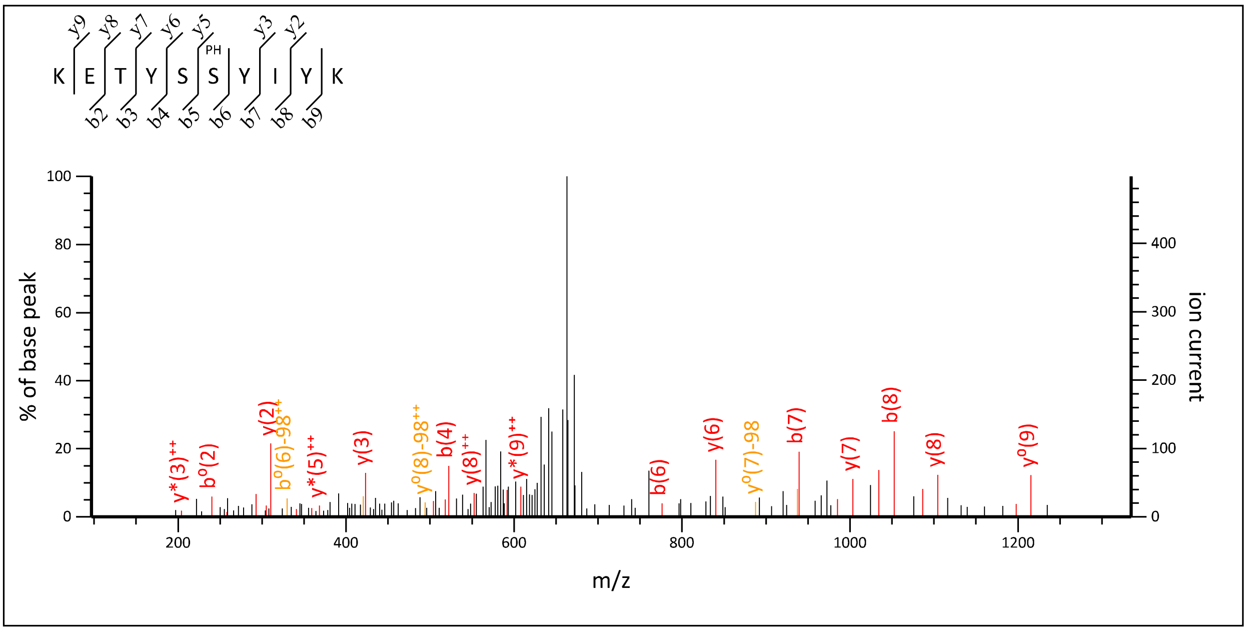

97.11 to 1334.7

Label all possible matches ◉ Label matches used for scoring ○

Monoisotopic mass of neutral peptide Mr(calc): 1360.5952
Fixed modifications: Carbamidomethyl (C) (apply to specified residues or termini only)
Variable modifications:
S6 : Phospho (ST), with neutral losses 0.0000(shown in table), 97.9769
Ions Score: 28 **Expect:** 0.065 (help)

#	b	b++	b*	b*++	b0	b0++	Seq.	y	y++	y*	y*++	y0	y0++	#
1	129.1022	65.0548	112.0757	56.5415			K							10
2	**258.1448**	129.5761	241.1183	121.0628	**240.1343**	120.5708	E	1233.5075	617.2574	1216.4810	**608.7441**	**1215.4969**	**608.2521**	9
3	**359.1925**	180.0999	342.1660	171.5866	**341.1819**	171.0946	T	**1104.4649**	**552.7361**	1087.4384	544.2228	**1086.4544**	543.7308	8
4	**522.2558**	261.6316	505.2293	253.1183	**504.2453**	252.6263	Y	**1003.4172**	502.2123	986.3907	**493.6990**	**985.4067**	493.2070	7
5	609.2879	**305.1476**	592.2613	296.6343	**591.2773**	296.1423	S	**840.3539**	**420.6806**	823.3274	412.1673	822.3433	411.6753	6
6	**776.2862**	388.6468	759.2597	380.1335	758.2757	379.6415	S	753.3219	377.1646	736.2953	**368.6513**	735.3113	**368.1593**	5
7	**939.3496**	470.1784	922.3230	461.6651	921.3390	461.1731	Y	586.3235	293.6654	569.2970	285.1521			4
8	**1052.4336**	526.7204	1035.4071	**518.2072**	**1034.4231**	**517.7152**	I	**423.2602**	212.1337	406.2336	**203.6205**			3
9	**1215.4969**	**608.2521**	1198.4704	599.7388	**1197.4864**	599.2468	Y	**310.1761**	155.5917	**293.1496**	147.0784			2
10							K	147.1128	74.0600	130.0863	65.5468			1

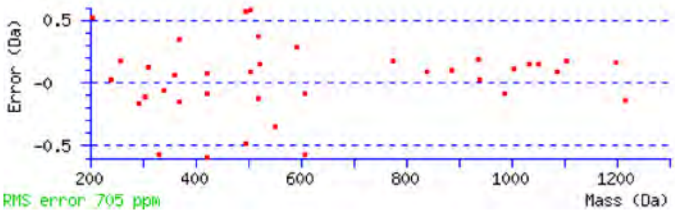

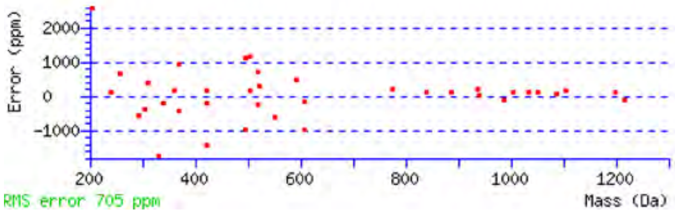

NCBI **BLAST** search of KETYSSYIYK
(Parameters: blastp, nr protein database, expect=20000, no filter, PAM30)
Other BLAST web gateways

All matches to this query

Score	Mr(calc)	Delta	Sequence
27.7	1360.5952	-0.0057	KETYSSYIYK
27.7	1360.5952	-0.0057	KETYSSYIYK
27.7	1360.5952	-0.0057	KETYSSYIYK
18.2	1360.5911	-0.0017	KSDGNDSILYK
11.6	1360.5911	-0.0017	KSDGNDSILYK
7.9	1360.5925	-0.0030	SAANKHNWPDK
5.6	1360.5869	0.0025	VSFKPGSFYK
1.3	1360.5869	0.0025	VSFKPGSFYK
0.5	1360.5911	-0.0017	YEAKEKDTNK
0.2	1360.5958	-0.0063	KLFSTRLTK

Mascot: http://www.matrixscience.com/

Mascot Search Results

Peptide View

MS/MS Fragmentation of **ETYSSYIYK**
Found in **H2B2_YEAST** in **S_cerevisiae_D**, sp|P02294|H2B2_YEAST Histone H2B.2 OS=Saccharomyces cerevisiae (strain ATCC 204508 / S288c) GN=HTB2 PE=1 SV=2

Match to Query 86023: 1194.545708 from(598.280130,2+) intensity(28208.6840) scans(4996) rawscans(sn4996) rtinseconds(1663.85) index(290344)
Title: 4236: Scan 4996 (rt=27.7308) [D:\MSData\All\VELOS23978.raw]
Data file D:\Data\MGF\530 Final H2A H2B yeast classical PTMs\mascot_daemon_merge.mgf

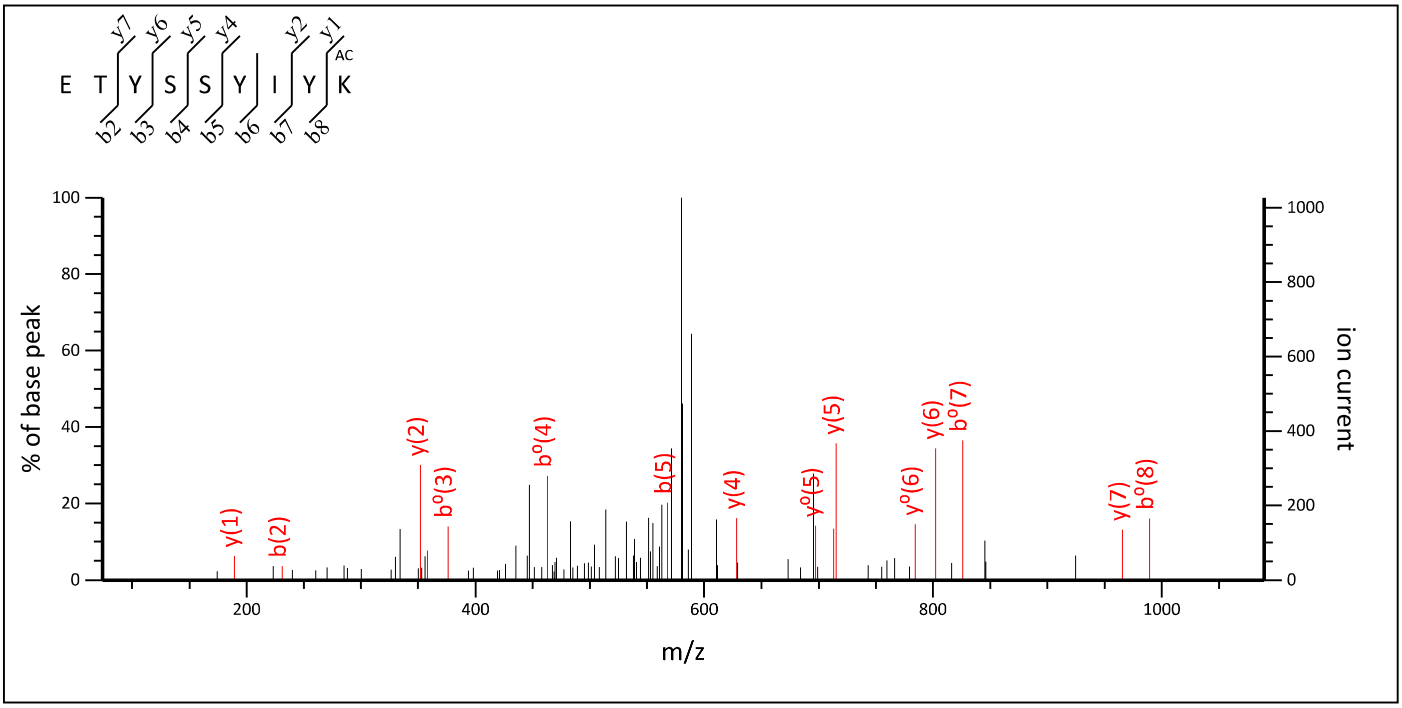

74.15 to 1089.42

Label all possible matches ○ Label matches used for scoring ◉

Monoisotopic mass of neutral peptide Mr(calc): 1194.5444
Fixed modifications: Carbamidomethyl (C) (apply to specified residues or termini only)
Variable modifications:
K9 : Acetyl (K)
Ions Score: 35 **Expect:** 0.0022
Matches : 16/72 fragment ions using 31 most intense peaks (help)

#	b	b^{++}	b^0	b^{0++}	Seq.	y	y^{++}	y*	y^{*++}	y^0	y^{0++}	#
1	130.0499	65.5286	112.0393	56.5233	E							9
2	**231.0975**	116.0524	213.0870	107.0471	T	1066.5092	533.7582	1049.4826	525.2449	1048.4986	524.7529	8
3	394.1609	197.5841	376.1503	188.5788	Y	***965.4615***	483.2344	948.4349	474.7211	947.4509	474.2291	7
4	481.1929	241.1001	463.1823	232.0948	S	***802.3981***	401.7027	785.3716	393.1894	784.3876	392.6974	6
5	**568.2249**	284.6161	550.2144	275.6108	S	***715.3661***	358.1867	698.3396	349.6734	697.3556	349.1814	5
6	731.2883	366.1478	713.2777	357.1425	Y	***628.3341***	314.6707	611.3075	306.1574			4
7	844.3723	422.6898	826.3618	413.6845	I	465.2708	233.1390	448.2442	224.6257			3
8	1007.4357	504.2215	989.4251	495.2162	Y	***352.1867***	176.5970	335.1601	168.0837			2
9					K	***189.1234***	95.0653	172.0968	86.5520			1

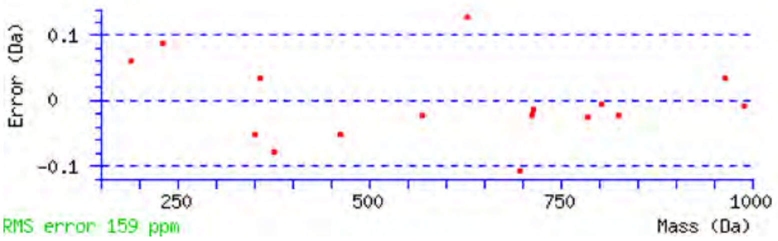

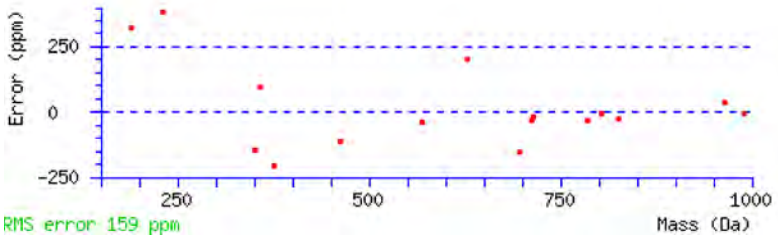

NCBI **BLAST** search of ETYSSYIYK
(Parameters: blastp, nr protein database, expect=20000, no filter, PAM30)
Other BLAST web gateways

All matches to this query

Score	Mr(calc)	Delta	Sequence
34.9	1194.5444	0.0013	ETYSSYIYK
6.1	1194.5451	0.0006	SSLIFVTIR
6.0	1194.5451	0.0006	SSLIFVTIR
6.0	1194.5451	0.0006	RYLTSIVK
4.1	1194.5517	-0.0060	IFDSSQTNNK
2.6	1194.5451	0.0006	FPKSGKSSK
1.4	1194.5517	-0.0060	KGDEYNGNDK
1.3	1194.5517	-0.0060	TLTQYDGNNK
0.9	1194.5484	-0.0027	KTSKSVKGM
0.1	1194.5451	0.0006	LQSGSKFVK

Mascot: http://www.matrixscience.com/

Mascot Search Results

Peptide View

MS/MS Fragmentation of **ETYSSYIYK**
Found in **H2B2_YEAST** in **S_cerevisiae_D**, sp|P02294|H2B2_YEAST Histone H2B.2 OS=Saccharomyces cerevisiae (strain ATCC 204508 / S288c) GN=HTB2 PE=1 SV=2

Match to Query 80912: 1166.549668 from(584.282110,2+) intensity(19309.8980) scans(5325) rawscans(sn5325) rtinseconds(1741.7943) index(290635)
Title: 4527: Scan 5325 (rt=29.0299) [D:\MSData\All\VELOS23978.raw]
Data file D:\Data\MGF\530 Final H2A H2B yeast classical PTMs\mascot_daemon_merge.mgf

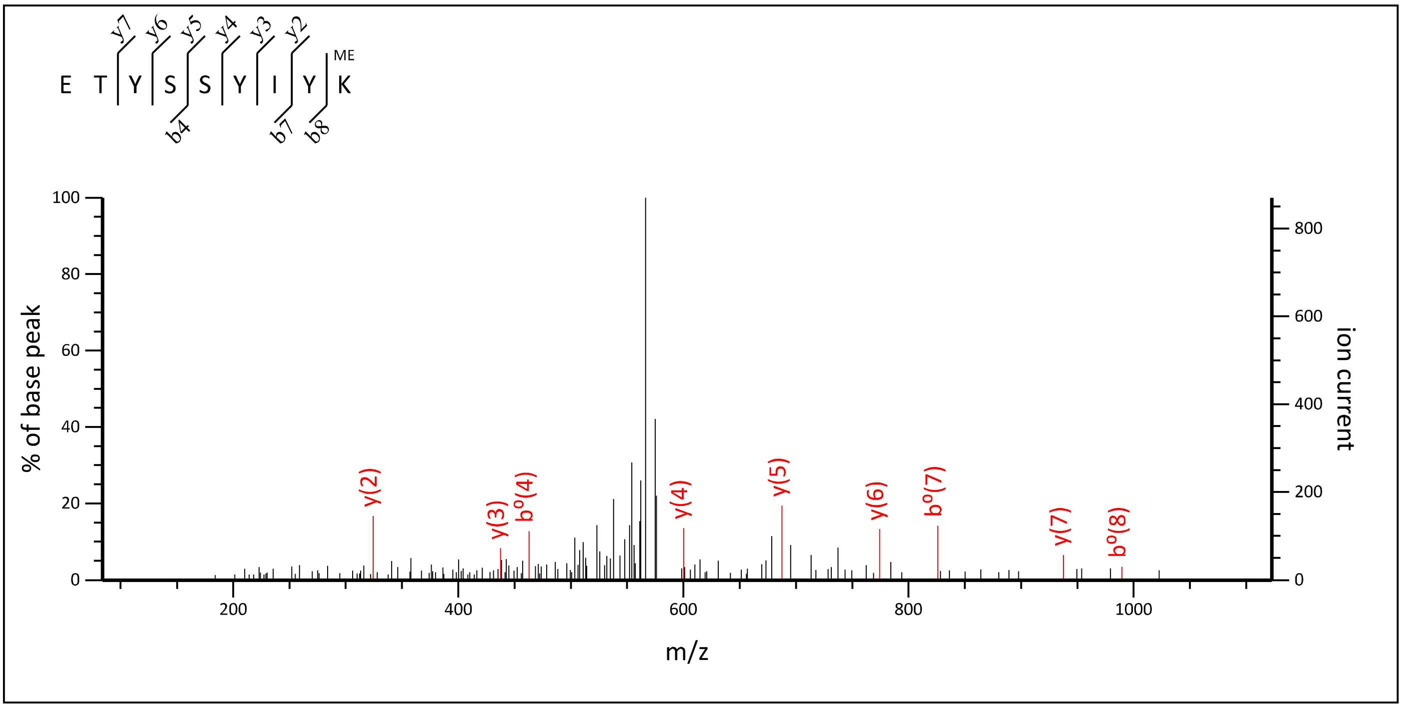

84.04 to 1122.67

Label all possible matches ○ Label matches used for scoring ◉

Monoisotopic mass of neutral peptide Mr(calc): 1166.5495
Fixed modifications: Carbamidomethyl (C) (apply to specified residues or termini only)
Variable modifications:
K9 : Methyl (K)
Ions Score: 27 **Expect:** 0.0067
Matches : 9/72 fragment ions using 18 most intense peaks (help)

#	b	b^{++}	b^{0}	b^{0++}	Seq.	y	y^{++}	y*	y^{*++}	y^{0}	y^{0++}	#
1	130.0499	65.5286	112.0393	56.5233	E							9
2	231.0975	116.0524	213.0870	107.0471	T	1038.5142	519.7608	1021.4877	511.2475	1020.5037	510.7555	8
3	394.1609	197.5841	376.1503	188.5788	Y	***937.4666***	469.2369	920.4400	460.7236	919.4560	460.2316	7
4	481.1929	241.1001	463.1823	232.0948	S	***774.4032***	387.7053	757.3767	379.1920	756.3927	378.7000	6
5	568.2249	284.6161	550.2144	275.6108	S	***687.3712***	344.1892	670.3447	335.6760	669.3606	335.1840	5
6	731.2883	366.1478	713.2777	357.1425	Y	***600.3392***	300.6732	583.3126	292.1600			4
7	844.3723	422.6898	826.3618	413.6845	I	***437.2758***	219.1416	420.2493	210.6283			3
8	1007.4357	504.2215	989.4251	495.2162	Y	***324.1918***	162.5995	307.1652	154.0863			2
9					K	161.1285	81.0679	144.1019	72.5546			1

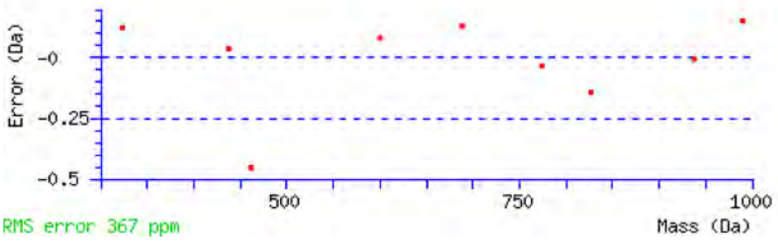

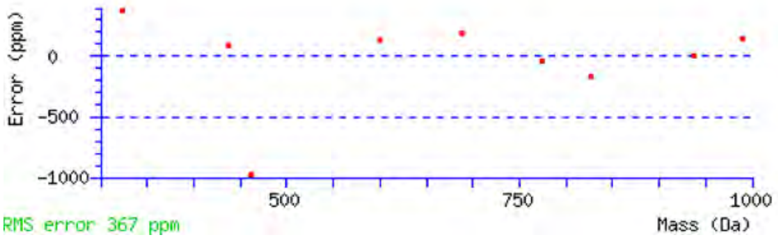

NCBI **BLAST** search of ETYSSYIYK
(Parameters: blastp, nr protein database, expect=20000, no filter, PAM30)
Other BLAST web gateways

All matches to this query

Score	Mr(calc)	Delta	Sequence
27.0	1166.5495	0.0001	ETYSSYIYK
3.6	1166.5501	-0.0005	SKKKAFTK
3.5	1166.5485	0.0012	DEIHKFTR
1.7	1166.5455	0.0042	ENVKSSYEK
1.6	1166.5525	-0.0029	FSFLRSFK
1.4	1166.5489	0.0008	TLSCISETSK
1.1	1166.5455	0.0042	EEEEKEYR
0.9	1166.5445	0.0052	TLKAQNGASR
0.5	1166.5444	0.0052	SRAEREQK

Mascot: http://www.matrixscience.com/

MATRIX SCIENCE Mascot Search Results

Peptide View

MS/MS Fragmentation of **VLKQTHPDTGISQK**
Found in **H2B2_YEAST** in **S_cerevisiae_D**, sp|P02294|H2B2_YEAST Histone H2B.2 OS=Saccharomyces cerevisiae (strain ATCC 204508 / S288c) GN=HTB2 PE=1 SV=2

Match to Query 162951: 1592.851062 from(531.957630,3+) intensity(30684.2190) scans(2149) rawscans(sn2149) rtinseconds(1015.2969) index(55618)
Title: 1713: Scan 2149 (rt=16.9216) [D:\MSData\All\VELOS23666.raw]
Data file D:\Data\MGF\530 Final H2A H2B yeast classical PTMs\mascot_daemon_merge.mgf

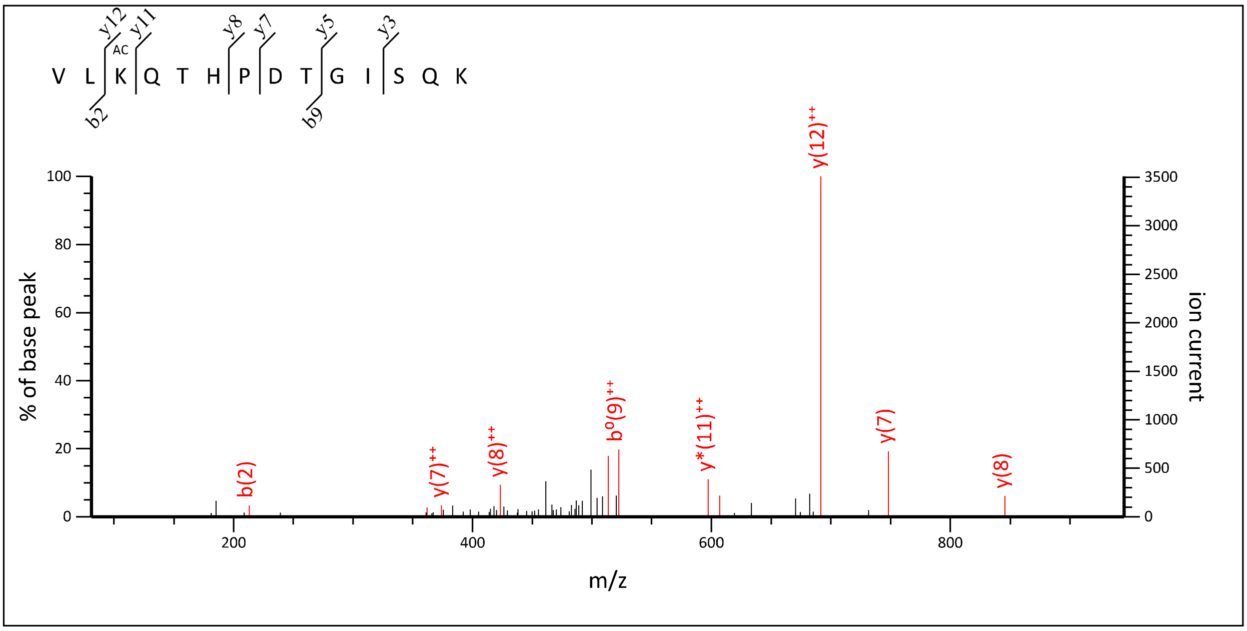

81.12 to 945.5

Label all possible matches Label matches used for scoring

```
Monoisotopic mass of neutral peptide Mr(calc): 1592.8522
Fixed modifications: Carbamidomethyl (C) (apply to specified residues or termini only)
Variable modifications:
K3     : Acetyl (K)
Ions Score: 33  Expect: 0.094
Matches : 13/140 fragment ions using 13 most intense peaks   (help)
```

#	b	b++	b*	b*++	b0	b0++	Seq.	y	y++	y*	y*++	y0	y0++	#
1	100.0757	50.5415					V							14
2	213.1598	107.0835					L	1494.7911	***747.8992***	1477.7645	739.3859	1476.7805	738.8939	13
3	383.2653	192.1363	366.2387	183.6230			K	1381.7070	***691.3571***	1364.6805	682.8439	1363.6965	682.3519	12
4	511.3239	256.1656	494.2973	247.6523			Q	1211.6015	***606.3044***	1194.5749	597.7911	1193.5909	597.2991	11
5	612.3715	306.6894	595.3450	298.1761	594.3610	297.6841	T	1083.5429	542.2751	1066.5164	533.7618	1065.5323	533.2698	10
6	749.4305	375.2189	732.4039	366.7056	731.4199	366.2136	H	982.4952	491.7513	965.4687	483.2380	964.4847	482.7460	9
7	846.4832	423.7452	829.4567	415.2320	828.4726	414.7400	P	**845.4363**	***423.2218***	828.4098	414.7085	827.4258	414.2165	8
8	961.5102	481.2587	944.4836	472.7454	943.4996	472.2534	D	**748.3836**	***374.6954***	731.3570	366.1821	730.3730	365.6901	7
9	1062.5578	531.7826	1045.5313	523.2693	1044.5473	522.7773	T	633.3566	317.1819	616.3301	308.6687	615.3461	308.1767	6
10	1119.5793	560.2933	1102.5528	551.7800	1101.5687	551.2880	G	532.3089	266.6581	515.2824	258.1448	514.2984	257.6528	5
11	1232.6634	616.8353	1215.6368	608.3220	1214.6528	607.8300	I	475.2875	238.1474	458.2609	229.6341	457.2769	229.1421	4
12	1319.6954	660.3513	1302.6688	651.8381	1301.6848	651.3461	S	**362.2034**	181.6053	345.1769	173.0921	344.1928	172.6001	3
13	1447.7540	724.3806	1430.7274	715.8673	1429.7434	715.3753	Q	275.1714	138.0893	258.1448	129.5761			2
14							K	147.1128	74.0600	130.0863	65.5468			1

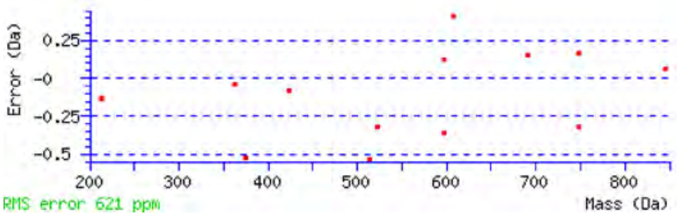

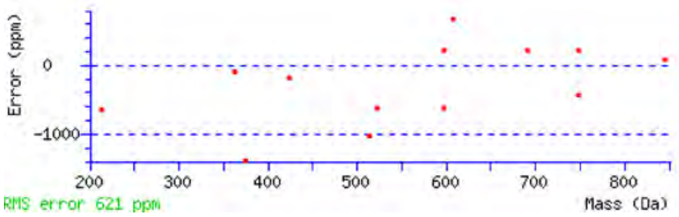

NCBI **BLAST** search of VLKQTHPDTGISQK
(Parameters: blastp, nr protein database, expect=20000, no filter, PAM30)
Other BLAST web gateways

All matches to this query

Score	Mr(calc)	Delta	Sequence
32.8	1592.8522	-0.0012	VLKQTHPDTGISQK
20.8	1592.8514	-0.0003	MVVFKNIGHIITK
17.8	1592.8569	-0.0058	VLAHEAQNRMNLR
14.3	1592.8569	-0.0058	VLAHEAQNRMNLR
13.6	1592.8511	-0.0001	NKSVRSRNNANK
13.6	1592.8511	-0.0001	NKSVRSRNNANK
13.5	1592.8539	-0.0028	IVKSLETGIDQVR
13.5	1592.8539	-0.0028	LGTGLGKSAIDGVKK
13.5	1592.8539	-0.0028	LGTGLGKSAIDGVKK
13.5	1592.8539	-0.0028	LGTGLGKSAIDGVKK

Mascot: http://www.matrixscience.com/

Mascot Search Results

Peptide View

MS/MS Fragmentation of **IATEASKLAAYNK**
Found in **H2B2_YEAST** in **S_cerevisiae_D**, sp|P02294|H2B2_YEAST Histone H2B.2 OS=Saccharomyces cerevisiae (strain ATCC 204508 / S288c) GN=HTB2 PE=1 SV=2

Match to Query 134482: 1420.754688 from(711.384620,2+) intensity(31025.3330) scans(4830-4839) rawscans(sn4830:sn4839) rtinseconds(1748.5231-1751.0392) index(159720)
Title: 3952: Sum of 2 scans in range 4830 (rt=29.1421) to 4839 (rt=29.184) [D:\MSData\All\VELOS23670.raw]
Data file D:\Data\MGF\530 Final H2A H2B yeast classical PTMs\mascot_daemon_merge.mgf

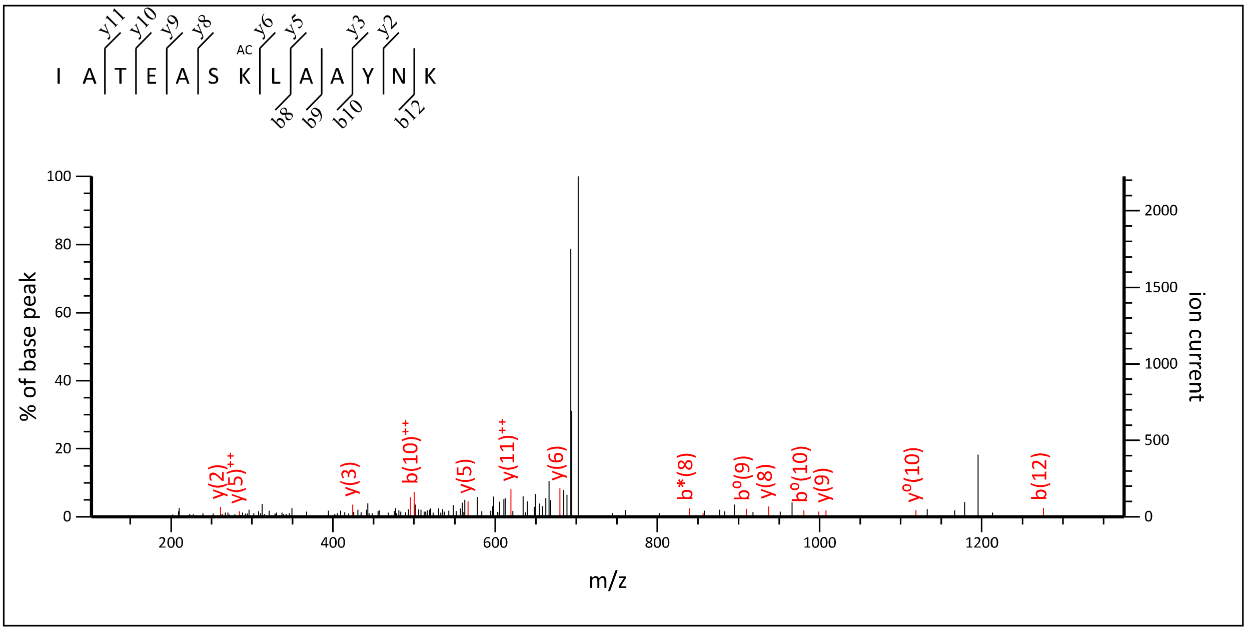

102.12 to 1375.63

Label all possible matches ○ Label matches used for scoring ◉

```
Monoisotopic mass of neutral peptide Mr(calc): 1420.7561
Fixed modifications: Carbamidomethyl (C) (apply to specified residues or termini only)
Variable modifications:
K7     : Acetyl (K)
Ions Score: 24  Expect: 0.058
Matches : 19/114 fragment ions using 53 most intense peaks   (help)
```

#	b	b^{++}	b*	b^{*++}	b^0	b^{0++}	Seq.	y	y^{++}	y*	y^{*++}	y^0	y^{0++}	#
1	114.0913	57.5493					I							13
2	185.1285	93.0679					A	1308.6794	654.8433	1291.6529	646.3301	1290.6688	645.8381	12
3	286.1761	143.5917			268.1656	134.5864	T	1237.6423	619.3248	1220.6157	610.8115	1219.6317	610.3195	11
4	415.2187	208.1130			397.2082	199.1077	E	1136.5946	568.8009	1119.5681	560.2877	1118.5841	559.7957	10
5	486.2558	243.6316			468.2453	234.6263	A	*1007.5520*	504.2796	990.5255	495.7664	989.5415	495.2744	9
6	573.2879	287.1476			555.2773	278.1423	S	*936.5149*	468.7611	919.4884	460.2478	918.5043	459.7558	8
7	743.3934	372.2003	726.3668	363.6871	725.3828	363.1951	K	849.4829	425.2451	832.4563	416.7318			7
8	**856.4775**	428.7424	839.4509	420.2291	838.4669	419.7371	L	*679.3774*	340.1923	662.3508	331.6790			6
9	927.5146	464.2609	910.4880	455.7477	909.5040	455.2556	A	*566.2933*	283.6503	549.2667	275.1370			5
10	**998.5517**	499.7795	981.5251	491.2662	980.5411	490.7742	A	*495.2562*	248.1317	478.2296	239.6185			4
11	1161.6150	581.3111	1144.5885	572.7979	1143.6045	572.3059	Y	*424.2191*	212.6132	407.1925	204.0999			3
12	1275.6579	638.3326	1258.6314	629.8193	1257.6474	629.3273	N	*261.1557*	131.0815	244.1292	122.5682			2
13							K	147.1128	74.0600	130.0863	65.5468			1

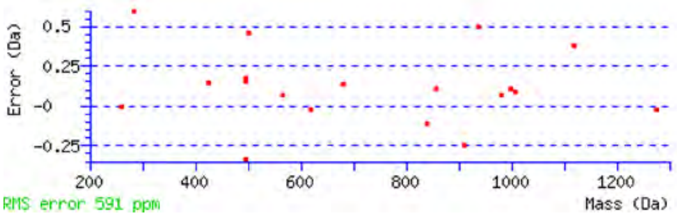

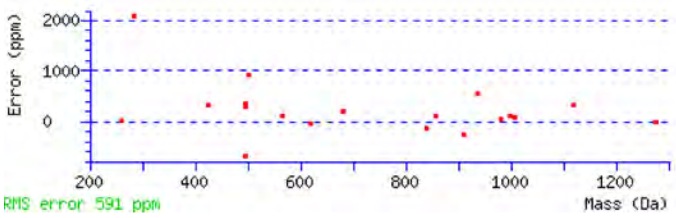

NCBI **BLAST** search of IATEASKLAAYNK
(Parameters: blastp, nr protein database, expect=20000, no filter, PAM30)
Other BLAST web gateways

All matches to this query

Score	Mr(calc)	Delta	Sequence
23.6	1420.7561	-0.0014	IATEASKLAAYNK
9.9	1420.7496	0.0051	FLSKKGIISAK
9.1	1420.7479	0.0068	LLSFGAGPNPLKK
7.8	1420.7479	0.0068	TSYKRKYTPK
7.8	1420.7479	0.0068	TSYKRKYTPK
7.5	1420.7570	-0.0023	DMKNIFCIKPK
7.4	1420.7479	0.0068	YKAGKYLKTNK
7.1	1420.7562	-0.0015	LFKSSDGVNEKK
6.0	1420.7479	0.0068	NTKLYKGAKYK
5.7	1420.7575	-0.0028	ERNNSLVWPHK

Mascot: http://www.matrixscience.com/

Mascot Search Results

Peptide View

MS/MS Fragmentation of **LILPGELAK**
Found in **H2B2_YEAST** in **S_cerevisiae_D**, sp|P02294|H2B2_YEAST Histone H2B.2 OS=Saccharomyces cerevisiae (strain ATCC 204508 / S288c) GN=HTB2 PE=1 SV=2

Match to Query 37555: 952.595368 from(477.304960,2+) intensity(15880.6040) scans(14418) rawscans(sn14418) rtinseconds(4933.5697) index(269171)
Title: 11652: Scan 14418 (rt=82.2262) [D:\MSData\All\VELOS23664.raw]
Data file D:\Data\MGF\530 Final H2A H2B yeast classical PTMs\mascot_daemon_merge.mgf

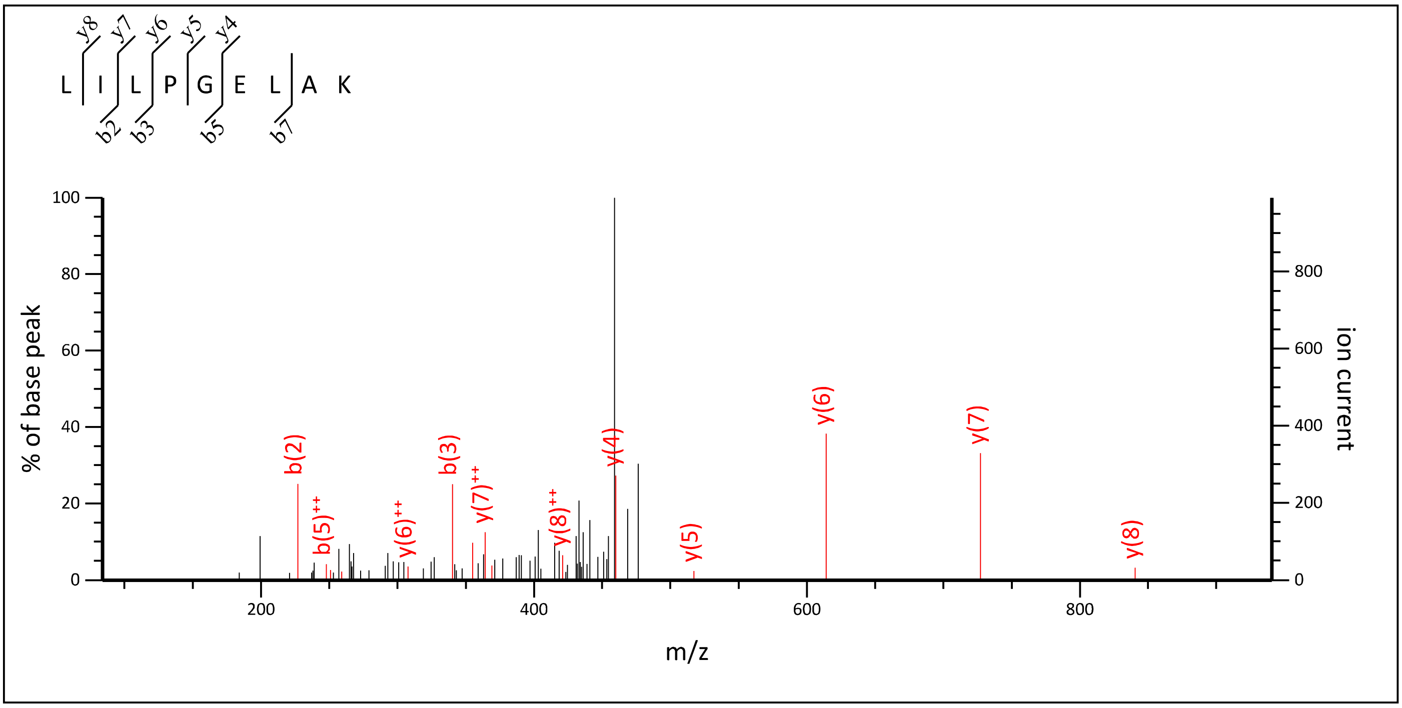

83.96 to 940.61

Label all possible matches Label matches used for scoring

Monoisotopic mass of neutral peptide Mr(calc): 952.5957
Fixed modifications: Carbamidomethyl (C) (apply to specified residues or termini only)
Ions Score: 38 **Expect:** 0.0041 (help)

#	b	b++	b0	b0++	Seq.	y	y++	y*	y*++	y0	y0++	#
1	114.0913	57.5493			L							9
2	**227.1754**	114.0913			I	**840.5189**	**420.7631**	823.4924	412.2498	822.5084	411.7578	8
3	**340.2595**	170.6334			L	**727.4349**	**364.2211**	710.4083	355.7078	709.4243	**355.2158**	7
4	437.3122	219.1598			P	**614.3508**	**307.6790**	597.3243	299.1658	596.3402	298.6738	6
5	494.3337	**247.6705**			G	**517.2980**	**259.1527**	500.2715	**250.6394**	499.2875	250.1474	5
6	623.3763	312.1918	605.3657	303.1865	E	**460.2766**	230.6419	443.2500	222.1287	442.2660	221.6366	4
7	736.4604	**368.7338**	718.4498	359.7285	L	331.2340	166.1206	314.2074	157.6074			3
8	807.4975	404.2524	789.4869	395.2471	A	218.1499	109.5786	201.1234	101.0653			2
9					K	147.1128	74.0600	130.0863	65.5468			1

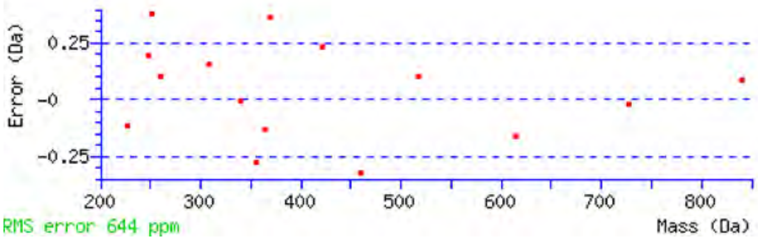

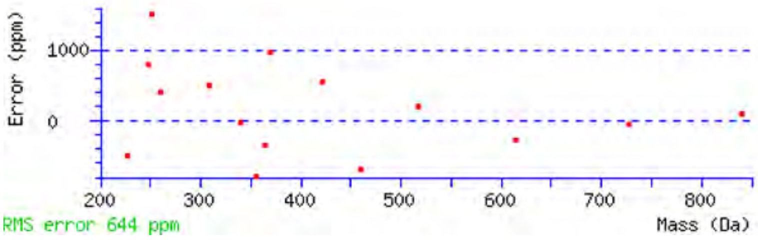

NCBI **BLAST** search of LILPGELAK
(Parameters: blastp, nr protein database, expect=20000, no filter, PAM30)
Other BLAST web gateways

All matches to this query

Score	Mr(calc)	Delta	Sequence
38.2	952.5957	-0.0003	LILPGELAK
22.5	952.5957	-0.0003	ILINDPIK
22.5	952.5957	-0.0003	EPLPSLKK
22.5	952.5957	-0.0003	EPLPSLKK
22.5	952.5957	-0.0003	EPLPSLKK
22.5	952.5957	-0.0003	EPLPSLKK
13.1	952.5957	-0.0003	ILPLDLQK
7.5	952.5957	-0.0003	IPETPVKK
7.5	952.5957	-0.0003	IPETPVKK
7.0	952.5957	-0.0003	IIGIPKDK

Mascot: http://www.matrixscience.com/

MATRIX SCIENCE Mascot Search Results

Peptide View

MS/MS Fragmentation of **LILPGELAK**
Found in **H2B2_YEAST** in **S_cerevisiae_D**, sp|P02294|H2B2_YEAST Histone H2B.2 OS=Saccharomyces cerevisiae (strain ATCC 204508 / S288c) GN=HTB2 PE=1 SV=2

Match to Query 48552: 994.605748 from(498.310150,2+) intensity(389924.0900) scans(7593) rawscans(sn7593) rtinseconds(2563.5222) index(74306)
Title: 6303: Scan 7593 (rt=42.7254) [D:\MSData\All\VELOS23668.raw]
Data file D:\Data\MGF\530 Final H2A H2B yeast classical PTMs\mascot_daemon_merge.mgf

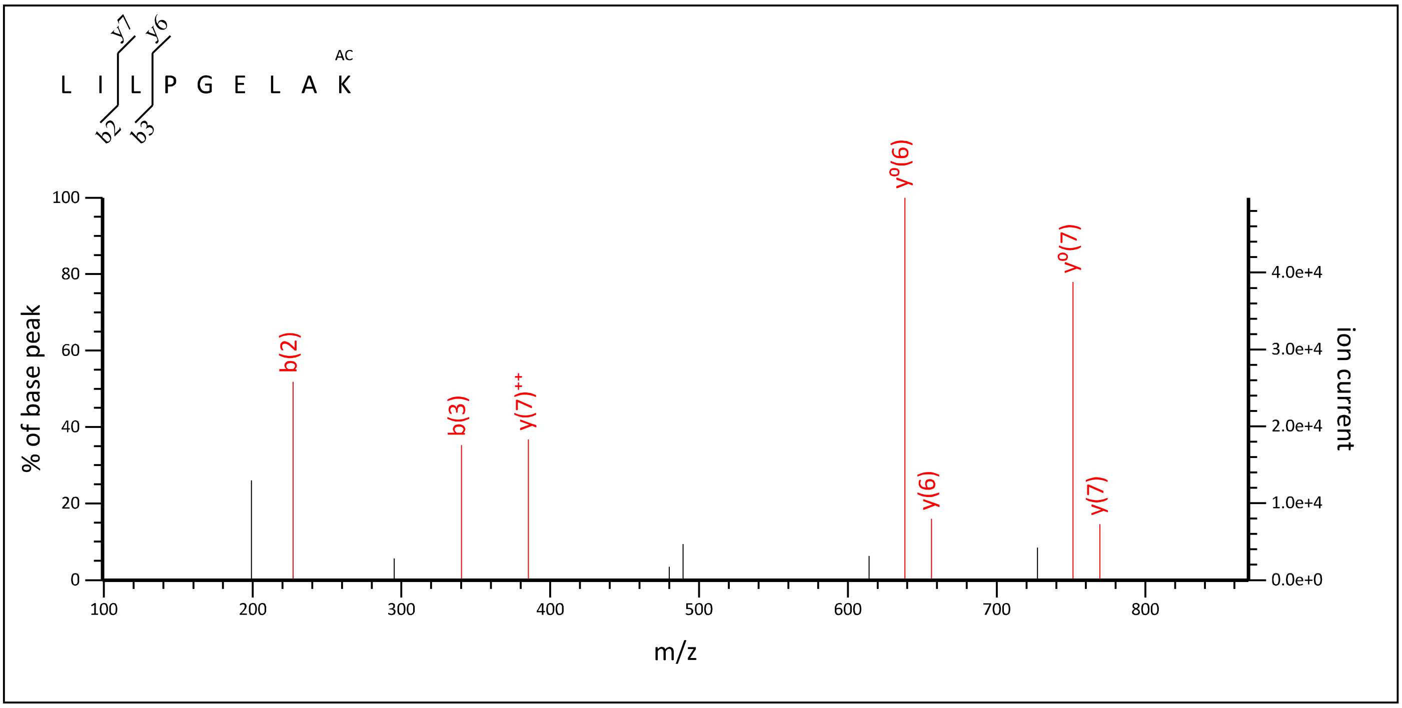

99.12 to 869.36

Label all possible matches ◉ Label matches used for scoring ○

Monoisotopic mass of neutral peptide Mr(calc): 994.6062
Fixed modifications: Carbamidomethyl (C) (apply to specified residues or termini only)
Variable modifications:
K9 : Acetyl (K)
Ions Score: 15 **Expect:** 0.28 (help)

#	b	b^{++}	b^{0}	b^{0++}	Seq.	y	y^{++}	y*	y^{*++}	y^{0}	y^{0++}	#
1	114.0913	57.5493			L							9
2	**227.1754**	114.0913			I	882.5295	441.7684	865.5029	433.2551	864.5189	432.7631	8
3	**340.2595**	170.6334			L	**769.4454**	**385.2264**	752.4189	376.7131	**751.4349**	376.2211	7
4	437.3122	219.1598			P	**656.3614**	328.6843	639.3348	320.1710	**638.3508**	319.6790	6
5	494.3337	247.6705			G	559.3086	280.1579	542.2821	271.6447	541.2980	271.1527	5
6	623.3763	312.1918	605.3657	303.1865	E	502.2871	251.6472	485.2606	243.1339	484.2766	242.6419	4
7	736.4604	368.7338	718.4498	359.7285	L	373.2445	187.1259	356.2180	178.6126			3
8	807.4975	404.2524	789.4869	395.2471	A	260.1605	130.5839	243.1339	122.0706			2
9					K	189.1234	95.0653	172.0968	86.5520			1

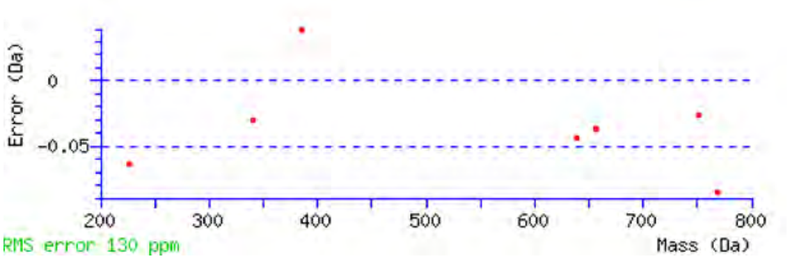

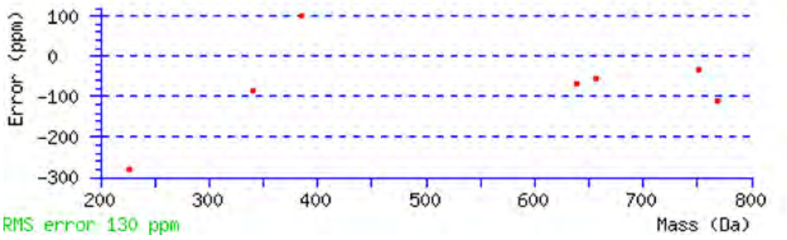

NCBI **BLAST** search of LILPGELAK
(Parameters: blastp, nr protein database, expect=20000, no filter, PAM30)
Other BLAST web gateways

All matches to this query

Score	Mr(calc)	Delta	Sequence
15.2	994.6062	-0.0005	EPLPSLKK
15.2	994.6062	-0.0005	EPLPSLKK
15.2	994.6062	-0.0005	LILPGELAK
8.7	994.6052	0.0005	RKKGSKK
8.7	994.6052	0.0005	RKKGSKK
3.7	994.6052	0.0005	RKKKGTK
3.7	994.6052	0.0005	RKKKGTK
3.7	994.6052	0.0005	RKKKGTK
3.7	994.6052	0.0005	RKKKGTK
3.5	994.6036	0.0022	GIITRSHR

Mascot: http://www.matrixscience.com/

Mascot Search Results

Peptide View

MS/MS Fragmentation of **LILPGELAK**
Found in **H2B2_YEAST** in **S_cerevisiae_D**, sp|P02294|H2B2_YEAST Histone H2B.2 OS=Saccharomyces cerevisiae (strain ATCC 204508 / S288c) GN=HTB2 PE=1 SV=2

Match to Query 41957: 966.611588 from(484.313070,2+) intensity(25441.6130) scans(8548) rawscans(sn8548) rtinseconds(2549.5655) index(293444)
Title: 7336: Scan 8548 (rt=42.4928) [D:\MSData\All\VELOS23978.raw]
Data file D:\Data\MGF\530 Final H2A H2B yeast classical PTMs\mascot_daemon_merge.mgf

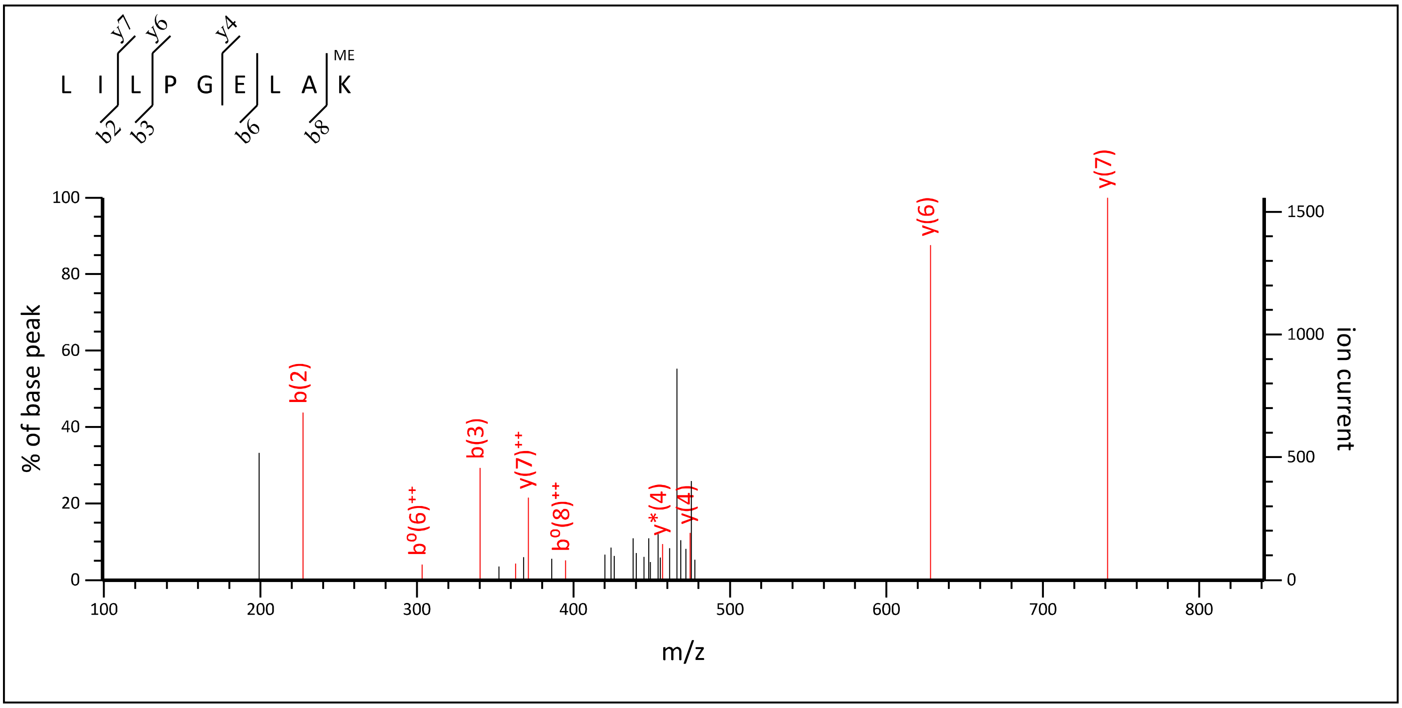

99.11 to 841.41

Label all possible matches ◉ Label matches used for scoring ○

Monoisotopic mass of neutral peptide Mr(calc): 966.6113
Fixed modifications: Carbamidomethyl (C) (apply to specified residues or termini only)
Variable modifications:
K9 : Methyl (K)
Ions Score: 20 **Expect:** 0.026 (help)

#	b	b^{++}	b^0	b^{0++}	Seq.	y	y^{++}	y^*	y^{*++}	y^0	y^{0++}	#
1	114.0913	57.5493			L							9
2	**227.1754**	114.0913			I	854.5346	427.7709	837.5080	419.2577	836.5240	418.7656	8
3	**340.2595**	170.6334			L	**741.4505**	**371.2289**	724.4240	**362.7156**	723.4400	362.2236	7
4	437.3122	219.1598			P	**628.3665**	314.6869	611.3399	306.1736	610.3559	305.6816	6
5	494.3337	247.6705			G	531.3137	266.1605	514.2871	257.6472	513.3031	257.1552	5
6	623.3763	312.1918	605.3657	**303.1865**	E	**474.2922**	237.6498	**457.2657**	229.1365	456.2817	228.6445	4
7	736.4604	368.7338	718.4498	359.7285	L	345.2496	173.1285	328.2231	164.6152			3
8	807.4975	404.2524	789.4869	**395.2471**	A	232.1656	116.5864	215.1390	108.0731			2
9					K	161.1285	81.0679	144.1019	72.5546			1

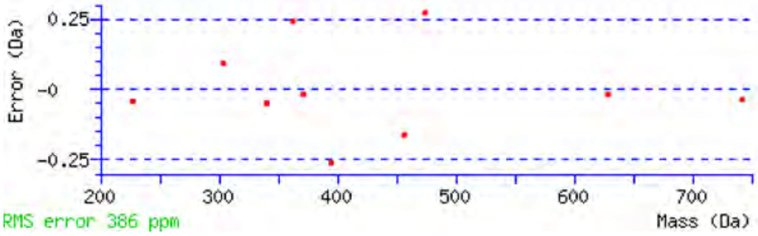

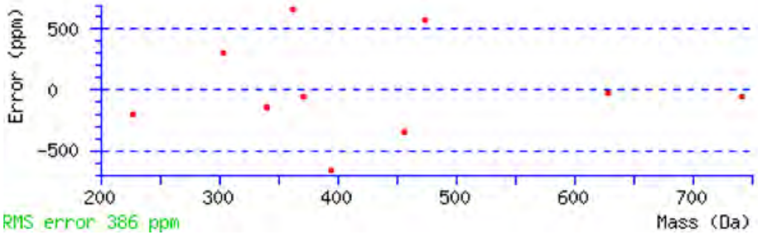

NCBI **BLAST** search of LILPGELAK
(Parameters: blastp, nr protein database, expect=20000, no filter, PAM30)
Other BLAST web gateways

All matches to this query

Score	Mr(calc)	Delta	Sequence
20.3	966.6113	0.0002	LILPGELAK
19.1	966.6113	0.0003	EPLPSLKK
19.1	966.6113	0.0002	EPLPSLKK
19.1	966.6113	0.0002	EPLPSLKK
19.1	966.6113	0.0002	ILINDPIK
19.1	966.6113	0.0002	LIITPIQK
3.3	966.6113	0.0003	PIEIILNK
2.6	966.6113	0.0002	IIGIPKDK
2.6	966.6113	0.0002	IIGIPKDK
1.9	966.6087	0.0029	GGKRGRPK

Mascot: http://www.matrixscience.com/

Mascot Search Results

Peptide View

MS/MS Fragmentation of **LILPGELAK**
Found in **H2B2_YEAST** in **S_cerevisiae_D**, sp|P02294|H2B2_YEAST Histone H2B.2 OS=Saccharomyces cerevisiae (strain ATCC 204508 / S288c) GN=HTB2 PE=1 SV=2

Match to Query 45234: 980.627228 from(491.320890,2+) intensity(24935.5640) scans(10840) rawscans(sn10840) rtinseconds(2825.3616) index(341195)
Title: 9529: Scan 10840 (rt=47.0894) [D:\MSData\All\VELOS23984.raw]
Data file D:\Data\MGF\530 Final H2A H2B yeast classical PTMs\mascot_daemon_merge.mgf

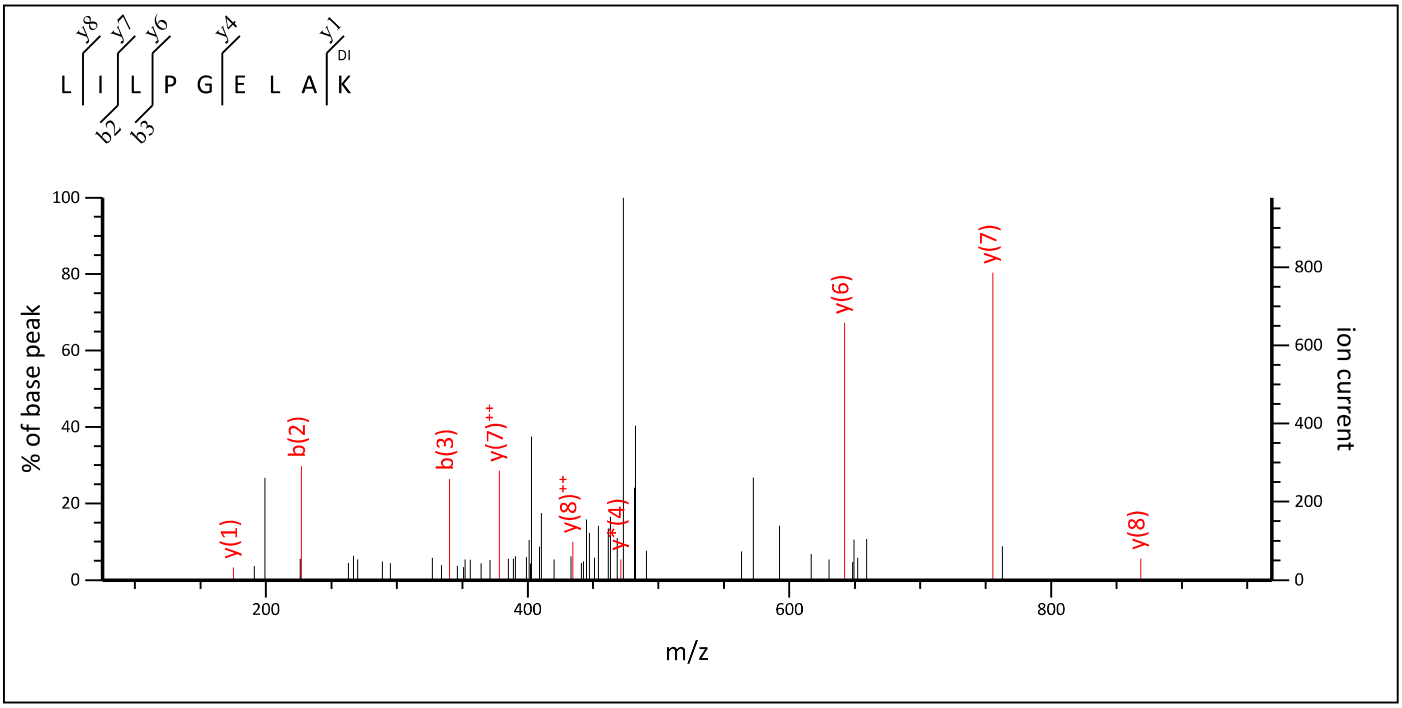

75.21 to 968.6

Label all possible matches Label matches used for scoring

Monoisotopic mass of neutral peptide Mr(calc): 980.6270
Fixed modifications: Carbamidomethyl (C) (apply to specified residues or termini only)
Variable modifications:
K9 : Dimethyl (K)
Ions Score: 26 **Expect:** 0.066 (help)

#	b	b^{++}	b^{0}	b^{0++}	Seq.	y	y^{++}	y^{*}	y^{*++}	y^{0}	y^{0++}	#
1	114.0913	57.5493			L							9
2	**227.1754**	114.0913			I	**868.5502**	**434.7788**	851.5237	426.2655	850.5397	425.7735	8
3	**340.2595**	170.6334			L	**755.4662**	**378.2367**	738.4396	369.7234	737.4556	369.2314	7
4	437.3122	219.1598			P	**642.3821**	321.6947	625.3556	313.1814	624.3715	312.6894	6
5	494.3337	247.6705			G	545.3293	273.1683	528.3028	264.6550	527.3188	264.1630	5
6	623.3763	312.1918	605.3657	303.1865	E	488.3079	244.6576	**471.2813**	236.1443	470.2973	235.6523	4
7	736.4604	368.7338	718.4498	359.7285	L	359.2653	180.1363	342.2387	171.6230			3
8	807.4975	404.2524	789.4869	395.2471	A	246.1812	123.5942	229.1547	115.0810			2
9					K	**175.1441**	88.0757	158.1176	79.5624			1

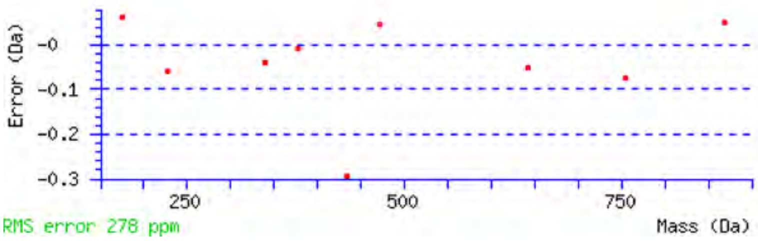

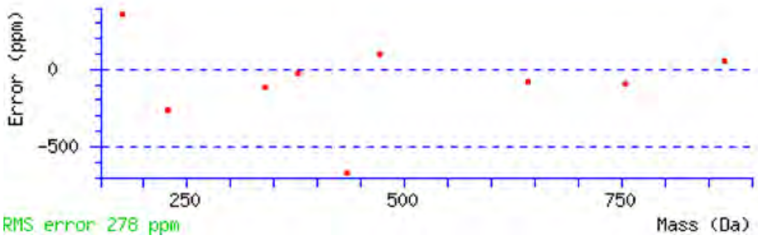

NCBI **BLAST** search of LILPGELAK
(Parameters: blastp, nr protein database, expect=20000, no filter, PAM30)
Other BLAST web gateways

All matches to this query

Score	Mr(calc)	Delta	Sequence
26.5	980.6270	0.0002	LILPGELAK
14.4	980.6270	0.0002	EPLPSLKK
14.4	980.6270	0.0002	EPLPSLKK
7.9	980.6270	0.0002	LLNELPLK
3.7	980.6270	0.0002	LLTPLPLSK
1.2	980.6243	0.0029	RGKQPKR
0.9	980.6270	0.0002	PIEIILNK
0.7	980.6270	0.0002	ILPLDLQK
0.2	980.6270	0.0002	IPETPVKK
0.2	980.6270	0.0002	IPETPVKK

Mascot: http://www.matrixscience.com/

Mascot Search Results

Peptide View

MS/MS Fragmentation of **HAVSEGTRAVTKYSSSTQA**
Found in **H2B2_YEAST** in **S_cerevisiae_D**, sp|P02294|H2B2_YEAST Histone H2B.2 OS=Saccharomyces cerevisiae (strain ATCC 204508 / S288c) GN=HTB2 PE=1 SV=2

Match to Query 241325: 2058.935562 from(687.319130,3+) intensity(36024.4840) scans(1976) rawscans(sn1976) rtinseconds(948.7934) index(157340)
Title: 1572: Scan 1976 (rt=15.8132) [D:\MSData\All\VELOS23670.raw]
Data file D:\Data\MGF\530 Final H2A H2B yeast classical PTMs\mascot_daemon_merge.mgf

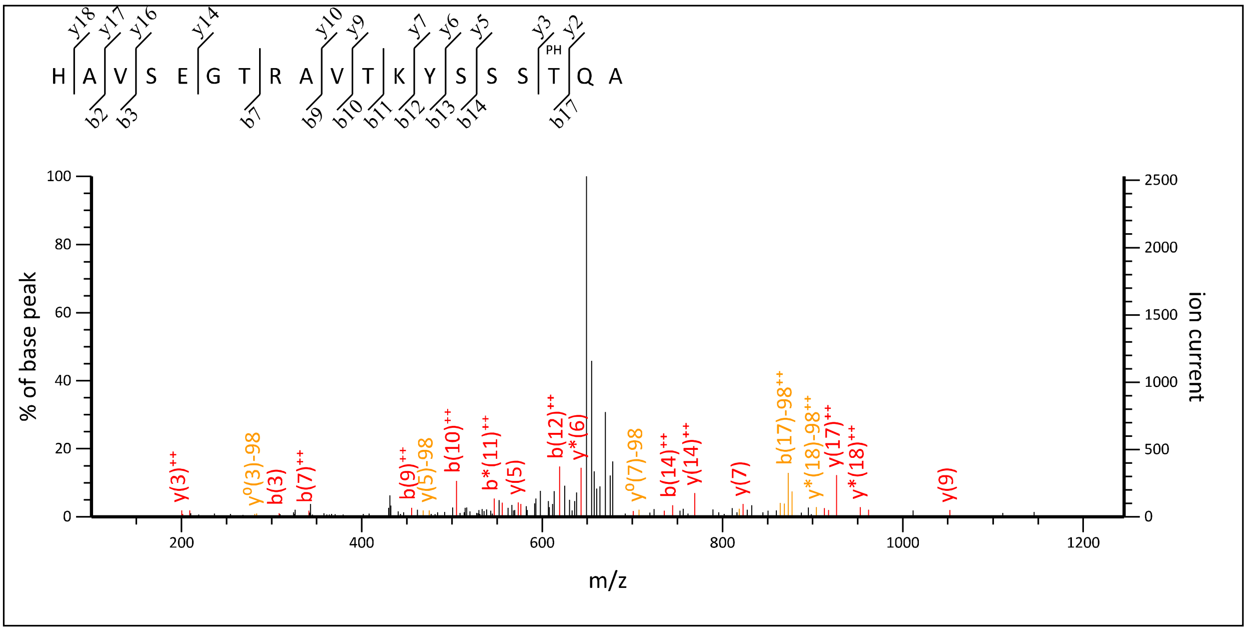

100.28 to 1245.54

Label all possible matches ○ Label matches used for scoring ◉

```
Monoisotopic mass of neutral peptide Mr(calc): 2058.9371
Fixed modifications: Carbamidomethyl (C) (apply to specified residues or termini only)
Variable modifications:
T17    : Phospho (ST), with neutral losses 0.0000(shown in table), 97.9769
Ions Score: 25  Expect: 0.036
Matches : 51/298 fragment ions using 73 most intense peaks   (help)
```

#	b	b++	b*	b*++	b0	b0++	Seq.	y	y++	y*	y*++	y0	y0++	#
1	138.0662	69.5367					H							19
2	209.1033	105.0553					A	1922.8855	961.9464	1905.8590	953.4331	1904.8750	952.9411	18
3	308.1717	154.5895					V	1851.8484	926.4278	1834.8219	917.9146	1833.8379	917.4226	17
4	395.2037	198.1055			377.1932	189.1002	S	1752.7800	876.8936	1735.7535	868.3804	1734.7694	867.8884	16
5	524.2463	262.6268			506.2358	253.6215	E	1665.7480	833.3776	1648.7214	824.8644	1647.7374	824.3723	15
6	581.2678	291.1375			563.2572	282.1323	G	1536.7054	768.8563	1519.6788	760.3431	1518.6948	759.8510	14
7	682.3155	341.6614			664.3049	332.6561	T	1479.6839	740.3456	1462.6574	731.8323	1461.6734	731.3403	13
8	838.4166	419.7119	821.3900	411.1987	820.4060	410.7067	R	1378.6362	689.8218	1361.6097	681.3085	1360.6257	680.8165	12
9	909.4537	455.2305	892.4272	446.7172	891.4431	446.2252	A	1222.5351	611.7712	1205.5086	603.2579	1204.5246	602.7659	11
10	1008.5221	504.7647	991.4956	496.2514	990.5116	495.7594	V	1151.4980	576.2526	1134.4715	567.7394	1133.4874	567.2474	10
11	1109.5698	555.2885	1092.5432	546.7753	1091.5592	546.2833	T	1052.4296	526.7184	1035.4030	518.2052	1034.4190	517.7132	9
12	1237.6648	619.3360	1220.6382	610.8227	1219.6542	610.3307	K	951.3819	476.1946	934.3554	467.6813	933.3714	467.1893	8
13	1400.7281	700.8677	1383.7015	692.3544	1382.7175	691.8624	Y	823.2870	412.1471	806.2604	403.6338	805.2764	403.1418	7
14	1487.7601	744.3837	1470.7336	735.8704	1469.7496	735.3784	S	660.2236	330.6155	643.1971	322.1022	642.2131	321.6102	6
15	1574.7921	787.8997	1557.7656	779.3864	1556.7816	778.8944	S	573.1916	287.0994	556.1651	278.5862	555.1810	278.0942	5
16	1661.8242	831.4157	1644.7976	822.9025	1643.8136	822.4104	S	486.1596	243.5834	469.1330	235.0701	468.1490	234.5781	4
17	1842.8382	921.9227	1825.8116	913.4095	1824.8276	912.9174	T	399.1275	200.0674	382.1010	191.5541	381.1170	191.0621	3
18	1970.8968	985.9520	1953.8702	977.4387	1952.8862	976.9467	Q	218.1135	109.5604	201.0870	101.0471			2

19							A	90.0550	45.5311					1

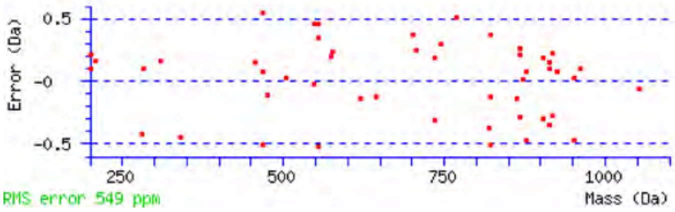

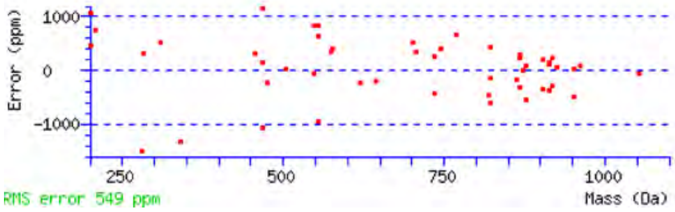

NCBI **BLAST** search of HAVSEGTRAVTKYSSSTQA
(Parameters: blastp, nr protein database, expect=20000, no filter, PAM30)
Other BLAST web gateways

All matches to this query

Score	Mr(calc)	Delta	Sequence	Site Analysis
24.7	2058.9371	-0.0016	HAVSEGTRAVTKYSSSTQA	Phospho T17 51.24%
22.3	2058.9371	-0.0016	HAVSEGTRAVTKYSSSTQA	Phospho S15 29.35%
19.6	2058.9371	-0.0016	HAVSEGTRAVTKYSSSTQA	Phospho S16 15.73%
9.7	2058.9371	-0.0016	HAVSEGTRAVTKYSSSTQA	Phospho S14 1.64%
8.8	2058.9346	0.0010	STRFIPKPLYGSNQK	
8.3	2058.9388	-0.0032	PQDASSSSLSSSPPKIRK	
8.3	2058.9388	-0.0032	PQDASSSSLSSSPPKIRK	
6.9	2058.9388	-0.0032	KGKKSKHSSDEGDKSK	
6.9	2058.9388	-0.0032	KGKKSKHSSDEGDKSK	
6.9	2058.9388	-0.0032	KGKKSKHSSDEGDKSK	

Mascot: http://www.matrixscience.com/

MATRIX SCIENCE Mascot Search Results

Peptide View

MS/MS Fragmentation of **AVTKYSSSTQA**
Found in **H2B2_YEAST** in **S_cerevisiae_D**, sp|P02294|H2B2_YEAST Histone H2B.2 OS=Saccharomyces cerevisiae (strain ATCC 204508 / S288c) GN=HTB2 PE=1 SV=2

Match to Query 84530: 1183.571928 from(592.793240,2+) intensity(45193.7070) scans(2700) rawscans(sn2700) rtinseconds(1120.6672) index(188209)
Title: 2246: Scan 2700 (rt=18.6778) [D:\MSData\All\VELOS23956.raw]
Data file D:\Data\MGF\530 Final H2A H2B yeast classical PTMs\mascot_daemon_merge.mgf

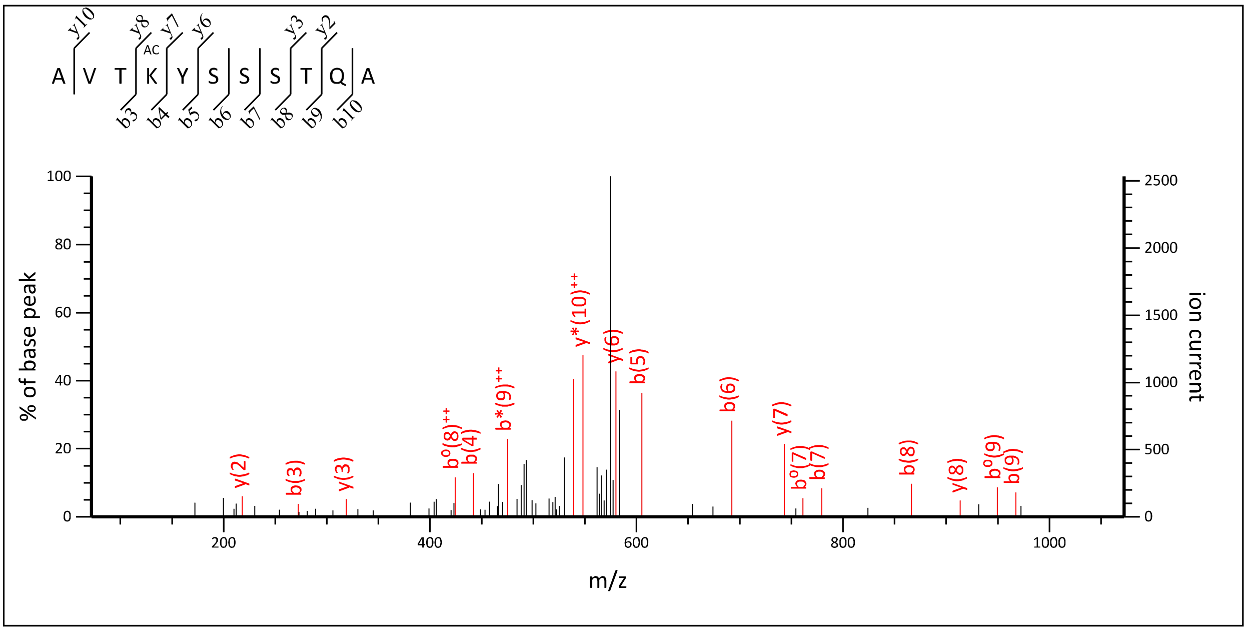

72.19 to 1072.51

Label all possible matches ○ Label matches used for scoring ◉

Monoisotopic mass of neutral peptide Mr(calc): 1183.5721
Fixed modifications: Carbamidomethyl (C) (apply to specified residues or termini only)
Variable modifications:
K4 : Acetyl (K)
Ions Score: 57 **Expect:** 1.1e-05
Matches : 23/104 fragment ions using 25 most intense peaks (help)

#	b	b^{++}	b*	b^{*++}	b^0	b^{0++}	Seq.	y	y^{++}	y*	y^{*++}	y^0	y^{0++}	#
1	72.0444	36.5258					A							11
2	171.1128	86.0600					V	1113.5422	557.2748	1096.5157	548.7615	1095.5317	548.2695	10
3	***272.1605***	136.5839			254.1499	127.5786	T	1014.4738	507.7406	997.4473	499.2273	996.4633	498.7353	9
4	***442.2660***	221.6366	425.2395	213.1234	424.2554	212.6314	K	**913.4262**	457.2167	896.3996	448.7034	895.4156	448.2114	8
5	***605.3293***	303.1683	588.3028	294.6550	587.3188	294.1630	Y	**743.3206**	372.1640	726.2941	363.6507	725.3101	363.1587	7
6	***692.3614***	346.6843	675.3348	338.1710	674.3508	337.6790	S	**580.2573**	290.6323	563.2307	282.1190	562.2467	281.6270	6
7	***779.3934***	390.2003	762.3668	381.6871	761.3828	381.1951	S	493.2253	247.1163	476.1987	238.6030	475.2147	238.1110	5
8	***866.4254***	433.7163	849.3989	425.2031	848.4149	424.7111	S	406.1932	203.6003	389.1667	195.0870	388.1827	194.5950	4
9	***967.4731***	484.2402	950.4466	475.7269	949.4625	475.2349	T	**319.1612**	160.0842	302.1347	151.5710	301.1506	151.0790	3
10	1095.5317	548.2695	1078.5051	539.7562	1077.5211	539.2642	Q	**218.1135**	109.5604	201.0870	101.0471			2
11							A	90.0550	45.5311					1

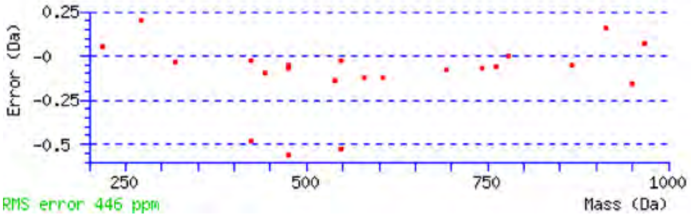

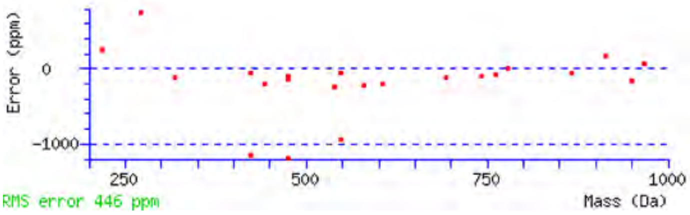

NCBI **BLAST** search of AVTKYSSSTQA
(Parameters: blastp, nr protein database, expect=20000, no filter, PAM30)
Other BLAST web gateways

All matches to this query

Score	Mr(calc)	Delta	Sequence
56.8	1183.5721	-0.0001	AVTKYSSSTQA
3.6	1183.5751	-0.0031	KTFEPRTR
1.9	1183.5750	-0.0031	KTFEPRTR
1.7	1183.5710	0.0009	RSSRTNGDK
0.9	1183.5750	-0.0031	KTFEPRTR

Mascot: http://www.matrixscience.com/

MATRIX SCIENCE Mascot Search Results

Peptide View

MS/MS Fragmentation of **AVTKYSSSTQA**
Found in **H2B2_YEAST** in **S_cerevisiae_D**, sp|P02294|H2B2_YEAST Histone H2B.2 OS=Saccharomyces cerevisiae (strain ATCC 204508 / S288c) GN=HTB2 PE=1 SV=2

Match to Query 90998: 1221.527328 from(611.770940,2+) intensity(63813.9770) scans(1817) rawscans(sn1817) rtinseconds(907.3459) index(258984)
Title: 1465: Scan 1817 (rt=15.1224) [D:\MSData\All\VELOS23664.raw]
Data file D:\Data\MGF\530 Final H2A H2B yeast classical PTMs\mascot_daemon_merge.mgf

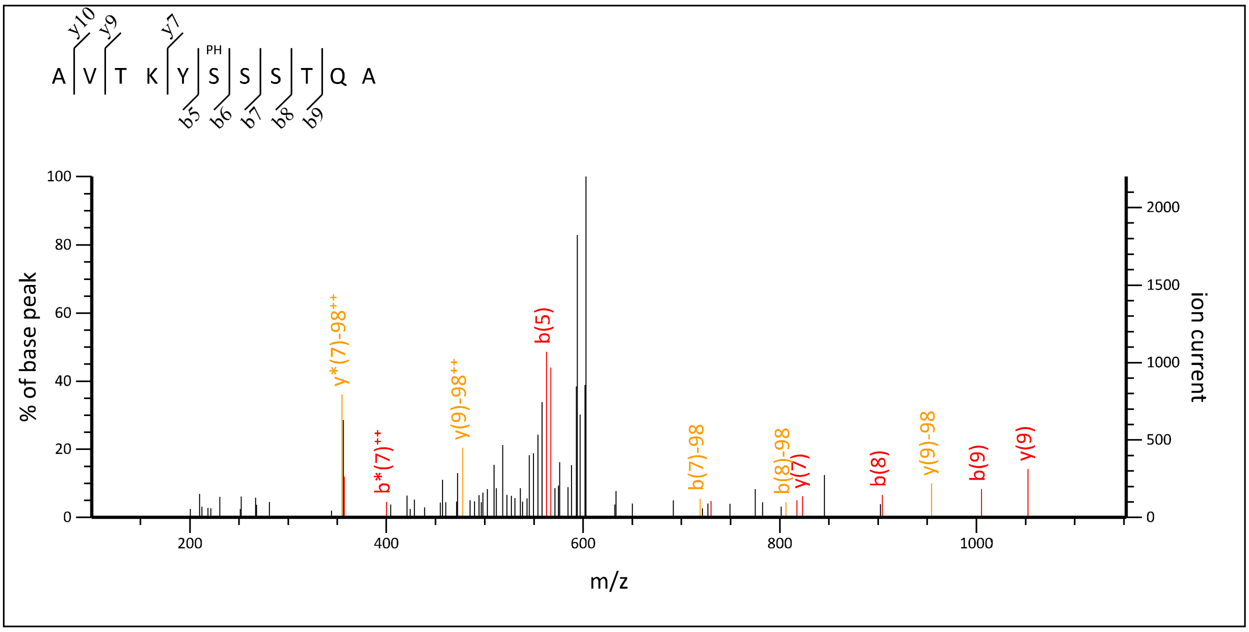

100.26 to 1152.38

Label all possible matches ○ Label matches used for scoring ◉

Monoisotopic mass of neutral peptide Mr(calc): 1221.5278
Fixed modifications: Carbamidomethyl (C) (apply to specified residues or termini only)
Variable modifications:
S6 : Phospho (ST), with neutral losses 0.0000(shown in table), 97.9769
Ions Score: 28 **Expect:** 0.0046
Matches : 21/164 fragment ions using 33 most intense peaks (help)

#	b	b^{++}	b*	b^{*++}	b^0	b^{0++}	Seq.	y	y^{++}	y*	y^{*++}	y^0	y^{0++}	#
1	72.0444	36.5258					A							11
2	171.1128	86.0600					V	1151.4980	576.2526	1134.4715	567.7394	1133.4874	567.2474	10
3	272.1605	136.5839			254.1499	127.5786	T	1052.4296	526.7184	1035.4030	518.2052	1034.4190	517.7132	9
4	400.2554	200.6314	383.2289	192.1181	382.2449	191.6261	K	951.3819	476.1946	934.3554	467.6813	933.3714	467.1893	8
5	563.3188	282.1630	546.2922	273.6498	545.3082	273.1577	Y	823.2870	412.1471	806.2604	403.6338	805.2764	403.1418	7
6	730.3171	365.6622	713.2906	357.1489	712.3066	356.6569	S	660.2236	330.6155	643.1971	322.1022	642.2131	321.6102	6
7	817.3492	409.1782	800.3226	400.6649	799.3386	400.1729	S	493.2253	247.1163	476.1987	238.6030	475.2147	238.1110	5
8	904.3812	452.6942	887.3546	444.1810	886.3706	443.6890	S	406.1932	203.6003	389.1667	195.0870	388.1827	194.5950	4
9	1005.4289	503.2181	988.4023	494.7048	987.4183	494.2128	T	319.1612	160.0842	302.1347	151.5710	301.1506	151.0790	3
10	1133.4874	567.2474	1116.4609	558.7341	1115.4769	558.2421	Q	218.1135	109.5604	201.0870	101.0471			2
11							A	90.0550	45.5311					1

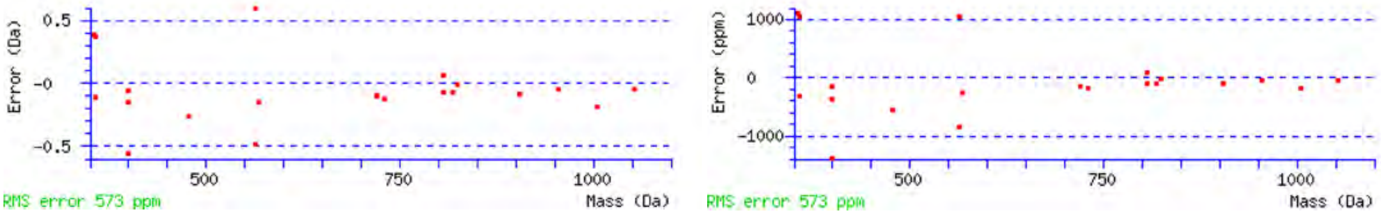

NCBI **BLAST** search of AVTKYSSSTQA
(Parameters: blastp, nr protein database, expect=20000, no filter, PAM30)
Other BLAST web gateways

All matches to this query

Score	Mr(calc)	Delta	Sequence	Site Analysis
28.1	1221.5278	-0.0005	AVTKYSSSTQA	Phospho S6 73.83%
22.5	1221.5278	-0.0005	AVTKYSSSTQA	Phospho S7 20.38%
15.3	1221.5278	-0.0005	AVTKYSSSTQA	Phospho S8 3.88%
10.1	1221.5278	-0.0005	AVTKYSSSTQA	Phospho T9 1.16%
8.2	1221.5278	-0.0005	AVTKYSSSTQA	Phospho T3 0.75%
3.0	1221.5229	0.0044	LSKINSQMK	
1.2	1221.5319	-0.0045	DGELFYGLSK	
1.1	1221.5251	0.0022	GRSNDAHTSR	
1.0	1221.5325	-0.0052	KRQHSSCK	
1.0	1221.5325	-0.0052	KRQHSSCK	

Mascot: http://www.matrixscience.com/

MATRIX SCIENCE Mascot Search Results

Peptide View

MS/MS Fragmentation of **KSTGGKAPR**
Found in **H3_YEAST** in **S_cerevisiae_D**, sp|P61830|H3_YEAST Histone H3 OS=Saccharomyces cerevisiae (strain ATCC 204508 / S288c) GN=HHT1 PE=1 SV=2

Match to Query 15297: 984.534408 from(493.274480,2+) intensity(15473.0830) scans(914) rawscans(sn914) rtinseconds(633.2113) index(110881)
Title: 669: Scan 914 (rt=10.5535) [D:\MSData\All\VELOS25245.raw]
Data file D:\Data\MGF\533 Final H3 yeast classical PTMs\mascot_daemon_merge.mgf

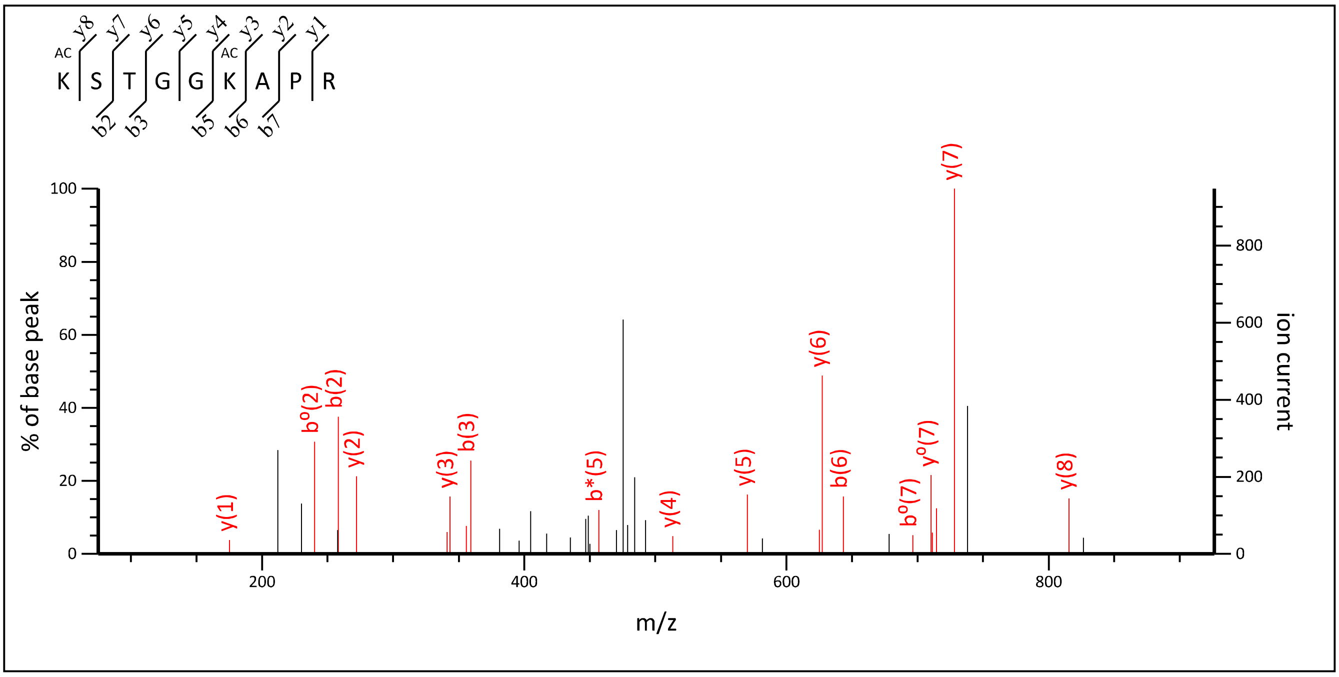

75.14 to 926.38

Label all possible matches ○ Label matches used for scoring ◉

Monoisotopic mass of neutral peptide Mr(calc): 984.5352
Fixed modifications: Carbamidomethyl (C) (apply to specified residues or termini only)
Variable modifications:
K1 : Acetyl (K)
K6 : Acetyl (K)
Ions Score: 63 **Expect:** 1.7e-05
Matches : 22/82 fragment ions using 37 most intense peaks (help)

#	b	b^{++}	b*	b^{*++}	b^0	b^{0++}	Seq.	y	y^{++}	y*	y^{*++}	y^0	y^{0++}	#
1	171.1128	86.0600	154.0863	77.5468			K							9
2	**258.1448**	129.5761	241.1183	121.0628	240.1343	120.5708	S	***815.4370***	408.2221	798.4104	399.7089	797.4264	399.2169	8
3	**359.1925**	180.0999	342.1660	171.5866	341.1819	171.0946	T	***728.4050***	364.7061	711.3784	356.1928	710.3944	355.7008	7
4	416.2140	208.6106	399.1874	200.0974	398.2034	199.6053	G	***627.3573***	314.1823	610.3307	305.6690			6
5	473.2354	237.1214	456.2089	228.6081	455.2249	228.1161	G	***570.3358***	285.6715	553.3093	277.1583			5
6	**643.3410**	322.1741	626.3144	313.6608	625.3304	313.1688	K	***513.3144***	257.1608	496.2878	248.6475			4
7	**714.3781**	357.6927	697.3515	349.1794	696.3675	348.6874	A	***343.2088***	172.1081	326.1823	163.5948			3
8	811.4308	406.2191	794.4043	397.7058	793.4203	397.2138	P	***272.1717***	136.5895	255.1452	128.0762			2
9							R	***175.1190***	88.0631	158.0924	79.5498			1

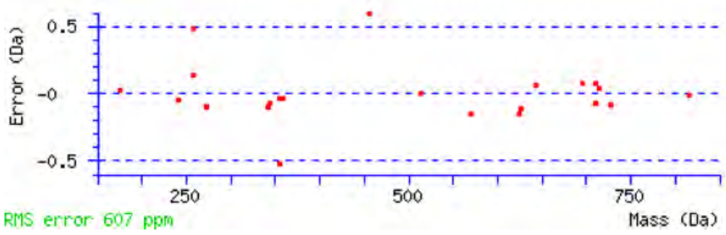

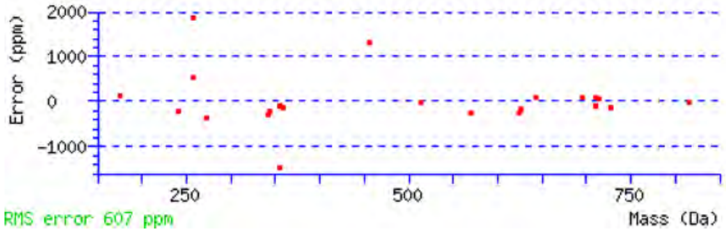

NCBI **BLAST** search of KSTGGKAPR
(Parameters: blastp, nr protein database, expect=20000, no filter, PAM30)
Other BLAST web gateways

All matches to this query

Score	Mr(calc)	Delta	Sequence
63.2	984.5352	-0.0008	KSTGGKAPR
33.4	984.5369	-0.0025	KSTTSRK
33.4	984.5369	-0.0025	KSTTSRK
33.4	984.5369	-0.0025	KSTTSRK
33.4	984.5369	-0.0025	KSTTSRK
33.4	984.5369	-0.0025	RSTTSKK
33.4	984.5369	-0.0025	RSTTSKK
22.3	984.5369	-0.0025	KSTKSGKK
22.3	984.5369	-0.0025	KSTTSRK
22.3	984.5369	-0.0025	KSTTSRK

Mascot: http://www.matrixscience.com/

MATRIX SCIENCE Mascot Search Results

Peptide View

MS/MS Fragmentation of **KSAPSTGGVK**
Found in **H3_YEAST** in **S_cerevisiae_D**, sp|P61830|H3_YEAST Histone H3 OS=Saccharomyces cerevisiae (strain ATCC 204508 / S288c) GN=HHT1 PE=1 SV=2

Match to Query 21007: 1052.489568 from(527.252060,2+) intensity(14674.1880) scans(1718) rawscans(sn1718) rtinseconds(887.8376) index(87783)
Title: 1329: Scan 1718 (rt=14.7973) [D:\MSData\All\VELOS25241.raw]
Data file D:\Data\MGF\533 Final H3 yeast classical PTMs\mascot_daemon_merge.mgf

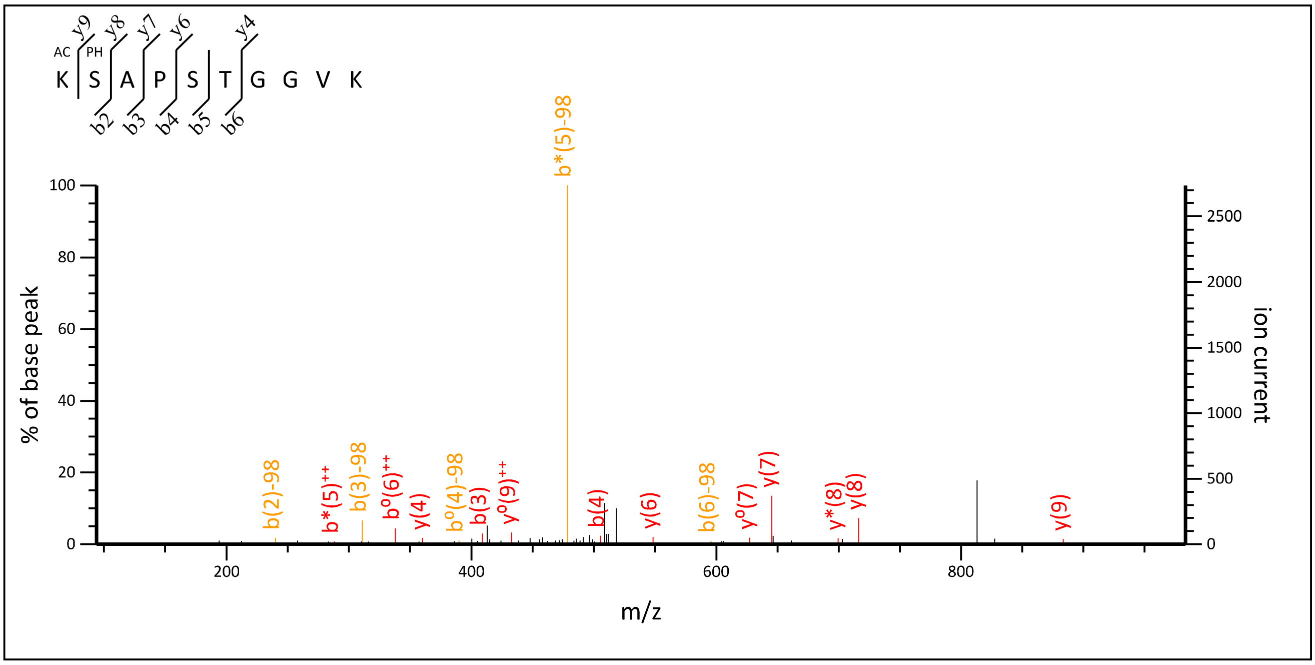

93.97 to 983.48

Label all possible matches ○ Label matches used for scoring ●

Monoisotopic mass of neutral peptide Mr(calc): 1052.4903
Fixed modifications: Carbamidomethyl (C) (apply to specified residues or termini only)
Variable modifications:
K1 : Acetyl (K)
S2 : Phospho (ST), with neutral losses 0.0000(shown in table), 97.9769
Ions Score: 23 **Expect:** 0.14
Matches : 20/152 fragment ions using 40 most intense peaks (help)

#	b	b^{++}	b^*	b^{*++}	b^0	b^{0++}	Seq.	y	y^{++}	y^*	y^{*++}	y^0	y^{0++}	#
1	171.1128	86.0600	154.0863	77.5468			K							10
2	**338.1112**	169.5592	321.0846	161.0459	320.1006	160.5539	S	***883.3921***	442.1997	866.3655	433.6864	865.3815	433.1944	9
3	**409.1483**	205.0778	392.1217	196.5645	391.1377	196.0725	A	***716.3937***	358.7005	699.3672	350.1872	698.3832	349.6952	8
4	**506.2010**	253.6042	489.1745	245.0909	488.1905	244.5989	P	***645.3566***	323.1819	628.3301	314.6687	627.3461	314.1767	7
5	593.2331	297.1202	576.2065	288.6069	575.2225	288.1149	S	***548.3039***	274.6556	531.2773	266.1423	530.2933	265.6503	6
6	694.2807	347.6440	677.2542	339.1307	676.2702	338.6387	T	461.2718	231.1396	444.2453	222.6263	443.2613	222.1343	5
7	751.3022	376.1547	734.2757	367.6415	733.2916	367.1495	G	***360.2241***	180.6157	343.1976	172.1024			4
8	808.3237	404.6655	791.2971	396.1522	790.3131	395.6602	G	303.2027	152.1050	286.1761	143.5917			3
9	907.3921	454.1997	890.3655	445.6864	889.3815	445.1944	V	246.1812	123.5942	229.1547	115.0810			2
10							K	147.1128	74.0600	130.0863	65.5468			1

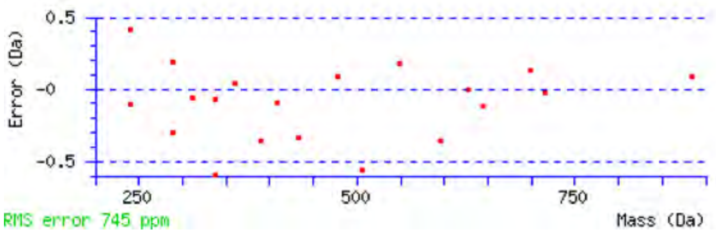

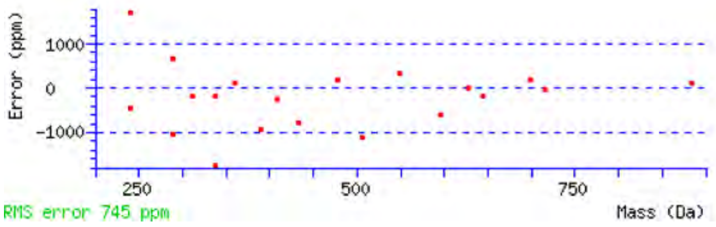

NCBI **BLAST** search of KSAPSTGGVK
(Parameters: blastp, nr protein database, expect=20000, no filter, PAM30)
Other BLAST web gateways

All matches to this query

Score	Mr(calc)	Delta	Sequence
22.8	1052.4903	-0.0008	KSAPSTGGVK
12.8	1052.4943	-0.0048	KTAYSSFK
11.3	1052.4903	-0.0008	LNTTPSVNK
8.8	1052.4903	-0.0007	KSEEKLR
8.8	1052.4903	-0.0007	KSGDVELR
8.6	1052.4903	-0.0007	KSKGENVK
7.7	1052.4903	-0.0007	KSVANKDK
6.6	1052.4903	-0.0008	LNTTPSVNK
4.9	1052.4903	-0.0007	QQALEAVSK
4.9	1052.4903	-0.0007	AVSRIEEK

Mascot: http://www.matrixscience.com/

MATRIX SCIENCE Mascot Search Results

Peptide View

MS/MS Fragmentation of **KQLASKAAR**
Found in **H3_YEAST** in **S_cerevisiae_D**, sp|P61830|H3_YEAST Histone H3 OS=Saccharomyces cerevisiae (strain ATCC 204508 / S288c) GN=HHT1 PE=1 SV=2

Match to Query 21307: 1055.607988 from(528.811270,2+) intensity(175468.8800) scans(2294) rawscans(sn2294) rtinseconds(985.9898) index(32175)
Title: 1869: Scan 2294 (rt=16.4332) [D:\MSData\All\VELOS25233.raw]
Data file D:\Data\MGF\533 Final H3 yeast classical PTMs\mascot_daemon_merge.mgf

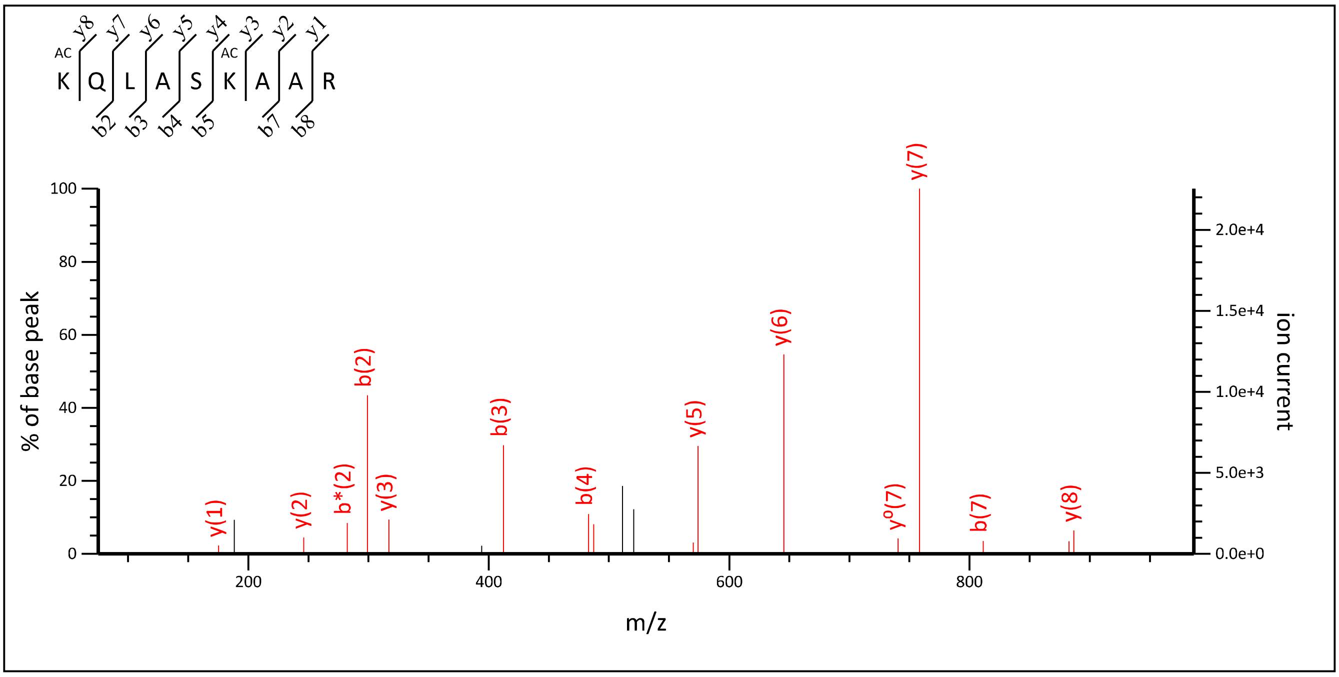

75.26 to 986.62

Label all possible matches ○ Label matches used for scoring ◉

Monoisotopic mass of neutral peptide Mr(calc): 1055.6087
Fixed modifications: Carbamidomethyl (C) (apply to specified residues or termini only)
Variable modifications:
K1 : Acetyl (K)
K6 : Acetyl (K)
Ions Score: 85 **Expect:** 2.1e-07
Matches : 17/80 fragment ions using 20 most intense peaks (help)

#	b	b^{++}	b*	b^{*++}	b^{0}	b^{0++}	Seq.	y	y^{++}	y*	y^{*++}	y^{0}	y^{0++}	#
1	171.1128	86.0600	154.0863	77.5468			K							9
2	**299.1714**	150.0893	282.1448	141.5761			Q	***886.5105***	443.7589	869.4839	435.2456	868.4999	434.7536	8
3	**412.2554**	206.6314	395.2289	198.1181			L	***758.4519***	379.7296	741.4254	371.2163	740.4413	370.7243	7
4	**483.2926**	242.1499	466.2660	233.6366			A	***645.3679***	323.1876	628.3413	314.6743	627.3573	314.1823	6
5	**570.3246**	285.6659	553.2980	277.1527	552.3140	276.6606	S	***574.3307***	287.6690	557.3042	279.1557	556.3202	278.6637	5
6	**740.4301**	370.7187	723.4036	362.2054	722.4196	361.7134	K	***487.2987***	244.1530	470.2722	235.6397			4
7	**811.4672**	406.2373	794.4407	397.7240	793.4567	397.2320	A	***317.1932***	159.1002	300.1666	150.5870			3
8	**882.5043**	441.7558	865.4778	433.2425	864.4938	432.7505	A	***246.1561***	123.5817	229.1295	115.0684			2
9							R	***175.1190***	88.0631	158.0924	79.5498			1

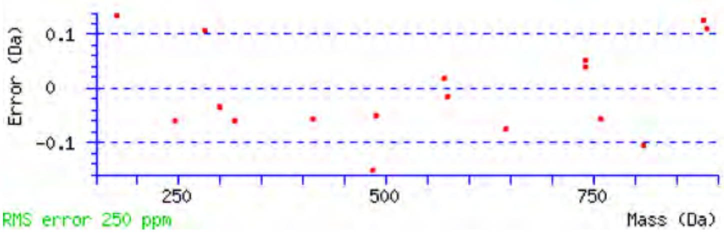

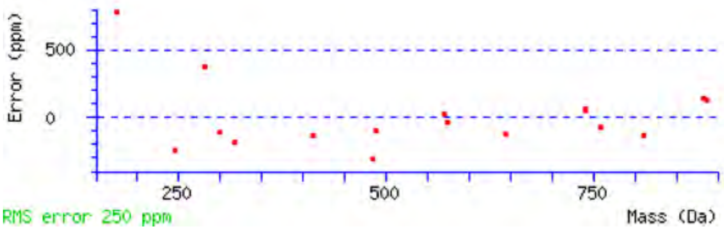

NCBI **BLAST** search of KQLASKAAR
(Parameters: blastp, nr protein database, expect=20000, no filter, PAM30)
Other BLAST web gateways

All matches to this query

Score	Mr(calc)	Delta	Sequence
85.3	1055.6087	-0.0007	KQLASKAAR
27.1	1055.6087	-0.0007	AKGLAARGDK
23.9	1055.6087	-0.0007	AVQERNLAK
22.6	1055.6087	-0.0007	QKEKAKAR
22.4	1055.6087	-0.0007	KQEKAAKR
20.5	1055.6087	-0.0007	KQEKAAKR
19.9	1055.6087	-0.0007	KRAAQEKK
19.9	1055.6087	-0.0007	KRAAQEKK
18.9	1055.6087	-0.0007	AKGLAARGDK
18.9	1055.6087	-0.0007	AKGLAARGDK

Mascot: http://www.matrixscience.com/

MATRIX SCIENCE Mascot Search Results

Peptide View

MS/MS Fragmentation of **KSAPSTGGVK**
Found in **H3_YEAST** in **S_cerevisiae_D**, sp|P61830|H3_YEAST Histone H3 OS=Saccharomyces cerevisiae (strain ATCC 204508 / S288c) GN=HHT1 PE=1 SV=2

Match to Query 14742: 972.523888 from(487.269220,2+) intensity(12132.8630) scans(1363) rawscans(sn1363) rtinseconds(720.2363) index(46363)
Title: 1051: Scan 1363 (rt=12.0039) [D:\MSData\All\VELOS25235.raw]
Data file D:\Data\MGF\533 Final H3 yeast classical PTMs\mascot_daemon_merge.mgf

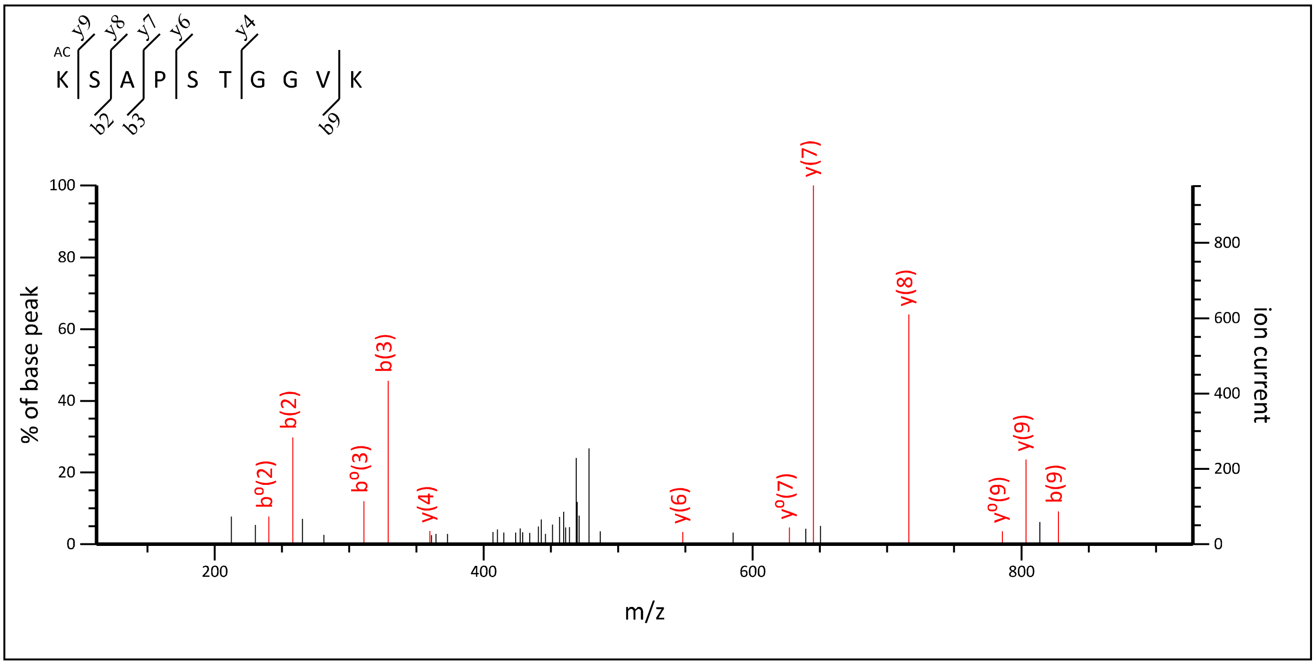

112.22 to 927.43

Label all possible matches ○ Label matches used for scoring ◉

Monoisotopic mass of neutral peptide Mr(calc): 972.5240
Fixed modifications: Carbamidomethyl (C) (apply to specified residues or termini only)
Variable modifications:
K1 : Acetyl (K)
Ions Score: 40 **Expect:** 0.0074
Matches : 12/98 fragment ions using 19 most intense peaks (help)

#	b	b^{++}	b*	b^{*++}	b^0	b^{0++}	Seq.	y	y^{++}	y*	y^{*++}	y^0	y^{0++}	#
1	171.1128	86.0600	154.0863	77.5468			K							10
2	**258.1448**	129.5761	241.1183	121.0628	240.1343	120.5708	S	***803.4258***	402.2165	786.3992	393.7032	785.4152	393.2112	9
3	**329.1819**	165.0946	312.1554	156.5813	311.1714	156.0893	A	***716.3937***	358.7005	699.3672	350.1872	698.3832	349.6952	8
4	426.2347	213.6210	409.2082	205.1077	408.2241	204.6157	P	***645.3566***	323.1819	628.3301	314.6687	627.3461	314.1767	7
5	513.2667	257.1370	496.2402	248.6237	495.2562	248.1317	S	***548.3039***	274.6556	531.2773	266.1423	530.2933	265.6503	6
6	614.3144	307.6608	597.2879	299.1476	596.3039	298.6556	T	461.2718	231.1396	444.2453	222.6263	443.2613	222.1343	5
7	671.3359	336.1716	654.3093	327.6583	653.3253	327.1663	G	***360.2241***	180.6157	343.1976	172.1024			4
8	728.3573	364.6823	711.3308	356.1690	710.3468	355.6770	G	303.2027	152.1050	286.1761	143.5917			3
9	**827.4258**	414.2165	810.3992	405.7032	809.4152	405.2112	V	246.1812	123.5942	229.1547	115.0810			2
10							K	147.1128	74.0600	130.0863	65.5468			1

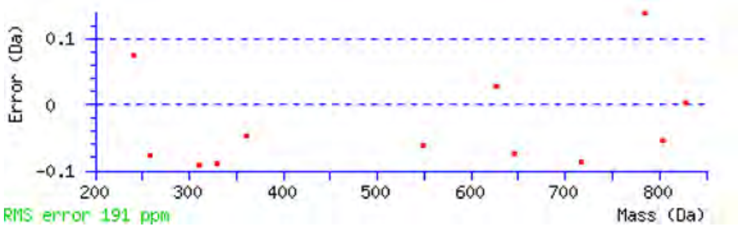

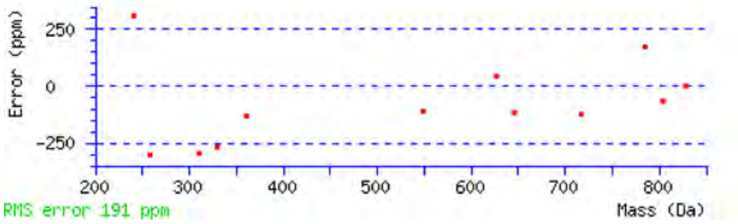

NCBI **BLAST** search of KSAPSTGGVK
(Parameters: blastp, nr protein database, expect=20000, no filter, PAM30)
Other BLAST web gateways

All matches to this query

Score	Mr(calc)	Delta	Sequence
39.6	972.5240	-0.0001	KSAPSTGGVK
15.9	972.5280	-0.0041	KTAYSSFK
14.0	972.5240	-0.0001	KSKGENVK
10.2	972.5240	-0.0001	VTGNEKSPK
10.2	972.5240	-0.0001	KSKEAQAK
9.3	972.5240	-0.0001	KSKEAQAK
8.5	972.5240	-0.0001	VDNKKASK
8.5	972.5240	-0.0001	VDNKKASK
8.4	972.5240	-0.0001	KSDKGGKK
8.2	972.5240	-0.0001	KSVANKDK

Mascot: http://www.matrixscience.com/

MATRIX SCIENCE Mascot Search Results

Peptide View

MS/MS Fragmentation of **KSAPSTGGVKKPHR**
Found in **H3_YEAST** in **S_cerevisiae_D**, sp|P61830|H3_YEAST Histone H3 OS=Saccharomyces cerevisiae (strain ATCC 204508 / S288c) GN=HHT1 PE=1 SV=2

Match to Query 60884: 1532.842362 from(511.954730,3+) intensity(8879.4229) scans(1235) rawscans(sn1235) rtinseconds(734.2615) index(937)
Title: 938: Scan 1235 (rt=12.2377) [D:\MSData\All\VELOS25229.raw]
Data file D:\Data\MGF\533 Final H3 yeast classical PTMs\mascot_daemon_merge.mgf

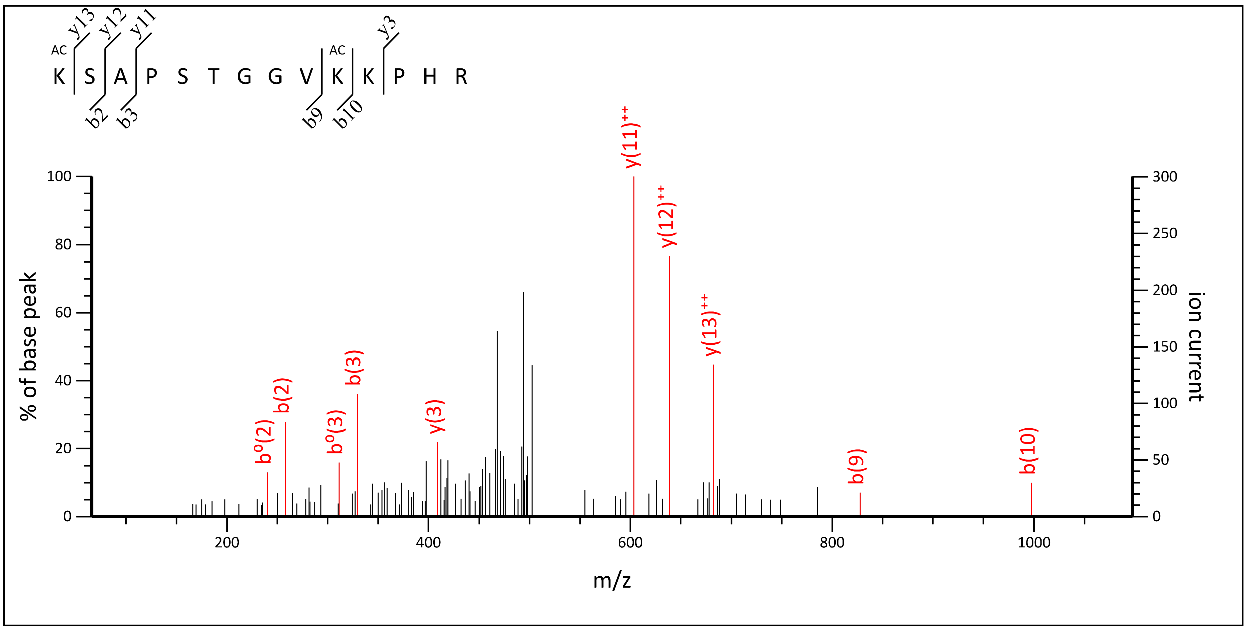

66.04 to 1097.63

Label all possible matches ○ Label matches used for scoring ◉

```
Monoisotopic mass of neutral peptide Mr(calc): 1532.8423
Fixed modifications: Carbamidomethyl (C) (apply to specified residues or termini only)
Variable modifications:
K1     : Acetyl (K)
K10    : Acetyl (K)
Ions Score: 22  Expect: 0.11
Matches : 13/138 fragment ions using 15 most intense peaks   (help)
```

#	b	b^{++}	b*	b^{*++}	b^0	b^{0++}	Seq.	y	y^{++}	y*	y^{*++}	y^0	y^{0++}	#
1	171.1128	86.0600	154.0863	77.5468			K							14
2	***258.1448***	129.5761	241.1183	121.0628	240.1343	120.5708	S	1363.7441	***682.3757***	1346.7175	673.8624	1345.7335	673.3704	13
3	***329.1819***	165.0946	312.1554	156.5813	311.1714	156.0893	A	1276.7120	***638.8597***	1259.6855	630.3464	1258.7015	629.8544	12
4	426.2347	213.6210	409.2082	205.1077	408.2241	204.6157	P	1205.6749	***603.3411***	1188.6484	594.8278	1187.6644	594.3358	11
5	513.2667	257.1370	496.2402	248.6237	495.2562	248.1317	S	1108.6222	554.8147	1091.5956	546.3014	1090.6116	545.8094	10
6	614.3144	307.6608	597.2879	299.1476	596.3039	298.6556	T	1021.5901	511.2987	1004.5636	502.7854	1003.5796	502.2934	9
7	671.3359	336.1716	654.3093	327.6583	653.3253	327.1663	G	920.5425	460.7749	903.5159	452.2616			8
8	728.3573	364.6823	711.3308	356.1690	710.3468	355.6770	G	863.5210	432.2641	846.4944	423.7509			7
9	***827.4258***	414.2165	810.3992	405.7032	809.4152	405.2112	V	806.4995	403.7534	789.4730	395.2401			6
10	***997.5313***	499.2693	980.5047	490.7560	979.5207	490.2640	K	707.4311	354.2192	690.4046	345.7059			5
11	1125.6263	563.3168	1108.5997	554.8035	1107.6157	554.3115	K	537.3256	269.1664	520.2990	260.6532			4
12	1222.6790	611.8431	1205.6525	603.3299	1204.6684	602.8379	P	409.2306	205.1190	392.2041	196.6057			3
13	1359.7379	680.3726	1342.7114	671.8593	1341.7274	671.3673	H	312.1779	156.5926	295.1513	148.0793			2
14							R	175.1190	88.0631	158.0924	79.5498			1

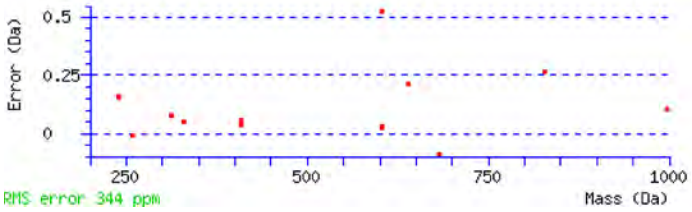

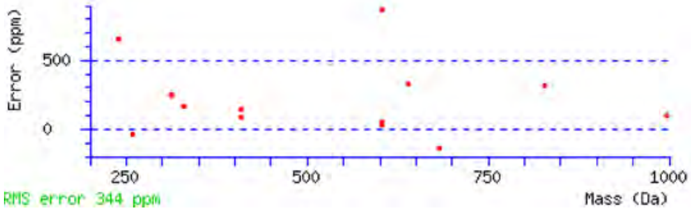

NCBI **BLAST** search of KSAPSTGGVKKPHR
(Parameters: blastp, nr protein database, expect=20000, no filter, PAM30)
Other BLAST web gateways

All matches to this query

Score	Mr(calc)	Delta	Sequence
22.2	1532.8423	0.0001	KSAPSTGGVKKPHR
14.8	1532.8423	0.0001	KSAPSTGGVKKPHR
11.2	1532.8384	0.0039	KLVYGICRKEK
8.7	1532.8351	0.0073	GLSKFVWEGKANK
8.7	1532.8351	0.0073	GLSKFVWEGKANK
8.6	1532.8450	-0.0026	KSAITKNLLDDFV
8.4	1532.8418	0.0006	LESAKLCKEIMR
7.6	1532.8384	0.0039	TPMAFKEINSRK
7.6	1532.8384	0.0039	TPMAFKEINSRK
7.4	1532.8423	0.0001	SRAKSNASFKGLR

Mascot: http://www.matrixscience.com/

Mascot Search Results

Peptide View

MS/MS Fragmentation of **KSAPSTGGVKKPHR**
Found in **H3_YEAST** in **S_cerevisiae_D**, sp|P61830|H3_YEAST Histone H3 OS=Saccharomyces cerevisiae (strain ATCC 204508 / S288c) GN=HHT1 PE=1 SV=2

Match to Query 60896: 1532.877672 from(511.966500,3+) intensity(9376.7949) scans(739) rawscans(sn739) rtinseconds(587.6734) index(137312)
Title: 525: Scan 739 (rt=9.79456) [D:\MSData\All\VELOS25249.raw]
Data file D:\Data\MGF\533 Final H3 yeast classical PTMs\mascot_daemon_merge.mgf

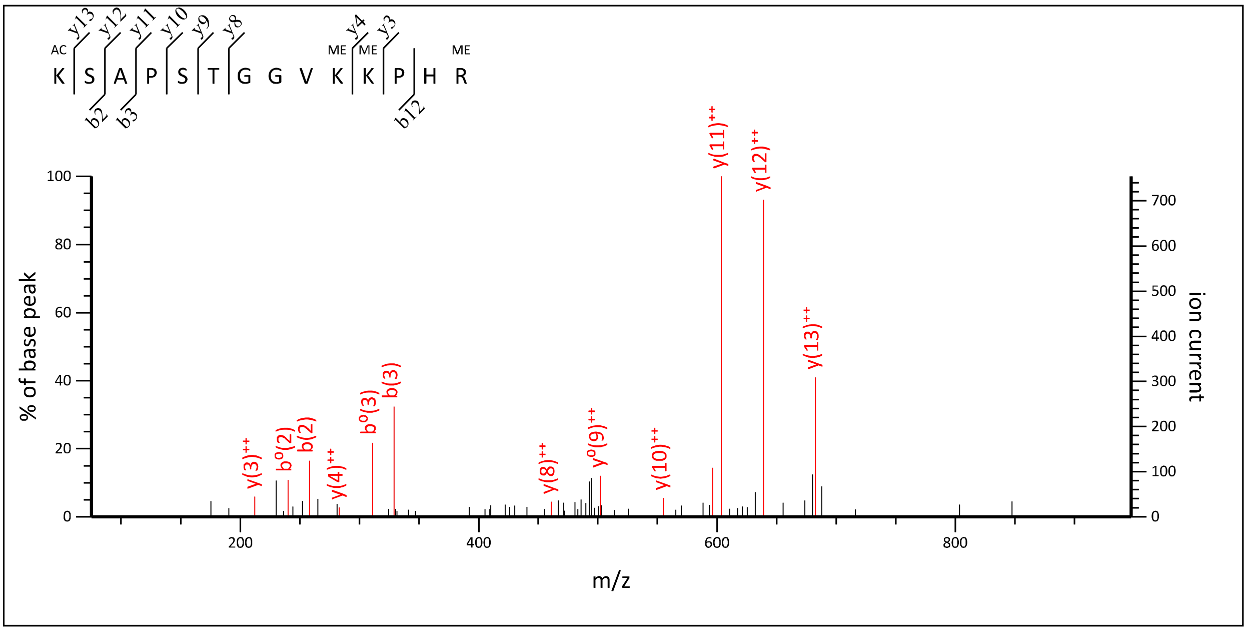

75.17 to 947.45

Label all possible matches Label matches used for scoring

Monoisotopic mass of neutral peptide Mr(calc): 1532.8787
Fixed modifications: Carbamidomethyl (C) (apply to specified residues or termini only)
Variable modifications:
K1 : Acetyl (K)
K10 : Methyl (K)
K11 : Methyl (K)
R14 : Methyl (R)
Ions Score: 37 **Expect:** 0.023
Matches : 14/138 fragment ions using 26 most intense peaks (help)

#	b	b^{++}	b*	b^{*++}	b^0	b^{0++}	Seq.	y	y^{++}	y*	y^{*++}	y^0	y^{0++}	#
1	171.1128	86.0600	154.0863	77.5468			K							14
2	**258.1448**	129.5761	241.1183	121.0628	240.1343	120.5708	S	1363.7805	***682.3939***	1346.7539	673.8806	1345.7699	673.3886	13
3	**329.1819**	165.0946	312.1554	156.5813	311.1714	156.0893	A	1276.7484	***638.8779***	1259.7219	630.3646	1258.7379	629.8726	12
4	426.2347	213.6210	409.2082	205.1077	408.2241	204.6157	P	1205.7113	***603.3593***	1188.6848	594.8460	1187.7008	594.3540	11
5	513.2667	257.1370	496.2402	248.6237	495.2562	248.1317	S	1108.6586	***554.8329***	1091.6320	546.3196	1090.6480	545.8276	10
6	614.3144	307.6608	597.2879	299.1476	596.3039	298.6556	T	1021.6265	511.3169	1004.6000	502.8036	1003.6160	502.3116	9
7	671.3359	336.1716	654.3093	327.6583	653.3253	327.1663	G	920.5788	***460.7931***	903.5523	452.2798			8
8	728.3573	364.6823	711.3308	356.1690	710.3468	355.6770	G	863.5574	432.2823	846.5308	423.7691			7
9	827.4258	414.2165	810.3992	405.7032	809.4152	405.2112	V	806.5359	403.7716	789.5094	395.2583			6
10	969.5364	485.2718	952.5098	476.7585	951.5258	476.2665	K	707.4675	354.2374	690.4410	345.7241			5
11	1111.6470	556.3271	1094.6204	547.8139	1093.6364	547.3218	K	565.3569	***283.1821***	548.3303	274.6688			4
12	1208.6997	604.8535	1191.6732	596.3402	1190.6892	595.8482	P	423.2463	***212.1268***	406.2197	203.6135			3
13	1345.7587	673.3830	1328.7321	664.8697	1327.7481	664.3777	H	326.1935	163.6004	309.1670	155.0871			2
14							R	189.1346	95.0709	172.1081	86.5577			1

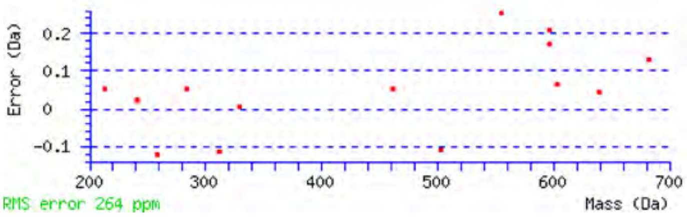

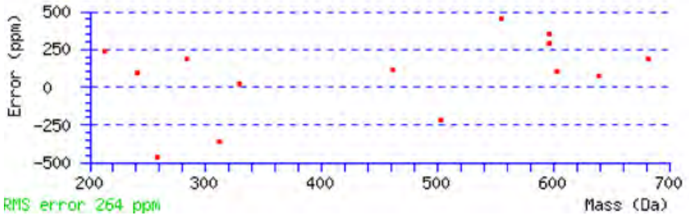

NCBI **BLAST** search of KSAPSTGGVKKPHR
(Parameters: blastp, nr protein database, expect=20000, no filter, PAM30)
Other BLAST web gateways

All matches to this query

Score	Mr(calc)	Delta	Sequence
37.1	1532.8787	-0.0010	KSAPSTGGVKKPHR
27.3	1532.8787	-0.0010	KSAPSTGGVKKPHR
27.2	1532.8787	-0.0010	KSAPSTGGVKKPHR
27.2	1532.8787	-0.0010	KSAPSTGGVKKPHR
27.1	1532.8787	-0.0010	KSAPSTGGVKKPHR
20.8	1532.8787	-0.0010	KSAPSTGGVKKPHR
20.8	1532.8787	-0.0010	KSAPSTGGVKKPHR
20.8	1532.8787	-0.0010	KSAPSTGGVKKPHR
20.8	1532.8787	-0.0010	KSAPSTGGVKKPHR
20.8	1532.8787	-0.0010	KSAPSTGGVKKPHR

Mascot: http://www.matrixscience.com/

MATRIX SCIENCE Mascot Search Results

Peptide View

MS/MS Fragmentation of **YKPGTVALR**
Found in **H3_YEAST** in **S_cerevisiae_D**, sp|P61830|H3_YEAST Histone H3 OS=Saccharomyces cerevisiae (strain ATCC 204508 / S288c) GN=HHT1 PE=1 SV=2

Match to Query 20267: 1045.592128 from(523.803340,2+) intensity(18360.2930) scans(4111) rawscans(sn4111) rtinseconds(1539.4178) index(89810)
Title: 3356: Scan 4111 (rt=25.657) [D:\MSData\All\VELOS25241.raw]
Data file D:\Data\MGF\533 Final H3 yeast classical PTMs\mascot_daemon_merge.mgf

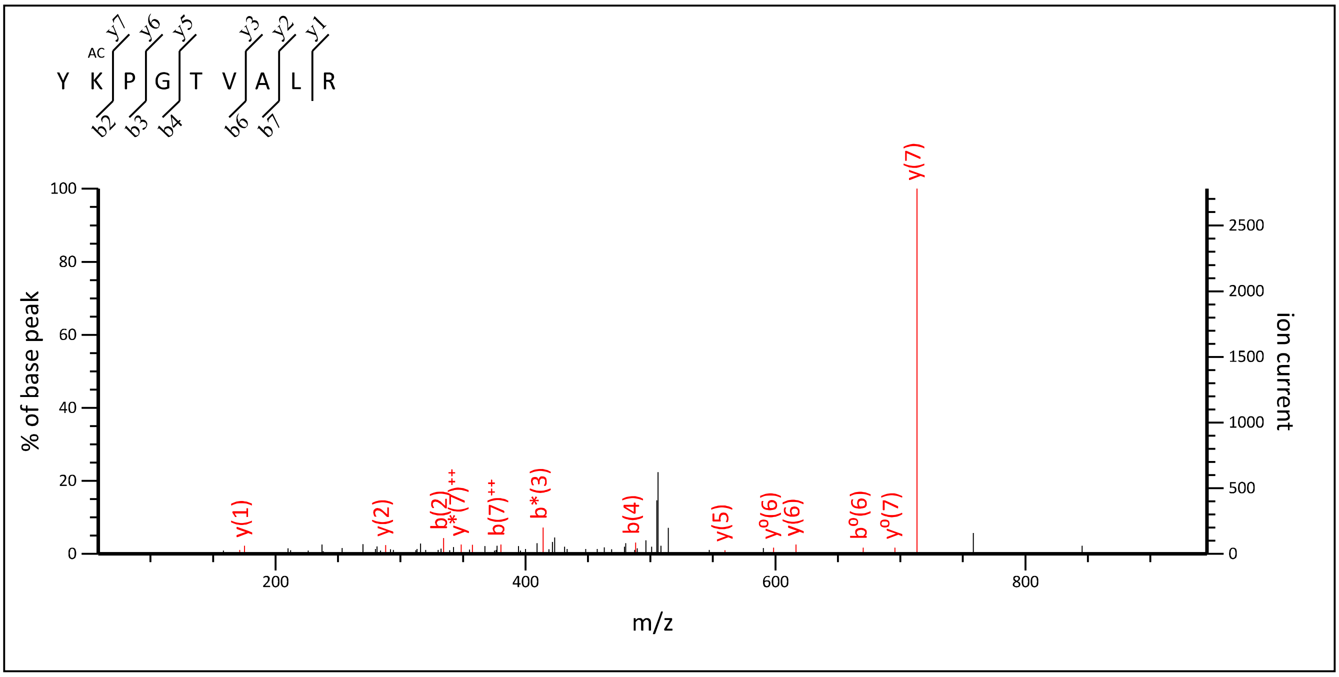

58.18 to 945.31

Label all possible matches ○ Label matches used for scoring ◉

Monoisotopic mass of neutral peptide Mr(calc): 1045.5920
Fixed modifications: Carbamidomethyl (C) (apply to specified residues or termini only)
Variable modifications:
K2 : Acetyl (K)
Ions Score: 23 **Expect:** 0.062
Matches : 16/78 fragment ions using 33 most intense peaks (help)

#	b	b^{++}	b*	b^{*++}	b^0	b^{0++}	Seq.	y	y^{++}	y*	y^{*++}	y^0	y^{0++}	#
1	164.0706	82.5389					Y							9
2	**334.1761**	167.5917	317.1496	159.0784			K	883.5360	442.2716	866.5094	433.7584	865.5254	433.2663	8
3	431.2289	216.1181	414.2023	207.6048			P	***713.4305***	357.2189	696.4039	348.7056	695.4199	348.2136	7
4	**488.2504**	244.6288	471.2238	236.1155			G	***616.3777***	308.6925	599.3511	300.1792	598.3671	299.6872	6
5	589.2980	295.1527	572.2715	286.6394	571.2875	286.1474	T	***559.3562***	280.1817	542.3297	271.6685	541.3457	271.1765	5
6	688.3665	344.6869	671.3399	336.1736	670.3559	335.6816	V	458.3085	229.6579	441.2820	221.1446			4
7	759.4036	380.2054	742.3770	371.6921	741.3930	371.2001	A	359.2401	180.1237	342.2136	171.6104			3
8	872.4876	436.7475	855.4611	428.2342	854.4771	427.7422	L	***288.2030***	144.6051	271.1765	136.0919			2
9							R	***175.1190***	88.0631	158.0924	79.5498			1

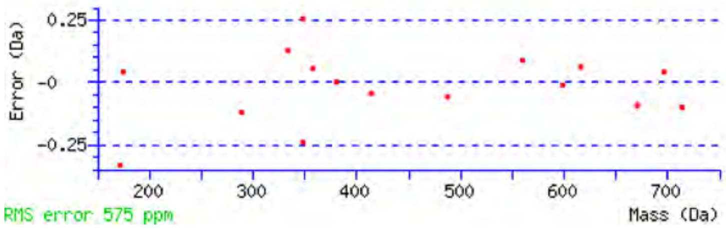

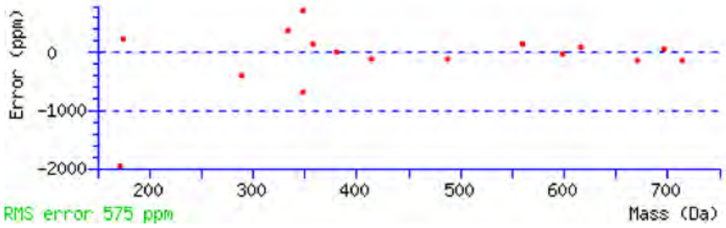

NCBI **BLAST** search of YKPGTVALR
(Parameters: blastp, nr protein database, expect=20000, no filter, PAM30)
Other BLAST web gateways

All matches to this query

Score	Mr(calc)	Delta	Sequence
23.0	1045.5920	0.0001	YKPGTVALR
9.5	1045.5936	-0.0015	YKKSSKK
9.5	1045.5936	-0.0015	YKKSSKK
8.7	1045.5880	0.0041	NRTKDDKK
7.8	1045.5920	0.0001	KYSRIAPK
5.7	1045.5920	0.0001	FADGKAVLR
5.2	1045.5880	0.0041	SSLRSPTSR
4.8	1045.5936	-0.0015	YKKSSKK
4.8	1045.5936	-0.0015	YKKSSKK
4.5	1045.5880	0.0042	NSKKRSSK

Mascot: http://www.matrixscience.com/

MATRIX SCIENCE Mascot Search Results

Peptide View

MS/MS Fragmentation of **YKPGTVALR**
Found in **H3_YEAST** in **S_cerevisiae_D**, sp|P61830|H3_YEAST Histone H3 OS=Saccharomyces cerevisiae (strain ATCC 204508 / S288c) GN=HHT1 PE=1 SV=2

Match to Query 23282: 1083.549768 from(542.782160,2+) intensity(24354.3070) scans(2978) rawscans(sn2978) rtinseconds(1191.549) index(126366)
Title: 2440: Scan 2978 (rt=19.8592) [D:\MSData\All\VELOS25247.raw]
Data file D:\Data\MGF\533 Final H3 yeast classical PTMs\mascot_daemon_merge.mgf

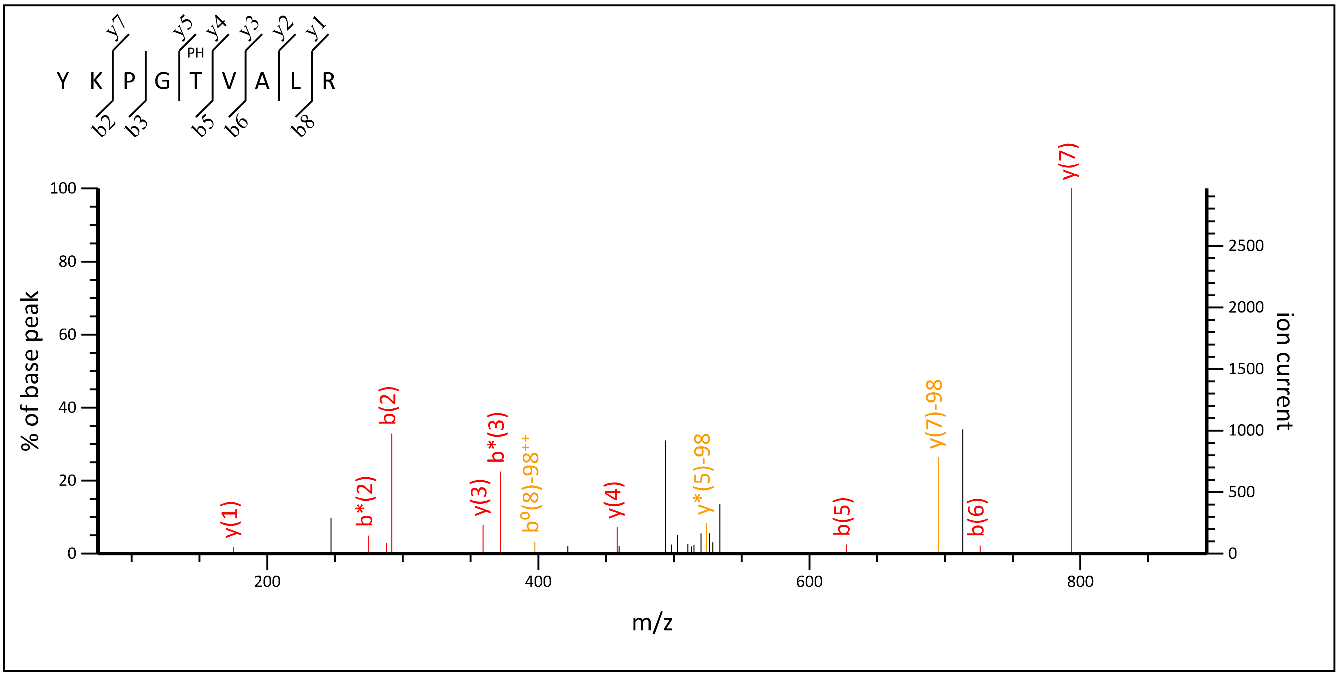

75.18 to 893.3

Label all possible matches ○ Label matches used for scoring ◉

Monoisotopic mass of neutral peptide Mr(calc): 1083.5478
Fixed modifications: Carbamidomethyl (C) (apply to specified residues or termini only)
Variable modifications:
T5 : Phospho (ST), with neutral losses 0.0000(shown in table), 97.9769
Ions Score: 34 **Expect:** 0.0098
Matches : 14/126 fragment ions using 20 most intense peaks (help)

#	b	b^{++}	b*	b^{*++}	b^0	b^{0++}	Seq.	y	y^{++}	y*	y^{*++}	y^0	y^{0++}	#
1	164.0706	82.5389					Y							9
2	**292.1656**	146.5864	275.1390	138.0731			K	921.4917	461.2495	904.4652	452.7362	903.4812	452.2442	8
3	389.2183	195.1128	372.1918	186.5995			P	***793.3968***	397.2020	776.3702	388.6888	775.3862	388.1967	7
4	446.2398	223.6235	429.2132	215.1103			G	696.3440	348.6756	679.3175	340.1624	678.3335	339.6704	6
5	**627.2538**	314.1305	610.2273	305.6173	609.2432	305.1253	T	639.3226	320.1649	622.2960	311.6516	621.3120	311.1596	5
6	**726.3222**	363.6647	709.2957	355.1515	708.3117	354.6595	V	***458.3085***	229.6579	441.2820	221.1446			4
7	797.3593	399.1833	780.3328	390.6700	779.3488	390.1780	A	***359.2401***	180.1237	342.2136	171.6104			3
8	910.4434	455.7253	893.4168	447.2121	892.4328	446.7201	L	***288.2030***	144.6051	271.1765	136.0919			2
9							R	***175.1190***	88.0631	158.0924	79.5498			1

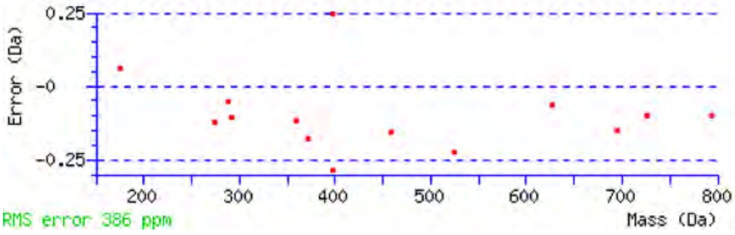

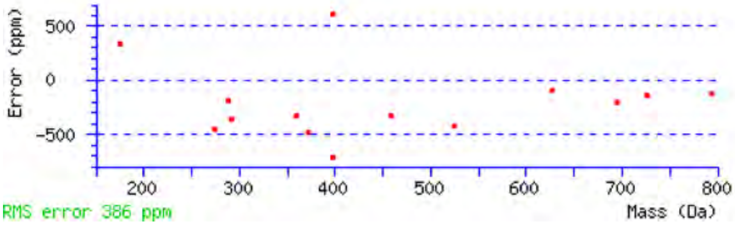

NCBI **BLAST** search of YKPGTVALR
(Parameters: blastp, nr protein database, expect=20000, no filter, PAM30)
Other BLAST web gateways

All matches to this query

Score	Mr(calc)	Delta	Sequence
33.9	1083.5478	0.0020	YKPGTVALR
11.7	1083.5511	-0.0013	MLSARSAIK
9.0	1083.5518	-0.0020	WPFKSALK
7.2	1083.5511	-0.0014	PRSSKSMK
6.5	1083.5511	-0.0014	KGMKSEIR
5.8	1083.5478	0.0020	FINTALLGR
5.8	1083.5550	-0.0052	RSSSSLRR
5.0	1083.5457	0.0041	YKMVDGVMK
4.6	1083.5511	-0.0013	MLSARSAIK
4.3	1083.5511	-0.0014	PRSSKSMK

Mascot: http://www.matrixscience.com/

Mascot Search Results

Peptide View

MS/MS Fragmentation of **FQKSTELLIR**
Found in **H3_YEAST** in **S_cerevisiae_D**, sp|P61830|H3_YEAST Histone H3 OS=Saccharomyces cerevisiae (strain ATCC 204508 / S288c) GN=HHT1 PE=1 SV=2

Match to Query 37347: 1275.718108 from(638.866330,2+) intensity(264040.9400) scans(7610) rawscans(sn7610) rtinseconds(2547.3091) index(106035)
Title: 6226: Scan 7610 (rt=42.4552) [D:\MSData\All\VELOS25243.raw]
Data file D:\Data\MGF\533 Final H3 yeast classical PTMs\mascot_daemon_merge.mgf

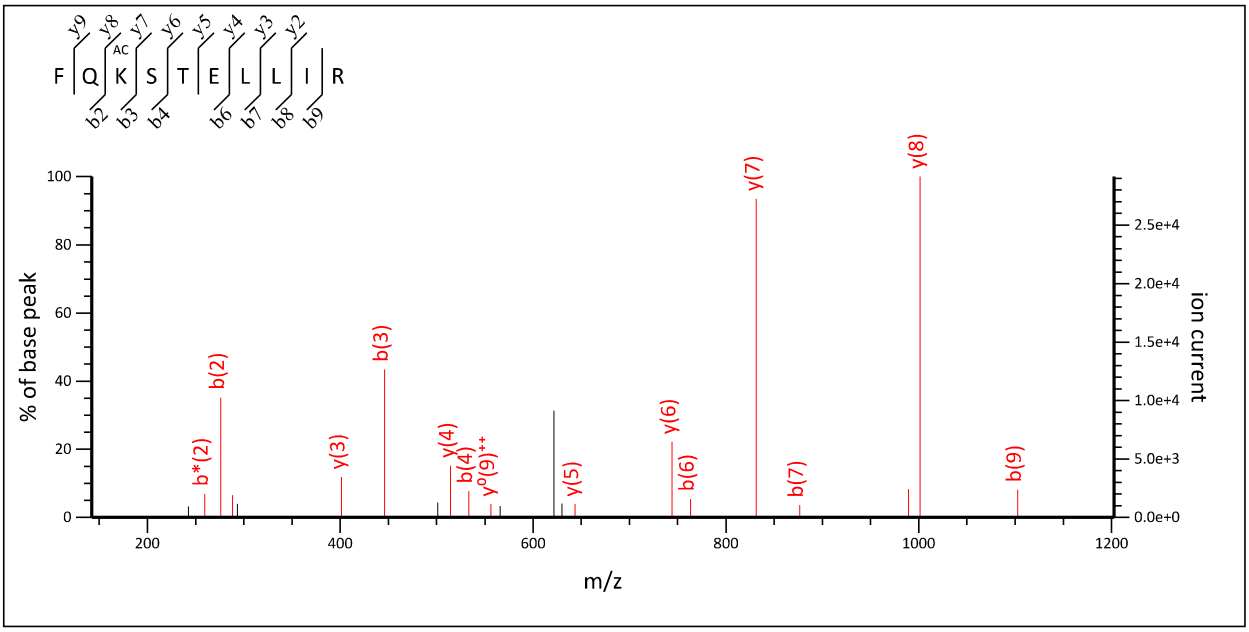

142.23 to 1202.55

Label all possible matches ○ Label matches used for scoring ●

```
Monoisotopic mass of neutral peptide Mr(calc): 1275.7187
Fixed modifications: Carbamidomethyl (C) (apply to specified residues or termini only)
Variable modifications:
K3     : Acetyl (K)
Ions Score: 86  Expect: 2.9e-07
Matches : 17/92 fragment ions using 18 most intense peaks   (help)
```

#	b	b^{++}	b*	b^{*++}	b^0	b^{0++}	Seq.	y	y^{++}	y*	y^{*++}	y^0	y^{0++}	#
1	148.0757	74.5415					F							10
2	***276.1343***	138.5708	259.1077	130.0575			Q	1129.6575	565.3324	1112.6310	556.8191	1111.6470	556.3271	9
3	***446.2398***	223.6235	429.2132	215.1103			K	***1001.5990***	501.3031	984.5724	492.7898	983.5884	492.2978	8
4	***533.2718***	267.1396	516.2453	258.6263	515.2613	258.1343	S	***831.4934***	416.2504	814.4669	407.7371	813.4829	407.2451	7
5	634.3195	317.6634	617.2930	309.1501	616.3089	308.6581	T	***744.4614***	372.7343	727.4349	364.2211	726.4509	363.7291	6
6	***763.3621***	382.1847	746.3355	373.6714	745.3515	373.1794	E	***643.4137***	322.2105	626.3872	313.6972	625.4032	313.2052	5
7	***876.4462***	438.7267	859.4196	430.2134	858.4356	429.7214	L	***514.3711***	257.6892	497.3446	249.1759			4
8	***989.5302***	495.2688	972.5037	486.7555	971.5197	486.2635	L	***401.2871***	201.1472	384.2605	192.6339			3
9	***1102.6143***	551.8108	1085.5877	543.2975	1084.6037	542.8055	I	***288.2030***	144.6051	271.1765	136.0919			2
10							R	175.1190	88.0631	158.0924	79.5498			1

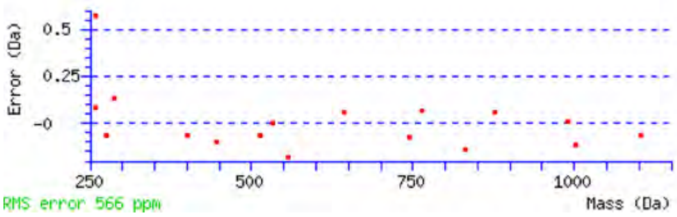

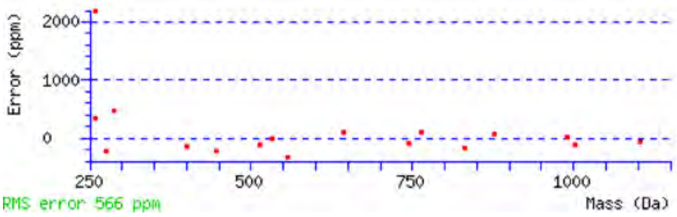

NCBI **BLAST** search of FQKSTELLIR
(Parameters: blastp, nr protein database, expect=20000, no filter, PAM30)
Other BLAST web gateways

All matches to this query

Score	Mr(calc)	Delta	Sequence
85.8	1275.7187	-0.0006	FQKSTELLIR
39.9	1275.7146	0.0035	LSSISQKSRSK
24.8	1275.7146	0.0035	SSLSNKTARSK
24.8	1275.7146	0.0035	SSLSNKTARSK
24.5	1275.7146	0.0035	SSERNKSKDK
21.4	1275.7146	0.0035	SSERNKSKDK
21.4	1275.7146	0.0035	SSERNKSKDK
21.2	1275.7186	-0.0005	SLSPKYLKTR
21.0	1275.7186	-0.0005	SSIKNWSDKK
19.6	1275.7187	-0.0006	SSIKNWSDKK

Mascot: http://www.matrixscience.com/

MATRIX SCIENCE Mascot Search Results

Peptide View

MS/MS Fragmentation of **FQKSTELLIR**
Found in **H3_YEAST** in **S_cerevisiae_D**, sp|P61830|H3_YEAST Histone H3 OS=Saccharomyces cerevisiae (strain ATCC 204508 / S288c) GN=HHT1 PE=1 SV=2

Match to Query 40885: 1313.675108 from(657.844830,2+) intensity(8211.1826) scans(5603) rawscans(sn5603) rtinseconds(1861.8676) index(34984)
Title: 4678: Scan 5603 (rt=31.0311) [D:\MSData\All\VELOS25233.raw]
Data file D:\Data\MGF\533 Final H3 yeast classical PTMs\mascot_daemon_merge.mgf

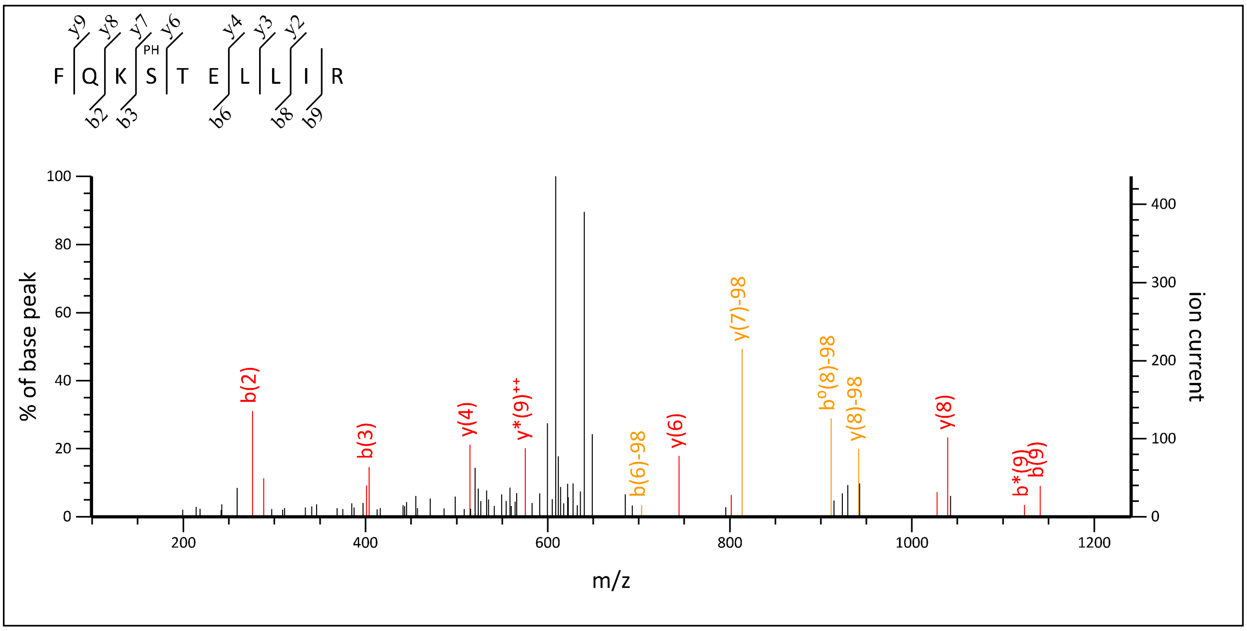

99.2 to 1240.54

Label all possible matches   Label matches used for scoring

```
Monoisotopic mass of neutral peptide Mr(calc): 1313.6744
Fixed modifications: Carbamidomethyl (C) (apply to specified residues or termini only)
Variable modifications:
S4     : Phospho (ST), with neutral losses 0.0000(shown in table), 97.9769
Ions Score: 53  Expect: 0.00047
Matches : 21/146 fragment ions using 20 most intense peaks   (help)
```

#	b	b^{++}	b*	b^{*++}	b^0	b^{0++}	Seq.	y	y^{++}	y*	y^{*++}	y^0	y^{0++}	#
1	148.0757	74.5415					F							10
2	**276.1343**	138.5708	259.1077	130.0575			Q	1167.6133	584.3103	1150.5868	575.7970	1149.6028	575.3050	9
3	**404.2292**	202.6183	387.2027	194.1050			K	***1039.5547***	520.2810	1022.5282	511.7677	1021.5442	511.2757	8
4	571.2276	286.1174	554.2010	277.6042	553.2170	277.1122	S	***911.4598***	456.2335	894.4332	447.7203	893.4492	447.2282	7
5	672.2753	336.6413	655.2487	328.1280	654.2647	327.6360	T	***744.4614***	372.7343	727.4349	364.2211	726.4509	363.7291	6
6	**801.3179**	**401.1626**	784.2913	392.6493	783.3073	392.1573	E	643.4137	322.2105	626.3872	313.6972	625.4032	313.2052	5
7	914.4019	457.7046	897.3754	449.1913	896.3914	448.6993	L	***514.3711***	257.6892	497.3446	249.1759			4
8	**1027.4860**	**514.2466**	1010.4594	505.7334	1009.4754	505.2414	L	***401.2871***	201.1472	384.2605	192.6339			3
9	**1140.5701**	570.7887	1123.5435	562.2754	1122.5595	561.7834	I	***288.2030***	144.6051	271.1765	136.0919			2
10							R	175.1190	88.0631	158.0924	79.5498			1

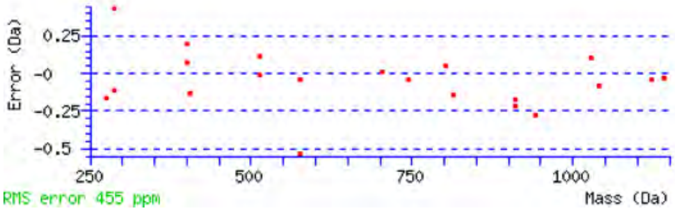

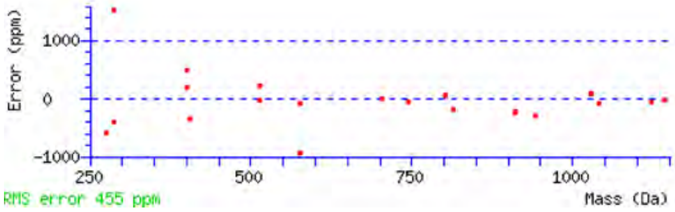

NCBI **BLAST** search of FQKSTELLIR
(Parameters: blastp, nr protein database, expect=20000, no filter, PAM30)
Other BLAST web gateways

All matches to this query

Score	Mr(calc)	Delta	Sequence	Site Analysis
52.9	1313.6744	0.0007	FQKSTELLIR	Phospho S4 94.12%
40.8	1313.6744	0.0007	FQKSTELLIR	Phospho T5 5.88%
20.2	1313.6778	-0.0027	SIMKQQTKTK	
17.7	1313.6744	0.0007	NKGTKYPGATK	
17.4	1313.6744	0.0007	NKGTKYPGATK	
17.4	1313.6745	0.0007	QFKVVDGGSKK	
17.0	1313.6704	0.0047	LSSISQKSRSK	
15.5	1313.6704	0.0047	LSSISQKSRSK	
15.2	1313.6778	-0.0027	SIMKQQTKTK	
15.2	1313.6744	0.0007	SLSPKYLKTR	

Mascot: http://www.matrixscience.com/

MATRIX SCIENCE Mascot Search Results

Peptide View

MS/MS Fragmentation of **EIAQDFKTDLR**
Found in **H3_YEAST** in **S_cerevisiae_D**, sp|P61830|H3_YEAST Histone H3 OS=Saccharomyces cerevisiae (strain ATCC 204508 / S288c) GN=HHT1 PE=1 SV=2

Match to Query 43399: 1348.698328 from(675.356440,2+) intensity(16275.3450) scans(5045) rawscans(sn5045) rtinseconds(1701.9898) index(77201)
Title: 4176: Scan 5045 (rt=28.3665) [D:\MSData\All\VELOS25239.raw]
Data file D:\Data\MGF\533 Final H3 yeast classical PTMs\mascot_daemon_merge.mgf

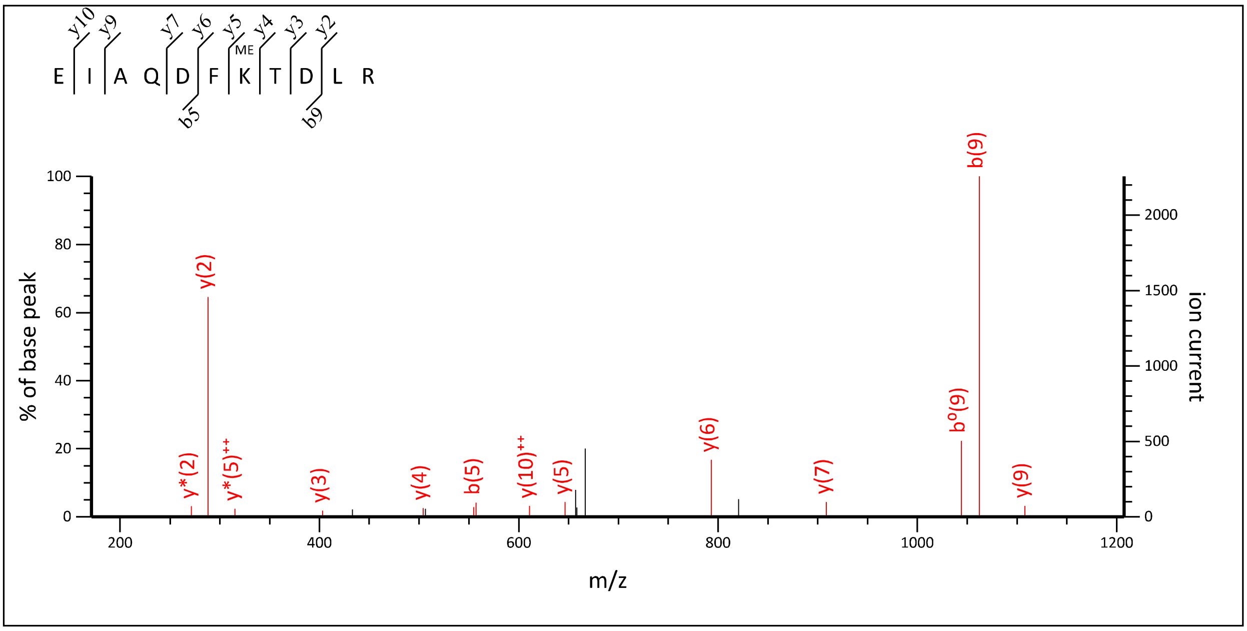

171.28 to 1207.61

Label all possible matches ○ Label matches used for scoring ◉

Monoisotopic mass of neutral peptide Mr(calc): 1348.6987
Fixed modifications: Carbamidomethyl (C) (apply to specified residues or termini only)
Variable modifications:
K7 : Methyl (K)
Ions Score: 62 **Expect:** 5e-05
Matches : 15/110 fragment ions using 20 most intense peaks (help)

#	b	b^{++}	b*	b^{*++}	b^0	b^{0++}	Seq.	y	y^{++}	y*	y^{*++}	y^0	y^{0++}	#
1	130.0499	65.5286			112.0393	56.5233	E							11
2	243.1339	122.0706			225.1234	113.0653	I	1220.6634	**610.8353**	1203.6368	602.3220	1202.6528	601.8300	10
3	314.1710	157.5892			296.1605	148.5839	A	***1107.5793***	**554.2933**	1090.5528	545.7800	1089.5687	545.2880	9
4	442.2296	221.6185	425.2031	213.1052	424.2191	212.6132	Q	1036.5422	518.7747	1019.5156	510.2615	1018.5316	509.7694	8
5	**557.2566**	279.1319	540.2300	270.6186	539.2460	270.1266	D	***908.4836***	454.7454	891.4571	446.2322	890.4730	445.7402	7
6	704.3250	352.6661	687.2984	344.1529	686.3144	343.6608	F	***793.4567***	397.2320	776.4301	388.7187	775.4461	388.2267	6
7	846.4356	423.7214	829.4090	415.2082	828.4250	414.7162	K	***646.3883***	323.6978	629.3617	315.1845	628.3777	314.6925	5
8	947.4833	474.2453	930.4567	465.7320	929.4727	465.2400	T	***504.2776***	252.6425	487.2511	244.1292	486.2671	243.6372	4
9	**1062.5102**	531.7587	1045.4837	523.2455	1044.4997	522.7535	D	***403.2300***	202.1186	386.2034	193.6053	385.2194	193.1133	3
10	1175.5943	588.3008	1158.5677	579.7875	1157.5837	579.2955	L	***288.2030***	144.6051	271.1765	136.0919			2
11							R	175.1190	88.0631	158.0924	79.5498			1

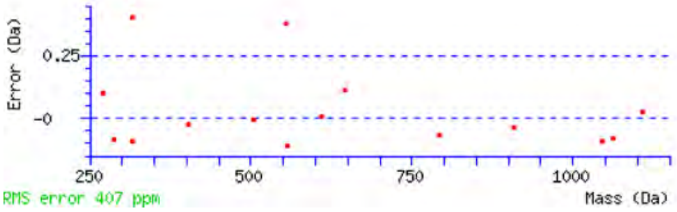

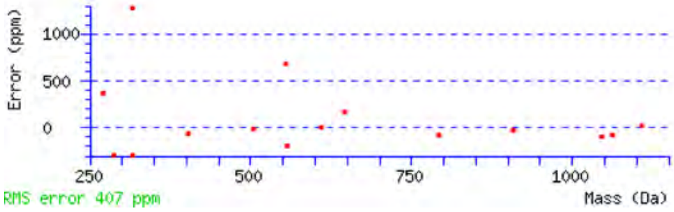

NCBI **BLAST** search of EIAQDFKTDLR
(Parameters: blastp, nr protein database, expect=20000, no filter, PAM30)
Other BLAST web gateways

All matches to this query

Score	Mr(calc)	Delta	Sequence
62.3	1348.6987	-0.0003	EIAQDFKTDLR
15.9	1348.7003	-0.0020	EGASVVKKVVK
13.7	1348.7020	-0.0037	SSLDNCKTAAVK
13.7	1348.7003	-0.0020	SKTPESPKVVK
11.9	1348.7020	-0.0037	SASDKTKLCNK
11.4	1348.6987	-0.0003	EIAQDFKTDLR
11.3	1348.7020	-0.0037	SASDKTKLCNK
10.4	1348.6986	-0.0003	KNIEDEYRVK
10.0	1348.7020	-0.0037	SASDKTKLCNK
10.0	1348.7020	-0.0037	SASDKTKLCNK

Mascot: http://www.matrixscience.com/

MATRIX SCIENCE Mascot Search Results

Peptide View

MS/MS Fragmentation of **EIAQDFKTDLR**
Found in **H3_YEAST** in **S_cerevisiae_D**, sp|P61830|H3_YEAST Histone H3 OS=Saccharomyces cerevisiae (strain ATCC 204508 / S288c) GN=HHT1 PE=1 SV=2

Match to Query 47747: 1376.730492 from(459.917440,3+) intensity(6303.6157) scans(12092) rawscans(sn12092) rtinseconds(3940.7472) index(109444)
Title: 9635: Scan 12092 (rt=65.6791) [D:\MSData\All\VELOS25243.raw]
Data file D:\Data\MGF\533 Final H3 yeast classical PTMs\mascot_daemon_merge.mgf

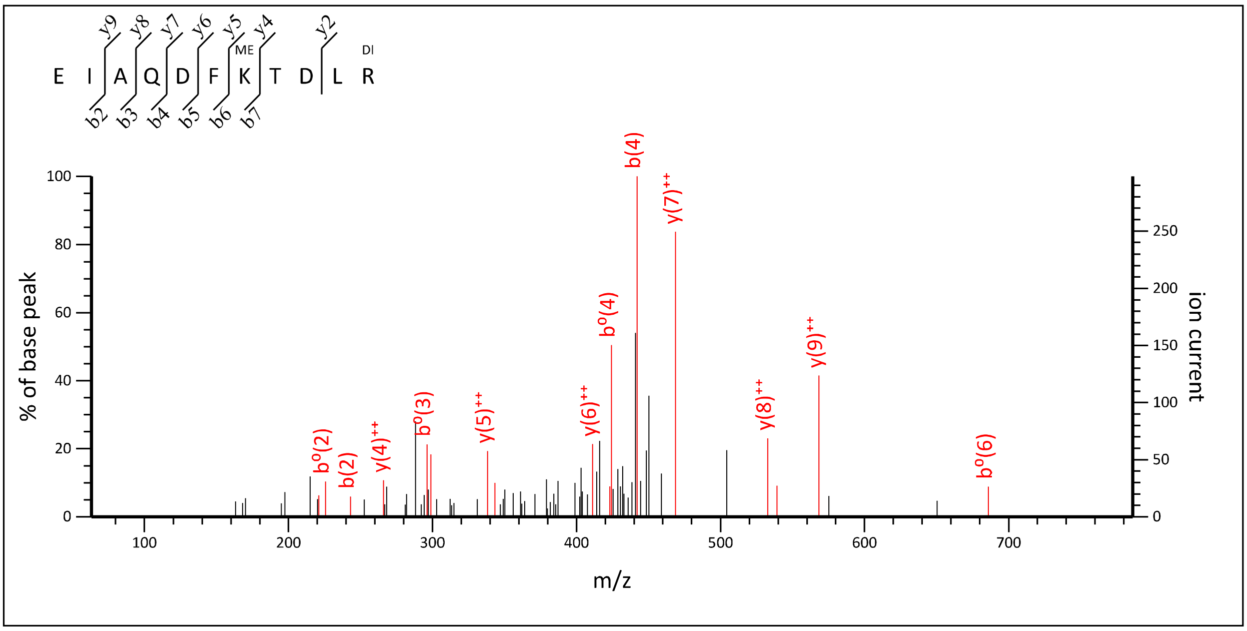

63.21 to 786.15

Label all possible matches ○ Label matches used for scoring ◉

```
Monoisotopic mass of neutral peptide Mr(calc): 1376.7300
Fixed modifications: Carbamidomethyl (C) (apply to specified residues or termini only)
Variable modifications:
K7     : Methyl (K)
R11    : Dimethyl (R)
Ions Score: 34  Expect: 0.026
Matches : 18/110 fragment ions using 26 most intense peaks   (help)
```

#	b	b^{++}	b*	b^{*++}	b^0	b^{0++}	Seq.	y	y^{++}	y*	y^{*++}	y^0	y^{0++}	#
1	130.0499	65.5286			112.0393	56.5233	E							11
2	**243.1339**	122.0706			225.1234	113.0653	I	1248.6947	624.8510	1231.6681	616.3377	1230.6841	615.8457	10
3	314.1710	157.5892			296.1605	148.5839	A	1135.6106	***568.3089***	1118.5841	559.7957	1117.6000	559.3037	9
4	**442.2296**	**221.6185**	425.2031	213.1052	424.2191	212.6132	Q	1064.5735	***532.7904***	1047.5469	524.2771	1046.5629	523.7851	8
5	557.2566	279.1319	540.2300	270.6186	539.2460	270.1266	D	936.5149	***468.7611***	919.4884	460.2478	918.5043	459.7558	7
6	704.3250	352.6661	687.2984	344.1529	686.3144	343.6608	F	821.4880	***411.2476***	804.4614	402.7343	803.4774	402.2423	6
7	846.4356	**423.7214**	829.4090	415.2082	828.4250	414.7162	K	674.4196	***337.7134***	657.3930	329.2001	656.4090	328.7081	5
8	947.4833	474.2453	930.4567	465.7320	929.4727	465.2400	T	532.3089	***266.6581***	515.2824	258.1448	514.2984	257.6528	4
9	1062.5102	531.7587	1045.4837	523.2455	1044.4997	522.7535	D	431.2613	216.1343	414.2347	207.6210	413.2507	207.1290	3
10	1175.5943	588.3008	1158.5677	579.7875	1157.5837	579.2955	L	316.2343	158.6208	299.2078	150.1075			2
11							R	203.1503	102.0788	186.1237	93.5655			1

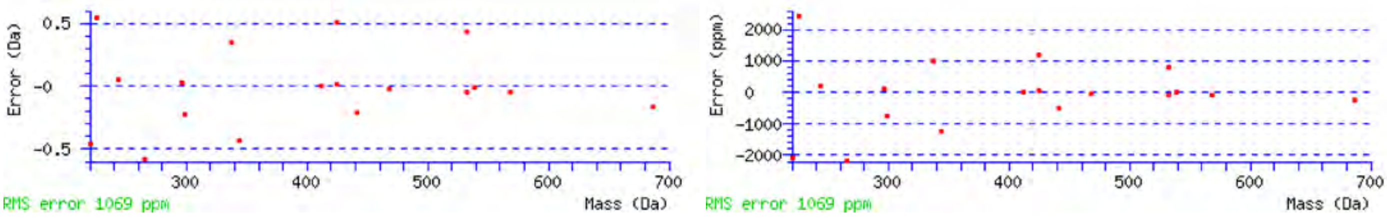

NCBI **BLAST** search of EIAQDFKTDLR
(Parameters: blastp, nr protein database, expect=20000, no filter, PAM30)
Other BLAST web gateways

All matches to this query

Score	Mr(calc)	Delta	Sequence
33.8	1376.7300	0.0005	EIAQDFKTDLR
30.6	1376.7300	0.0005	EIAQDFKTDLR
23.8	1376.7300	0.0005	EIAQDFKTDLR
15.9	1376.7316	-0.0011	KLPSKVASVEK
11.1	1376.7329	-0.0024	SFSGKKFRSR
10.0	1376.7329	-0.0024	SFSGKKFRSR
10.0	1376.7330	-0.0025	SFSGKKFRSR
9.6	1376.7316	-0.0011	KLAETKPELVK
7.2	1376.7316	-0.0011	KYSKKDTTTK
7.2	1376.7316	-0.0011	KYSKKDTTTK

Mascot: http://www.matrixscience.com/

Mascot Search Results

Peptide View

MS/MS Fragmentation of **EIAQDFKTDLR**
Found in **H3_YEAST** in **S_cerevisiae_D**, sp|P61830|H3_YEAST Histone H3 OS=Saccharomyces cerevisiae (strain ATCC 204508 / S288c) GN=HHT1 PE=1 SV=2

Match to Query 45369: 1362.713908 from(682.364230,2+) intensity(10323.3560) scans(5147) rawscans(sn5147) rtinseconds(1732.086) index(77286)
Title: 4261: Scan 5147 (rt=28.8681) [D:\MSData\All\VELOS25239.raw]
Data file D:\Data\MGF\533 Final H3 yeast classical PTMs\mascot_daemon_merge.mgf

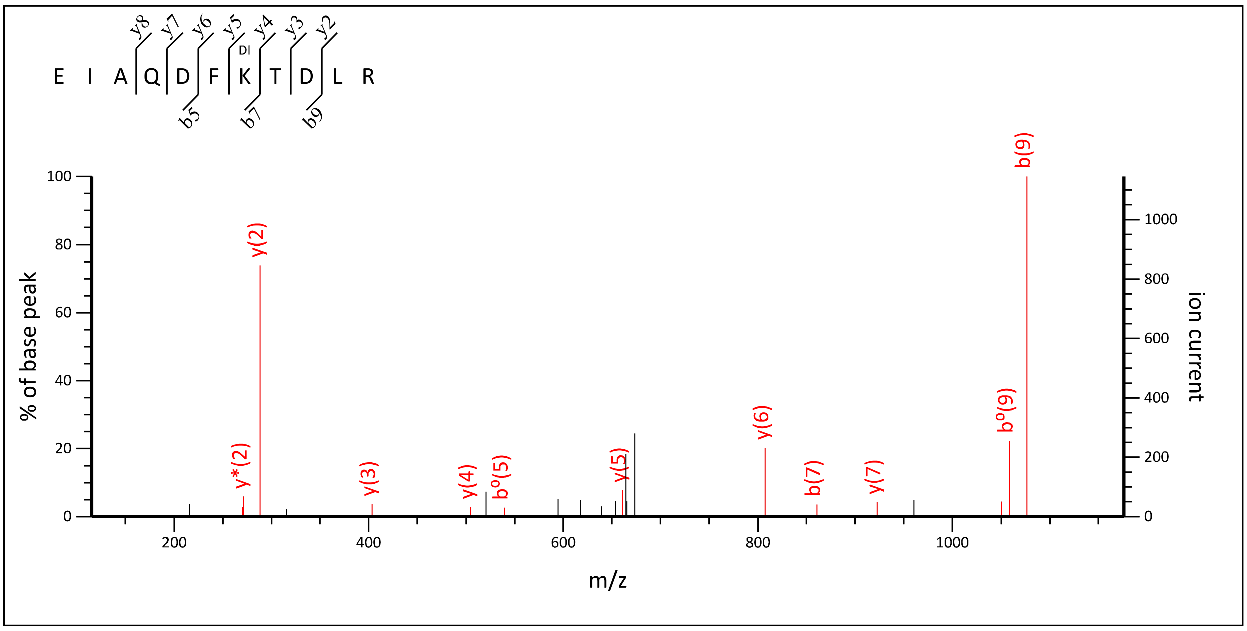

115.31 to 1176.41

Label all possible matches ○ Label matches used for scoring ◉

Monoisotopic mass of neutral peptide Mr(calc): 1362.7143
Fixed modifications: Carbamidomethyl (C) (apply to specified residues or termini only)
Variable modifications:
K7 : Dimethyl (K)
Ions Score: 59 **Expect:** 0.00011
Matches : 13/110 fragment ions using 18 most intense peaks (help)

#	b	b^{++}	b*	b^{*++}	b^{0}	b^{0++}	Seq.	y	y^{++}	y*	y^{*++}	y^{0}	y^{0++}	#
1	130.0499	65.5286			112.0393	56.5233	E							11
2	243.1339	122.0706			225.1234	113.0653	I	1234.6790	617.8431	1217.6525	609.3299	1216.6684	608.8379	10
3	314.1710	157.5892			296.1605	148.5839	A	1121.5949	561.3011	1104.5684	552.7878	1103.5844	552.2958	9
4	442.2296	221.6185	425.2031	213.1052	424.2191	212.6132	Q	**1050.5578**	525.7826	1033.5313	517.2693	1032.5473	516.7773	8
5	557.2566	279.1319	540.2300	270.6186	539.2460	270.1266	D	**922.4993**	461.7533	905.4727	453.2400	904.4887	452.7480	7
6	704.3250	352.6661	687.2984	344.1529	686.3144	343.6608	F	**807.4723**	404.2398	790.4458	395.7265	789.4617	395.2345	6
7	**860.4512**	430.7293	843.4247	422.2160	842.4407	421.7240	K	**660.4039**	330.7056	643.3774	322.1923	642.3933	321.7003	5
8	961.4989	481.2531	944.4724	472.7398	943.4884	472.2478	T	**504.2776**	252.6425	487.2511	244.1292	486.2671	243.6372	4
9	**1076.5259**	538.7666	1059.4993	530.2533	1058.5153	529.7613	D	**403.2300**	202.1186	386.2034	193.6053	385.2194	193.1133	3
10	1189.6099	595.3086	1172.5834	586.7953	1171.5994	586.3033	L	**288.2030**	144.6051	271.1765	136.0919			2
11							R	175.1190	88.0631	158.0924	79.5498			1

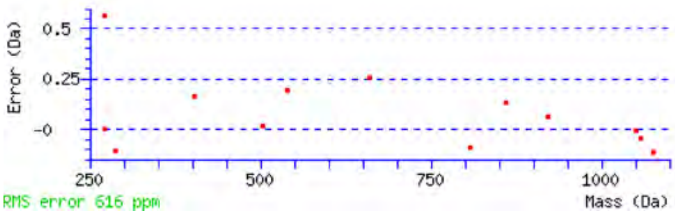

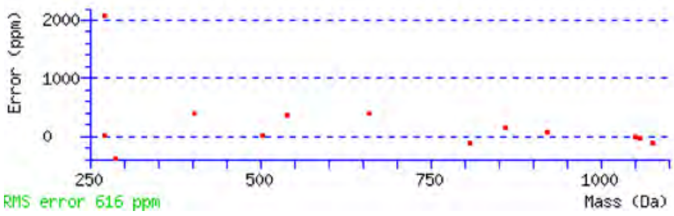

NCBI **BLAST** search of EIAQDFKTDLR
(Parameters: blastp, nr protein database, expect=20000, no filter, PAM30)
Other BLAST web gateways

All matches to this query

Score	Mr(calc)	Delta	Sequence
58.5	1362.7143	-0.0004	EIAQDFKTDLR
15.3	1362.7177	-0.0038	SSLDNCKTAAVK
13.0	1362.7143	-0.0004	LKGDDYNELVR
11.7	1362.7177	-0.0038	SASDKTKLCNK
11.1	1362.7177	-0.0038	SASDKTKLCNK
11.1	1362.7177	-0.0037	NNKTTEAKMSK
11.1	1362.7177	-0.0037	NNKTTEAKMSK
10.5	1362.7160	-0.0021	KYSKKDTTTK
10.4	1362.7159	-0.0020	EKKPSAKEVK
10.1	1362.7159	-0.0020	EKKPSAKEVK

Mascot: http://www.matrixscience.com/

MATRIX SCIENCE Mascot Search Results

Peptide View

MS/MS Fragmentation of **EIAQDFKTDLR**
Found in **H3_YEAST** in **S_cerevisiae_D**, sp|P61830|H3_YEAST Histone H3 OS=Saccharomyces cerevisiae (strain ATCC 204508 / S288c) GN=HHT1 PE=1 SV=2

Match to Query 47779: 1376.730522 from(459.917450,3+) intensity(9033.6123) scans(11810) rawscans(sn11810) rtinseconds(3659.4635) index(119727)
Title: 9515: Scan 11810 (rt=60.9911) [D:\MSData\All\VELOS25245.raw]
Data file D:\Data\MGF\533 Final H3 yeast classical PTMs\mascot_daemon_merge.mgf

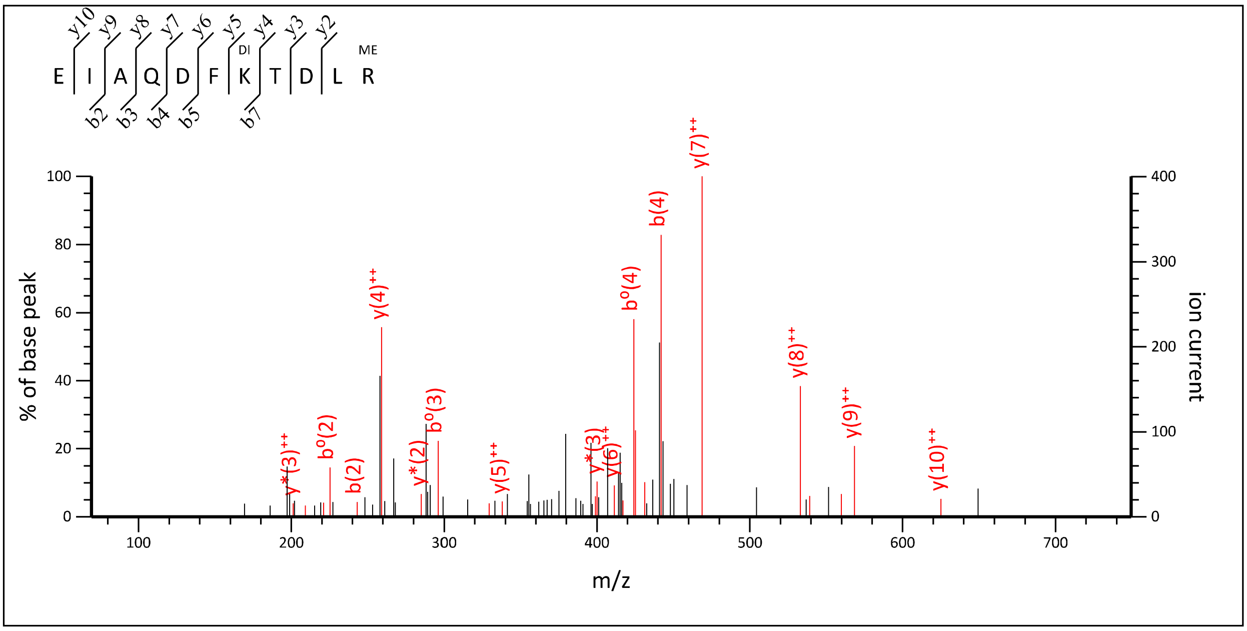

69.24 to 749.46

Label all possible matches ◉ Label matches used for scoring ○

Monoisotopic mass of neutral peptide Mr(calc): 1376.7300
Fixed modifications: Carbamidomethyl (C) (apply to specified residues or termini only)
Variable modifications:
K7 : Dimethyl (K)
R11 : Methyl (R)
Ions Score: 28 **Expect:** 0.16 (help)

#	b	b^{++}	b*	b^{*++}	b^{0}	b^{0++}	Seq.	y	y^{++}	y*	y^{*++}	y^{0}	y^{0++}	#
1	130.0499	65.5286			112.0393	56.5233	E							11
2	**243.1339**	122.0706			**225.1234**	113.0653	I	1248.6947	**624.8510**	1231.6681	616.3377	1230.6841	615.8457	10
3	314.1710	157.5892			**296.1605**	148.5839	A	1135.6106	**568.3089**	1118.5841	**559.7957**	1117.6000	**559.3037**	9
4	**442.2296**	**221.6185**	**425.2031**	213.1052	**424.2191**	212.6132	Q	1064.5735	**532.7904**	1047.5469	524.2771	1046.5629	523.7851	8
5	557.2566	279.1319	540.2300	270.6186	**539.2460**	270.1266	D	936.5149	**468.7611**	919.4884	460.2478	918.5043	459.7558	7
6	704.3250	352.6661	687.2984	344.1529	686.3144	343.6608	F	821.4880	**411.2476**	804.4614	402.7343	803.4774	402.2423	6
7	860.4512	**430.7293**	843.4247	422.2160	842.4407	421.7240	K	674.4196	**337.7134**	657.3930	**329.2001**	656.4090	328.7081	5
8	961.4989	481.2531	944.4724	472.7398	943.4884	472.2478	T	518.2933	**259.6503**	501.2667	251.1370	500.2827	250.6450	4
9	1076.5259	**538.7666**	1059.4993	530.2533	1058.5153	529.7613	D	**417.2456**	**209.1264**	**400.2191**	**200.6132**	**399.2350**	200.1212	3
10	1189.6099	595.3086	1172.5834	586.7953	1171.5994	586.3033	L	302.2187	151.6130	**285.1921**	143.0997			2
11							R	189.1346	95.0709	172.1081	86.5577			1

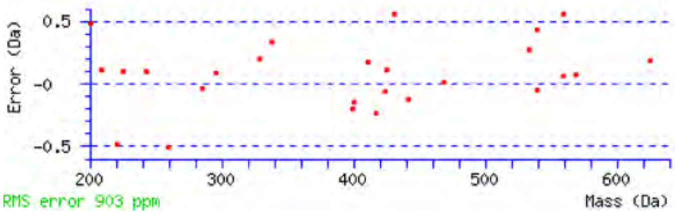

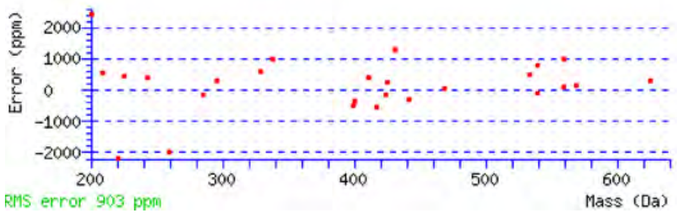

NCBI **BLAST** search of EIAQDFKTDLR
(Parameters: blastp, nr protein database, expect=20000, no filter, PAM30)
Other BLAST web gateways

All matches to this query

Score	Mr(calc)	Delta	Sequence
27.6	1376.7300	0.0006	EIAQDFKTDLR
21.4	1376.7299	0.0006	EQNAKIYNTLK
21.0	1376.7300	0.0006	EIAQDFKTDLR
16.5	1376.7300	0.0006	EIAQDFKTDLR
16.5	1376.7316	-0.0011	KLAETKPELVK
16.0	1376.7316	-0.0011	KLAETKPELVK
14.1	1376.7299	0.0006	AEIQQYKENLK
12.9	1376.7316	-0.0011	EIAKLTPQSLK
9.3	1376.7259	0.0046	RATSTSKGSEQK
9.3	1376.7259	0.0046	RATSTSKGSEQK

Mascot: http://www.matrixscience.com/

Mascot Search Results

Peptide View

MS/MS Fragmentation of **EIAQDFKTDLR**
Found in **H3_YEAST** in **S_cerevisiae_D**, sp|P61830|H3_YEAST Histone H3 OS=Saccharomyces cerevisiae (strain ATCC 204508 / S288c) GN=HHT1 PE=1 SV=2

Match to Query 46862: 1376.729888 from(689.372220,2+) intensity(5558.0596) scans(8130) rawscans(sn8130) rtinseconds(2557.2812) index(116930)
Title: 6718: Scan 8130 (rt=42.6214) [D:\MSData\All\VELOS25245.raw]
Data file D:\Data\MGF\533 Final H3 yeast classical PTMs\mascot_daemon_merge.mgf

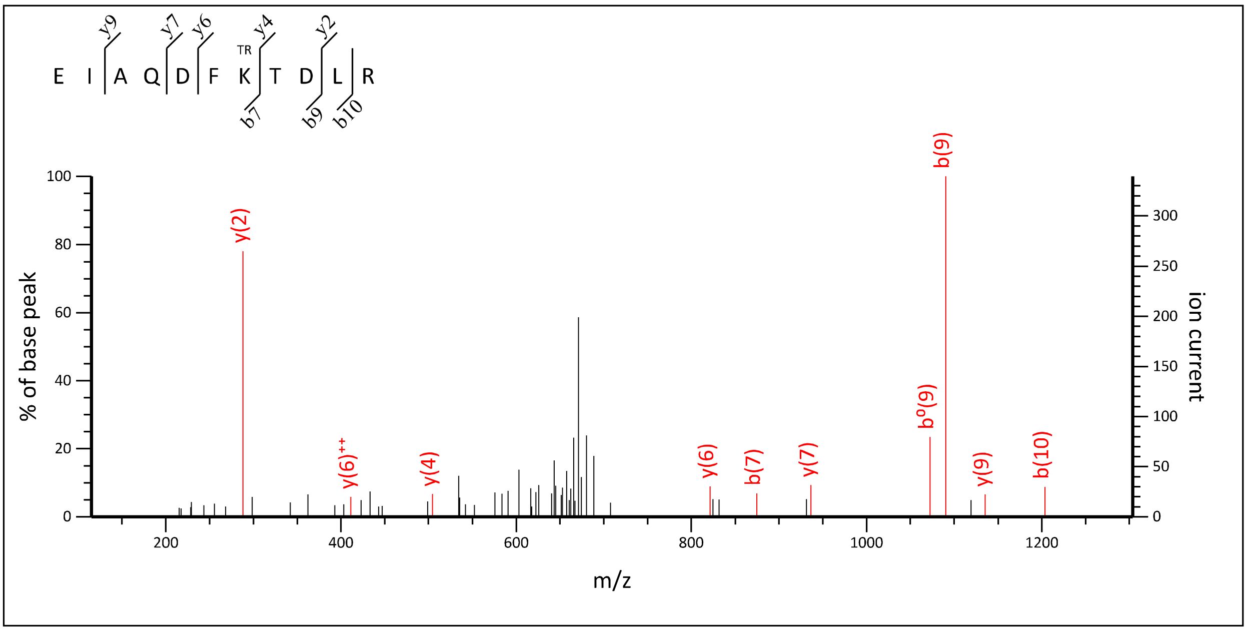

115.14 to 1303.74

Label all possible matches ○ Label matches used for scoring ◉

Monoisotopic mass of neutral peptide Mr(calc): 1376.7300
Fixed modifications: Carbamidomethyl (C) (apply to specified residues or termini only)
Variable modifications:
K7 : Trimethyl (K), with neutral losses 0.0000(shown in table), 59.0735
Ions Score: 29 **Expect:** 0.03
Matches : 10/170 fragment ions using 18 most intense peaks (help)

#	b	b^{++}	b*	b^{*++}	b^{0}	b^{0++}	Seq.	y	y^{++}	y*	y^{*++}	y^{0}	y^{0++}	#
1	130.0499	65.5286			112.0393	56.5233	E							11
2	243.1339	122.0706			225.1234	113.0653	I	1248.6947	624.8510	1231.6681	616.3377	1230.6841	615.8457	10
3	314.1710	157.5892			296.1605	148.5839	A	**1135.6106**	568.3089	1118.5841	559.7957	1117.6000	559.3037	9
4	442.2296	221.6185	425.2031	213.1052	424.2191	212.6132	Q	1064.5735	532.7904	1047.5469	524.2771	1046.5629	523.7851	8
5	557.2566	279.1319	540.2300	270.6186	539.2460	270.1266	D	**936.5149**	468.7611	919.4884	460.2478	918.5043	459.7558	7
6	704.3250	352.6661	687.2984	344.1529	686.3144	343.6608	F	**821.4880**	411.2476	804.4614	402.7343	803.4774	402.2423	6
7	**874.4669**	437.7371	857.4403	429.2238	856.4563	428.7318	K	674.4196	337.7134	657.3930	329.2001	656.4090	328.7081	5
8	975.5146	488.2609	958.4880	479.7477	957.5040	479.2556	T	**504.2776**	252.6425	487.2511	244.1292	486.2671	243.6372	4
9	**1090.5415**	545.7744	1073.5150	537.2611	1072.5310	536.7691	D	403.2300	202.1186	386.2034	193.6053	385.2194	193.1133	3
10	**1203.6256**	602.3164	1186.5990	593.8032	1185.6150	593.3111	L	**288.2030**	144.6051	271.1765	136.0919			2
11							R	175.1190	88.0631	158.0924	79.5498			1

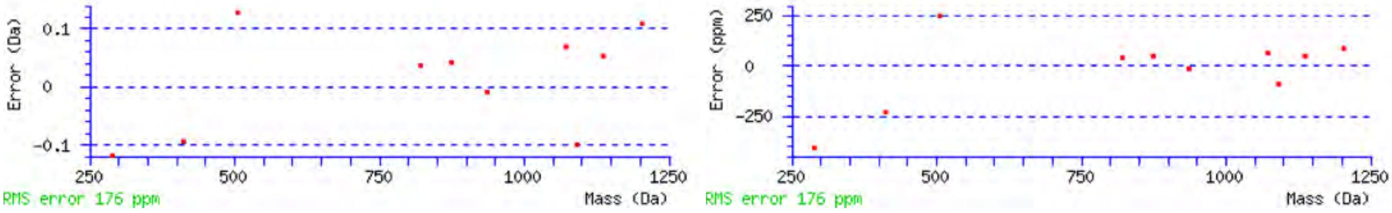

NCBI **BLAST** search of EIAQDFKTDLR
(Parameters: blastp, nr protein database, expect=20000, no filter, PAM30)
Other BLAST web gateways

All matches to this query

Score	Mr(calc)	Delta	Sequence
29.0	1376.7300	-0.0001	EIAQDFKTDLR
11.9	1376.7267	0.0032	MKKQLKTTK
11.9	1376.7347	-0.0048	MRFRSSSHSLK
11.9	1376.7300	-0.0001	PTYINLTDATLR
11.9	1376.7251	0.0048	SGHMAKLKELK
11.9	1376.7251	0.0048	SGHMAKLKELK
11.9	1376.7251	0.0048	SGHMAKLKELK
11.9	1376.7316	-0.0017	LDLKPENASLK
11.8	1376.7333	-0.0034	MKSDDSRDLIK
11.8	1376.7333	-0.0034	SSLDNCKTAAVK

Mascot: http://www.matrixscience.com/

MATRIX SCIENCE Mascot Search Results

Peptide View

MS/MS Fragmentation of **SGRGKGGKGLGKGGAK**
Found in **H4_YEAST** in **S_cerevisiae_D**, sp|P02309|H4_YEAST Histone H4 OS=Saccharomyces cerevisiae (strain ATCC 204508 / S288c) GN=HHF1 PE=1 SV=2

Match to Query 99335: 1595.873028 from(798.943790,2+) intensity(5865.8413) scans(2807) rawscans(sn2807) rtinseconds(1125.3195) index(19387)
Title: 2260: Scan 2807 (rt=18.7553) [D:\MSData\All\VELOS25410.raw]
Data file D:\Data\MGF\531 Final H4 yeast classical PTMs\mascot_daemon_merge.mgf

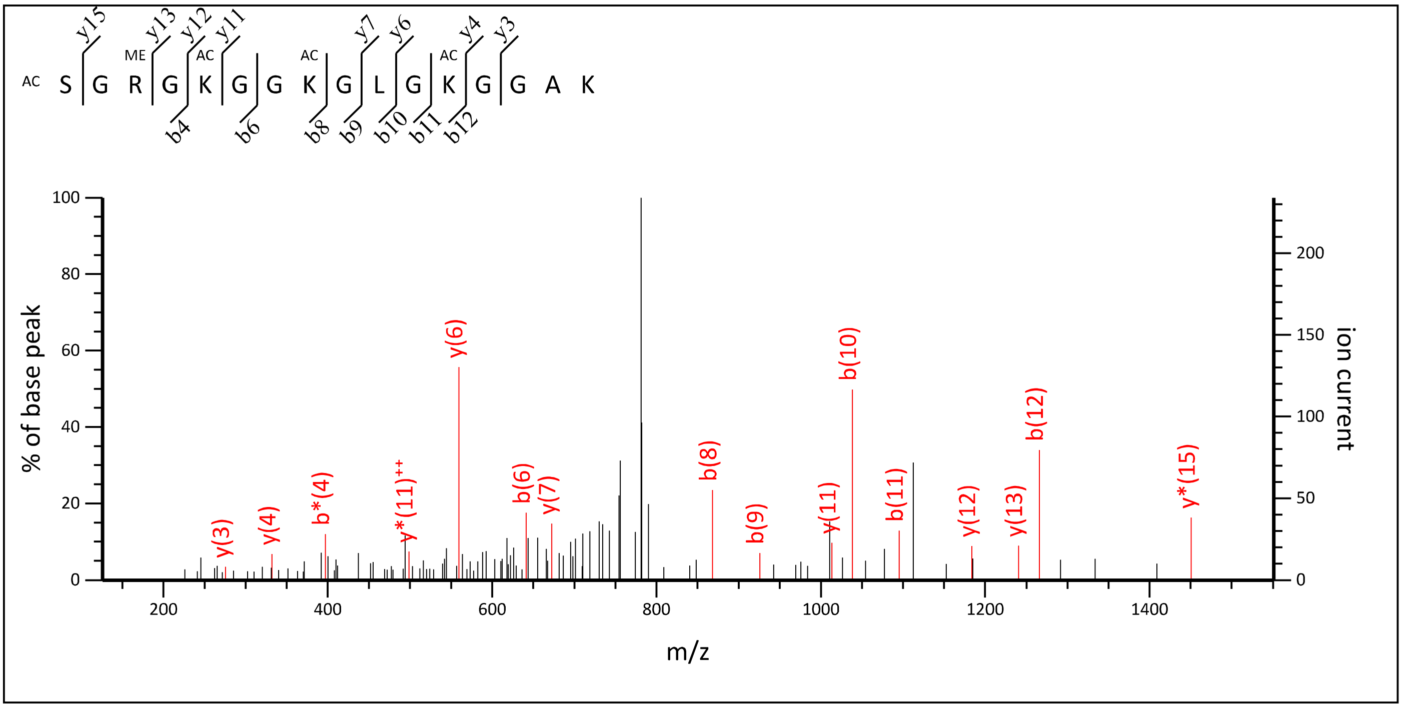

125.99 to 1550.76

Label all possible matches ○ Label matches used for scoring ◉

Monoisotopic mass of neutral peptide Mr(calc): 1595.8743
Fixed modifications: Carbamidomethyl (C) (apply to specified residues or termini only)
Variable modifications:
N-term : Acetyl (Protein N-term)
R3 : Methyl (R)
K5 : Acetyl (K)
K8 : Acetyl (K)
K12 : Acetyl (K)
Ions Score: 45 **Expect:** 0.0013
Matches : 17/146 fragment ions using 36 most intense peaks (help)

#	b	b++	b*	b*++	b0	b0++	Seq.	y	y++	y*	y*++	#
1	130.0499	65.5286			112.0393	56.5233	S					16
2	187.0713	94.0393			169.0608	85.0340	G	1467.8390	734.4232	1450.8125	725.9099	15
3	357.1881	179.0977	340.1615	170.5844	339.1775	170.0924	R	1410.8176	705.9124	1393.7910	697.3992	14
4	414.2096	207.6084	397.1830	199.0951	396.1990	198.6031	G	***1240.7008***	620.8540	1223.6743	612.3408	13
5	584.3151	292.6612	567.2885	284.1479	566.3045	283.6559	K	***1183.6794***	592.3433	1166.6528	583.8300	12
6	***641.3365***	321.1719	624.3100	312.6586	623.3260	312.1666	G	***1013.5738***	507.2905	996.5473	498.7773	11
7	698.3580	349.6826	681.3315	341.1694	680.3474	340.6774	G	956.5524	478.7798	939.5258	470.2665	10
8	***868.4635***	434.7354	851.4370	426.2221	850.4530	425.7301	K	899.5309	450.2691	882.5043	441.7558	9
9	***925.4850***	463.2461	908.4585	454.7329	907.4744	454.2409	G	729.4254	365.2163	712.3988	356.7030	8

**10**	***1038.5691***	519.7882	1021.5425	511.2749	1020.5585	510.7829	**L**	***672.4039***	336.7056	655.3774	328.1923	**7**
**11**	***1095.5905***	548.2989	1078.5640	539.7856	1077.5800	539.2936	**G**	***559.3198***	280.1636	542.2933	271.6503	**6**
**12**	***1265.6961***	633.3517	1248.6695	624.8384	1247.6855	624.3464	**K**	502.2984	251.6528	485.2718	243.1396	**5**
**13**	1322.7175	661.8624	1305.6910	653.3491	1304.7070	652.8571	**G**	***332.1928***	166.6001	315.1663	158.0868	**4**
**14**	1379.7390	690.3731	1362.7124	681.8599	1361.7284	681.3679	**G**	***275.1714***	138.0893	258.1448	129.5761	**3**
**15**	***1450.7761***	725.8917	1433.7496	717.3784	1432.7655	716.8864	**A**	218.1499	109.5786	201.1234	101.0653	**2**
**16**							**K**	147.1128	74.0600	130.0863	65.5468	**1**

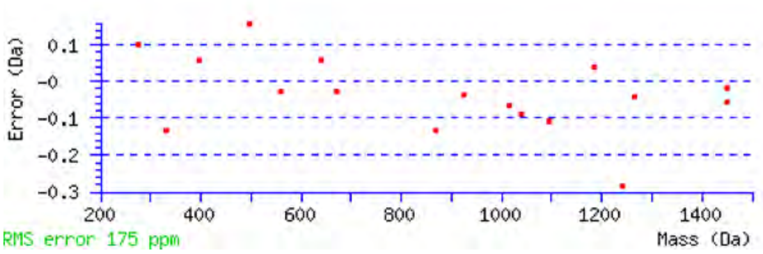

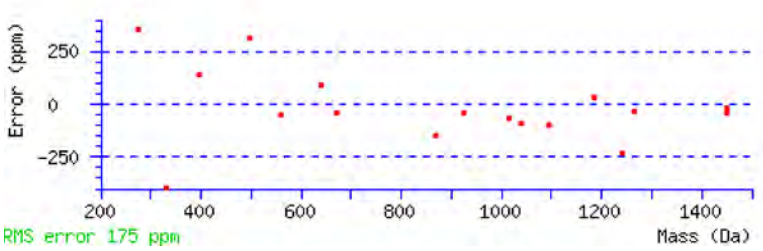

NCBI **BLAST** search of SGRGKGGKGLGKGGAK
(Parameters: blastp, nr protein database, expect=20000, no filter, PAM30)
Other BLAST web gateways

All matches to this query

Score	Mr(calc)	Delta	Sequence	Site Analysis
44.6	1595.8743	-0.0013	SGRGKGGKGLGKGGAK	Methyl R3, Acetyl N-term, K5, K8, K12; 97.44%
28.0	1595.8743	-0.0013	SGRGKGGKGLGKGGAK	Methyl R3, Acetyl N-term, K5, K8, K16; 2.10%
12.9	1595.8783	-0.0053	APDLERWREGIIK	
10.8	1595.8760	-0.0029	KKLSKKNGSGSGNK	
10.6	1595.8760	-0.0029	KKLSKKNGSGSGNK	
10.2	1595.8760	-0.0029	KKLSKKNGSGSGNK	
9.9	1595.8760	-0.0029	KKLSKKNGSGSGNK	
9.9	1595.8760	-0.0029	KKLSKKNGSGSGNK	
9.7	1595.8760	-0.0029	KKLSKKNGSGSGNK	
8.7	1595.8760	-0.0030	SVVKTTDGKTRLR	

Mascot: http://www.matrixscience.com/

Mascot Search Results

Peptide View

MS/MS Fragmentation of **GKGGKGLGKGGAKR**
Found in **H4_YEAST** in **S_cerevisiae_D**, sp|P02309|H4_YEAST Histone H4 OS=Saccharomyces cerevisiae (strain ATCC 204508 / S288c) GN=HHF1 PE=1 SV=2

Match to Query 86140: 1437.804028 from(719.909290,2+) intensity(25900.0740) scans(3111) rawscans(sn3111) rtinseconds(1204.3071) index(2501)
Title: 2502: Scan 3111 (rt=20.0718) [D:\MSData\All\VELOS25408.raw]
Data file D:\Data\MGF\531 Final H4 yeast classical PTMs\mascot_daemon_merge.mgf

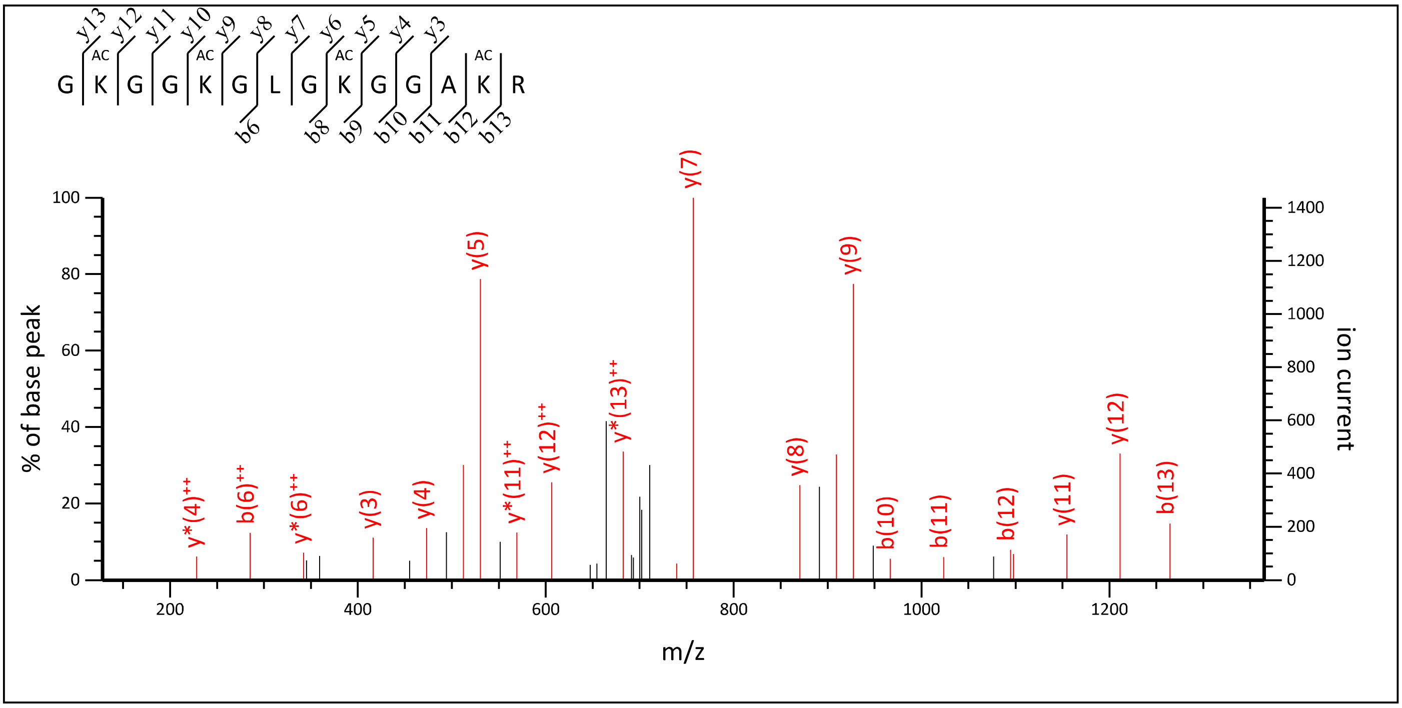

128.29 to 1364.65

Label all possible matches ○ Label matches used for scoring ◉

Monoisotopic mass of neutral peptide Mr(calc): 1437.8052
Fixed modifications: Carbamidomethyl (C) (apply to specified residues or termini only)
Variable modifications:
K2 : Acetyl (K)
K5 : Acetyl (K)
K9 : Acetyl (K)
K13 : Acetyl (K)
Ions Score: 117 **Expect:** 2.7e-10
Matches : 29/102 fragment ions using 28 most intense peaks (help)

#	b	b++	b*	b*++	Seq.	y	y++	y*	y*++	#
1	58.0287	29.5180			G					14
2	228.1343	114.5708	211.1077	106.0575	K	1381.7910	691.3992	1364.7645	682.8859	13
3	285.1557	143.0815	268.1292	134.5682	G	1211.6855	606.3464	1194.6589	597.8331	12
4	342.1772	171.5922	325.1506	163.0790	G	1154.6640	577.8357	1137.6375	569.3224	11
5	512.2827	256.6450	495.2562	248.1317	K	1097.6426	549.3249	1080.6160	540.8116	10
6	569.3042	285.1557	552.2776	276.6425	G	927.5370	464.2722	910.5105	455.7589	9
7	682.3883	341.6978	665.3617	333.1845	L	870.5156	435.7614	853.4890	427.2482	8
8	739.4097	370.2085	722.3832	361.6952	G	757.4315	379.2194	740.4050	370.7061	7
9	909.5152	455.2613	892.4887	446.7480	K	700.4101	350.7087	683.3835	342.1954	6
10	966.5367	483.7720	949.5102	475.2587	G	530.3045	265.6559	513.2780	257.1426	5

11	1023.5582	512.2827	1006.5316	503.7694	G	473.2831	237.1452	456.2565	228.6319	4
12	1094.5953	547.8013	1077.5687	539.2880	A	416.2616	208.6344	399.2350	200.1212	3
13	1264.7008	632.8540	1247.6743	624.3408	K	345.2245	173.1159	328.1979	164.6026	2
14					R	175.1190	88.0631	158.0924	79.5498	1

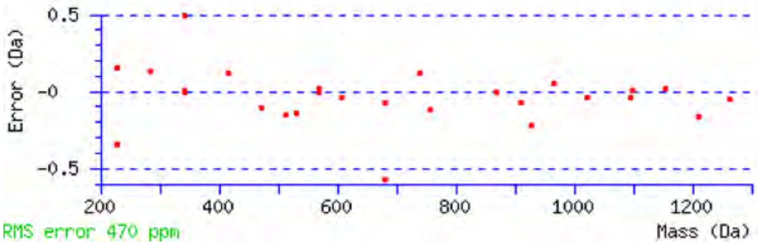

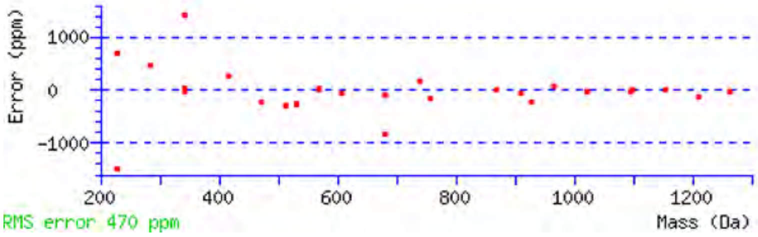

NCBI **BLAST** search of GKGGKGLGKGGAKR
(Parameters: blastp, nr protein database, expect=20000, no filter, PAM30)
Other BLAST web gateways

All matches to this query

Score	Mr(calc)	Delta	Sequence
117.3	1437.8052	-0.0011	GKGGKGLGKGGAKR
32.6	1437.8068	-0.0028	GKSSKKRASKK
24.0	1437.8068	-0.0028	GKSSKKRASKK
23.4	1437.8068	-0.0028	GKSSKKRASKK
21.7	1437.8030	0.0011	ACLKELKKSK
21.7	1437.8030	0.0011	ACLKELKKSK
21.3	1437.8068	-0.0028	GKSSKKRASKK
21.1	1437.8051	-0.0011	NKKGKNRNKK
20.8	1437.8068	-0.0028	GKSSKKRASKK
20.8	1437.8068	-0.0028	GKSSKKRASKK

Mascot: http://www.matrixscience.com/

MATRIX SCIENCE Mascot Search Results

Peptide View

MS/MS Fragmentation of **GKGGKGLGKGGAKR**
Found in **H4_YEAST** in **S_cerevisiae_D**, sp|P02309|H4_YEAST Histone H4 OS=Saccharomyces cerevisiae (strain ATCC 204508 / S288c) GN=HHF1 PE=1 SV=2

Match to Query 83189: 1409.808552 from(470.943460,3+) intensity(31009.3130) scans(1967) rawscans(sn1967) rtinseconds(875.9551) index(141458)
Title: 1585: Scan 1967 (rt=14.5993) [D:\MSData\All\VELOS25426.raw]
Data file D:\Data\MGF\531 Final H4 yeast classical PTMs\mascot_daemon_merge.mgf

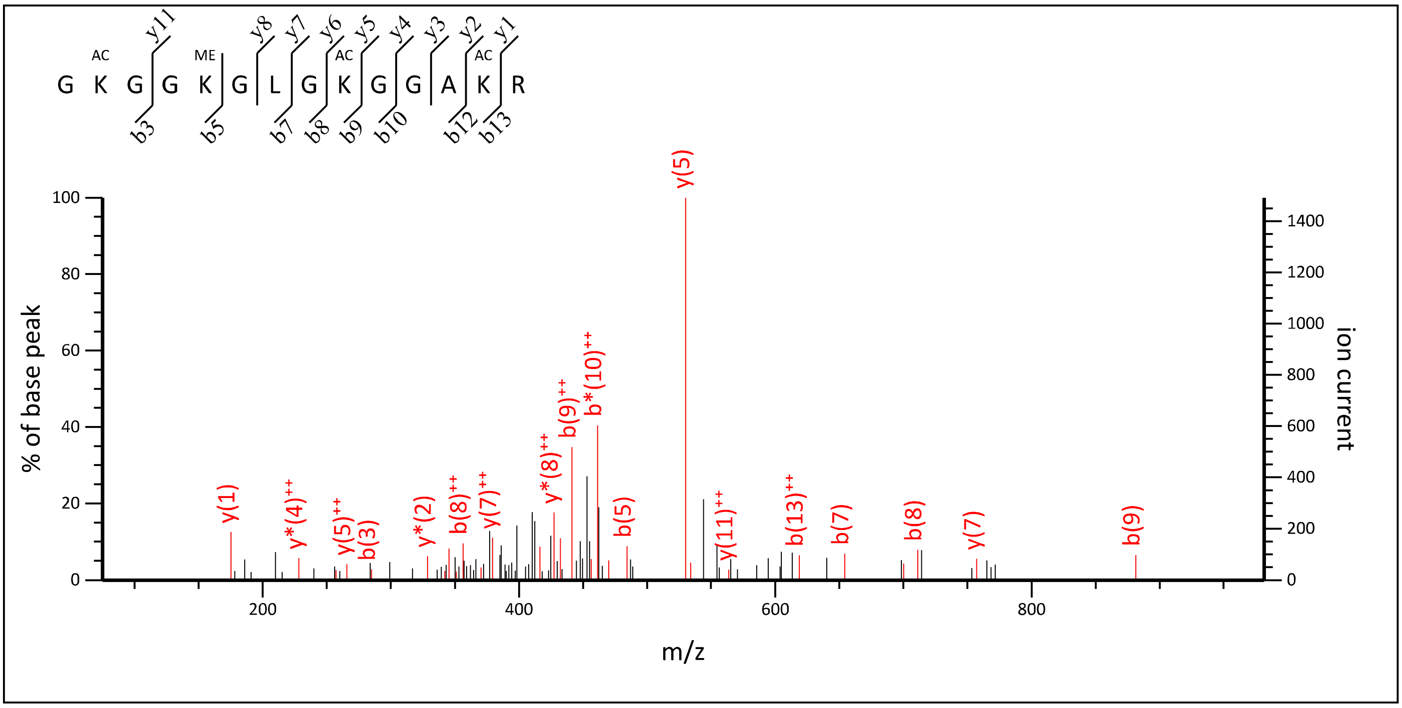

75.1 to 981.48

Label all possible matches ◉ Label matches used for scoring ○

Monoisotopic mass of neutral peptide Mr(calc): 1409.8103
Fixed modifications: Carbamidomethyl (C) (apply to specified residues or termini only)
Variable modifications:
K2 : Acetyl (K)
K5 : Methyl (K)
K9 : Acetyl (K)
K13 : Acetyl (K)
Ions Score: 28 **Expect:** 0.14 (help)

#	b	b++	b*	b*++	Seq.	y	y++	y*	y*++	#
1	58.0287	29.5180			G					14
2	**228.1343**	114.5708	211.1077	106.0575	K	1353.7961	677.4017	1336.7696	668.8884	13
3	**285.1557**	143.0815	268.1292	134.5682	G	1183.6906	592.3489	1166.6640	583.8357	12
4	**342.1772**	171.5922	325.1506	163.0790	G	1126.6691	**563.8382**	1109.6426	555.3249	11
5	**484.2878**	242.6475	467.2613	234.1343	K	1069.6477	535.3275	1052.6211	526.8142	10
6	541.3093	271.1583	524.2827	262.6450	G	927.5370	464.2722	910.5105	**455.7589**	9
7	**654.3933**	327.7003	637.3668	319.1870	L	870.5156	435.7614	853.4890	**427.2482**	8
8	**711.4148**	**356.2110**	694.3883	347.6978	G	**757.4315**	**379.2194**	740.4050	**370.7061**	7
9	**881.5203**	**441.2638**	864.4938	**432.7505**	K	**700.4101**	**350.7087**	683.3835	**342.1954**	6
10	938.5418	**469.7745**	921.5152	**461.2613**	G	**530.3045**	**265.6559**	513.2780	**257.1426**	5

11	995.5633	498.2853	978.5367	489.7720	G	473.2831	237.1452	456.2565	228.6319	4
12	1066.6004	533.8038	1049.5738	525.2905	A	416.2616	208.6344	399.2350	200.1212	3
13	1236.7059	618.8566	1219.6794	610.3433	K	345.2245	173.1159	328.1979	164.6026	2
14					R	175.1190	88.0631	158.0924	79.5498	1

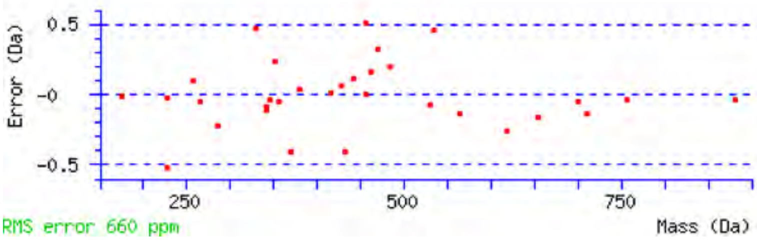

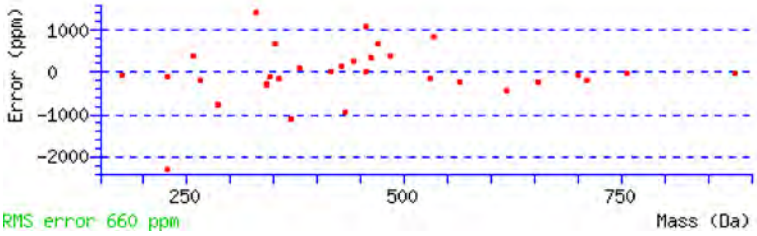

NCBI **BLAST** search of GKGGKGLGKGGAKR
(Parameters: blastp, nr protein database, expect=20000, no filter, PAM30)
Other BLAST web gateways

All matches to this query

Score	Mr(calc)	Delta	Sequence
27.8	1409.8103	-0.0017	GKGGKGLGKGGAKR
22.0	1409.8103	-0.0017	GKGGKGLGKGGAKR
17.4	1409.8103	-0.0017	KAGGKGLGKGGKGR
12.5	1409.8132	-0.0047	RFKKIRSRR
11.3	1409.8103	-0.0017	KAGGKGLGKGGKGR
11.3	1409.8119	-0.0033	GKSSKKRASKK
11.3	1409.8119	-0.0033	GKSSKKRASKK
11.3	1409.8119	-0.0033	GKSSKKRASKK
11.3	1409.8119	-0.0033	GKSSKKRASKK
10.7	1409.8119	-0.0033	KSARKKSSKGK

Mascot: http://www.matrixscience.com/

Mascot Search Results

Peptide View

MS/MS Fragmentation of **GKGGKGLGKGGAKR**
Found in **H4_YEAST** in **S_cerevisiae_D**, sp|P02309|H4_YEAST Histone H4 OS=Saccharomyces cerevisiae (strain ATCC 204508 / S288c) GN=HHF1 PE=1 SV=2

Match to Query 87687: 1451.820588 from(726.917570,2+) intensity(22661.0760) scans(3492) rawscans(sn3492) rtinseconds(1239.0236) index(82259)
Title: 2908: Scan 3492 (rt=20.6504) [D:\MSData\All\VELOS25418.raw]
Data file D:\Data\MGF\531 Final H4 yeast classical PTMs\mascot_daemon_merge.mgf

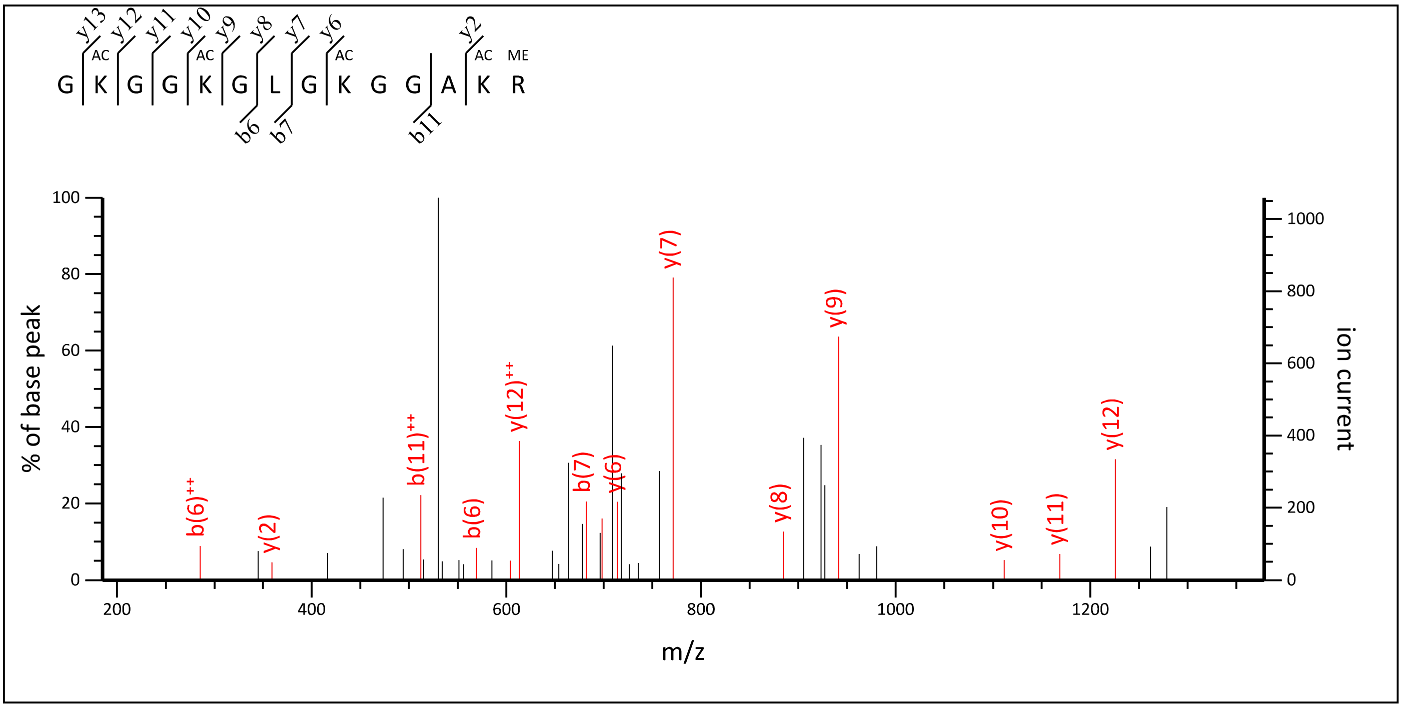

185.21 to 1378.54

Label all possible matches ○ Label matches used for scoring ●

Monoisotopic mass of neutral peptide Mr(calc): 1451.8208
Fixed modifications: Carbamidomethyl (C) (apply to specified residues or termini only)
Variable modifications:
K2 : Acetyl (K)
K5 : Acetyl (K)
K9 : Acetyl (K)
K13 : Acetyl (K)
R14 : Methyl (R)
Ions Score: 43 **Expect:** 0.0074
Matches : 17/102 fragment ions using 35 most intense peaks (help)

#	b	b++	b*	b*++	Seq.	y	y++	y*	y*++	#
1	58.0287	29.5180			G					14
2	228.1343	114.5708	211.1077	106.0575	K	1395.8067	**698.4070**	1378.7801	689.8937	13
3	**285.1557**	143.0815	268.1292	134.5682	G	***1225.7011***	**613.3542**	1208.6746	604.8409	12
4	342.1772	171.5922	325.1506	163.0790	G	***1168.6797***	584.8435	1151.6531	576.3302	11
5	**512.2827**	256.6450	495.2562	248.1317	K	***1111.6582***	556.3327	1094.6317	547.8195	10
6	**569.3042**	**285.1557**	552.2776	276.6425	G	***941.5527***	471.2800	924.5261	462.7667	9
7	**682.3883**	341.6978	665.3617	333.1845	L	***884.5312***	442.7693	867.5047	434.2560	8
8	739.4097	370.2085	722.3832	361.6952	G	***771.4472***	386.2272	754.4206	377.7139	7
9	909.5152	455.2613	892.4887	446.7480	K	***714.4257***	357.7165	697.3992	349.2032	6

**10**	966.5367	483.7720	949.5102	475.2587	**G**	544.3202	272.6637	527.2936	264.1504	**5**
**11**	1023.5582	**512.2827**	1006.5316	503.7694	**G**	487.2987	244.1530	470.2722	235.6397	**4**
**12**	1094.5953	547.8013	1077.5687	539.2880	**A**	430.2772	215.6423	413.2507	207.1290	**3**
**13**	1264.7008	632.8540	1247.6743	624.3408	**K**	***359.2401***	180.1237	342.2136	171.6104	**2**
**14**					**R**	189.1346	95.0709	172.1081	86.5577	**1**

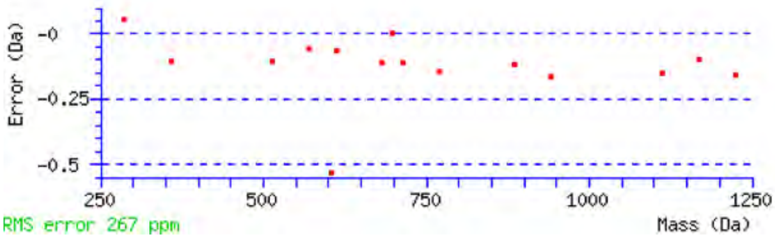

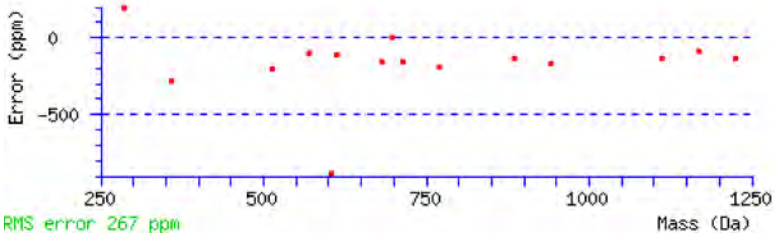

NCBI **BLAST** search of GKGGKGLGKGGAKR
(Parameters: blastp, nr protein database, expect=20000, no filter, PAM30)
Other BLAST web gateways

All matches to this query

Score	Mr(calc)	Delta	Sequence
43.0	1451.8208	-0.0002	GKGGKGLGKGGAKR
22.9	1451.8208	-0.0002	NGKKGKAKQKR
18.4	1451.8225	-0.0019	KKGKKSSNGGNK
18.4	1451.8225	-0.0019	KKGKKSSNGGNK
17.2	1451.8208	-0.0002	NKKGKNRNKK
17.1	1451.8225	-0.0019	KKGKKSSNGGNK
17.1	1451.8225	-0.0019	KKGKKSSNGGNK
16.0	1451.8265	-0.0059	ERIKFKPKSK
15.9	1451.8160	0.0046	CKKVIKRTVGR
15.9	1451.8160	0.0046	CKKVIKRTVGR

Mascot: http://www.matrixscience.com/

MATRIX SCIENCE Mascot Search Results

Peptide View

MS/MS Fragmentation of **DNIQGITKPAIR**
Found in **H4_YEAST** in **S_cerevisiae_D**, sp|P02309|H4_YEAST Histone H4 OS=Saccharomyces cerevisiae (strain ATCC 204508 / S288c) GN=HHF1 PE=1 SV=2

Match to Query 77200: 1366.756668 from(684.385610,2+) intensity(40341.5230) scans(6268) rawscans(sn6268) rtinseconds(2039.3151) index(84583)
Title: 5232: Scan 6268 (rt=33.9886) [D:\MSData\All\VELOS25418.raw]
Data file D:\Data\MGF\531 Final H4 yeast classical PTMs\mascot_daemon_merge.mgf

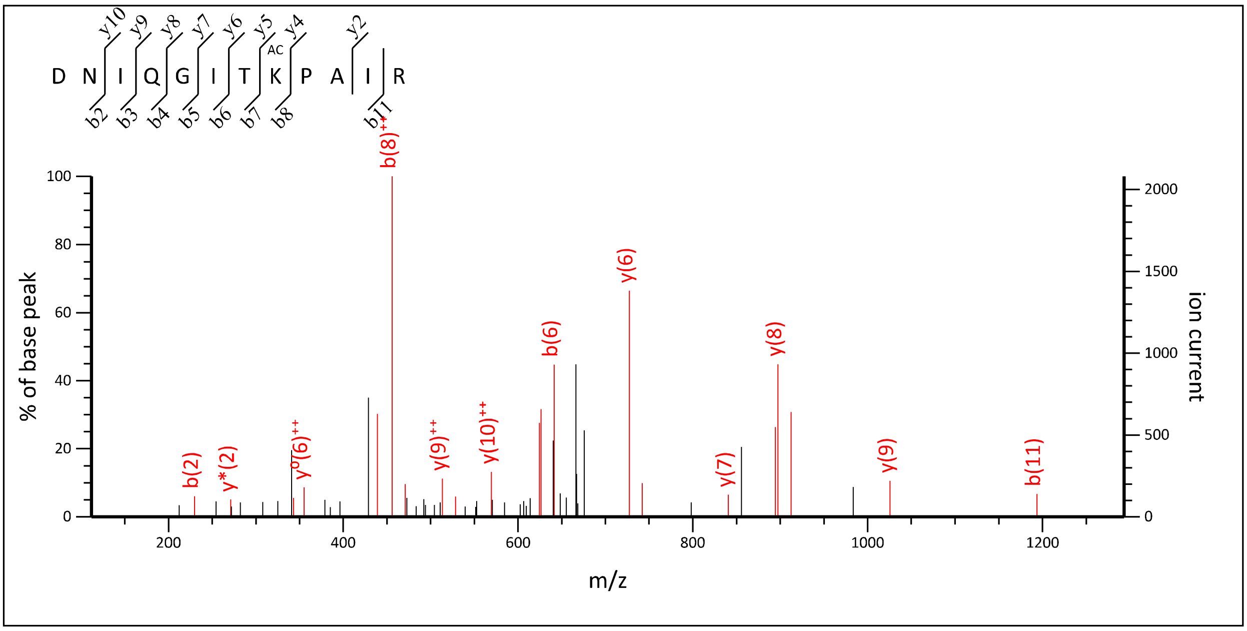

111.98 to 1293.59

Label all possible matches ○ Label matches used for scoring ◉

Monoisotopic mass of neutral peptide Mr(calc): 1366.7568
Fixed modifications: Carbamidomethyl (C) (apply to specified residues or termini only)
Variable modifications:
K8 : Acetyl (K)
Ions Score: 62 **Expect:** 8.2e-05
Matches : 22/120 fragment ions using 31 most intense peaks (help)

#	b	b^{++}	b*	b^{*++}	b^0	b^{0++}	Seq.	y	y^{++}	y*	y^{*++}	y^0	y^{0++}	#
1	116.0342	58.5207			98.0237	49.5155	D							12
2	***230.0771***	115.5422	213.0506	107.0289	212.0666	106.5369	N	1252.7372	626.8722	1235.7106	618.3590	1234.7266	617.8670	11
3	***343.1612***	172.0842	326.1347	163.5710	325.1506	163.0790	I	1138.6943	**569.8508**	1121.6677	561.3375	1120.6837	560.8455	10
4	***471.2198***	236.1135	454.1932	227.6003	453.2092	227.1082	Q	**1025.6102**	**513.3087**	1008.5837	504.7955	1007.5996	504.3035	9
5	***528.2413***	264.6243	511.2147	256.1110	510.2307	255.6190	G	**897.5516**	449.2795	880.5251	440.7662	879.5411	440.2742	8
6	***641.3253***	321.1663	624.2988	312.6530	623.3148	312.1610	I	**840.5302**	420.7687	823.5036	412.2554	822.5196	411.7634	7
7	***742.3730***	371.6901	725.3464	363.1769	724.3624	362.6849	T	**727.4461**	364.2267	710.4196	355.7134	709.4355	355.2214	6
8	***912.4785***	456.7429	895.4520	448.2296	894.4680	447.7376	K	**626.3984**	313.7028	609.3719	305.1896			5
9	1009.5313	505.2693	992.5047	496.7560	991.5207	496.2640	P	**456.2929**	228.6501	439.2663	220.1368			4
10	1080.5684	540.7878	1063.5419	532.2746	1062.5578	531.7826	A	359.2401	180.1237	342.2136	171.6104			3
11	***1193.6525***	597.3299	1176.6259	588.8166	1175.6419	588.3246	I	288.2030	144.6051	271.1765	136.0919			2
12							R	175.1190	88.0631	158.0924	79.5498			1

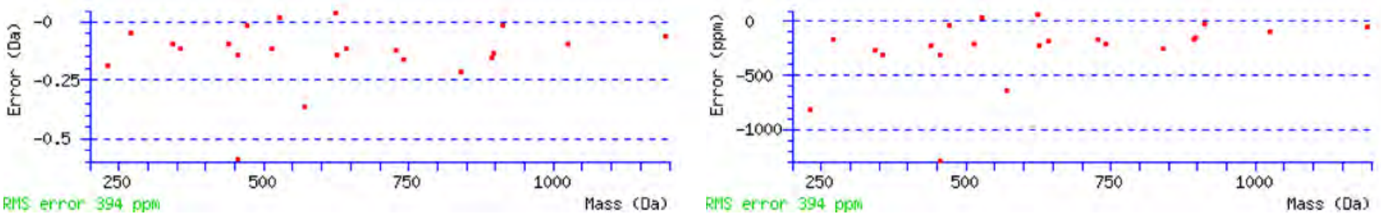

NCBI **BLAST** search of DNIQGITKPAIR
(Parameters: blastp, nr protein database, expect=20000, no filter, PAM30)
Other BLAST web gateways

All matches to this query

Score	Mr(calc)	Delta	Sequence
62.1	1366.7568	-0.0002	DNIQGITKPAIR
27.8	1366.7585	-0.0018	KGKKSEKKSK
24.7	1366.7585	-0.0018	SKKGKKSEKK
18.8	1366.7568	-0.0002	NDIAQVPKNANK
18.7	1366.7585	-0.0018	KTATSKPGGSKK
18.7	1366.7585	-0.0018	KTATSKPGGSKK
18.2	1366.7520	0.0047	MIKRSLASLVR
16.4	1366.7585	-0.0018	KKKTVKESNK
16.4	1366.7585	-0.0018	KKKTVKESNK
16.4	1366.7585	-0.0018	SKKGKKSEKK

Mascot: http://www.matrixscience.com/

MATRIX SCIENCE Mascot Search Results

Peptide View

MS/MS Fragmentation of **DNIQGITKPAIR**
Found in **H4_YEAST** in **S_cerevisiae_D**, sp|P02309|H4_YEAST Histone H4 OS=Saccharomyces cerevisiae (strain ATCC 204508 / S288c) GN=HHF1 PE=1 SV=2

Match to Query 74987: 1352.776708 from(677.395630,2+) intensity(14573.0800) scans(5257) rawscans(sn5257) rtinseconds(1684.8249) index(127975)
Title: 4494: Scan 5257 (rt=28.0804) [D:\MSData\All\VELOS25424.raw]
Data file D:\Data\MGF\531 Final H4 yeast classical PTMs\mascot_daemon_merge.mgf

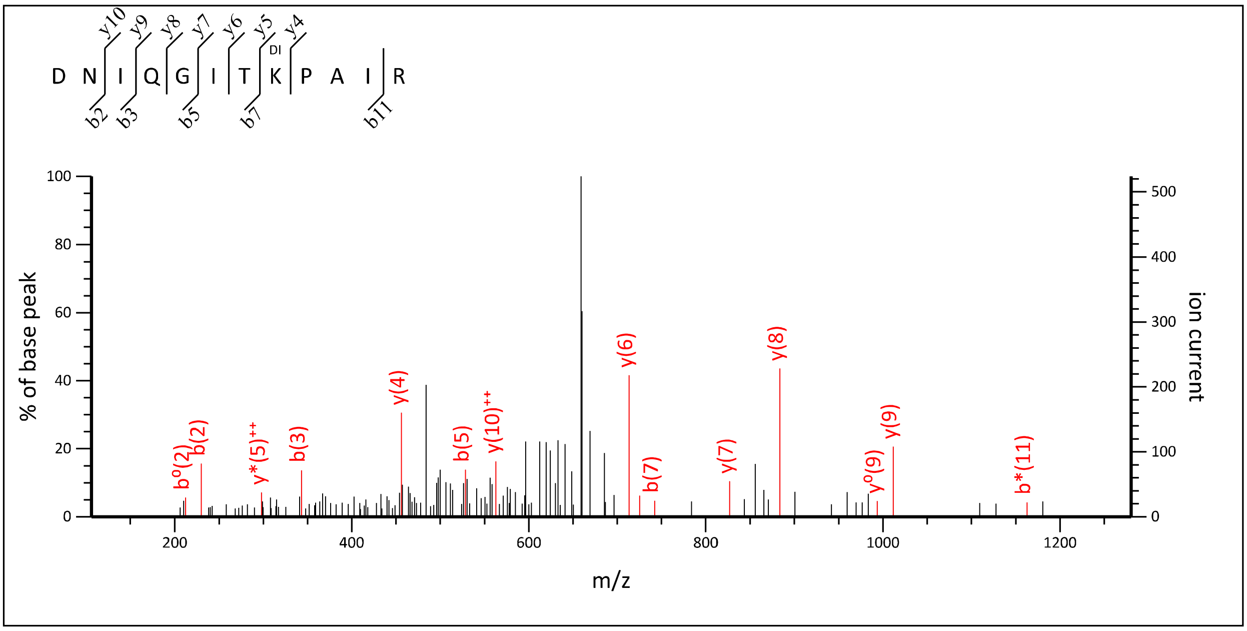

106.02 to 1280.32

Label all possible matches ○ Label matches used for scoring ◉

Monoisotopic mass of neutral peptide Mr(calc): 1352.7776
Fixed modifications: Carbamidomethyl (C) (apply to specified residues or termini only)
Variable modifications:
K8 : Dimethyl (K)
Ions Score: 29 **Expect:** 0.13
Matches : 15/120 fragment ions using 28 most intense peaks (help)

#	b	b^{++}	b*	b^{*++}	b^0	b^{0++}	Seq.	y	y^{++}	y*	y^{*++}	y^0	y^{0++}	#
1	116.0342	58.5207			98.0237	49.5155	D							12
2	***230.0771***	115.5422	213.0506	107.0289	212.0666	106.5369	N	1238.7579	619.8826	1221.7314	611.3693	1220.7474	610.8773	11
3	***343.1612***	172.0842	326.1347	163.5710	325.1506	163.0790	I	1124.7150	562.8611	1107.6885	554.3479	1106.7044	553.8559	10
4	471.2198	236.1135	454.1932	227.6003	453.2092	227.1082	Q	***1011.6309***	506.3191	994.6044	497.8058	993.6204	497.3138	9
5	***528.2413***	264.6243	511.2147	256.1110	510.2307	255.6190	G	***883.5724***	442.2898	866.5458	433.7765	865.5618	433.2845	8
6	641.3253	321.1663	624.2988	312.6530	623.3148	312.1610	I	***826.5509***	413.7791	809.5244	405.2658	808.5403	404.7738	7
7	***742.3730***	371.6901	725.3464	363.1769	724.3624	362.6849	T	***713.4668***	357.2371	696.4403	348.7238	695.4563	348.2318	6
8	898.4993	449.7533	881.4727	441.2400	880.4887	440.7480	K	612.4192	306.7132	595.3926	298.1999			5
9	995.5520	498.2796	978.5255	489.7664	977.5415	489.2744	P	***456.2929***	228.6501	439.2663	220.1368			4
10	1066.5891	533.7982	1049.5626	525.2849	1048.5786	524.7929	A	359.2401	180.1237	342.2136	171.6104			3
11	1179.6732	590.3402	1162.6467	581.8270	1161.6626	581.3350	I	288.2030	144.6051	271.1765	136.0919			2
12							R	175.1190	88.0631	158.0924	79.5498			1

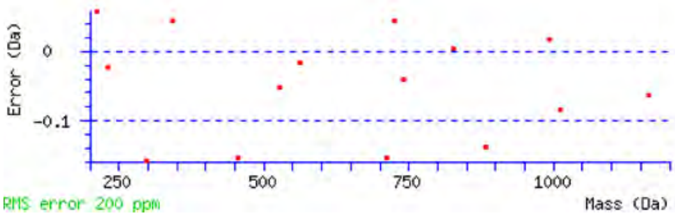

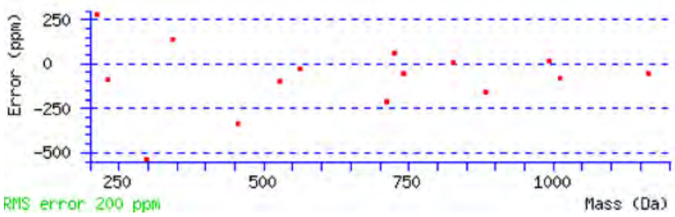

NCBI **BLAST** search of DNIQGITKPAIR
(Parameters: blastp, nr protein database, expect=20000, no filter, PAM30)
Other BLAST web gateways

All matches to this query

Score	Mr(calc)	Delta	Sequence
29.5	1352.7776	-0.0009	DNIQGITKPAIR
26.1	1352.7776	-0.0009	DNIQGITKPAIR
20.8	1352.7776	-0.0009	DNIQGITKPAIR
18.2	1352.7792	-0.0025	TKLKKDTKKK
15.6	1352.7792	-0.0025	TKLKKDTKKK
15.3	1352.7792	-0.0025	TKLKKDTKKK
14.6	1352.7776	-0.0009	GISVPGTKDPGKR
11.4	1352.7776	-0.0009	GISVPGTKDPGKR
11.4	1352.7776	-0.0009	GISVPGTKDPGKR
11.0	1352.7792	-0.0025	TKLKNKSKSK

Mascot: http://www.matrixscience.com/

Mascot Search Results

Peptide View

MS/MS Fragmentation of **ISGLIYEEVR**
Found in **H4_YEAST** in **S_cerevisiae_D**, sp|P02309|H4_YEAST Histone H4 OS=Saccharomyces cerevisiae (strain ATCC 204508 / S288c) GN=HHF1 PE=1 SV=2

Match to Query 53733: 1205.665688 from(603.840120,2+) intensity(10438.7610) scans(10087) rawscans(sn10087) rtinseconds(3037.4058) index(8447)
Title: 8448: Scan 10087 (rt=50.6234) [D:\MSData\All\VELOS25408.raw]
Data file D:\Data\MGF\531 Final H4 yeast classical PTMs\mascot_daemon_merge.mgf

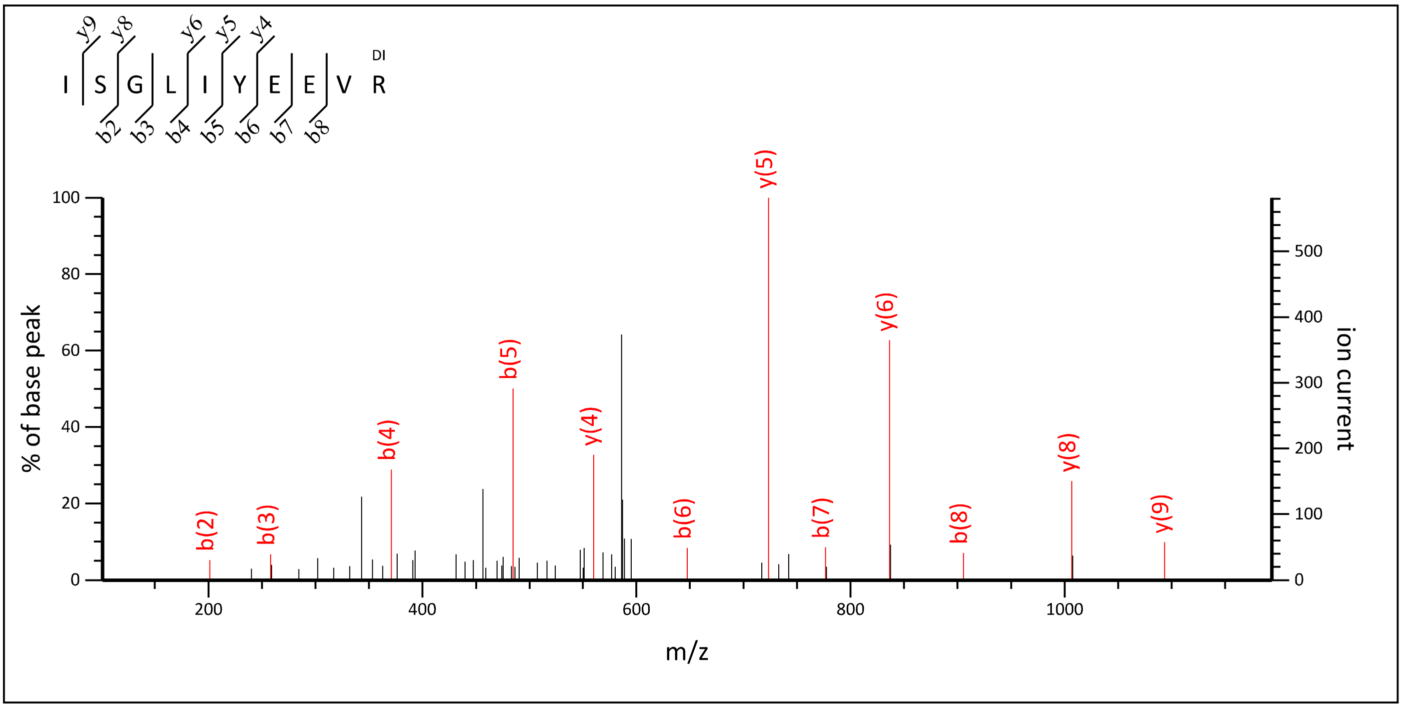

101.18 to 1193.56

Label all possible matches ○ Label matches used for scoring ◉

Monoisotopic mass of neutral peptide Mr(calc): 1205.6656
Fixed modifications: Carbamidomethyl (C) (apply to specified residues or termini only)
Variable modifications:
R10 : Dimethyl (R)
Ions Score: 63 **Expect:** 4.5e-05
Matches : 12/84 fragment ions using 16 most intense peaks (help)

#	b	b^{++}	b^0	b^{0++}	Seq.	y	y^{++}	y^*	y^{*++}	y^0	y^{0++}	#
1	114.0913	57.5493			I							10
2	*201.1234*	101.0653	183.1128	92.0600	S	**1093.5888**	547.2980	1076.5623	538.7848	1075.5782	538.2928	9
3	*258.1448*	129.5761	240.1343	120.5708	G	**1006.5568**	503.7820	989.5302	495.2688	988.5462	494.7767	8
4	*371.2289*	186.1181	353.2183	177.1128	L	949.5353	475.2713	932.5088	466.7580	931.5247	466.2660	7
5	*484.3130*	242.6601	466.3024	233.6548	I	**836.4512**	418.7293	819.4247	410.2160	818.4407	409.7240	6
6	*647.3763*	324.1918	629.3657	315.1865	Y	**723.3672**	362.1872	706.3406	353.6740	705.3566	353.1819	5
7	*776.4189*	388.7131	758.4083	379.7078	E	**560.3039**	280.6556	543.2773	272.1423	542.2933	271.6503	4
8	*905.4615*	453.2344	887.4509	444.2291	E	431.2613	216.1343	414.2347	207.6210	413.2507	207.1290	3
9	1004.5299	502.7686	986.5193	493.7633	V	302.2187	151.6130	285.1921	143.0997			2
10					R	203.1503	102.0788	186.1237	93.5655			1

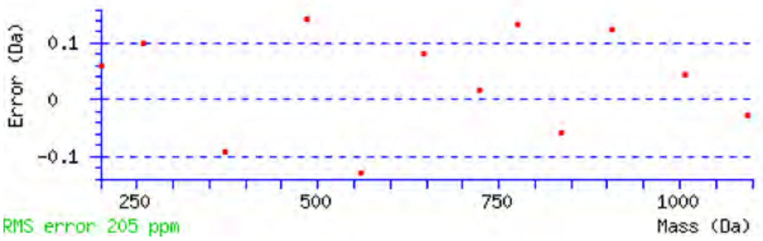

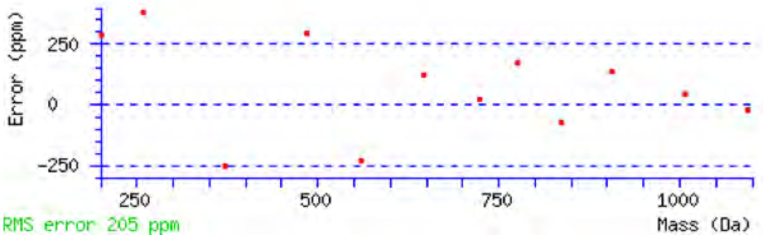

NCBI **BLAST** search of ISGLIYEEVR
(Parameters: blastp, nr protein database, expect=20000, no filter, PAM30)
Other BLAST web gateways

All matches to this query

Score	Mr(calc)	Delta	Sequence
63.1	1205.6656	0.0001	ISGLIYEEVR
17.0	1205.6655	0.0001	QLELYKSNK
17.0	1205.6655	0.0001	QLELYKSNK
17.0	1205.6656	0.0001	KDLLDYAAQK
15.6	1205.6655	0.0001	QKNYIEELK
12.0	1205.6655	0.0001	LEKEFEQKK
12.0	1205.6655	0.0001	LEKEFEQKK
12.0	1205.6656	0.0001	LEKEFEQKK
10.4	1205.6645	0.0012	RSIPSNKIR
10.0	1205.6689	-0.0033	LGMVAASSTSIK

Mascot: http://www.matrixscience.com/

MATRIX SCIENCE Mascot Search Results

Peptide View

MS/MS Fragmentation of **DSVTYTEHAKR**
Found in **H4_YEAST** in **S_cerevisiae_D**, sp|P02309|H4_YEAST Histone H4 OS=Saccharomyces cerevisiae (strain ATCC 204508 / S288c) GN=HHF1 PE=1 SV=2

Match to Query 73923: 1347.640348 from(674.827450,2+) intensity(13994.3370) scans(1876) rawscans(sn1876) rtinseconds(832.2466) index(125048)
Title: 1567: Scan 1876 (rt=13.8708) [D:\MSData\All\VELOS25424.raw]
Data file D:\Data\MGF\531 Final H4 yeast classical PTMs\mascot_daemon_merge.mgf

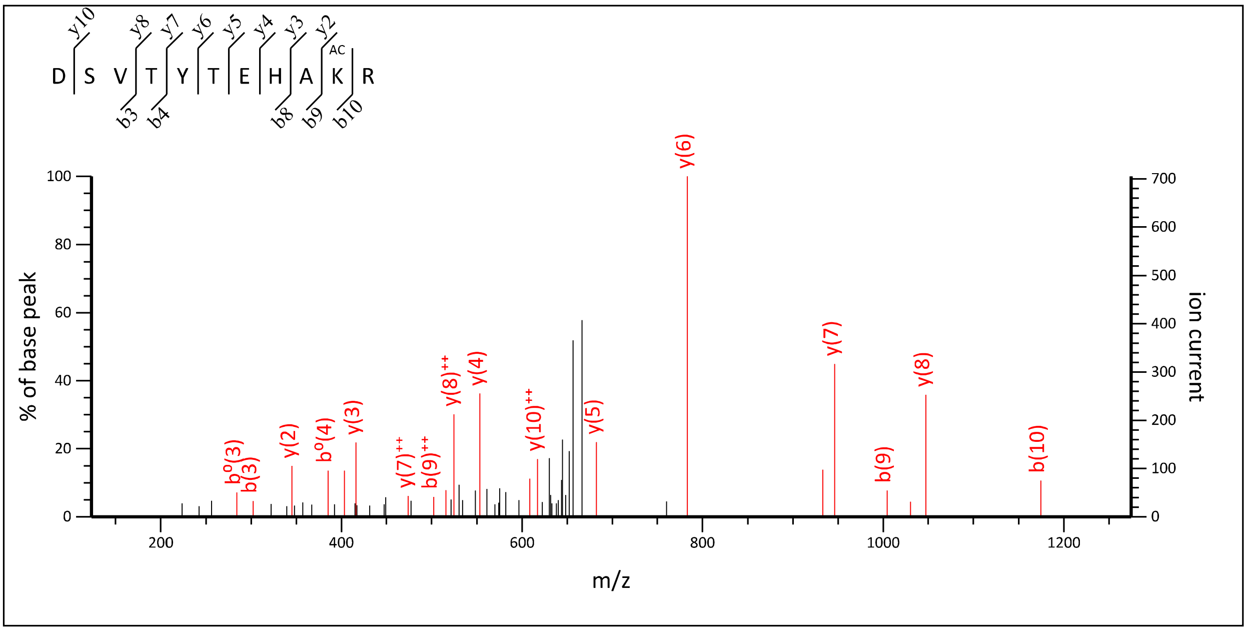

123.18 to 1274.47

Label all possible matches ○ Label matches used for scoring ◉

```
Monoisotopic mass of neutral peptide Mr(calc): 1347.6419
Fixed modifications: Carbamidomethyl (C) (apply to specified residues or termini only)
Variable modifications:
K10     : Acetyl (K)
Ions Score: 57  Expect: 6.9e-05
Matches : 24/94 fragment ions using 28 most intense peaks   (help)
```

#	b	b^{++}	b*	b^{*++}	b^0	b^{0++}	Seq.	y	y^{++}	y*	y^{*++}	y^0	y^{0++}	#
1	116.0342	58.5207			98.0237	49.5155	D							11
2	203.0662	102.0368			185.0557	93.0315	S	1233.6222	**617.3148**	1216.5957	608.8015	1215.6117	608.3095	10
3	**302.1347**	151.5710			284.1241	142.5657	V	1146.5902	573.7987	1129.5637	565.2855	1128.5796	564.7935	9
4	**403.1823**	202.0948			385.1718	193.0895	T	***1047.5218***	**524.2645**	1030.4952	515.7513	1029.5112	515.2592	8
5	566.2457	**283.6265**			548.2351	274.6212	Y	***946.4741***	**473.7407**	929.4476	465.2274	928.4635	464.7354	7
6	667.2933	334.1503			649.2828	325.1450	T	***783.4108***	392.2090	766.3842	383.6958	765.4002	383.2037	6
7	796.3359	398.6716			778.3254	389.6663	E	***682.3631***	341.6852	665.3366	333.1719	664.3525	332.6799	5
8	**933.3949**	467.2011			915.3843	458.1958	H	***553.3205***	277.1639	536.2940	268.6506			4
9	**1004.4320**	**502.7196**			986.4214	493.7143	A	***416.2616***	208.6344	399.2350	200.1212			3
10	**1174.5375**	587.7724	1157.5109	579.2591	1156.5269	578.7671	K	***345.2245***	173.1159	328.1979	164.6026			2
11							R	175.1190	88.0631	158.0924	79.5498			1

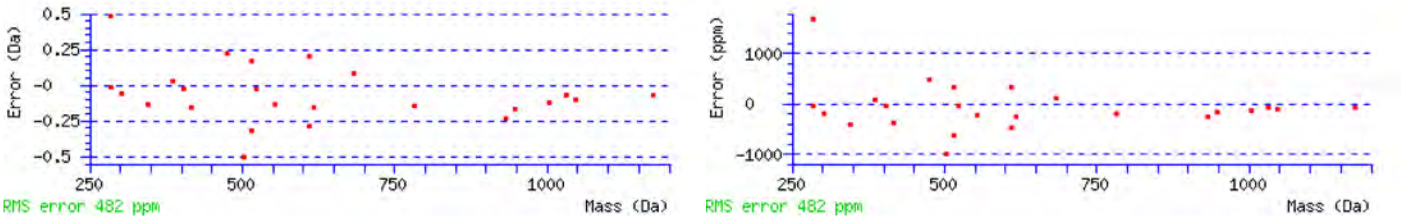

NCBI **BLAST** search of DSVTYTEHAKR
(Parameters: blastp, nr protein database, expect=20000, no filter, PAM30)
Other BLAST web gateways

All matches to this query

Score	Mr(calc)	Delta	Sequence
56.8	1347.6419	-0.0015	DSVTYTEHAKR
11.7	1347.6451	-0.0048	EITKTSKKK
11.3	1347.6353	0.0051	HTPPLNPSKK
10.6	1347.6451	-0.0048	EITKTSKKK
10.4	1347.6340	0.0064	MKNEDEENKK
10.1	1347.6435	-0.0032	ILDPNPESTLR
9.7	1347.6451	-0.0048	IASKSEKTLK
9.7	1347.6451	-0.0048	IASKSEKTLK
9.6	1347.6378	0.0025	TKDAEVNNGSSAR
9.6	1347.6408	-0.0005	SQDRKSHNIR

Mascot: http://www.matrixscience.com/

MATRIX SCIENCE Mascot Search Results

Peptide View

MS/MS Fragmentation of **KTVTSLDVVYALK**
Found in **H4_YEAST** in **S_cerevisiae_D**, sp|P02309|H4_YEAST Histone H4 OS=Saccharomyces cerevisiae (strain ATCC 204508 / S288c) GN=HHF1 PE=1 SV=2

Match to Query 89938: 1477.839948 from(739.927250,2+) intensity(7629.3867) scans(9865) rawscans(sn9865) rtinseconds(2963.6685) index(40642)
Title: 8325: Scan 9865 (rt=49.3945) [D:\MSData\All\VELOS25412.raw]
Data file D:\Data\MGF\531 Final H4 yeast classical PTMs\mascot_daemon_merge.mgf

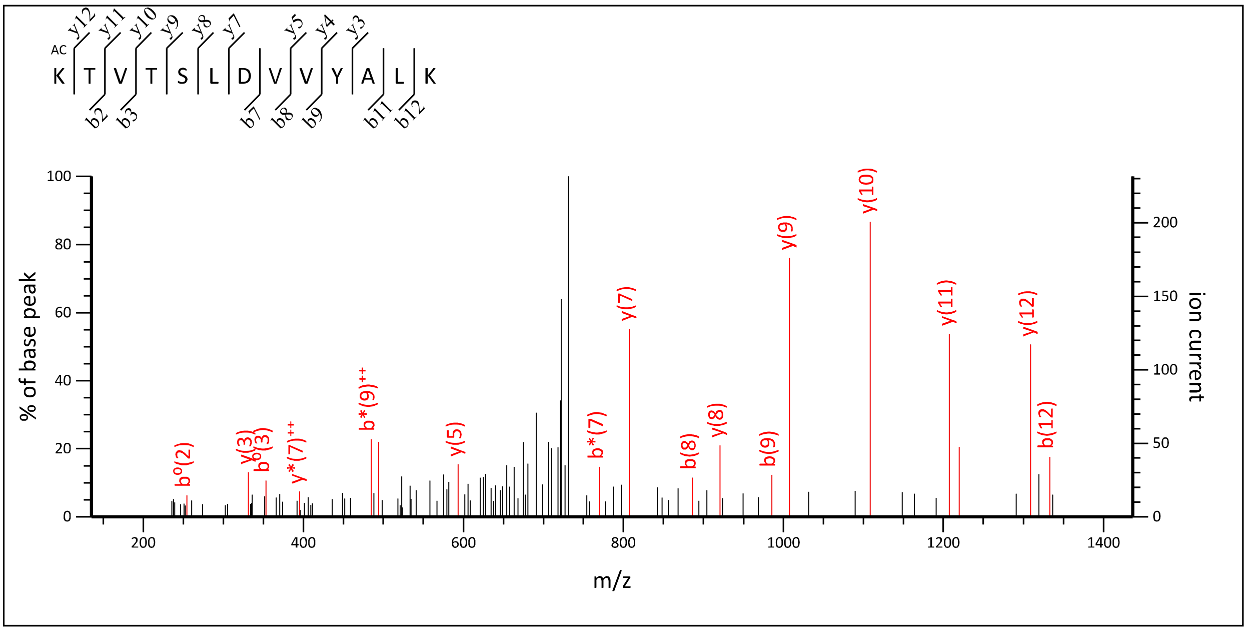

135.2 to 1436.44

Label all possible matches ○ Label matches used for scoring ◉

Monoisotopic mass of neutral peptide Mr(calc): 1477.8392
Fixed modifications: Carbamidomethyl (C) (apply to specified residues or termini only)
Variable modifications:
K1 : Acetyl (K)
Ions Score: 84 **Expect:** 7.6e-07
Matches : 20/130 fragment ions using 23 most intense peaks (help)

#	b	b^{++}	b*	b^{*++}	b^0	b^{0++}	Seq.	y	y^{++}	y*	y^{*++}	y^0	y^{0++}	#
1	171.1128	86.0600	154.0863	77.5468			K							13
2	272.1605	136.5839	255.1339	128.0706	254.1499	127.5786	T	*1308.7409*	654.8741	1291.7144	646.3608	1290.7304	645.8688	12
3	371.2289	186.1181	354.2023	177.6048	353.2183	177.1128	V	*1207.6933*	604.3503	1190.6667	595.8370	1189.6827	595.3450	11
4	472.2766	236.6419	455.2500	228.1287	454.2660	227.6366	T	*1108.6249*	554.8161	1091.5983	546.3028	1090.6143	545.8108	10
5	559.3086	280.1579	542.2821	271.6447	541.2980	271.1527	S	*1007.5772*	504.2922	990.5506	495.7790	989.5666	495.2869	9
6	672.3927	336.7000	655.3661	328.1867	654.3821	327.6947	L	*920.5451*	460.7762	903.5186	452.2629	902.5346	451.7709	8
7	787.4196	394.2134	770.3931	385.7002	769.4090	385.2082	D	*807.4611*	404.2342	790.4345	395.7209	789.4505	395.2289	7
8	**886.4880**	443.7477	869.4615	435.2344	868.4775	434.7424	V	692.4341	346.7207	675.4076	338.2074			6
9	**985.5564**	493.2819	968.5299	484.7686	967.5459	484.2766	V	*593.3657*	297.1865	576.3392	288.6732			5
10	1148.6198	574.8135	1131.5932	566.3002	1130.6092	565.8082	Y	*494.2973*	247.6523	477.2708	239.1390			4
11	**1219.6569**	610.3321	1202.6303	601.8188	1201.6463	601.3268	A	*331.2340*	166.1206	314.2074	157.6074			3
12	**1332.7409**	666.8741	1315.7144	658.3608	1314.7304	657.8688	L	260.1969	130.6021	243.1703	122.0888			2
13							K	147.1128	74.0600	130.0863	65.5468			1

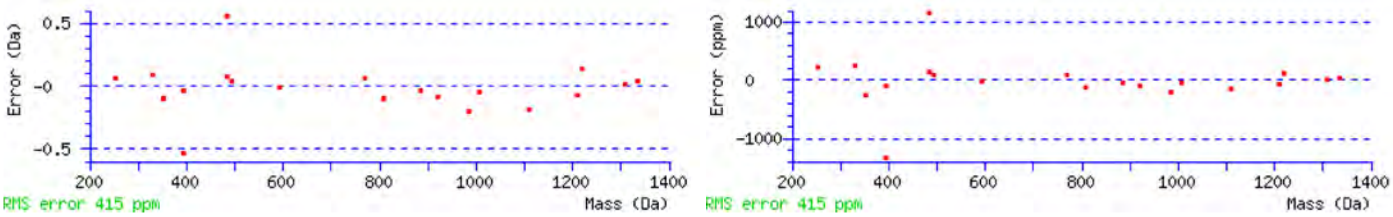

NCBI **BLAST** search of KTVTSLDVVYALK
(Parameters: blastp, nr protein database, expect=20000, no filter, PAM30)
Other BLAST web gateways

All matches to this query

Score	Mr(calc)	Delta	Sequence
83.6	1477.8392	0.0008	KTVTSLDVVYALK
22.0	1477.8405	-0.0006	DKVTAWKQYRK
18.0	1477.8405	-0.0005	SRKSYPPVYRK
17.9	1477.8405	-0.0006	SRKSYPPVYRK
16.9	1477.8405	-0.0005	SRKSYPPVYRK
15.3	1477.8405	-0.0006	DKVTAWKQYRK
14.8	1477.8405	-0.0006	DKVTAWKQYRK
14.6	1477.8381	0.0018	KTAKTPSKKRK
14.4	1477.8381	0.0018	RLSTISNKLPKK
13.3	1477.8381	0.0018	KTAKTPSKKRK

Mascot: http://www.matrixscience.com/

Mascot Search Results

Peptide View

MS/MS Fragmentation of **TVTSLDVVYALK**
Found in **H4_YEAST** in **S_cerevisiae_D**, sp|P02309|H4_YEAST Histone H4 OS=Saccharomyces cerevisiae (strain ATCC 204508 / S288c) GN=HHF1 PE=1 SV=2

Match to Query 74388: 1349.744548 from(675.879550,2+) intensity(6512.7119) scans(9988) rawscans(sn9988) rtinseconds(2994.029) index(131981)
Title: 8500: Scan 9988 (rt=49.9005) [D:\MSData\All\VELOS25424.raw]
Data file D:\Data\MGF\531 Final H4 yeast classical PTMs\mascot_daemon_merge.mgf

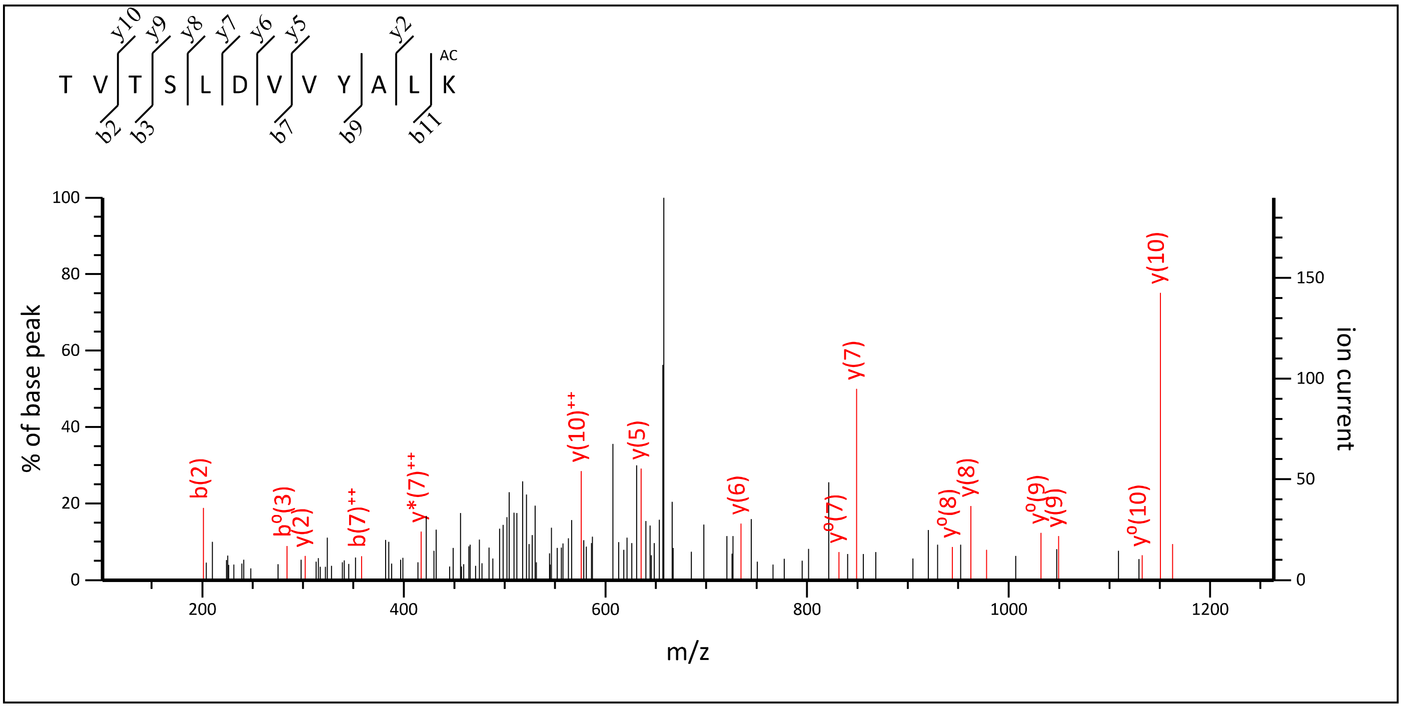

101.18 to 1262.75

Label all possible matches Label matches used for scoring

Monoisotopic mass of neutral peptide Mr(calc): 1349.7442
Fixed modifications: Carbamidomethyl (C) (apply to specified residues or termini only)
Variable modifications:
K12 : Acetyl (K)
Ions Score: 23 **Expect:** 0.14
Matches : 21/98 fragment ions using 57 most intense peaks (help)

#	b	b^{++}	b^0	b^{0++}	Seq.	y	y^{++}	y*	y^{*++}	y^0	y^{0++}	#
1	102.0550	51.5311	84.0444	42.5258	T							12
2	***201.1234***	101.0653	183.1128	92.0600	V	1249.7038	625.3556	1232.6773	616.8423	1231.6933	616.3503	11
3	***302.1710***	151.5892	284.1605	142.5839	T	***1150.6354***	575.8213	1133.6089	567.3081	1132.6249	566.8161	10
4	389.2031	195.1052	371.1925	186.0999	S	***1049.5877***	525.2975	1032.5612	516.7842	1031.5772	516.2922	9
5	502.2871	251.6472	484.2766	242.6419	L	***962.5557***	481.7815	945.5292	473.2682	944.5451	472.7762	8
6	617.3141	309.1607	599.3035	300.1554	D	***849.4716***	425.2395	832.4451	416.7262	831.4611	416.2342	7
7	716.3825	358.6949	698.3719	349.6896	V	***734.4447***	367.7260	717.4182	359.2127			6
8	815.4509	408.2291	797.4403	399.2238	V	***635.3763***	318.1918	618.3497	309.6785			5
9	***978.5142***	489.7608	960.5037	480.7555	Y	536.3079	268.6576	519.2813	260.1443			4
10	***1049.5514***	525.2793	1031.5408	516.2740	A	373.2445	187.1259	356.2180	178.6126			3
11	***1162.6354***	581.8213	1144.6249	572.8161	L	***302.2074***	151.6074	285.1809	143.0941			2
12					K	189.1234	95.0653	172.0968	86.5520			1

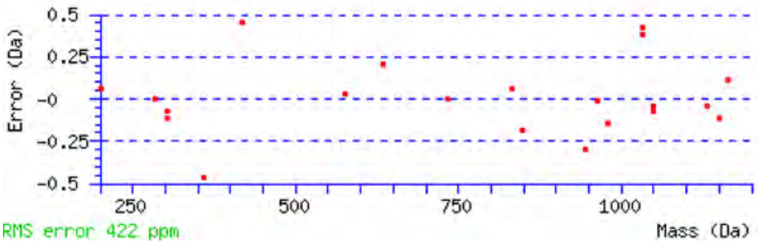

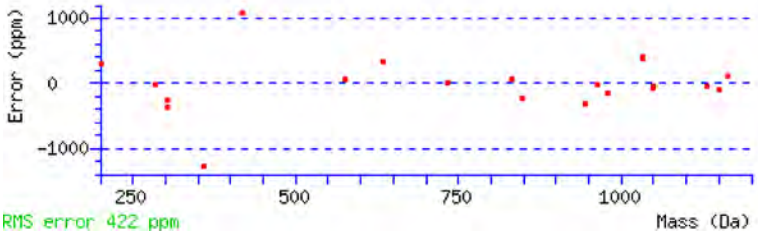

NCBI **BLAST** search of TVTSLDVVYALK
(Parameters: blastp, nr protein database, expect=20000, no filter, PAM30)
Other BLAST web gateways

All matches to this query

Score	Mr(calc)	Delta	Sequence
22.7	1349.7442	0.0003	TVTSLDVVYALK
12.8	1349.7415	0.0030	KRRFSTGESLK
10.1	1349.7431	0.0014	SPRTAKKAEIK
9.0	1349.7455	-0.0010	RFANWSSISIK
8.7	1349.7431	0.0014	SPRTAKKAEIK
8.5	1349.7393	0.0052	MPLTTKPLSLK
8.5	1349.7393	0.0052	MPLTTKPLSLK
7.8	1349.7431	0.0014	SIVLRKSNKK
7.8	1349.7431	0.0014	SIVLRKSNKK
6.4	1349.7402	0.0044	TSSITSSKKSLK

Mascot: http://www.matrixscience.com/
